# A Genetically Encoded Electrophilic Lysine Derivative Enables Sortase-Mediated Assembly of SUMO Activity-Based Probes

**DOI:** 10.1021/jacs.6c10081

**Published:** 2026-08-12

**Authors:** Vera Wanka, Denys Kvasha, Marko Cigler, Kristina Heymes-Krauskopf, Philipp Ruckgaber, Anna Heider, Maximilian Fottner, Michael Groll, Kathrin Lang

**Affiliations:** † Laboratory for Organic Chemistry (LOC), Department of Chemistry and Applied Biosciences (D-CHAB), 31064 ETH Zurich, 8093 Zurich, Switzerland; ‡ Department of Chemistry, Technical University of Munich, 85748 Garching, Germany; § TUM School of Natural Sciences, Department of Bioscience, Center for Protein Assemblies, Technical University of Munich, Garching 85748, Germany

## Abstract

Activity-based probes (ABPs) have become powerful tools for profiling enzymes that write and erase ubiquitin (Ub) and ubiquitin-like modifier (Ubl) signals. Extending this strategy to small ubiquitin-like modifier (SUMO)-specific proteases in defined substrate contexts remains challenging, because site-specific SUMO attachment must be combined with precise electrophile placement near the scissile isopeptide linkage. Here, we introduce a sortase-enabled chemoenzymatic platform for generating SUMO ABPs ranging from monoSUMO probes to native-like SUMO-substrate conjugates. The engineered sortase Srt2A ligates SUMO variants to glycine-bearing electrophiles, providing facile access to monoSUMO probes that trap deSUMOylases in vitro, in cellular lysates, and in living cells. To generate SUMO-substrate probes, we develop AzGVAisoK, a genetically encodable bifunctional lysine derivative containing both an azide-protected sortase handle and a vinyl amide electrophile. An engineered pyrrolysyl-tRNA synthetase/tRNA pair enables its site-specific incorporation into target proteins. Subsequent on-protein Staudinger reduction and sortase-mediated SUMOylation furnish defined SUMO-substrate ABPs under mild aqueous conditions. Applying this platform to PCNA and K11-linked diSUMO conjugates revealed distinct deSUMOylase trapping profiles governed by SUMO paralog and acceptor-substrate contexts. This work establishes a modular route to native-like SUMO probes and provides a general strategy for interrogating context-dependent enzyme recognition in Ubl signaling.

## Graphical abstract

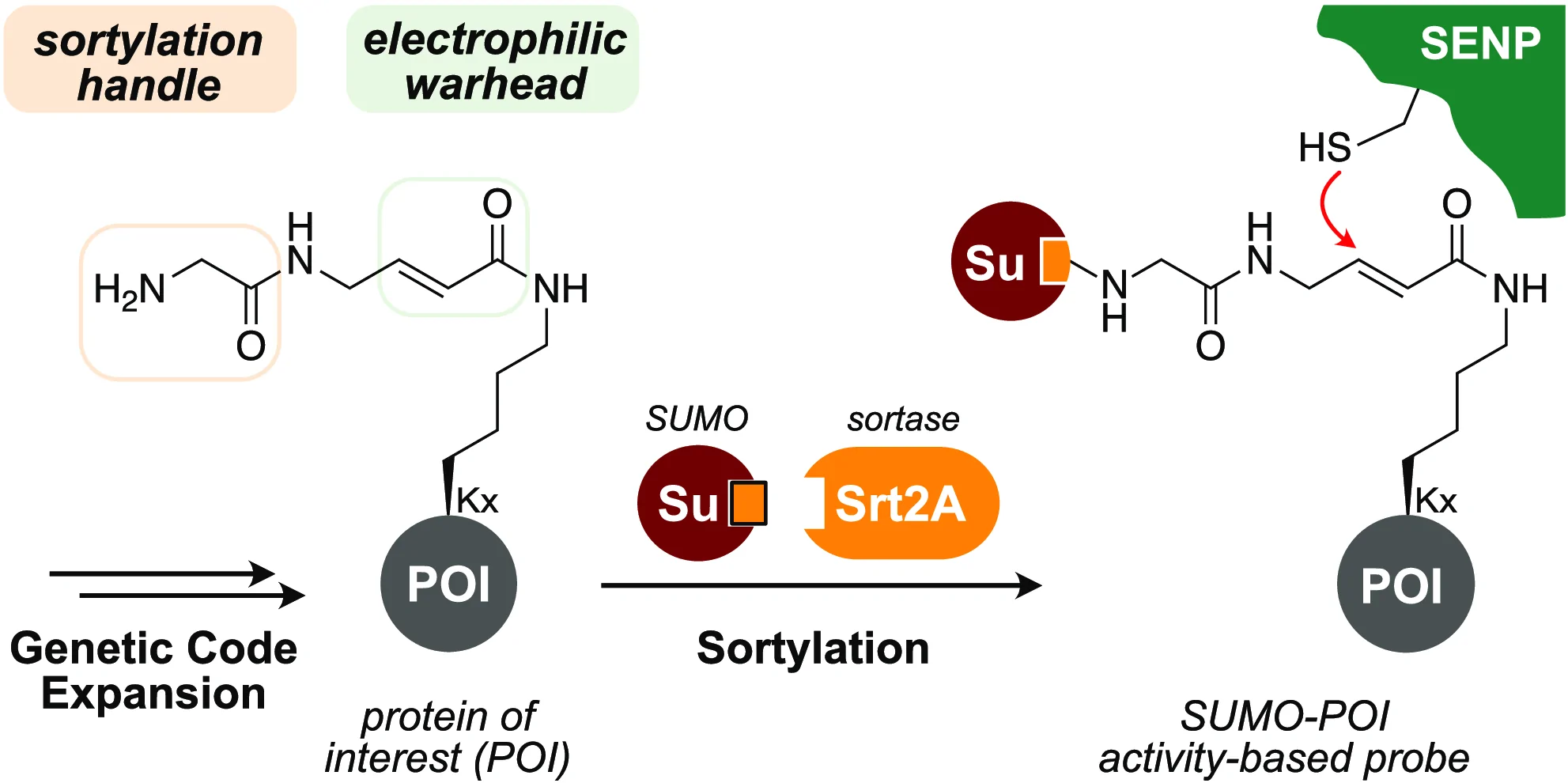

## Introduction

Modification of proteins with ubiquitin (Ub) and ubiquitin-like modifiers (Ubls) constitutes a central posttranslational mechanism that regulates protein function, localization, stability and interactions in eukaryotic cells.^1,2^ Among Ubls, the small ubiquitin-like modifier (SUMO) is conjugated to more than a thousand target proteins and controls numerous nuclear and cytoplasmic processes, including DNA replication, DNA damage response, chromatin organization, transcription, ribosome biogenesis and proteostasis.^3–5^ Human SUMOylation mainly involves attachment of SUMO1, SUMO2 or SUMO3 paralogs through their C-termini to specific lysine residues in target proteins forming an isopeptide bond. While all three paralogs can modify substrates as monomers, the closely related SUMO2/3 paralogs are prone to form polymeric chains, most prominently through K11, whereas the more divergent SUMO1 paralog is mainly found as a monomeric modifier or as a chain-terminating cap on SUMO2/3 polymers.^6–8^ Like ubiquitylation, SUMOylation is highly dynamic. It is reversed by active-site cysteine-dependent deSUMOylases, including SENP1, SENP2, SENP3, SENP5, SENP6, SENP7, DeSi1/2 and USPL1, which regulate SUMO maturation, substrate deconjugation, and SUMO-chain editing.^9,10^ Precise control of SUMO conjugation and deconjugation is essential, as dysregulation of these pathways is linked to various diseases including different types of cancer.^11–13^ Some of these links, such as the correlation between high SENP1 levels and c-Myc stabilization in breast cancer, are already known,^11^ yet, a comprehensive understanding of SENPs and their respective substrates in disease is still lacking. However, chemical tools that resolve these layers of specificity, particularly in native-like SUMO-substrate conjugates, remain limited.^12,13^

Chemical biology has provided powerful strategies to study Ub/Ubl signaling. Defined Ub/Ubl-protein-of-interest (Ub/Ubl-POI) conjugates enable analysis of reader interactions and substrate-dependent recognition, fluorogenic Ub/Ubl substrates allow kinetic monitoring of protease activity, and activity-based probes (ABPs) covalently trap catalytically active enzymes involved in conjugation/deconjugation.^14–19^ Ub/Ubl-based ABPs typically combine a Ub/Ubl recognition element, a detection or enrichment tag and an electrophilic warhead positioned to react with active-site cysteines present in many Ub/Ubl-conjugating and deconjugating enzymes. MonoUb/Ubl ABPs, in which the warhead is installed at the modifier C-terminus, have been widely used to discover and profile activity of deubiquitylases and Ubl proteases.^20–24^ However, because these probes present only the Ub/Ubl modifier, they cannot fully capture recognition elements contributed by the modified substrate or by higher-order SUMO architectures. More complex diUb/Ubl or Ub/Ubl-POI conjugate ABPs place the electrophile close to the isopeptide linkage and can therefore report on linkage- and substrate-dependent enzyme engagement.^14,16,17^ Access to such probes remains technically demanding. Existing approaches based on solid-phase peptide synthesis (SPPS), native chemical ligation, intein-derived thioesters, or thiol-elimination chemistry are powerful but restrict substrate scope.^25–29^ These limitations are particularly pronounced for Ubls such as SUMO, whose larger size can complicate probe synthesis, and for Ub/Ubl-POI conjugate ABPs, where site-specific modifier attachment must be combined with precise warhead installation near the isopeptide linkage. As a result, access to substrate-mimetic SUMO probes remains especially challenging for large, folded, multimeric, or cysteine-containing proteins.

To overcome these limitations, we built on a chemoenzymatic approach previously developed in our group, in which the engineered sortase variant Srt2A^30^ recognizes an LAXTG motif introduced into the C-terminus of Ub/Ubls and ligates it to a genetically encoded isopeptide linked diglycine handle installed within a target protein.^31,32^ Termed sortylation (Figure 1a), this strategy yields site-specific Ub/Ubl-POI conjugates that feature a native isopeptide linkage at the modified lysine and closely mimic their endogenous counterparts while avoiding total chemical protein synthesis and protein refolding. We reasoned that sortylation could be repurposed from conjugate assembly to ABP construction if an electrophilic warhead is introduced either at the SUMO C-terminus or within the linker connecting SUMO to a target protein. In doing so, sortylation could be extended beyond the generation of Ub/Ubl conjugates to provide substrate-mimetic SUMO-POI probes for covalent deSUMOylase capture.

**Figure 1:**
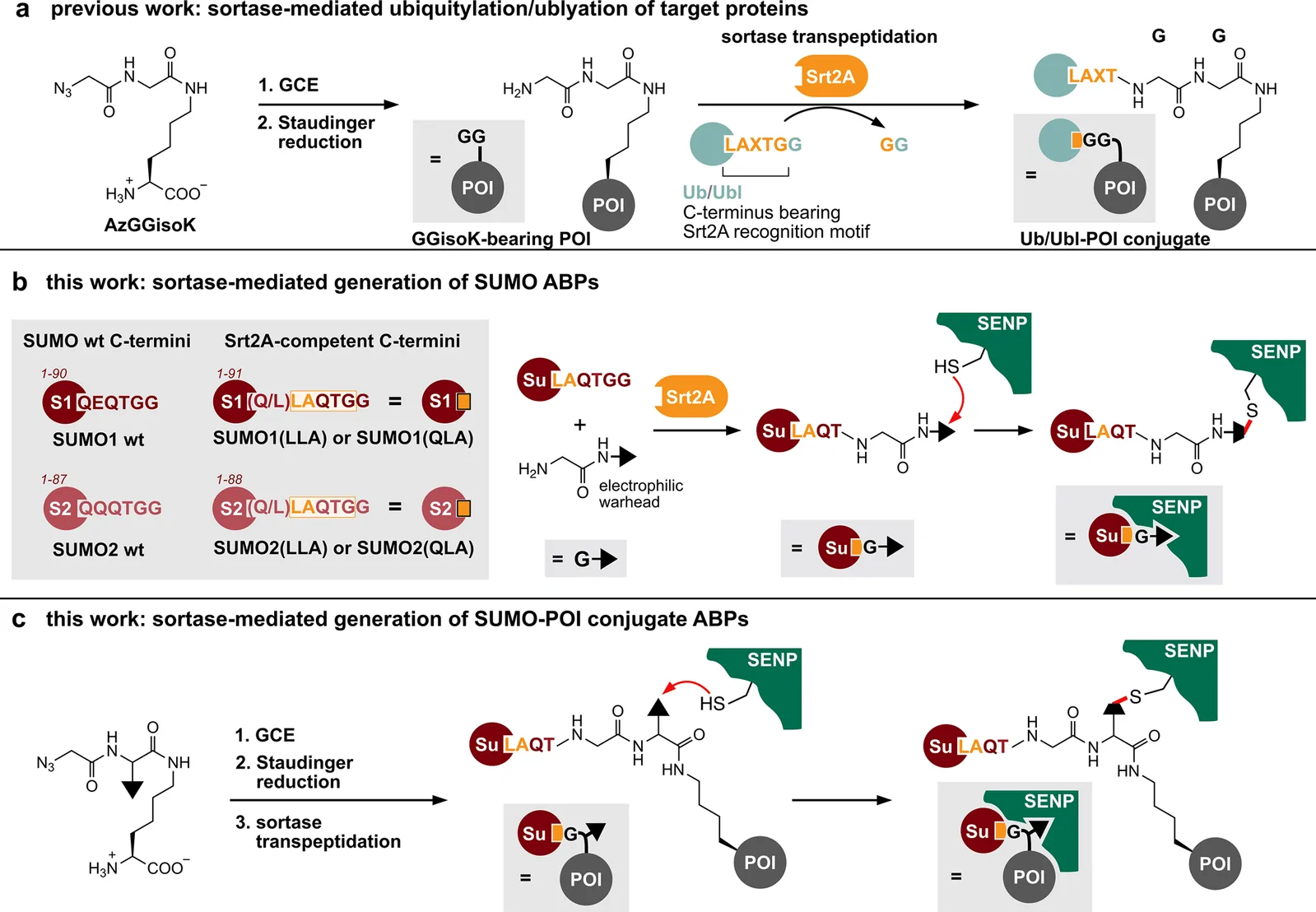
Sortase-mediated generation of Ub/Ubl-POI conjugates and SUMO-based ABPs. (a) The ncAA AzGGisoK is site-specifically incorporated into POIs by GCE and reduced via phosphine treatment to GGisoK on protein. GGisoK serves as transpeptidation handle for Srt2A-mediated attachment of a modified Ub/Ubl that presents the Srt2A recognition motif LAXTG within its C-terminus. Sortylation thereby grants access to defined, site-specific and isopeptidically linked Ub/Ubl-POI conjugates. (b) Compared to wt SUMO1/2, the Srt2A-compatible SUMO1/2­(LLA) and SUMO1/2­(QLA) variants contain point mutations at the fifth- and sixth-to-last positions (highlighted in yellow) that introduce the Srt2A recognition motif LAXTG (yellow box), together with an inserted leucine or glutamine spacer residue (indicated in brackets) to enhance motif accessibility. These engineered variants undergo Srt2A-catylzed transpeptidation with glycine-bearing electrophilic warheads (black tringle), yielding monoSUMO ABPs that covalently react with the active site cysteine of SENPs. (c) Incorporation of an AzGGisoK derivative bearing an electrophilic warhead instead of the internal glycine moiety enables access to Srt2A-generated SUMO­((Q/L)­LA)-POI conjugate ABPs for profiling substrate-specific SENPs. As this approach works under mild, physiological conditions it is compatible with POIs beyond small, monomeric, refoldable or cysteine-free target proteins.

We implement this concept in two stages. First, we show that Srt2A-compatible SUMO variants can be ligated to glycine-bearing electrophiles to produce monoSUMO ABPs (Figure 1b). These probes covalently capture deSUMOylases in an activity-dependent manner, function in cellular lysates, and can be assembled in cellulo. We then extend the approach to SUMO-POI ABPs by designing a bifunctional noncanonical amino acid (ncAA) that combines an azide-protected glycine handle for Srt2A-mediated SUMOylation with a Michael acceptor warhead for deSUMOylase trapping (Figure 1c). An engineered pyrrolysyl-tRNA synthetase (PylRS)/tRNA pair enables site-specific incorporation of this ncAA into target proteins, and subsequent sortylation furnishes SUMO-POI ABPs with the electrophile positioned near the native isopeptide bond. Profiling these probes against deSUMOylases reveals distinct interaction patterns that depend on SUMO paralog and acceptor substrate. Together, this work introduces a genetically programmable chemoenzymatic strategy for generating monoSUMO and SUMO-POI ABPs and enables deSUMOylase activity to be interrogated in defined paralog- and substrate-specific contexts.

## Results

### Sortase-Mediated Ligation Generates Functional monoSUMO ABPs

To create SUMO-based probes via Srt2A-mediated transpeptidation, we installed the Srt2A recognition motif LAQTG within the C-termini of SUMO1 and SUMO2 by introducing two point mutations. Accessibility of this recognition motif was enhanced by inserting either leucine or glutamine immediately upstream of the motif, affording SUMO1/2­(LLA) and SUMO1/2­(QLA) variants, respectively (Figure 1b). Importantly, these modifications preserve the native C-terminal QTGG sequence recognized by SUMO-processing enzymes, and despite their reduced processing efficiency relative to wt SUMO, the engineered variants remained substrates for the major SUMO proteases SENP1 and SENP2 (Figure S1).

We first asked whether SUMO1/2­(LLA) variants could be conjugated to small-molecule electrophiles containing an N-terminal glycine and a C-terminal electrophilic warhead. Such glycine-bearing warheads are straightforward to synthesize and are expected to be sufficiently stable in complex biological environments. Furthermore, Srt2A-mediated ligation to the SUMO C-terminus is expected to position the warhead in close proximity and optimal orientation to react with active-site cysteine residues of deSUMOylating enzymes (Figure 1b). We evaluated three electrophiles commonly used in Ub-based ABPs: the propargylamide (PA)^33^ warhead and the Michael acceptors vinyl methyl ester (VME)^20^ and the vinyl amide^25,34,35^ derivative vinyl dimethyl amide (VDMA). The corresponding glycine-derivatized compounds (GPA, GVME and GVDMA, Figure 2a–c) all reacted with SUMO2­(LLA) upon incubation with Srt2A at 37 °C as judged by liquid chromatography–mass spectrometry (LC-MS) analysis (Figure S2). Reactions with GPA and GVDMA proceeded quantitatively within 1–3 h, but the increased electrophilicity of GVME resulted in side-product formation (+37 Da) upon prolonged incubation (Figure S2c). Consistent with this higher intrinsic reactivity, only SUMO2­(LLA)-VME formed adducts with glutathione (GSH, 200 equiv) (Figure S3), whereas SUMO2­(LLA)-PA and SUMO2­(LLA)-VDMA remained stable, indicating that the latter probes are better suited for in cellulo applications.

**Figure 2:**
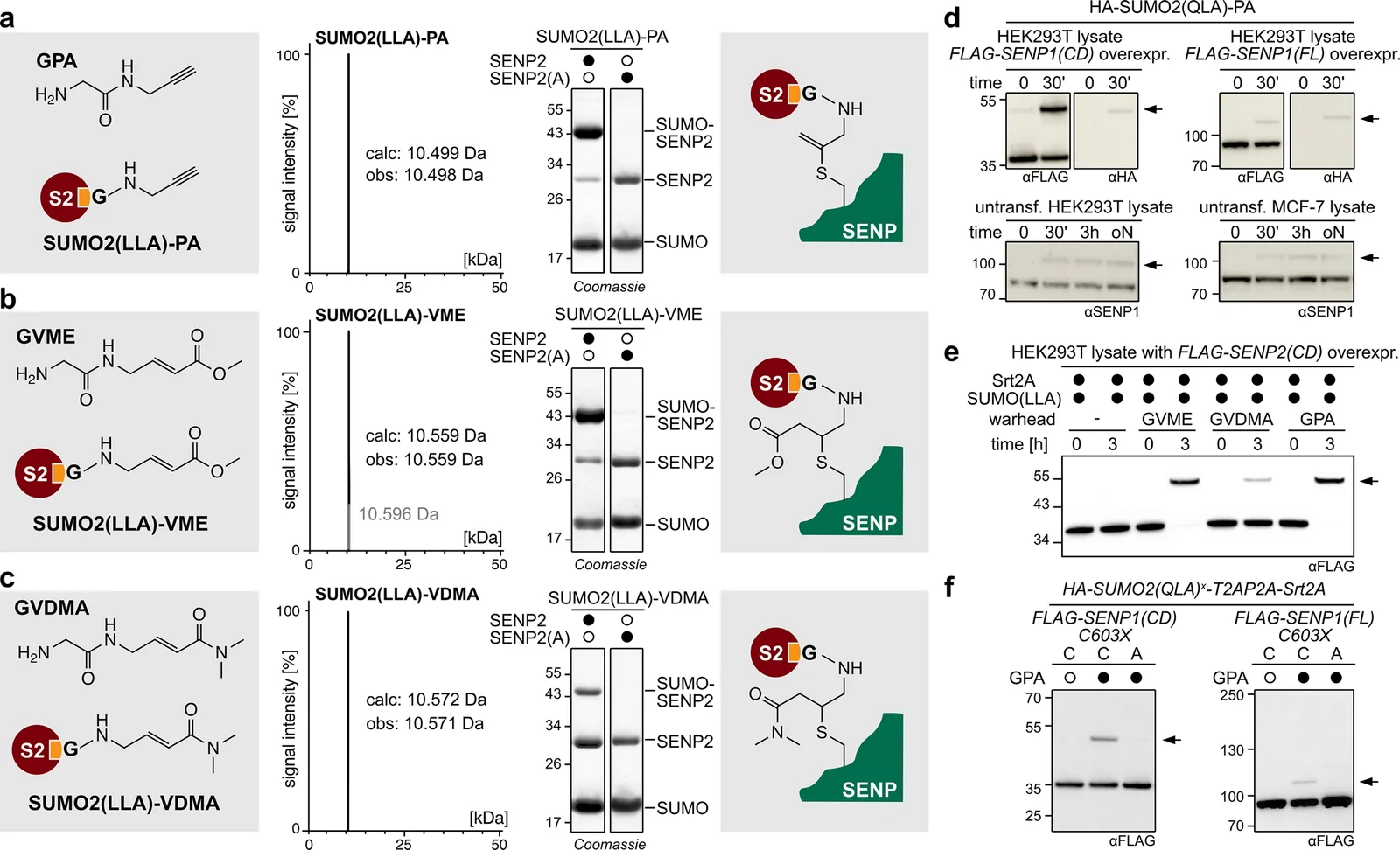
Sortase-mediated generation of monoSUMO ABPs in vitro, in lysates and intracellularly. (a–c) Srt2A-mediated ligation of 5 mM GPA, GVME, or GVDMA and 100 μM SUMO2­(LLA) at 37 °C affords the ABPs SUMO2­(LLA)-PA (M calc: 10.499 Da, obs: 10.498 Da), SUMO2­(LLA)-VME (M calc: 10.559 Da, obs: 10.559 Da and minor 10.596 Da) and SUMO2­(LLA)-VDMA (M calc: 10.572 Da, obs: 10.571 Da). Incubation of 50 μM ABP with 15 μM SENP2­(CD) at 37 °C leads to covalent SENP trapping while the active site mutant SENP2­(CD, C548A) remains unmodified. Uncropped gels can be found in Figure S4. (d) Incubation of 5 μM preassembled HA-SUMO2­(QLA)-PA with cellular extracts prepared from HEK293T that overexpress FLAG-SENP1­(CD) (top left, total protein concentration in lysate 4 mg/mL) or FLAG-SENP1­(FL) (top right, total protein concentration in lysate 4 mg/mL) or lysates prepared from untransfected HEK293T cells (bottom left, total protein concentration in lysate 5 mg/mL) or untransfected MCF7 cells (bottom right, total protein concentration in lysate 2 mg/mL) leads to covalent SENP trapping (indicated by black arrow) as judged by Western blot analysis. Uncropped Western Blots and further controls are shown in Figure S9 and S10. (e) Incubation of HEK293T lysate prepared from cells that overexpressed FLAG-SENP2­(CD) with 100 μM SUMO2­(LLA), 20 μM Srt2A and 5 mM GVME, GVDMA, or GPA at 37 °C permits one-pot assembly and application of monoSUMO ABPs. Warhead-dependent SENP-trapping was confirmed by Western blot analysis (indicated by black arrow). Uncropped WBs are shown in Figure S11. (f) In cellulo assembly of SUMO2­(QLA)-PA is achieved by transfecting HEK293T cells with HA-SUMO2­(QLA)^x^-T2AP2A-Srt2A and subsequent treatment with 0.5 mM GPA for 2 h (starting 40 h after transfection). Western blot against overexpressed FLAG-SENP1­(CD) or FLAG-SENP1­(FL) shows that ABP assembly and subsequent SENP trapping (indicated by black arrow) are dependent on GPA treatment and presence of the SENP1 active site cysteine; experiment was performed in triplicate. Uncropped Western Blots are shown in Figure S12.

Incubation of the purified ABPs SUMO2­(LLA)-PA, SUMO2­(LLA)-VME, and SUMO2­(LLA)-VDMA with the catalytic domain of SENP2 (SENP2­(CD)) at 37 °C resulted in robust trapping of the wild-type (wt) enzyme, but not the active site mutant SENP2­(CD, C548A), confirming the activity and specificity of all three ABPs (Figures 2a–c and S4). Among the two Michael acceptor probes, SUMO2­(LLA)-VME showed higher trapping efficiency than the VDMA-bearing probe, but also reduced specificity, as evidenced by trace-level labeling of the SENP2­(CD, C548A) active-site mutant (Figure 2b), consistent with its observed reactivity toward GSH (Figure S3). In contrast, SUMO2­(LLA)-PA combined efficient trapping with high specificity (Figures 2a, S3, and S4) and was therefore selected for further investigation.

To better mimic the native SUMO C-terminus, we replaced the leucine spacer in SUMO­(LLA) with glutamine, generating SUMO­(QLA) (Figure 1b). Unexpectedly, this modification also accelerated Srt2A-mediated ligation (Figure S5). We subsequently generated N-terminally HA-tagged SUMO1­(QLA)-PA and SUMO2­(QLA)-PA probes via Srt2A-mediated ligation (Figure S6) and profiled them against a panel of deSUMOylating enzymes (Figure S7a,b). The catalytic domains of SENP2, SENP6, SENP7 and USPL1 displayed preferential labeling by the SUMO2 probe compared to the SUMO1 probe, whereas SENP1­(CD) and SENP5­(CD) showed no pronounced paralog preference. Neither probe reacted with DeSi1 and no labeling was observed for the negative control SENP8, a deNEDDylase (Figure S7c).

Because introduction of the Srt2A recognition motif necessarily alters the SUMO C-terminal sequence, we benchmarked the sortase-generated probes against wt HA-SUMO-PA probes prepared by intein chemistry^20,36^ (Figure S8). Although overall SUMO:deSUMOylase complex formation was more efficient for wt ABPs, comparative assays largely confirmed the preferences observed for the Srt2A-generated ABPs with only minor differences (Figure S7). The most notable deviation was seen for SENP7 that was labeled by wt SUMO1-PA but not by the sortase-generated SUMO1­(QLA)-PA probe (Figure. S7). SENP6 and SENP7 are SUMO2/3-biased enzymes involved in polySUMO2/3-chain editing and their preferential labeling by our SUMO2 probe aligns with the described selectivity.^37,38^ SENP2 and the atypical SUMO protease USPL1 have been reported to favor SUMO2/3 substrates,^39,40^ consistent with their enhanced trapping by SUMO2­(QLA)-PA in this assay. In contrast, SENP1 displays broader activity,^39^ in agreement with the lack of discernible paralog selectivity observed with the sortase-generated probes. Finally, DeSi1 has primarily been described as a SUMO isopeptidase with limited SUMO-processing activity.^41^ Accordingly, the absence of labeling by our SUMO1 or SUMO2 probes was not unexpected. Together, these data show that Srt2A-mediated ligation provides rapid access to functional monoSUMO ABPs whose trapping profiles largely preserve known deSUMOylase preferences.

### MonoSUMO ABPs Can Be Generated and Applied in Lysates and in Cellulo

Encouraged by the efficient and selective in vitro trapping with the Srt2A-generated monoSUMO probes, we next evaluated their performance in cellular extracts. Treatment of HEK293T lysates overexpressing either FLAG-tagged SENP1­(CD) or full-length SENP1­(FL) with HA-SUMO2­(QLA)-PA resulted in robust SENP1 trapping after 30 min of incubation (Figures 2d and S9), whereas the corresponding active-site mutants remained unmodified (Figure S9). Endogenous SENP1 was also labeled in lysates of untransfected HEK293T and MCF-7 cells upon treatment with HA-SUMO2­(QLA)-PA as detected by anti-SENP1 Western-blotting (Figures 2d and S10).

We next explored whether probe generation and deSUMOylase trapping could be performed in a single step. As an initial proof-of-concept, purified SUMO2­(LLA), Srt2A and SENP2­(CD) were incubated with either GPA or triglycine as a negative control. Efficient SENP2 trapping was observed only in the presence of GPA, consistent with in situ generation of the PA-containing probe (Figure S11a). Notably, one-pot assembly and application of SUMO2­(LLA)-based probes proved also effective in complex biological environments. Incubation of lysates prepared from HEK293T cells overexpressing FLAG-SENP2­(CD) with SUMO2­(LLA), Srt2A and GVME, GVDMA or GPA at 37 °C for 3 h led to warhead-dependent SENP2 trapping, whereas no covalent complex formation was observed in the absence of a glycine-bearing electrophile (Figures 2e and S11b). Under these conditions in situ generated SUMO2­(LLA)-PA and SUMO2­(LLA)-VME trapped SENP2­(CD) nearly quantitatively, whereas SUMO2­(LLA)-VDMA showed lower trapping efficiency, as detected by anti-FLAG Western blotting. Overall, trapping efficiencies closely mirrored those obtained with preassembled probes (Figure 2a–c), indicating that SUMO-ABPs can be generated in situ in lysates.

Finally, we evaluated whether monoSUMO probes could be assembled in living cells.^42^ HEK293T cells were cotransfected with plasmids encoding for FLAG-tagged SENP1 and SUMO2­(QLA)^x^-T2AP2A-Srt2A, followed by GPA treatment (0.5 mM) for 2 h, starting 40 h post transfection. SENP1 trapping was assessed by anti-FLAG Western blotting (Figures 2f and S12). For both SENP1­(CD) and SENP1­(FL), complex formation was observed exclusively upon GPA treatment, but not in untreated cells or with the active-site mutant of SENP1. These results demonstrate that HA-SUMO­(QLA)-PA probes can be assembled in cellulo from expressed SUMO/Srt2A components and a cell-permeable glycine electrophile.

### Genetic Encoding of an Electrophilic Lysine Derivative for SUMO-POI ABP Generation

While monoSUMO ABPs report on modifier-dependent deSUMOlyase engagement, they cannot report on substrate- and conjugation site-specific deSUMOylase recognition. To address this, we aimed to adapt our sortase-based ABP-generation strategy to produce SUMO-POI conjugates in which an electrophile is positioned near the scissile isopeptide bond, i.e., within the linker between SUMO and the POI. Building on sortylation^31,32^ (Figure 1a), we envisioned combining Srt2A-mediated ligation with genetic code expansion (GCE) to install an electrophilic functionality site-specifically into target proteins (Figure 1c).

To design a sortylation-compatible lysine derivative containing both a sortase-reactive handle and an internal electrophile, we built on the warhead comparison performed with the GPA-, GVME- and GVDMA-based monoSUMO probes (Figure 2a–c). Electrophile stability is crucial for ncAA incorporation during recombinant protein expression and purification. Our in vitro GSH stability assays with assembled monoSUMO ABPs indicated that VME is overly reactive, whereas PA and VDMA-bearing probes remain stable over extended incubation times (Figure S3). Although the PA-bearing probe provides the most favorable combination of trapping efficiency and specificity in the monoSUMO format, installation of a propargylamide warhead within a genetically encodable, sortylation-compatible lysine scaffold is not straightforward. We therefore selected a vinyl amide electrophile, which combines the GSH stability observed for the VDMA-based monoSUMO probe with the established utility of vinyl amides in ABP design.^34,35^ The resulting ncAA, GVAisoK, contains a glycine that can serve as the nucleophile in Srt2A-mediated transpeptidation and is connected to the lysine N^ε^ through a vinyl amide-containing linker (Figure 3a).

**Figure 3:**
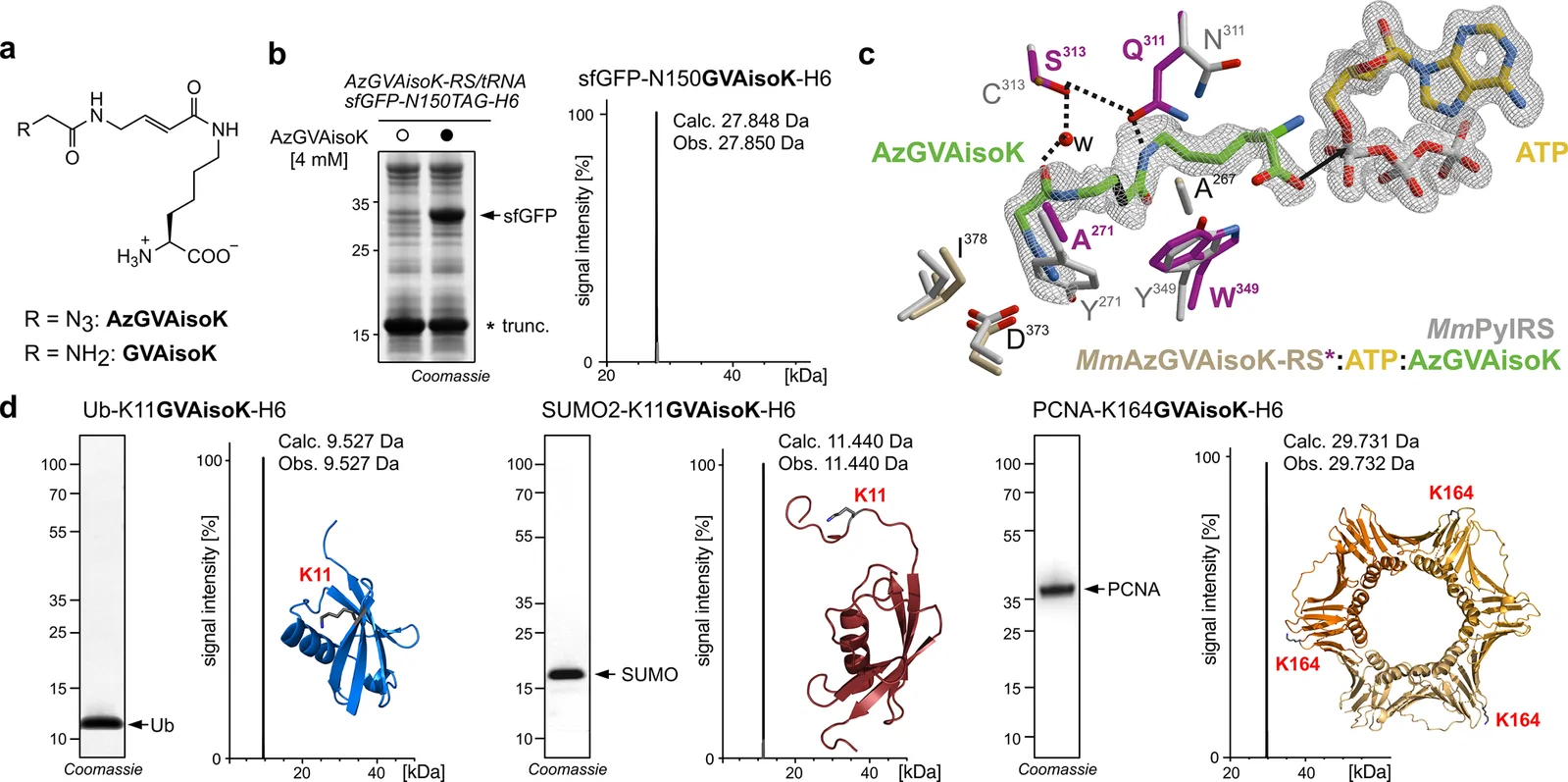
Site-specific incorporation of the vinyl amide bearing ncAA GVAisoK into POIs via GCE with an engineered Pyl-RS/tRNA variant. (a) The newly designed ncAA AzGVAisoK is suitable for amber suppression and on-demand Staudinger reduction to GVAisoK that should undergo sortylation. (b) SDS-PAGE analysis of *E. coli* K12 cotransformed with plasmids encoding *Mb*AzGVAisoK-RS (pBK) and an amber stop codon-bearing sfGFP­(N150TAG)-H6 as well as *Mm*PylT (pPylT) cultured in absence or presence of 4 mM AzGVAisoK in 14 aa autoinducing (AI) medium shows ncAA-dependent amber suppression without misincorporation of endogenous amino acids. NiNTA protein purification in the presence of TCEP affords sfGFP­(N150GVAisoK)-H6 as confirmed by LC-MS (M calc: 27.848 Da, obs: 27.850 Da). (c) Active site of *Mm*AzGVAisoK (*Mb* numbering used throughout for continuity) with Polder mF_o_-DF_c_ omit map contoured at 3.0 σ (gray mesh) for ATP and AzGVAisoK (PDB ID: 30XL). The black arrow indicates the site of nucleophilic attack of the ligand carboxylate on the ATP α-phosphate. Superposition with wt *Mm*PylRS (carbons in gray; PDB ID: 2Q7E) is shown. The C=C bond of AzGVAisoK (black) is stabilized by W349. Residues S313 and Q311 form hydrogen bonds to the ligand backbone. These interactions are absent in the wt enzyme. The A271 mutation enlarges the pocket to accommodate the N_3_ group, whereas Y271 in the wt enzyme would cause a steric clash. (d) AzGVAisoK was successfully incorporated into various POIs: Staudinger reduction and protein purification afforded Ub­(K11GVAisoK)-H6 (M calc: 9.527 Da, obs: 9.527 Da), SUMO2­(K11GVAisoK)-H6 (M calc: 11.440 Da, obs: 11.440 Da) and PCNA­(K164GVAisoK)-H6 (M calc: 29.731 Da, obs: 29.732 Da) as confirmed by SDS-PAGE and LC-MS.

Site-specific incorporation of GVAisoK into a POI, followed by sortylation with Srt2A-compatible SUMO variants, provides defined SUMO-POI conjugates bearing a Michael acceptor positioned in the linker between SUMO and the target protein. Because N-terminal glycine residues in ncAAs are unstable in cellular environments,^43^ we synthesized the azide-protected derivative AzGVAisoK, in which the α-amine group of glycine is masked to prevent cleavage by endogenous peptidases. As previously shown for AzGGisoK,^31^ site-specific incorporation followed by on-protein Staudinger reduction unmasks the glycine moiety, allowing it to engage in sortylation. In addition to improving intracellular stability, azide protection is expected to facilitate the identification of an orthogonal aaRS variant, building on previously established PylRS variants evolved for AzGGisoK incorporation.

AzGVAisoK was synthesized on solid support from immobilized N^α^-Boc-protected lysine by sequential coupling of Fmoc-protected (*E*)-4-aminobut-2-enoic acid and azidoacetic acid, followed by cleavage and global deprotection, affording AzGVAisoK on multigram scale within 1 day (Supporting Information Section 2.9.4).

Given the structural similarity between AzGVAisoK and AzGGisoK, we first tested the established *Methanosarcina barkeri* (*Mb*) AzGGisoK-RS (*Mb*PylRS L274A, N311Q, C313S),^31^ along with its *Methanosarcina mazei* (*Mm*) and *Methanomethylophilus alvus* (*Ma*) counterparts and a series of rationally designed variants for AzGVAisoK incorporation. However, none of the tested PylRS variants supported incorporation at useful levels (Figure S13). Likewise, screening of more than 150 additional PylRS variants available in the laboratory failed to identify a suitable candidate (Figure S14).

We therefore subjected the *Mb*PylRS library originally generated for the selection of AzGGisoK-RS^31^ to two rounds of positive selection using AzGVAisoK, resulting in the identification of a novel and efficient AzGVAisoK-RS variant (*Mb*PylRS Y271A, N311Q, C313S, Y349W) (Figure S15). Co-transformation of the *Mb*AzGVAisoK-RS/tRNA pair with an sfGFP­(N150TAG)-H6 reporter in *E. coli* K12 yielded AzGVAisoK-dependent expression of full-length protein, indicating successful amber suppression (Figures 3b and S16). Intact protein LC-MS analysis of sfGFP­(N150GVAisoK)-H6, purified in the presence of Tris­(2-carboxyethyl)­phosphine (TCEP), confirmed efficient incorporation and on-protein reduction of AzGVAisoK, without evidence for reaction with cellular nucleophiles or phospha-Michael addition of TCEP to the vinyl amide warhead (Figure 3b).

The failure of established AzGGisoK-RS variants to efficiently incorporate AzGVAisoK suggested that the vinyl amide-containing ncAA requires a distinct active-site environment. Because the selected AzGVAisoK-RS differs from AzGGisoK-RS by only a small set of active-site mutations, we sought to understand how these substitutions accommodate the larger bifunctional ncAA. Such structural insight could rationalize the newly acquired substrate specificity and guide future PylRS engineering for related electrophilic or otherwise functionalized lysine derivatives. To this end we purified the C-terminal domains of the *Mb* and *Mm*AzGVAisoK-RS variants and cocrystallized them in the presence of AzGVAisoK and ATP. Atomic-resolution crystals up to 1.25 Å were obtained for both enzymes with clear electron density of ATP in the active site (Figure S17). In contrast, well-defined density for AzGVAisoK was only obtained for the *Mm* variant, providing detailed insight into substrate recognition. AzGVAisoK is deeply buried within a hydrophobic pocket and forms defined interactions with the engineered residues. The vinyl group is stabilized by pi-stacking with W349 (*Mb* numbering). Q311 hydrogen bonds to the ε-amine of the lysine moiety, while S313 interacts with the backbone carboxyl group of the glycine residue of AzGVAisoK. Notably, the Y271A mutation enlarges the binding pocket to accommodate the azide moiety (Figures 3c and S17).

With an efficient AzGVAisoK-RS in hand, we expressed and purified Ub­(K11GVAisoK)-H6, SUMO2­(K11GVAisoK)-H6 and PCNA­(K164GVAisoK)-H6. Ub served as a well-behaved model acceptor protein, whereas SUMO2 and PCNA represent established and biologically relevant SUMOylation targets. Incorporation and on-protein reduction to GVAisoK were confirmed by SDS-PAGE and LC-MS analysis (Figures 3d and S18), yielding preparatively useful amounts of GVAisoK-bearing proteins for subsequent sortylation.

### Srt2A-Mediated SUMOylation of GVAisoK-Containing Target Proteins

We next tested whether GVAisoK-bearing proteins could serve as acceptors for Srt2A-mediated SUMOylation to generate SUMO-POI ABPs (Figure 4a).

**Figure 4:**
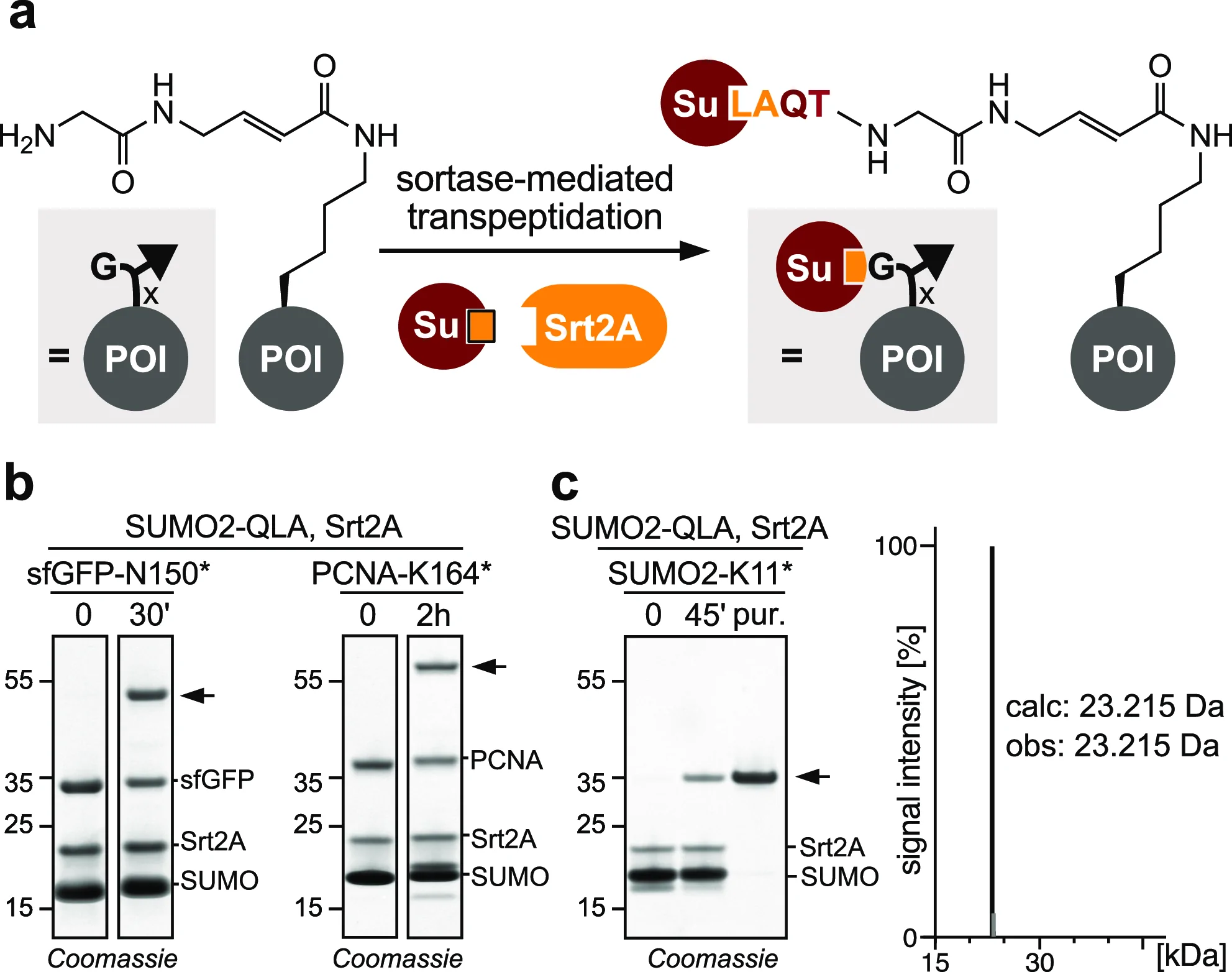
Sortylation of SUMO to GVAisoK-bearing POIs grants access to SUMO-POI conjugate ABPs. (a) Schematic display for generating SUMO-POI conjugate ABPs that display the vinyl amide in proximity to the isopeptidic linkage by reacting Srt2A-competent SUMO donors with GVAisoK-bearing POIs. (b) Incubation of 20 μM sfGFP­(N150GVAisoK)-H6 or PCNA­(K164GVAisoK)-H6 with 100 μM SUMO2­(QLA)-H6 and 20 μM Srt2A at 37 °C shows successful sortylation with maximum conversion reached after 30 min and 2 h, respectively, as shown by SDS-PAGE. Full gels and time courses are shown in Figure S19. (c) SDS-PAGE analysis of reaction start- and end-point of sortylation as well as 1 μg of isolated HA-SUMO­(QLA)-SUMO2­(K11GVAisoK, ΔG)-H6 (M calc: 23.215 Da, obs: 23.215 Da) obtained from incubation of 100 μM SUMO2 donor, 20 μM GVAisoK-bearing acceptor and 20 μM Srt2A at 37 °C for 45 min followed by isolation via NiNTA affinity chromatography and size exclusion chromatography.

Initial test-scale reactions with Srt2A-compatible SUMO donors yielded efficient sortylation of sfGFP­(N150GVAisoK)-H6, PCNA­(K164GVAisoK)-H6 and SUMO2­(K11GVAisoK)-H6. Maximal conversion was reached within 30 min to 2 h, depending on the target protein (Figures 4b and S19). In contrast, acceptor proteins containing N^ε^-Boc-protected lysine (BocK), remained unmodified, confirming that SUMO ligation requires the reduced glycine handle of GVAisoK and proceeds site-specifically (Figure S19). As the sortase reaction is reversible, prolonged incubation leads to accumulation of hydrolyzed, sortase-incompetent SUMO donor and reduced product formation. For preparative-scale sortylation reactions, we therefore quenched the reaction at maximal conversion using phenyl vinyl sulfone to covalently inhibit Srt2A. SUMO-POI conjugates were purified by affinity chromatography followed by size exclusion chromatography. For the SUMO2­(K11GVAisoK) acceptor, the C-terminal glycine preceding the H6-tag was removed to prevent processing by deSUMOylating enzymes. Using these optimized conditions, we generated HA-SUMO1/2­(QLA)-PCNA­(K164GVAisoK)-H6 (Figure S20) and HA-SUMO1/2­(QLA)-SUMO2­(K11GVAisoK, Δ*G*)-H6 and confirmed product identity and integrity by full-length protein LC-MS analysis (Figures 4c and S21).

### SUMO-POI ABPs Engage deSUMOylases in a Paralog- and Substrate-Specific Manner

Having established access to SUMO-POI ABPs, we next evaluated their ability to trap different deSUMOylating enzymes (Figure 5a). Incubation of the HA-tagged K11-linked diSUMO probe, bearing the vinyl amide electrophile between the two SUMO units with SENP2­(CD) at 37 °C resulted in covalent trapping of the enzyme. In contrast, no adduct formation was observed with the catalytically inactive mutant SENP2­(CD, C548A) (Figure 5b). The absence of detectable labeling of SENP2­(CD, C548A), which retains noncatalytic cysteines, indicates that trapping is directed by productive active-site engagement of the diSUMO probe rather than nonspecific modification of cysteine residues. Formation of the diSUMO2-SENP2­(CD) complex was further confirmed by intact protein LC-MS analysis (Figure 5c). In addition, GVAisoK-linked probes enabled SENP trapping in complex environments, as demonstrated for HA-SUMO2­(QLA)-PCNA­(GVAisoK)-H6 and FLAG-SENP1­(CD) in HEK293T lysates (Figure S23).

**Figure 5:**
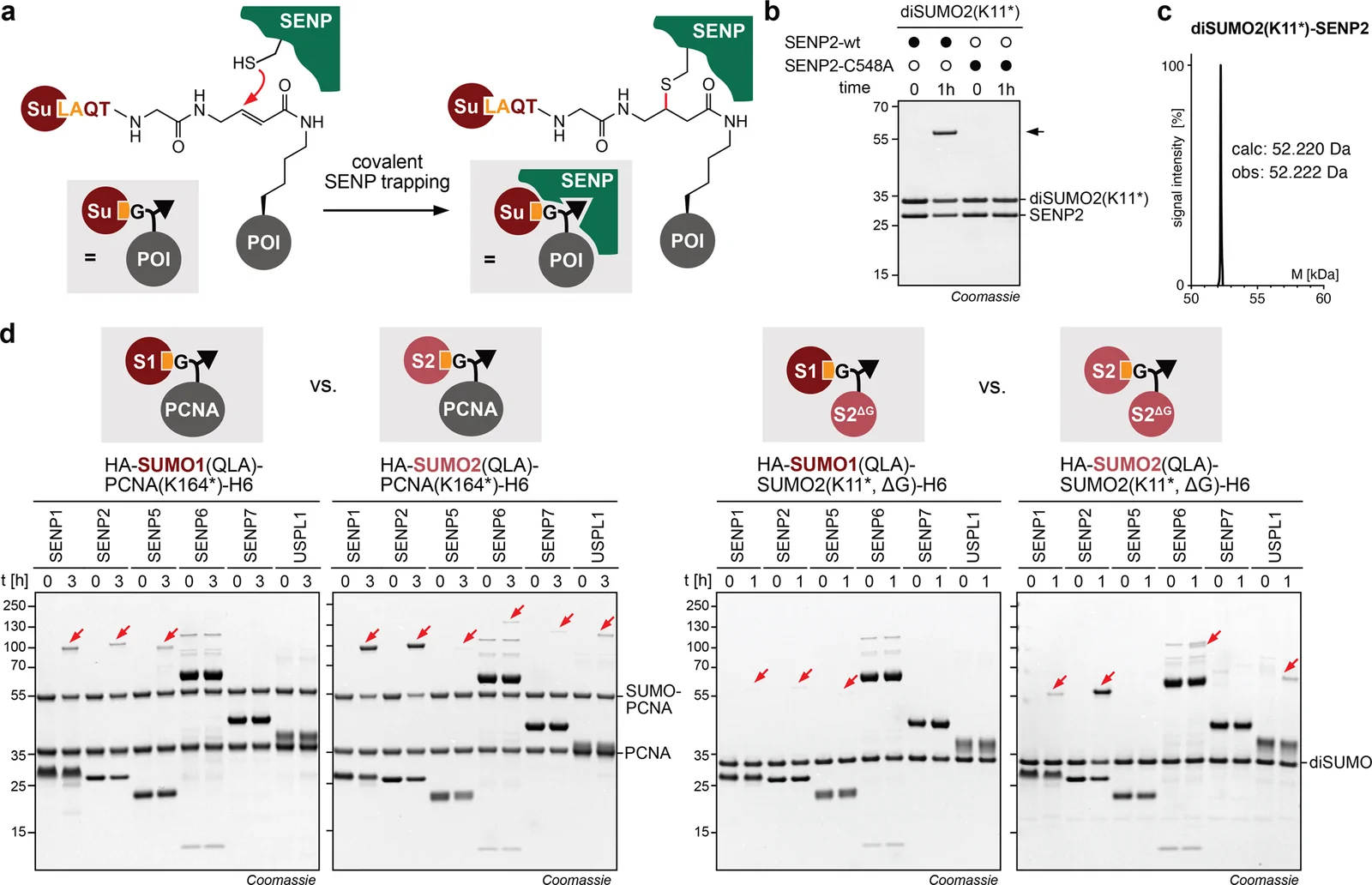
Profiling of SENP specificities with GVAisoK-linked SUMO-POI conjugate ABPs. (a) GVAisoK-linked SUMO-POI conjugate ABPs promote potential substrate-specific interactions with SENPs that can be covalently trapped by the vinyl amide warhead near the SUMO conjugation site. (b) Incubation of 15 μM diSUMO2­(K11GVAisoK) with 15 μM SENP2­(CD) in 20 mM Tris pH 7.5, 100 mM NaCl, 0.5 mM TCEP at 37 °C for 1 h leads to specific SENP trapping while no complex formation is observed for the catalytically inactive SENP2­(CD, C548A), as analyzed by SDS PAGE. (c) LC-MS analysis of the covalently linked diSUMO2­(K11GVAisoK)-SENP2 complex (M calc: 52.220 Da, obs: 52.222 Da). (d) SDS-PAGE analysis of in vitro SENP trapping assays: 15 μM SENP were incubated with 15 μM SUMO1-PCNA, SUMO2-PCNA, SUMO1-SUMO2, diSUMO2 in 20 mM Tris pH 7.5, 100 mM NaCl, 0.5 mM TCEP at 37 °C. Bands corresponding to covalently linked complexes are indicated by red arrows.

To assess whether GVAisoK-linked SUMO-POI conjugates could report on deSUMOylase specificity across SUMO paralog and substrate contexts, we first performed two sets of control experiments with a panel of purified SENP­(CDs). SUMO donors carrying C-terminal GVAisoK in the absence of an acceptor POI (“no acceptor POI” control) showed trapping preferences similar to the monoSUMO-PA probes (Figure S7), albeit with lower overall efficiencies (Figure S22a). In contrast, non-SUMOylated GVAisoK-bearing acceptor POIs (“no SUMO donor” control) did not form detectable complexes, confirming that SUMO recognition is required for productive SENP trapping (Figure S22b).

We then profiled the SUMO-PCNA and diSUMO ABPs against the same panel of deSUMOylases, revealing distinct labeling patterns for GVAisoK-linked probes (red arrows in Figures 5d and S24). For PCNA-based conjugates, SUMO1-PCNA selectively trapped SENP1, SENP2, and SENP5. Replacing the SUMO1 donor with SUMO2 markedly altered the trapping profile: SUMO2-PCNA displayed enhanced trapping of SENP1 and SENP2, lost detectable engagement of SENP5 and additionally labeled SENP6, SENP7 and USPL1. Thus, in the context of PCNA, the SUMO donor paralog strongly influences deSUMOylase engagement. Distinct deSUMOylase-trapping patterns were also observed for the diSUMO ABPs. SUMO1-SUMO2 exhibited only very weak trapping of SENP1, SENP2, and SENP5, whereas diSUMO2 engaged in more efficient complex formation with SENP1, SENP6, USPL1 and in particular SENP2. These differences highlight the ability of GVAisoK-linked probes to report changes in deSUMOylase engagement depending on SUMO-chain architecture.

Together, these results demonstrate that the sortase-generated SUMO-POI ABPs reveal pronounced effects of substrate identity, SUMO paralog and chain architecture on deSUMOylase recognition.

## Discussion and Conclusion

Here, we established a genetically programmable chemoenzymatic platform for the generation of SUMO-based ABPs. By combining Srt2A-comptatible SUMO variants with glycine-functionalized electrophiles, we first generated monoSUMO ABPs bearing propargylamide, vinyl ester or vinyl amide warheads. These probes covalently trap deSUMOylases in an active-site-dependent manner, are applicable in cellular lysates and can be assembled and applied in one pot. The efficient ligation of GPA further enables formation of SUMO propargylamide probes in living cells, demonstrating that sortase-mediated probe assembly and SENP trapping can be performed directly in cellular environments.

To extend this strategy beyond monoSUMO probes, we developed AzGVAisoK, a bifunctional ncAA that combines an azide-protected glycine handle for sortase-mediated SUMOylation with a vinyl amide electrophile for deSUMOylase trapping. Identification of an engineered PylRS/tRNA pair enabled site-specific incorporation of AzGVAisoK into protein substrates, followed by on-protein reduction to the sortase-reactive GVAisoK handle. Structural analysis of AzGVAisoK-RS rationalizes substrate recognition of this larger bifunctional ncAA and will support future PylRS engineering of related lysine derivatives. Srt2A-mediated ligation of GVAisoK-bearing substrate proteins with SUMO variants affords defined SUMO-POI ABPs in which the electrophile is positioned close to the native isopeptide linkage. Because this strategy proceeds under mild aqueous conditions and avoids total chemical protein synthesis, refolding or thiol-elimination chemistry, it is compatible with protein substrates that are difficult to access by established approaches,^29^ including folded, multimeric or cysteine-containing POIs.

Application of the resulting GVAisoK-linked SUMO-POI probes to a panel of deSUMOylases revealed distinct trapping patterns that depend on SUMO paralog and acceptor substrate. Importantly, these profiles were not simply recapitulations of monoSUMO probe reactivity, indicating that substrate context and linkage architecture contribute to deSUMOylase recognition. For PCNA-based probes, replacing SUMO1 with SUMO2 markedly altered the SENP trapping profile. This difference is consistent with the known biology of PCNA K164 SUMOylation. Under basal conditions, PCNA is predominantly modified by SUMO1, whereas SUMO2/3 conjugation becomes more prominent under certain stress-conditions such as transcription-replication conflicts.^44,45^ The broader deSUMOylase trapping observed with the SUMO2-PCNA probe is therefore consistent with the possibility that SUMO2/3-selective deSUMOylases contribute to processing stress-induced or chain-competent PCNA modifications. Similarly, the stronger engagement of SENP2, SENP6 and USPL1 by the K11-linked diSUMO2 probe aligns with the prevalence of SUMO2/3-based polySUMO chains and with reported preference of these enzymes.^38–40^ These findings highlight the central advantage of substrate-mimetic ABPs: by presenting the deSUMOylase with both the SUMO modifier and its local substrate/linkage environment, they reveal recognition features that are not accessible with conventional monoSUMO probes.

Looking forward, this chemoenzymatic platform should enable tailored SUMO-substrate probes for dissecting deSUMOylase specificity across additional cellular targets, conjugation sites and SUMO-chain architectures. In particular, probes based on DNA repair or chromatin-associated SUMO substrates could help define how substrate identity and cellular state influence deSUMOylase recognition.^46–49^ By varying the SUMO paralog, modification site, and chain length, this approach may provide native-like probes to define how deSUMOylases recognize and edit distinct SUMO-chain architectures, including polymeric SUMO2/3 chains and mixed or SUMO1-capped conjugates that are difficult to interrogate with conventional monoSUMO ABPs.^32^ Beyond biochemical profiling, covalent stabilization of deSUMOylase:SUMO-substrate complexes may facilitate structural studies of transient enzyme–substrate interactions and support enrichment-based proteomics to identify substrate-selective deSUMOylases in native cellular environments.

More broadly, the strategy presented here is not limited to SUMO-based probes, but can be readily adapted to other Ubl systems, thereby expanding the Ub/Ubl chemical biology toolbox for modifier-specific enzyme profiling. The use of alternative transpeptidases, either already available^50^ or accessible through directed evolution,^30,51–53^ may further expand the scope of chemoenzymatic Ub/Ubl and Ub/Ubl-POI ABP generation. Such enzymes could improve ligation efficiency, enable orthogonal assembly strategies, and minimize the impact of recognition-motif mutations on trapping characteristics. Together, these developments are expected to provide increasingly native-like conjugate ABPs for interrogating modifier-, linkage-, and substrate-specific enzyme recognition across the Ub/Ubl signaling network.

## Supplementary Material



## Notes

Supplementary figures and supporting additional experiments, full gels and Western blots, experimental procedures, plasmid list and protein amino acid sequences, NMR spectra (PDF)

## ∥

AITHYRA—Research Institute for Biomedical Artificial Intelligence, A-1030 Vienna, Austria

## Abbreviations

Ub: ubiquitin
Ubl: ubiquitin-like modifier
SUMO: small ubiquitin-like modifier
POI: protein of interest
ABP: activity-based probe
SPPS: solid-phase peptide synthesis
PA: propargylamide
VME: vinyl methyl ester
VDMA: vinyl dimethyl amide
TCEP: Tris­(2-carboxyethyl)­phosphine
ncAA: noncanonical amino acid
*Mm*: *Methanosarcina mazei*
*Mb*: *Methanosarcina barkeri*
*Ma*: *Methanomethylophilus alvus*
GCE: genetic code expansion
GSH: glutathione
Pyl: pyrrolysine
PylRS: pyrrolysyl-tRNA synthetase
SENP: sentrin-specific protease
PCNA: proliferating cell nuclear antigen
wt: wild type.
