# Supplementary material for: A Genetically Encoded Electrophilic Lysine Derivative Enables Sortase-Mediated Assembly of SUMO Activity-Based Probes
Source: J Am Chem Soc. 2026 Aug 12;148(35):38092–101. doi: 10.1021/jacs.6c10081 (PMC13564413; doi:10.1021/jacs.6c10081)
Supplement: Supplementary file 1 [file ja6c10081_si_001.pdf]

Supplementary Information to:

A Genetically Encoded Electrophilic Lysine Derivative Enables Sortase-Mediated Assembly of SUMO Activity-Based Probes

Vera Wanka,[1] Denys Kvasha,[1] Marko Cigler,[2,4] Kristina Heymes-Krauskopf,[1] Philipp Ruckgaber,[1] Anna Heider,[3] Maximilian Fottner,[1] Michael Groll,[3] Kathrin Lang[*1]

Affiliations

[1]Laboratory for Organic Chemistry (LOC), Department of Chemistry and Applied Biosciences (D-CHAB), ETH Zurich, 8093 Zurich, Switzerland

[2]Department of Chemistry, Technical University of Munich, 85748 Garching, Germany

[3]TUM School of Natural Sciences, Department of Bioscience, Center for Protein Assemblies, Technical University of Munich, Garching 85748, Germany

[4]Present address: AITHYRA – Research Institute for Biomedical Artificial Intelligence, A-1030 Vienna, Austria

Email corresponding author: kathrin.lang@org.chem.ethz.ch

1. Supplementary Figures S1-S24 3

2. Experimental Procedures 28

2.1 General Remarks 28

2.2 Protein Expression and Purification 29
2.2.1 Expression and purification of SUMO donors for sortase-mediated ligation 29
2.2.2 Expression of HA-SUMO(ΔG)-intein constructs and purification of HA-SUMO(ΔG)-MESNa thioesters 30
2.2.3 Expression and purification of SENP1(CD), SENP2(CD), SENP5(CD) and SENP2(CD, C548A) ... 31
2.2.4 Expression and purification of SENP6(CD) and SENP7(CD) 31
2.2.5 Expression and purification of USPL1(CD) 32
2.2.6 Expression and purification of Desi1 and SENP8 33
2.2.7 Expression and purification of Srt2A 34
2.2.8 Expression and purification of C-terminal domain (CTD) of *Mb* and *Mm*AzGVAisoK-RS for X-ray crystallography 35
2.2.9 Expression and purification of sfGFP(N150*)-H6, Ub(K11*)-H6, SUMO2(K11*)-H6 and PCNA(K164*) amber-suppressed with AzGVAisoK or BocK 37
2.2.10 GFP test expressions for determination of amber suppression efficiency promoted by PylRS variants 39

2.3 SUMO1/2(XLA)-H6 (X = L or Q) processing assay with SENP1(CD) and SENP2(CD) 40

2.4 In vitro Srt2A-mediated ligation 40
2.4.1 Test-scale Srt2A-mediated ligation 40
2.4.2 Prep-scale Srt2A-mediated ligation 41

2.5 Aminolysis of HA-SUMO(ΔG)-MESNa thioesters with propargylamine 46

2.6 SENP trapping assays 46
2.6.1 In vitro SENP trapping with catalytic domains of SENPs 46
2.6.2 SENP trapping in cell lysate with assembled ABPs 47
2.6.3 Concerted sortase reaction and SENP trapping 49

2.7 In cellulo SENP trapping 50
2.7.1 In cellulo SENP trapping assay 50
2.7.2 Cell viability assay 51

2.8 Synthetase screening and selection campaign 51
2.8.1 Synthetase screen for AzGVAisoK incorporation by previously published PylRS variants 51
2.8.2 Selection of *Mb*AzGVAisoK-RS from *Mb*PylRS library 52

2. 9 Chemical synthesis 54
2.9.1 Synthesis of GPA 54
2.9.2 Synthesis of GVME 55
2.9.3 Synthesis of GVDMA 57
2.9.4 Synthesis of ncAAs 60

3. Plasmids and Proteins 64

3.1 Plasmids 64

3.2 Amino acid sequences 67

4. NMR Spectra and HRMS 76

Supplementary Information References 90

1. Supplementary Figures S1-S24

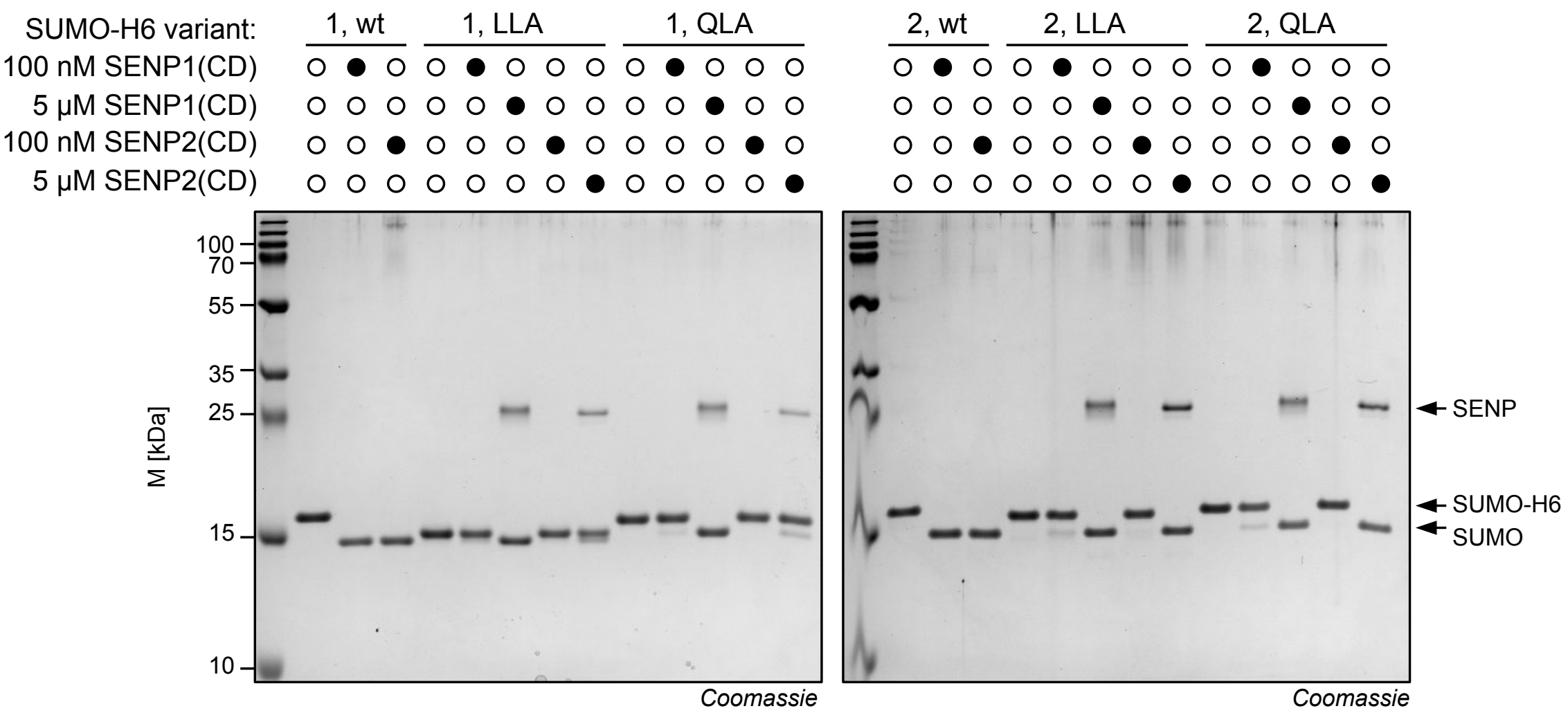

Supplementary Figure S1: Processing of Srt2A-competent SUMO-H6 variants by SENP1 and SENP2. Incubation of 15 µM wtSUMO1-H6, SUMO1(LLA)-H6, SUMO1(QLA)-H6, wtSUMO2-H6, SUMO2(LLA)-H6, or SUMO2(QLA)-H6 with 100 nM or 5 µM SENP1(CD) or SENP2(CD) in 20 mM Tris pH 7.5, 100 mM NaCl, 2 mM DTT at 37 °C for 20 h leads to cleavage of the C-terminal H6-tag.

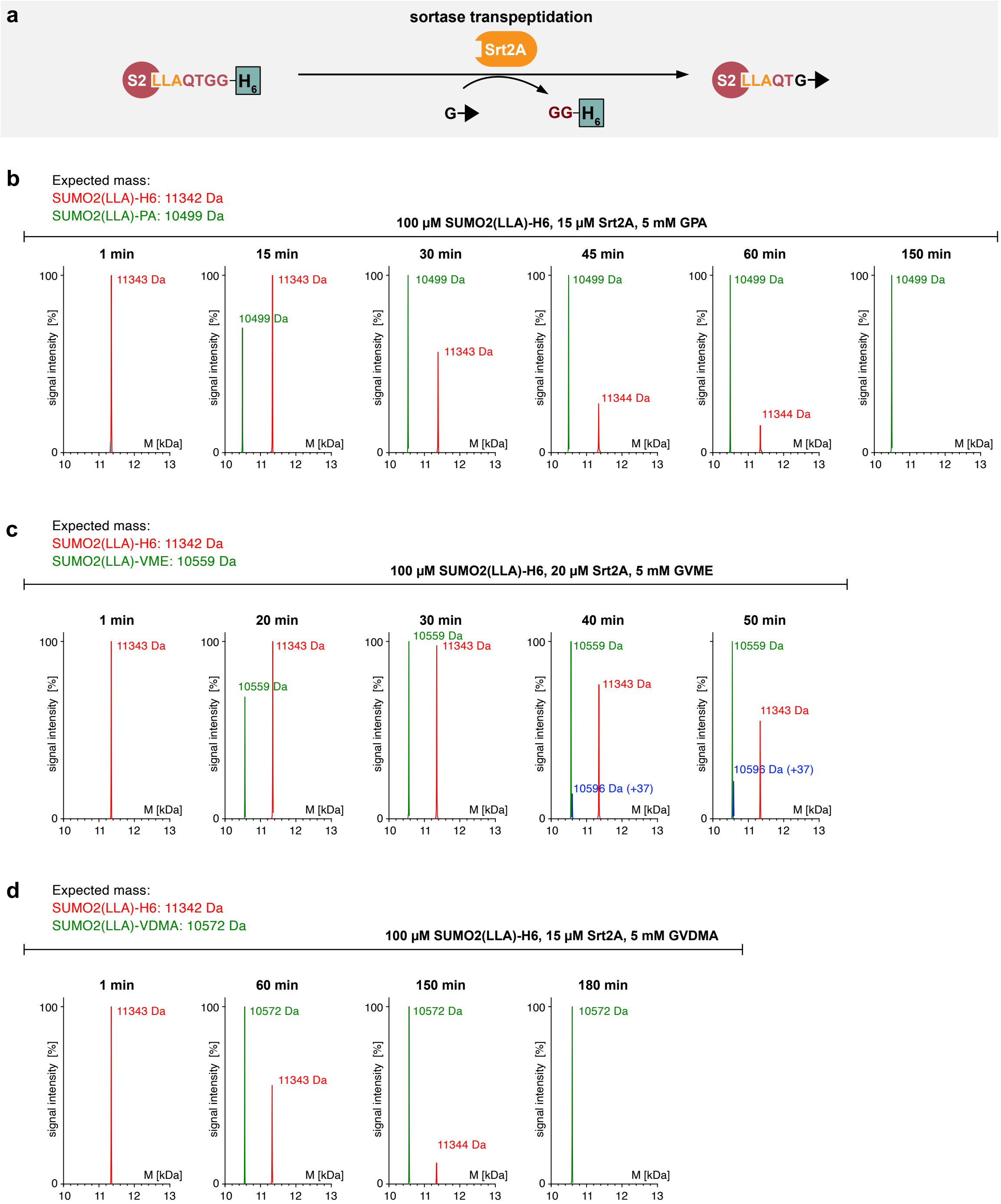

Supplementary Figure S2: *In vitro* generation of SUMO2(LLA) ABPs via Srt2A-mediated ligation with GPA, GVME and GVDMA. a: Srt2A attacks the LAQTG motif presented by SUMO2(LLA)-H6 and catalyzes the transpeptidation reaction between the Thr residue in the motif and a glycine-bearing electrophile (depicted as black triangle) affording SUMO2(LLA) ABPs with C-terminal electrophiles upon release of GG-H6. While the reaction is reversible, high excess of the small molecule warhead shifts the equilibrium towards the product side. **b:** LC-MS time course shows that the incubation of 100 µM SUMO2(LLA)-H6, 15 µM Srt2A, and 5 mM GPA in 50 mM Tris pH 7.5, 150 mM NaCl, 5 mM $CaCl_2$ at 37 °C quantitatively gives SUMO2(LLA)-PA (green peak; M calc: 10499 Da, obs: 10499 Da) in less than 2.5 h. **c:** Incubation of 100 µM SUMO2(LLA)-H6, 20 µM Srt2A, and 5 mM GVME in 50 mM Tris pH 7.5, 150 mM NaCl, 5 mM $CaCl_2$ at 37 °C affords the product SUMO2(LLA)-VME (green peak; M calc: 10559 Da, obs: 10559 Da) with 50% conversion within 30 min. Yet, prolonged incubation of the reaction leads to increasing side product formation (blue peak; M obs: 10596 Da). **d:** Incubation of 100 µM SUMO2(LLA)-H6, 15 µM Srt2A, and 5 mM GVDMA in 50 mM Tris pH 7.5, 150 mM NaCl, 5 mM $CaCl_2$ at 37 °C quantitatively gives SUMO2(LLA)-VDMA (green peak; M calc: 10572 Da, obs: 10572 Da) in less than 3 h.

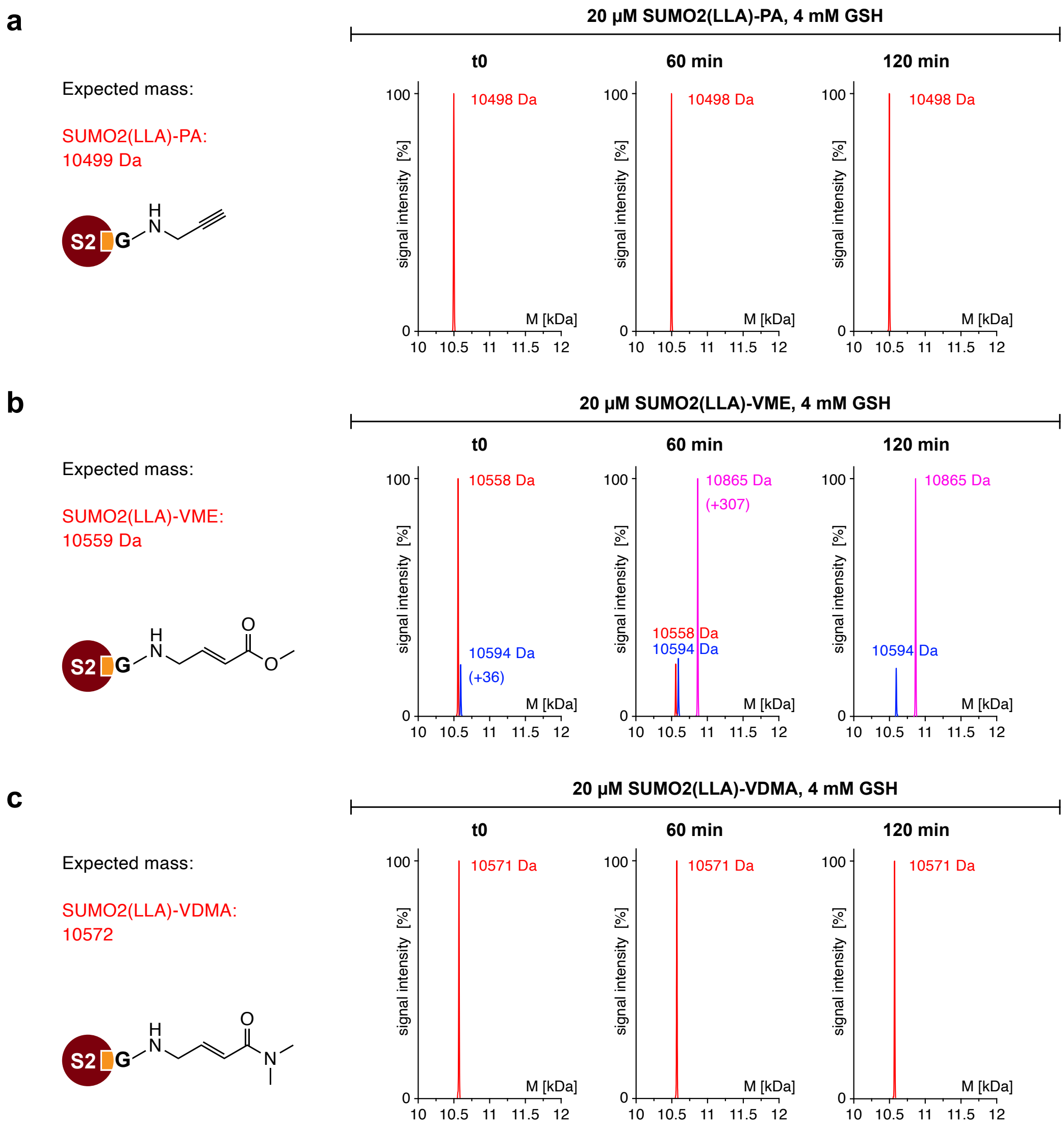

Supplementary Figure S3: SUMO2(LLA)-VME forms adducts with GSH while SUMO2(LLA)-PA and SUMO2(LLA)-VDMA remain stable. LC-MS time course of the incubation of 20 µM SUMO2(LLA) ABPs equipped with PA (in **a**), VME (in **b**), or VDMA (in **c**) with 4 mM GSH (200 eq.) in 20 mM Tris pH 7.5, 100 mM NaCl at 37 °C. Neither SUMO2(LLA)-PA nor SUMO2(LLA)-VDMA react with GSH. In stark contrast, SUMO2(LLA)-VME is fully modified by GSH (pink peak; M calc: 10559 Da + 307 Da = 10866 Da, obs: 10865 Da) within 2 h. The "+36" side product obtained during the Srt2A-mediated generation of SUMO2(LLA)-VME (blue peak, M obs: 10594 Da) remains unmodified, suggesting that both the "+36" side product as well as the GSH modification affect the electrophilic centre of VME.

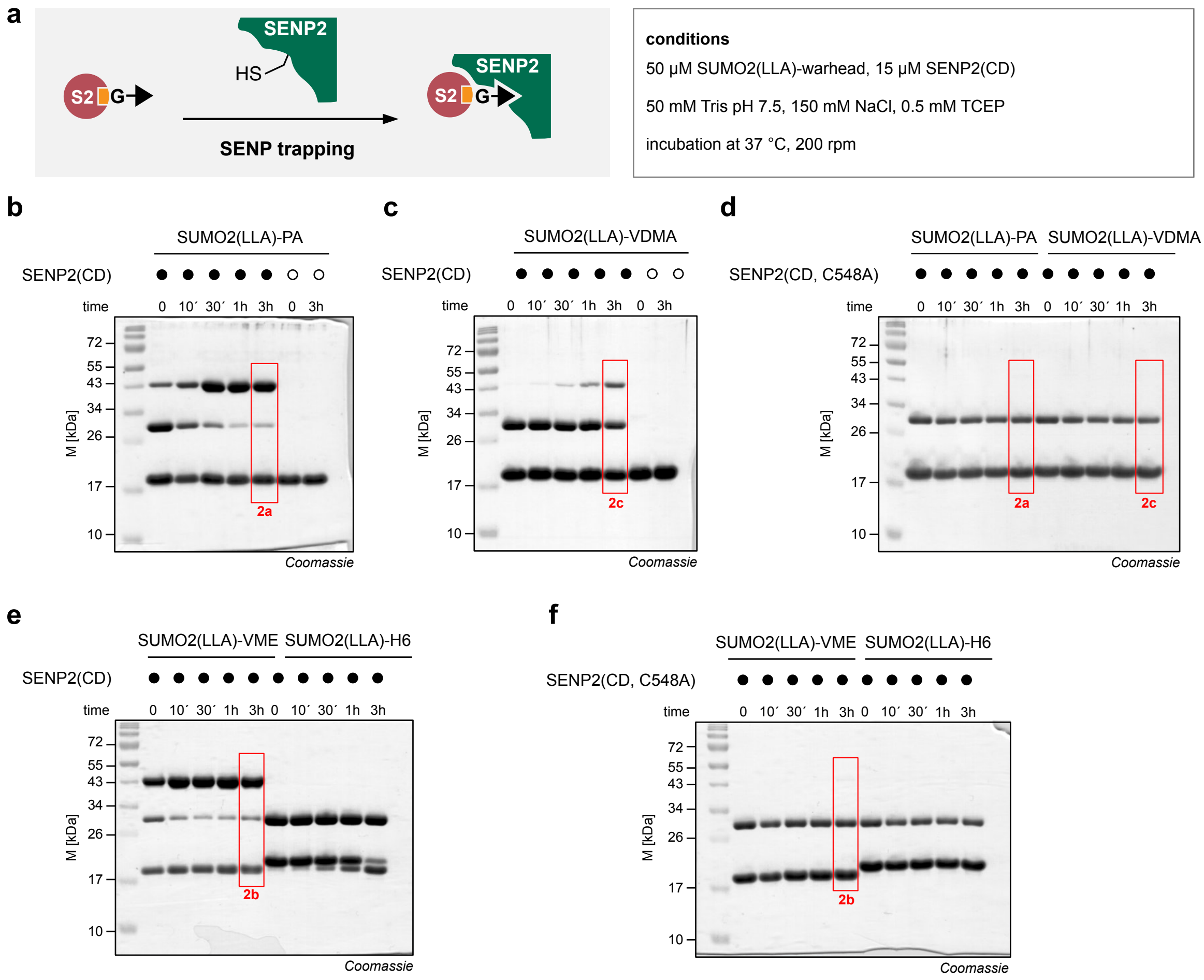

Supplementary Figure S4: SUMO2(LLA)-PA, -VME, and -VDMA covalently trap the active site cysteine of SENP2(CD) in an efficient and specific manner. a: Schematic display and conditions of the SENP2(CD) trapping assay: Incubation of 50 µM ABP and 15 µM SENP2(CD) in 50 mM Tris pH 7.5, 150 mM NaCl, 0.5 mM TCEP at 37 °C, 200 rpm, leads to covalent complex formation in dependence of the SENP active site cysteine. **b-f:** SDS-PAGE analysis of reaction time course of individual trapping assays: SUMO2(LLA)-PA traps SENP2(CD) more effectively than SUMO2(LLA)-VDMA and both ABPs leave the active site mutant SENP2(CD, C548A) unmodified (b-d). SUMO2(LLA)-VME is the most efficient SENP2(CD) probe in the tested panel but also reacts with the catalytically inactive mutant SENP2(CD, C548A), albeit to a very little extent (e, f). Control reactions with SUMO2(LLA)-H6 instead of either ABP show cleavage of the H6-tag by active SENP2(CD) while incubation with the active site mutant SENP2(CD, C548A) has no effect (e, f).
Red boxes indicate sections of the Coomassie-stained SDS gels that are shown in main Figure 2a-c.

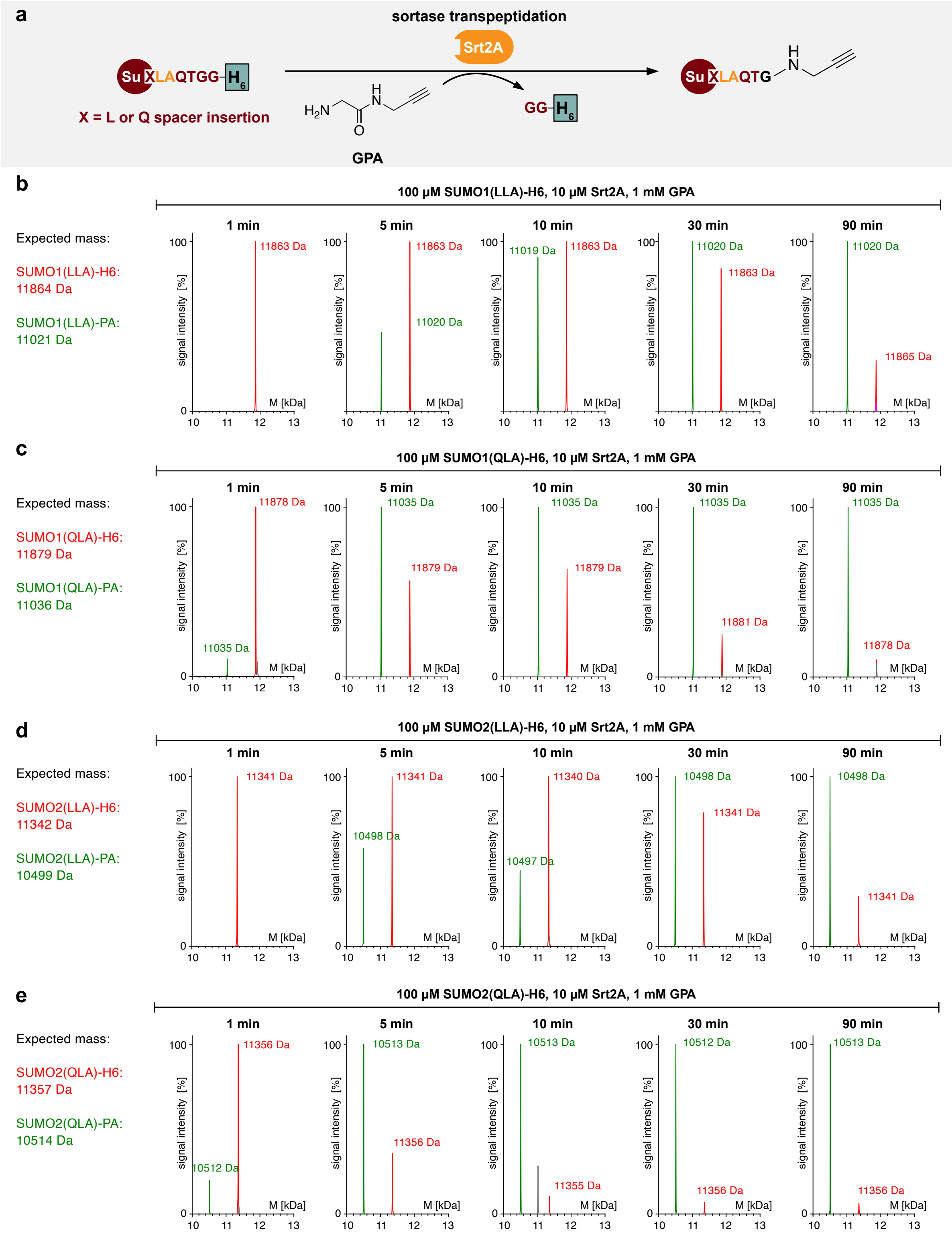

Supplementary Figure S5: Srt2A-mediated ligation of GPA proceeds faster with SUMO1/2(QLA) donors than with SUMO1/2(LLA) donors. a: Schematic display of the Srt2A transpeptidation between SUMO1/2 (LLA) or (QLA) donor proteins and small molecule GPA affording SUMO1/2((Q/L)LA)-PA. LC-MS time course analysis of the incubation of 10 µM Srt2A, 1 mM GPA and 100 µM SUMO1(LLA)-H6 (in **b**), 100 µM SUMO1(QLA)-H6 (in **c**), 100 µM SUMO2(LLA)-H6 (in **d**), or 100 µM SUMO2(QLA)-H6 (in **e**) in 50 mM Tris pH 7.5, 150 mM NaCl, 5 mM $CaCl_2$ at 37 °C reveals faster conversion of (QLA) donors compared to their (LLA) counterparts.

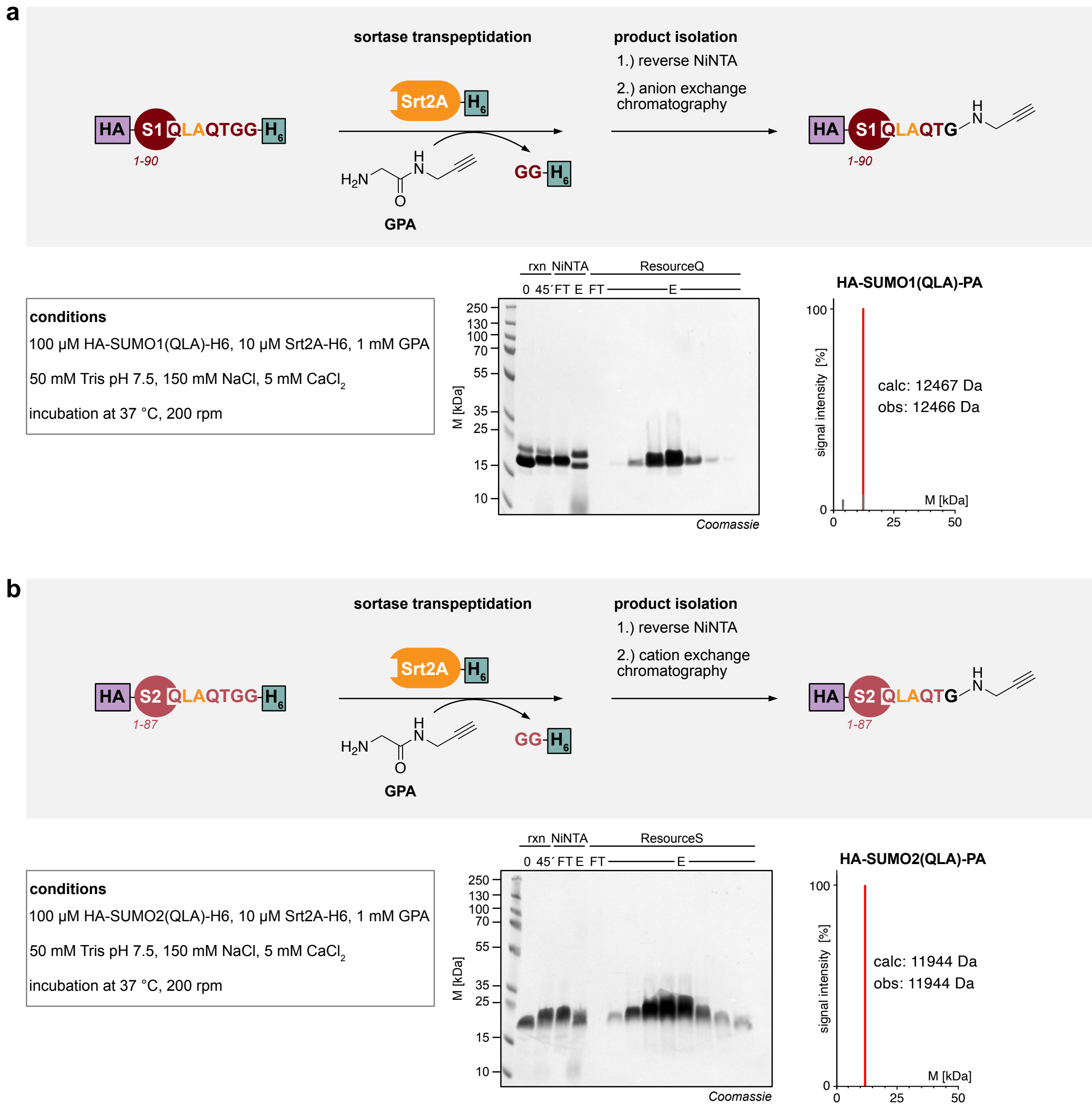

Supplementary Figure S6: Preparative generation of HA-SUMO1/2(QLA)-PA via Srt2A-mediated ligation. **a:** Incubation of recombinantly purified HA-SUMO1(QLA)-H6 (100 µM) with 10 µM Srt2A, 1 mM GPA in 50 mM Tris pH 7.5, 150 mM NaCl, 5 mM $CaCl_2$ at 37 °C, 200 rpm, for 45 min yielded HA-SUMO1(QLA)-PA. Product isolation proceeded via reverse NiNTA spin column followed by anion exchange chromatography of the NiNTA FT fraction over a ResourceQ column. SDS-PAGE analysis of the reaction and purification steps indicated successful conversion and product isolation. Product-containing elution fractions of the anion exchange column were pooled, and rebuffered to 20 mM Tris pH 7.5, 100 mM NaCl. LC-MS analysis of the final product confirmed identity and integrity of HA-SUMO1(QLA)-PA (M calc: 12467 Da, obs: 12466 Da). **b:** Incubation of recombinantly purified HA-SUMO2(QLA)-H6 (100 µM) with 10 µM Srt2A, 1 mM GPA in 50 mM Tris pH 7.5, 150 mM NaCl, 5 mM $CaCl_2$ at 37 °C, 200 rpm, for 45 min yielded HA-SUMO2(QLA)-PA. Product isolation proceeded via reverse NiNTA spin column followed by cation exchange chromatography of the NiNTA FT fraction over a ResourceS column. SDS-PAGE analysis of the reaction and purification steps indicated successful conversion and product isolation. Product-containing elution fractions of the cation exchange column were pooled, and rebuffered to 20 mM Tris pH 7.5, 100 mM NaCl. LC-MS analysis of the final product confirmed identity and integrity of HA-SUMO2(QLA)-PA (M calc: 11944 Da, obs: 11944 Da).
Rxn: reaction, FT: flow-through, E: elution

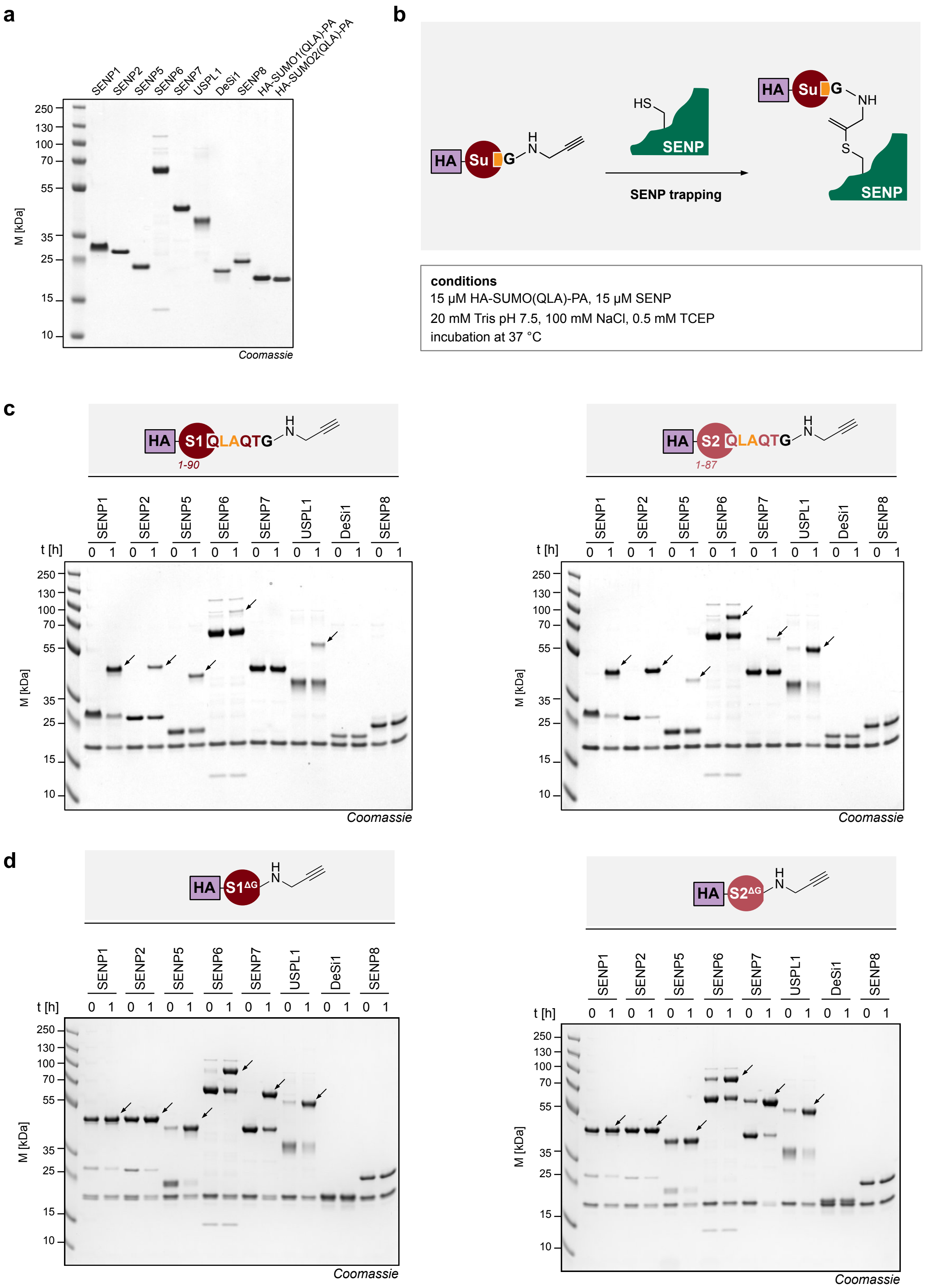

Supplementary Figure S7: In vitro SENP trapping assays with wt and (QLA) SUMO1/2-PA probes. a: SDS-PAGE evaluation of 1 µg of purified catalytic domains of recombinant deSUMOylating enzymes as well as Srt2A-generated SUMO1/2(QLA)-PA probes. All proteins ran at the expected height and, with the exception of SENP6, showed little to no contaminant bands. **b:** Schematic and conditions in vitro SENP trapping assay: 15 µM ABP and 15 µM SENP are incubated in 20 mM Tris pH 7.5, 100 mM NaCl, 0.5 mM TCEP at 37 °C, SDS samples are taken immediately after mixing (t=0) and after one hour. **c:** SDS-PAGE analysis of SENP trapping by HA-SUMO1(QLA)-PA (left) and HA-SUMO2(QLA)-PA (right). Trapped complexes are indicated by black arrows. **d:** SDS-PAGE analysis of SENP trapping by HA-SUMO1-PA (left) and HA-SUMO2-PA (right).

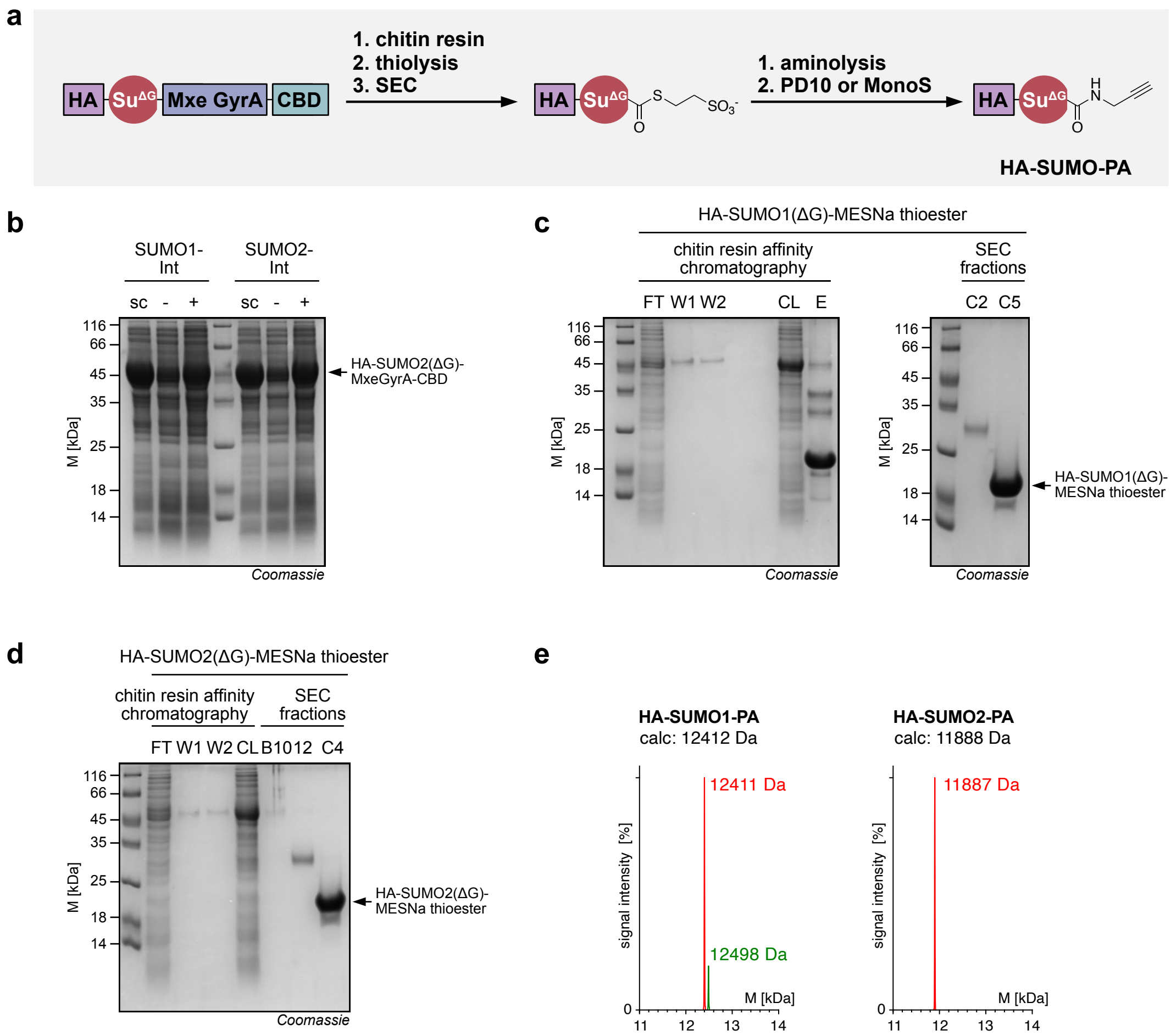

Supplementary Figure S8: Preparative generation of wt HA-SUMO1/2-PA via intein technology. a: HA-tagged SUMO(ΔG) is N-terminally fused to the Mxe GyrA intein followed by a chitin binding domain (CBD) and the recombinantly overexpressed protein is isolated from *E. coli* lysate via chitin resin. On-bead thiolysis of the thioester linkage between HA-SUMO(ΔG) and the acyl-shifted intein by addition of MESNa releases HA-SUMO(ΔG)-MESNa thioester from the column that is further purified by size exclusion chromatography (SEC). Aminolysis of the HA-SUMO(ΔG)-MESNa thioester with high excess of propargylamine yields HA-SUMO-PA that is isolated from the reaction either by a desalting PD10 column or via cation exchange over a MonoS column. **b:** SDS-PAGE analysis of SUMO-intein fusion expression. Intact full-length HA-SUMO1/2(ΔG)-MxeGyrA-CBD was highly abundant after expression in *E. coli* BL21(De3) for 3 h at 30 °C (sample "+"); leaky T7 caused expression even in the starter culture (sample "sc") and pre-induction (sample "-"). **c, d:** SDS-PAGE analysis of purification of HA-SUMO(ΔG)-MESNa thioesters (FT: flow-through, CL: cleared lysate, W1: wash without MESNa, W2: wash with MESNa, E: elution) **e:** LC-MS analysis of purified aminolysis products. HA-SUMO1-PA (M calc: 12412 Da, obs: 12412 Da) contained a fraction of unreacted HA-SUMO1(ΔG)-MESNa thioester (green peak, obs: 12498 Da), HA-SUMO2 -PA (M calc: 11888 Da, obs: 11887 Da) did not contain detectable impurities.

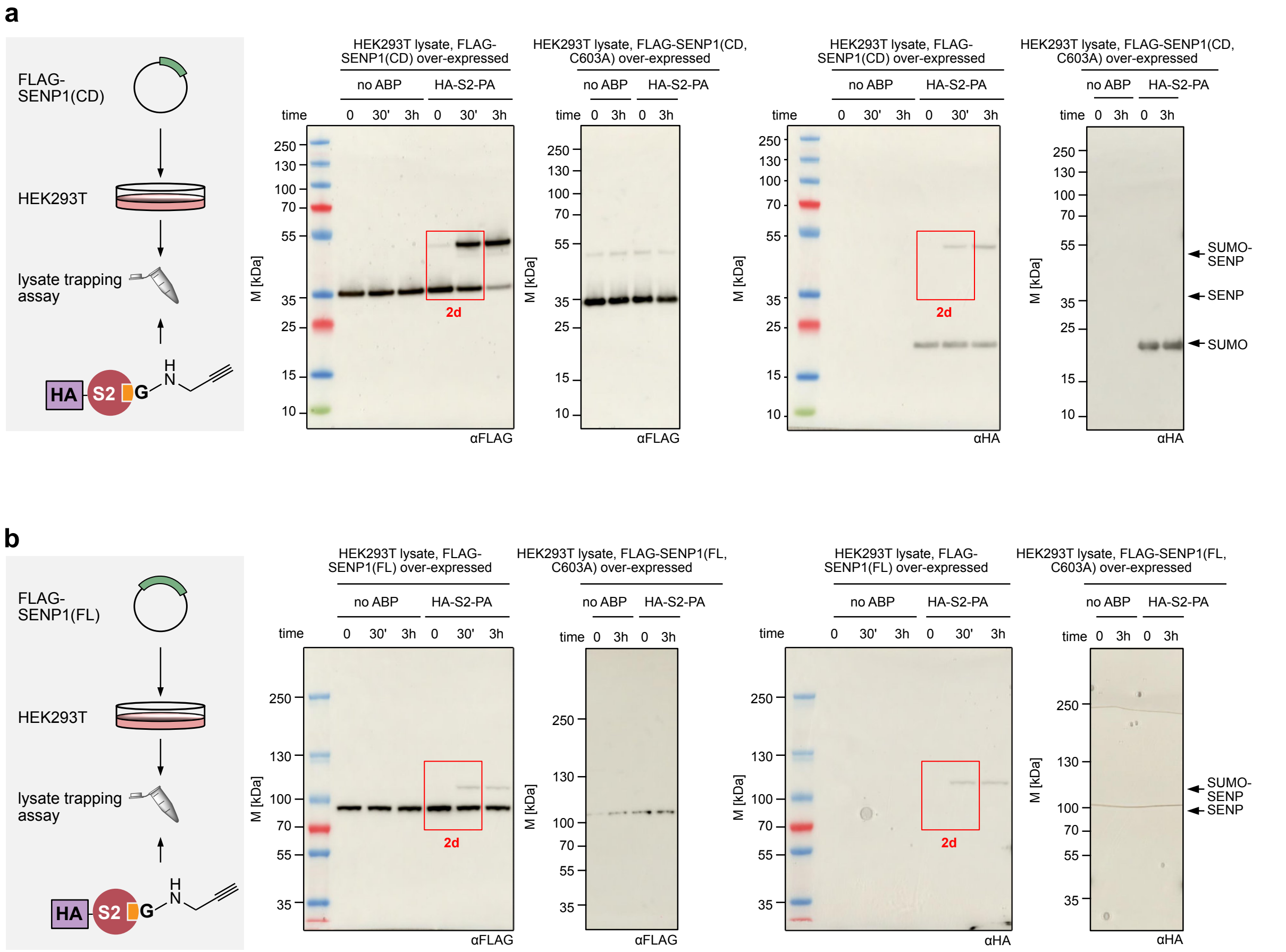

Supplementary Figure S9: Addition of assembled HA-SUMO2(QLA)-PA to HEK293T lysates leads to trapping of overexpressed FLAG-SENP1. a: HEK293T lysates (total protein concentration: 4 mg/mL; buffer: 50 mM Tris pH 7.5, 150 mM NaCl, 0.5% Triton-X-100 (w/v), 1x Roche protease inhibitor) prepared from cells overexpressing FLAG-SENP1(CD) or the catalytically inactive mutant FLAG-SENP1(CD, C603A) were incubated -/+ 5 µM HA-SUMO2(QLA)-PA at 37°C and SDS samples were taken at t = 0, 30 min, and 3 h. Samples were applied to SDS-PAGE (Bolt 4-12% Bis-Tris Plus gels) and active site cysteine-dependent SENP1(CD) trapping was confirmed by Western blot against FLAG and HA. **b:** HEK293T lysates (total protein concentration: 4 mg/mL; buffer: 50 mM Tris pH 7.5, 150 mM NaCl, 0.5% Triton-X-100 (w/v), 1x Roche protease inhibitor) prepared from cells overexpressing FLAG-SENP1(FL) or the catalytically inactive mutant FLAG-SENP1(FL, C603A) were incubated -/+ 5 µM HA-SUMO2(QLA)-PA at 37 °C and SDS samples were taken at t = 0, 30 min, and 3 h. Samples were applied to SDS-PAGE (NuPAGE 3-8% Tris-Acetate gels) and active site cysteine-dependent SENP1(FL) trapping was confirmed by Western blot against FLAG and HA.
Red boxes indicate sections of the Western blots that are shown in main Figure 2d.

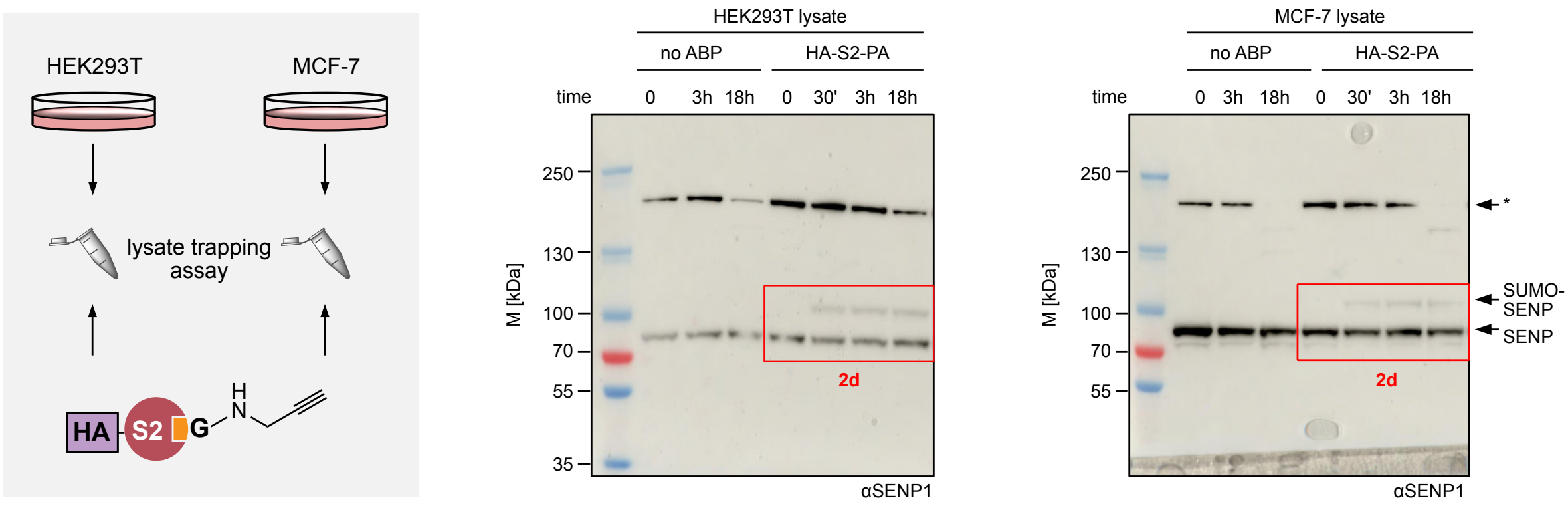

Supplementary Figure S10: Addition of assembled HA-SUMO2(QLA)-PA to HEK293T or MCF-7 lysates leads to trapping of endogenous SENP1. Lysate of untransfected HEK293T or MCF-7 cells (total protein concentration: 5 mg/mL for HEK293T or 2 mg/mL for MCF-7; buffer: 50 mM Tris pH 7.5, 150 mM NaCl, 0.5% Triton-X-100 (w/v), 1x Roche protease inhibitor) was incubated -/+ 5 µM of HA-SUMO2(QLA)-PA at 37 °C. SDS samples were taken at t = 0, 3 h, and 18 h and applied to SDS-PAGE (NuPAGE 3-8% Tris-Acetate gels). Trapping of endogenous SENP1 by the ABP was confirmed by Western blot against SENP1.
Red boxes indicate sections of the Western blots that are shown in main Figure 2d, "*" indicates an off-target band of the αSENP1 antibody.

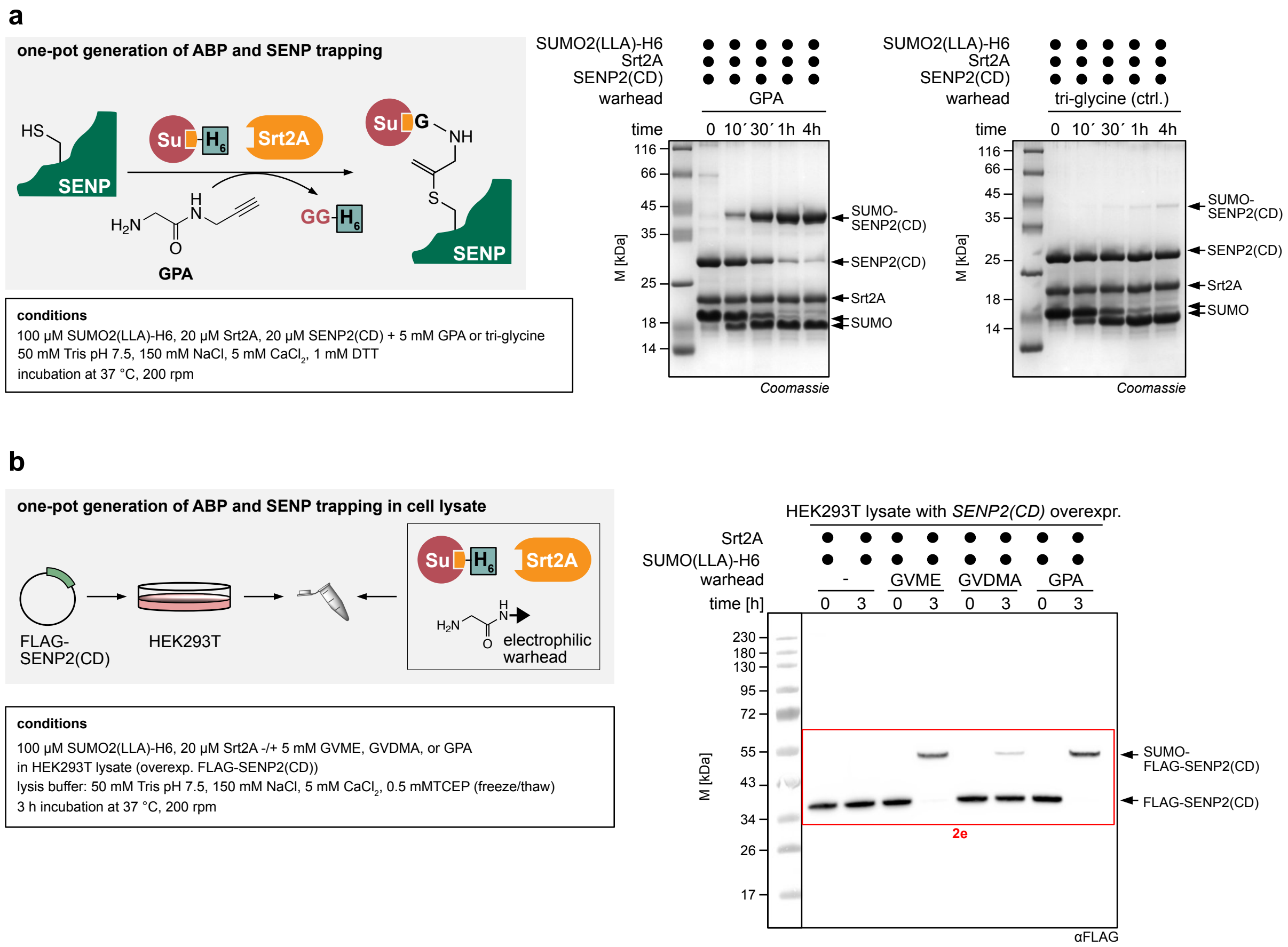

Supplementary Figure S11: One-pot generation and application of SUMO(LLA) ABPs in buffer and cell lysates. a: SDS-PAGE analysis of the incubation of 100 µM SUMO2(LLA), 20 µM Srt2A, 20 µM SENP2(CD) and 5 mM GPA in 50 mM Tris pH 7.5, 150 mM NaCl, 5 mM $CaCl_2$, 1 mM DTT at 37 °C shows fast covalent trapping of SENP2(CD) by the in situ formed SUMO2(LLA)-PA probe. When GPA was replaced by 5 mM tri-glycine, no trapping was observed although a weak band appearing over time indicates mis-sortylation of SUMO2(LLA) to the N-terminus of SENP2(CD) **b:** Lysate prepared from HEK293T cells overexpressing FLAG-SENP2(CD) (buffer: 50 mM Tris pH 7.5, 150 mM NaCl, 5 mM $CaCl_2$, 0.5 mM TCEP, lysis by freeze/thaw) was treated with 100 µM SUMO2(LLA), 20 µM Srt2A and -/+ 5 mM of GVME, GPA, or GVDMA and incubated at 37 °C for 3 h. Western blot against FLAG shows warhead-dependent FLAG-SENP2(CD) trapping, indicating successful one-pot generation and application of SUMO2(LLA) ABPs in complex mixtures like cell extracts.
Red box indicates sections of the Western blot that is shown in main Figure 2e.

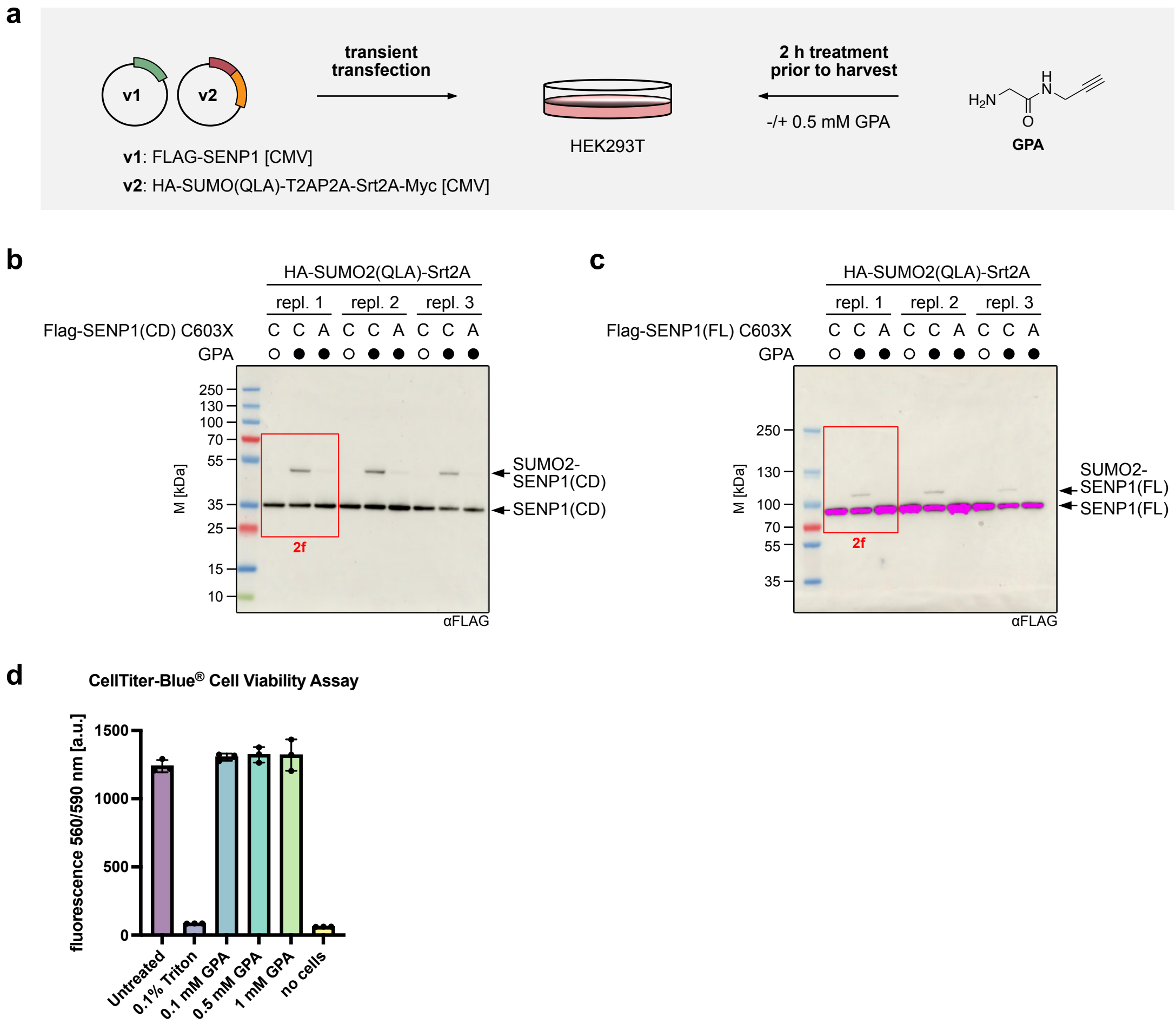

Supplementary Figure S12: In cellulo generation and application of SUMO(QLA) ABPs. a: Schematic workflow for in cellulo SENP trapping: HEK293T cells are co-transfected with vector 1 (v1) for expression of FLAG-SENP under a CMV promoter and vector 2 (v2) for expression of HA-SUMO2(QLA)-T2AP2A-Srt2A-Myc under a CMV promoter; T2AP2A denotes a self-cleaving peptide sequence. Forty hours after transfection, the cells are treated with 0.5 mM GPA and cultured for two more hours. Successful trapping of overexpressed SENP1 is then evaluated by Western blot against FLAG. **b:** Western blot of SDS samples (Bolt 4-12% Bis-Tris Plus gel) shows warhead-dependent trapping of FLAG-SENP1(CD) but not the catalytically inactive mutant FLAG-SENP1(CD, C603A) in three independent biological replicates. **c:** Western blot of SDS samples (NuPAGE 3-8% Tris-Acetate gel) shows warhead dependent trapping of FLAG-SENP1(FL) but not the catalytically inactive mutant FLAG-SENP1(FL, C603A) in three independent biological replicates. Pink color indicates signal saturation stemming from over-exposure of the Western blot. **d:** CellTiter-Blue Cell Viability Assay shows that treatment of HEK293T cells with 0.1 mM GPA, 0.5 mM GPA and 1 mM GPA for 2 h (+3.5 h assays time) does not lead to reduced viability compared to untreated cells. Control wells that were treated with 0.1% Triton-X-100 showed a severe reduction in viability, indicating correct execution of the assay. The experiment was performed in triplicate.

Red boxes indicate sections of the Western blot that are shown in main Figure 2f.

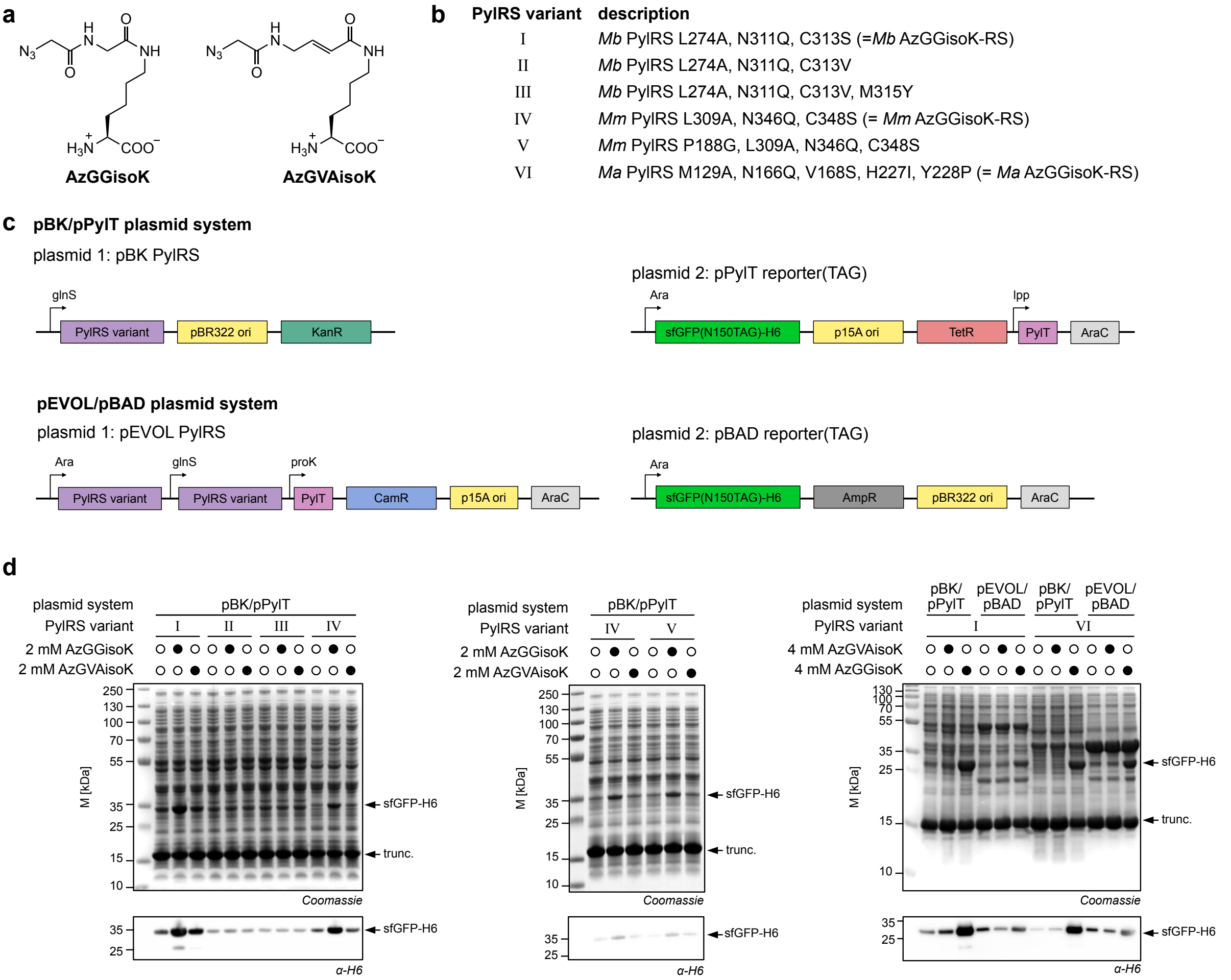

Supplementary Figure S13: Comparison of AzGGisoK and AzGVAisoK incorporation by AzGGisoK-RS and derivatives. a: Structures of AzGGisoK and AzGVAisoK. **b:** Overview of investigated AzGGisoK-RS variants with mutations annotated relative to wt PylRS. **c:** Overview of pBK/pPylT and pEVOL/pBAD plasmid systems used for amber suppression. The pBK plasmid (pBR322 ori, Kan resistance) contains a constitutively expressed PylRS variant (glnS promoter) and is used in combination with the pPylT reporter plasmid (p15A ori, Tet resistance) that contains constitutively transcribed tRNA PylT (lpp promoter) and sfGFP(N150TAG)-H6 that is expressed under an inducible AraBAD promoter. The pEVOL plasmid (p15A ori, Cam resistance) contains constitutively transcribed tRNA PylT (proK promoter) as well as two copies of the PylRS variant (constitutively expressed under glnS promoter and inducible under AraBAD promoter) and is combined with the pBAD reporter plasmid (pBR322 ori, Amp resistance) that contains sfGFP(N150TAG)-H6 under an inducible AraBAD promoter. **d:** SDS-PAGE and αH6 Western blot analysis of AzGGisoK/AzGVAisoK (2 mM or 4 mM) dependent expression of full-length sfGFP-H6 in *E. coli* K12 cultured in 14 aa AI medium for indicated PylRS variants and plasmid systems.

a

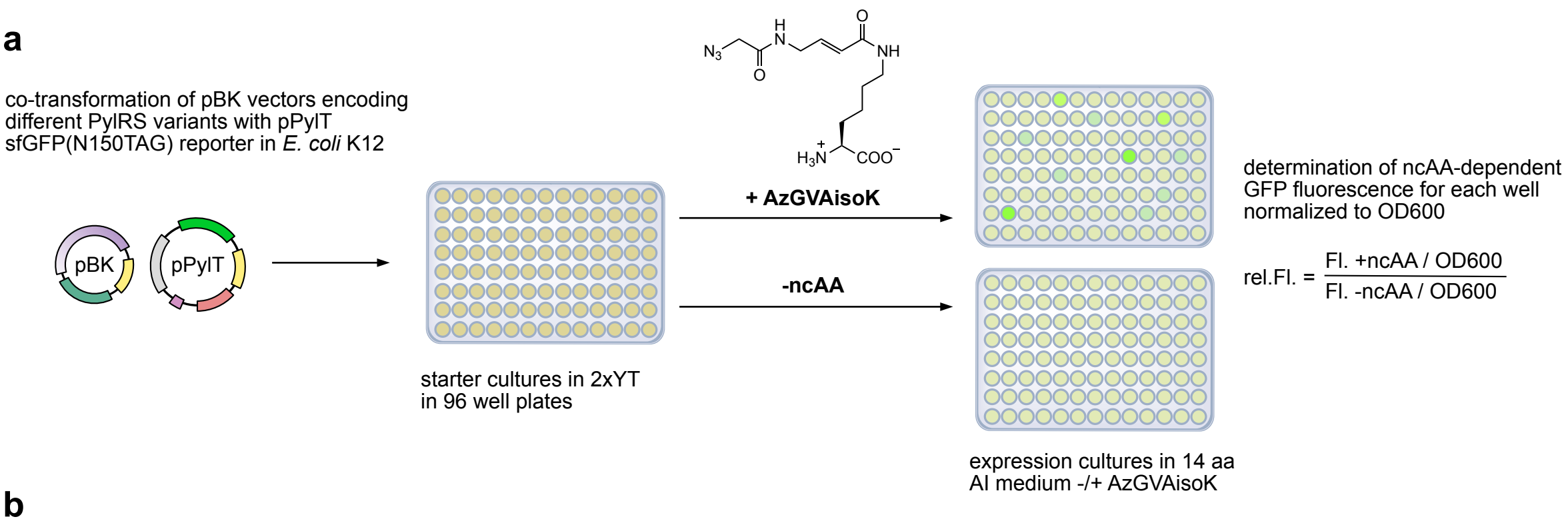

b

PlateK1 48h	1	2	3	4	5	6	7	8	9	10	11	12
A	1.49	0.65	1.54	1.35	1.47	1.37	1.76	1.60	0.66	0.73	1.28	1.21
B	1.17	1.17	1.26	1.12	1.28	1.18	1.40	0.90	1.02	1.47	1.38	1.24
C	1.11	1.22	1.06	1.04	0.96	0.92	1.37	1.39	1.32	1.36	1.01	1.21
D	1.02	1.09	0.93	0.80	1.35	0.87	1.12	1.33	1.56	1.32	1.22	1.26
E	1.21	0.88	0.82	0.70	0.81	0.85	1.01	1.19	1.33	1.28	0.81	1.12
F	0.92	0.94	0.91	1.00	0.60	0.64	0.82	1.02	0.89	1.05	2.11	1.17
G	0.92	0.94	0.91	1.00	0.98	0.87	0.99	0.92	1.03			
H												

PlateK2 48h	1	2	3	4	5	6	7	8	9	10	11	12
A	1.255	1.244	1.203	0.957	0.763	0.893	0.957	0.982	0.874	0.869	0.827	0.940
B	1.084	1.086	1.183	0.722	error	error	error	error	error	error	error	error
C	0.892	0.956	0.904	0.642	0.635	1.233	0.953	0.861	1.055	0.863	0.930	1.141
D	1.076	2.168	1.917	1.874	3.940	2.257	2.548	2.781	2.405	2.572	3.073	3.051

PlateA1 48h	1	2	3	4	5	6	7	8	9	10	11	12
A	0.525	0.613	0.802	0.731	1.156	0.739	0.727	0.618	0.599	0.618	0.742	0.740
B	0.907	1.092	0.834	0.709	0.902	0.546	0.998	0.301	0.968	0.716	1.138	1.207
C	0.514	0.540	0.568	0.604	0.637	0.539	0.907	0.755	0.671	0.620	0.802	0.761
D	0.677	0.597	0.639	0.704	0.645	0.497	0.758	0.584	0.638	0.694	0.689	0.837
E	0.578	0.554	0.592	0.787	0.567	0.613	0.677	0.619	0.693	0.760	0.601	0.622
F	0.537	0.563	0.605	0.533	0.646	0.512	0.753	0.562	0.726	0.750	0.618	0.861
G	0.640	0.515	0.905	3.073	0.736	0.622	0.761	0.626	0.694	2.001	2.018	1.845
H	0.609	0.902	0.681	0.956	0.841	0.799	0.776	0.761	0.815	0.575	0.465	0.700

Supplementary Figure S14: Investigation of established PylRS variants for amber suppression of sfGFP(N150TAG) with AzGVAisoK in fluorescence-based screening. a: Schematic overview of the workflow: *E. coli* K12 cells co-transformed with pBK/pPylT harbouring different PylRS variants (AmpR or KanR) and sfGFP(N150TAG)-H6 (TetR), respectively, are cultured in 2xYT (Kan/Tet) to grow dense starter cultures. The starter cultures are used to inoculate expression cultures in 14 aa AI medium (Amp or Kan, Tet) in duplicate and to one of the plates, 2 mM of AzGVAisoK is added. After 48 h of protein expression at 37 °C, 200 rpm, absorbance at 600 nm and GFP fluorescence are assessed. GFP fluorescence is normalized to optical density and the ncAA-dependent GFP fluorescence is calculated. **b:** Results of ncAA-dependent GFP fluorescence shows that addition of AzGVAisoK does not increase GFP fluorescence more than four-fold over background for any of the investigated PylRS variants, indicating that none of the investigated synthetases promoted efficient incorporation of AzGVAisoK.

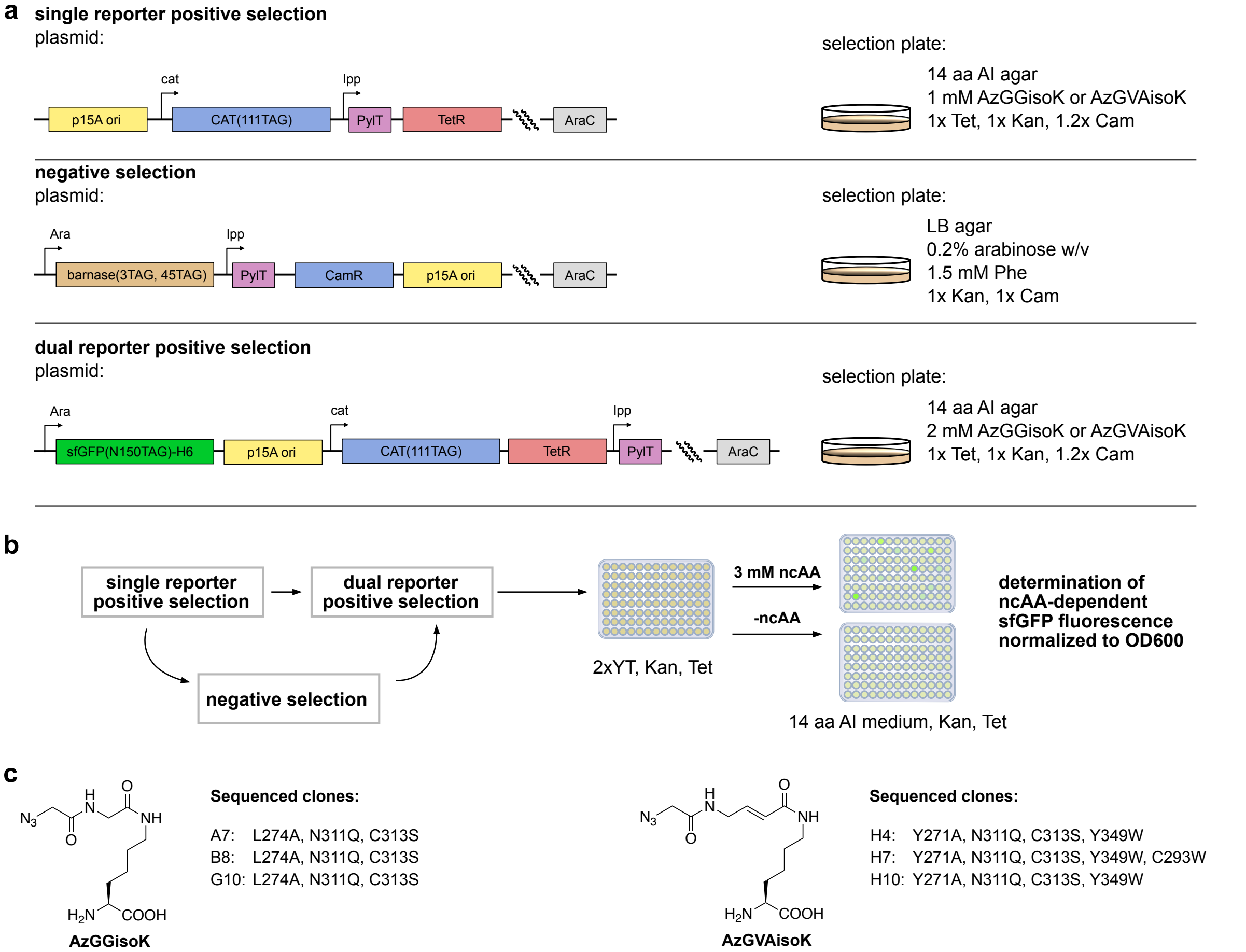

AzGGisoK (rows A–F: positive-negative-positive selection; rows G–H: positive-positive selection)

	1	2	3	4	5	6	7	8	9	10	11	12
A	1.05	0.84	1.25	0.7	9.23	1.08	20.86	10.89	0.96	16.37	8.88	1.51
B	1.33	6.85	0.92	7.61	1.13	1.12	12.92	19.2	10.88	0.82	0.85	0.83
C	0.95	0.82	1	1.29	0.76	1.14	0.81	0.98	1.23	22.94	1.05	0.95
D	0.97	0.97	1.17	0.96	1.04	1.27	0.78	0.87	0.89	1.24	1.08	0.98
E	1.12	1.07	0.81	0.94	1.17	1.26	0.86	1.14	0.89	1.02	0.92	0.99
F	1.03	0.94	1.16	1.44	0.97	1.34	0.99	1.01	0.81	1.29	0.6	0.99
G	1.63	1.21	3.47	0.92	1.15	14.3	10.58	3.18	9.37	19.23	0.96	13.59
H	7.49	12	1.81	1.06	16.14	16.59	0.98	6.97	1.03	0.88	0.68	6.08

AzGVAisoK (rows A–G: positive-negative-positive selection; row H: positive-positive selection)

	1	2	3	4	5	6	7	8	9	10	11	12
A	0.93	0.97	1.09	1.52	1.79	2.22	2.49	1.99	2.25	10.84	1.93	1.92
B	0.99	8.96	1.5	1.12	1.79	1.68	0.91	4.16	1.48	19.02	4.98	1.44
C	0.95	1.22	3.84	1.06	24.3	17.13	2.05	2.63	1.67	1.4	2.83	0.79
D	1.08	12.27	5.69	0.89	0.87	3.4	1.3	1.05	0.69	1.68	1.3	19.04
E	7.11	11.86	1.5	1.03	1.53	1.7	21.73	12.38	1.15	1.64	1.87	0.79
F	1.01	1.74	0.89	0.88	1.14	1.1	1.93	7.55	0.92	1.22	1.45	3.07
G	10.65	1	0.99	3.48	1.3	0.85	1.74	1.38	0.84	0.72	2.41	0.77
H	1.63	1.3	1.08	93.98	4.97	5.58	76.71	0.96	1.88	81.51	0.72	1.93

Supplementary Figure S15: Positive/negative selection screen for identification of AzGVAisoK-RS from PylRS variant library. a: Overview of selection plasmids and plates used in AzGVAisoK-RS selection campaign: The single reporter positive selection plasmid (p15A ori, Tet resistance) contains constitutively transcribed suppressor tRNA PylT (lpp promoter) and constitutively expressed chloramphenicol acetyl transferase with an amber stop codon at position 111 (CAT(111TAG)). The respective 14 aa AI selection plate (Kan, Tet) contains 1 mM of ncAA and Cam at 1.2-fold concentration, hence, cells that express a PylRS variant which promotes amber suppression with the ncAA (or a natural AA) express full-length CAT and can survive on the plate. The negative selection plasmid (p15A ori, Cam resistance) contains constitutively transcribed suppressor tRNA PylT (lpp promoter) and a gene encoding the toxic protein barnase with two amber stop codons (3TAG, 45TAG) under an inducible AraBAD promoter. The respective LB agar selection plate (0.2% arabinose w/v, Kan, Cam) does not contain ncAA but 1.5 mM phenylalanine (Phe). Therefore, cells that harbour a PylRS variant which promotes amber suppression with a natural AA (Phe being the most likely) will express full-length barnase and die. The dual reporter positive selection plasmid (p15A ori, Tet resistance) resembles the single reporter positive selection plasmid but features an additional open reading frame that encodes sfGFP(N150TAG) under an inducible AraBAD promoter. The respective 14 aa AI selection plate (Kan, Tet) contains 2 mM of ncAA and 1.2-fold Cam as selection pressure. Cells that harbour a PylRS variant which promotes amber suppression with the ncAA (or a natural AA) survive on the plate and form green colonies. **b:** Overview of AzGVAisoK-RS selection workflow: The *Mb*PylRS variant library encoded on pBK plasmids (pBR322 ori, Kan resistance, see Figure S13 for plasmid architecture) is transformed into electrocompetent *E. coli* DH10β cells harbouring the single reporter positive selection plasmid and positive selection is performed on the corresponding plate. The obtained sub-library is isolated and subjected to a second round of positive selection with an optional negative selection step in between. From the second positive selection plate, single colonies are used to inoculate 2xYT (Kan, Tet) precultures that are used for determination of ncAA-dependent GFP fluorescence (which indicates successful amber suppression) in 14 aa AI

cultures (Kan, Tet, -/+ 3 mM ncAA). **c:** Results of ncAA-dependent GFP fluorescence for the control selection for AzGGisoK (left) shows multiple wells with approx. 20-fold GFP fluorescence in presence of 3 mM AzGGisoK compared to -ncAA background. Sequencing of the PylRS variants present in well A7, B8 and G10 reveals convergence to *Mb*PylRS L274A, N311Q, C313S confirming successful re-selection of AzGGisoK-RS. For the AzGVAisoK selection, three wells obtained from the positive-positive selection show an averaged approx. 85-fold increase in GFP fluorescence in presence of 3 mM AzGVAisoK compared to -ncAA background. Sequencing identifies to *Mb*PylRS Y271A, N311Q, C313S, Y349W (in the following referred to as AzGVAisoK-RS) as suitable synthetase for amber suppression with AzGVAisoK.

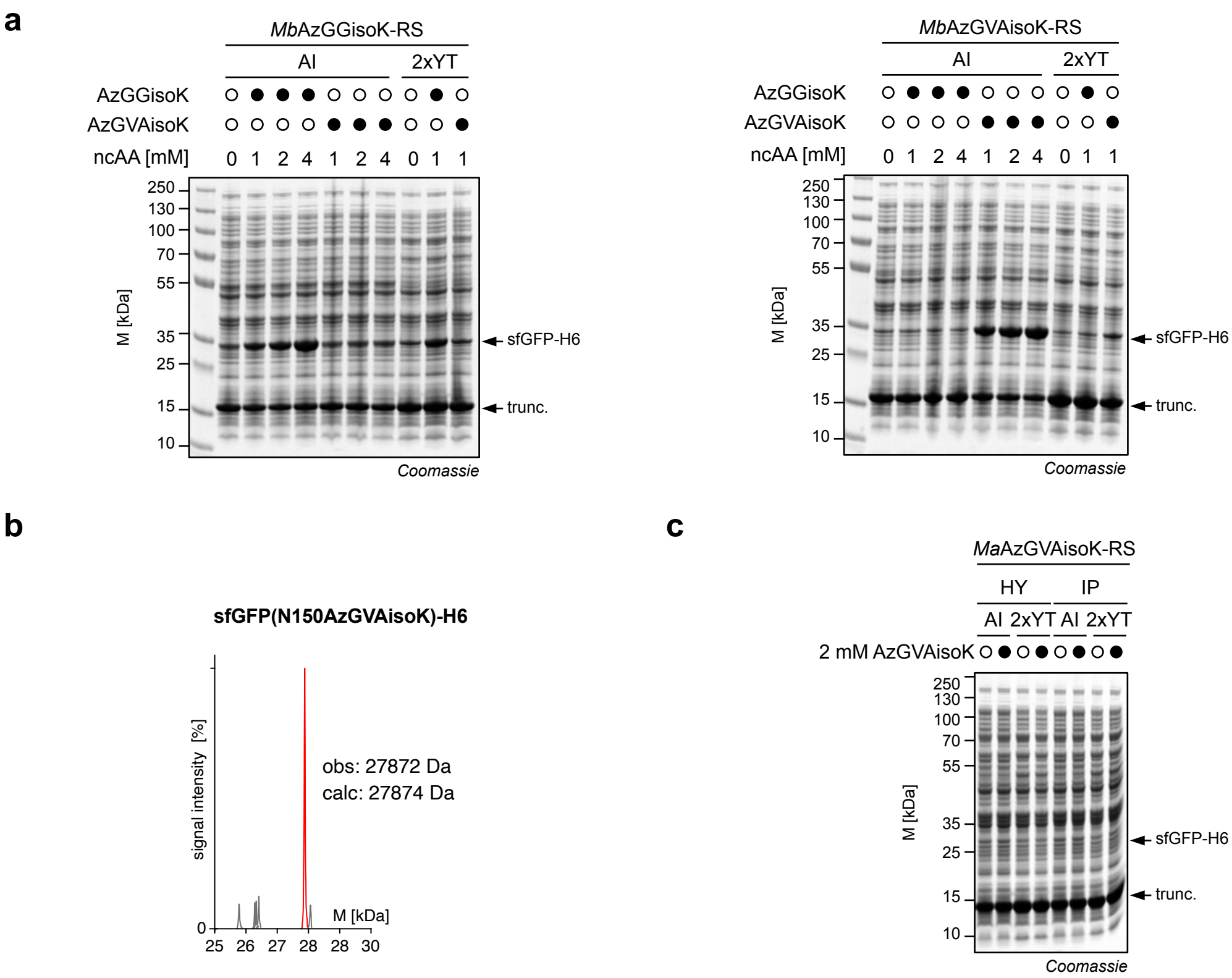

Supplementary Figure S16: Characterization of AzGVAisoK-RS. a: *E. coli* K12 cells co-transformed with pBK *Mb*AzGGisoK-RS and pPylT sfGFP(N150TAG)-H6 cultured in 14 aa AI medium (Kan, Tet) show increasing levels of full-length GFP in response to elevated AzGGisoK concentrations (1, 2, 4 mM) in the medium while supplementing the medium with AzGVAisoK does not raise GFP levels much over background as judged by SDS-PAGE. Similarly, *E. coli* K12 co-transformed with pBK *Mb*AzGVAisoK-RS and pPylT sfGFP(N150TAG)-H6 show increasing GFP levels in response to elevated AzGVAisoK but not AzGGisoK concentrations (1, 2, 4 mM). Interestingly, cells cultured in 2xYT instead of 14 aa AI medium show drastically reduced amber suppression efficiency, indicating a potential active uptake of both ncAAs by *E. coli* K12 that is outcompeted by high tryptone concentrations present in 2xYT but not 14 aa AI medium. **b:** LC-MS analysis of NiNTA-purified sfGFP(N150AzGVAisoK)-H6 expressed in *E. coli* K12 (14 aa AI medium, 37 °C, 2 mM AzGVAisoK) confirms successful AzGVAisoK-RS-promoted amber suppression with AzGVAisoK (M calc: 27874 Da, obs: 27872 Da). Furthermore, the azide moiety is not reduced to the amine when AzGVAisoK is incorporated into sfGFP at position 150 and the protein is purified without addition of reducing agents to the purification buffers. **c:** Interestingly, we found that transplanting the *Mb*AzGVAisoK-RS mutations to *Ma*PylRS did not yield a functional synthetase for amber suppression with AzGVAisoK. SDS-PAGE analysis of full-length expression of sfGFP(N150AzGVAisoK)-H6 in *E. coli* K12 showed no amber suppression in cells harbouring *Ma*PylRS (Y126A, N166Q, V168S, Y206W) without second layer mutations (denoted as “HY” in the figure) or *Ma*PylRS (Y126A, N166Q, V168S, Y206W, H227I, Y228P) with the commonly used second-layer mutations in position 227 and 228.
Black arrow with “trunc.” indicates truncation band for sfGFP(1-149).

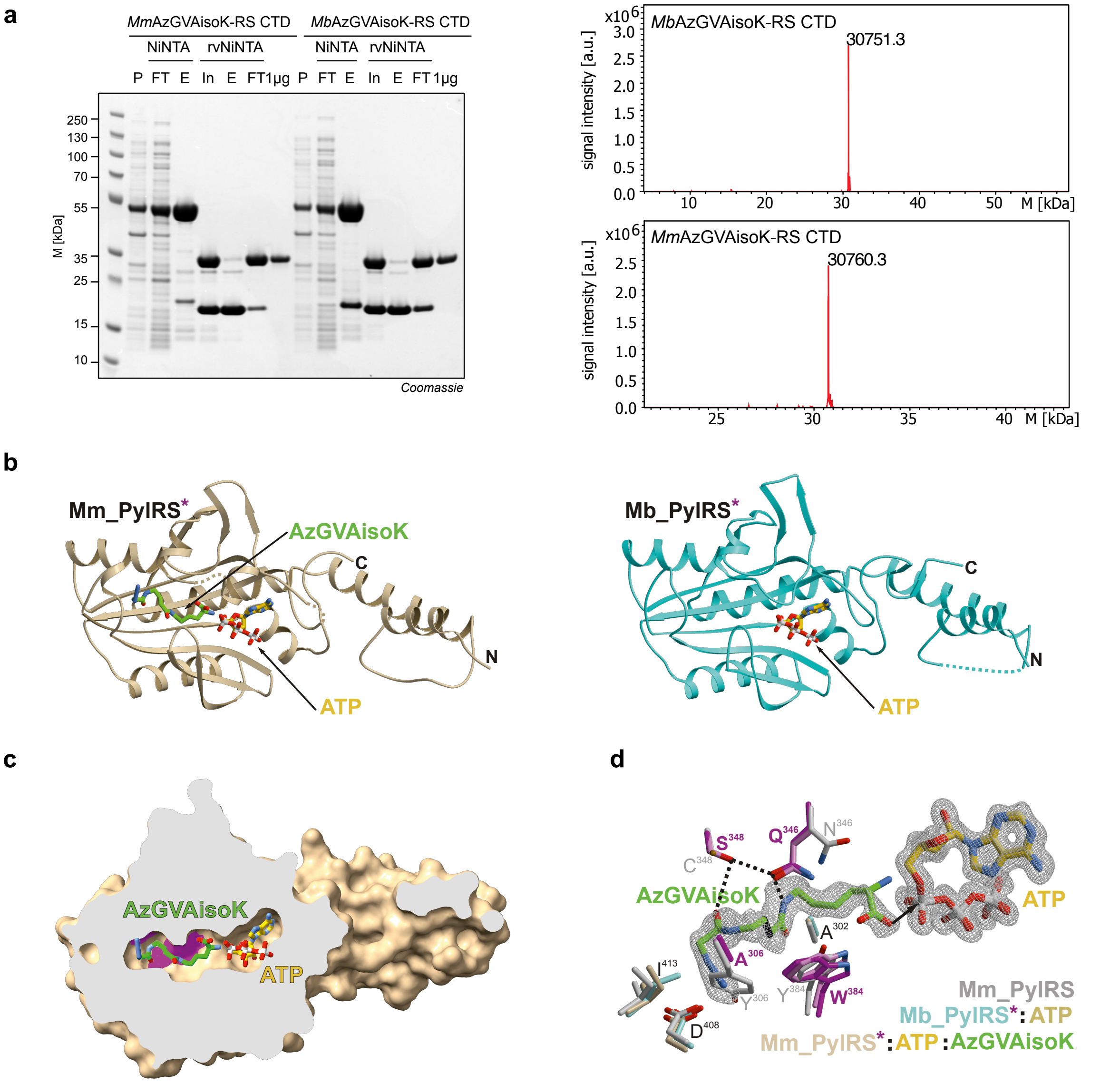

Supplementary Figure S17: Purification and structural analysis of *Mm* and *Mb*AzGVAisoK-RS C-terminal domain (CTD). a: SDS-PAGE analysis of AzGVAisoK-RS CTD purification procedure: The proteins are expressed as N-terminal H6-SUMO fusions and isolated from *E. coli* Rosetta II(De3) lysate via Ni-NTA purification. The H6-SUMO-tag is cleaved by treatment with SUMO protease and AzGVAisoK-RS is isolated by reverse Ni-NTA purification followed by SEC. Samples: P: (pellet after clearing crude lysate), FT (flow-through), E (elution), In (input), 1 µg (purified protein after SEC). SDS-PAGE shows that the purification strategy is adequate for the purification of AzGVAisoK-RS CTD and LC-MS analysis confirms isolation of intact and correct proteins (*Mb*AzGVAisoK-RS M calc: 30752 Da, obs: 30751 Da; *Mm*AzGVAisoK-RS M calc: 30761 Da, obs: 30760 Da). **b:** Ribbon representation of the C-terminal domains of *Methanosarcina mazei* AzGVAisoK-RS (Mm_PylRS*, tan; PDB ID: 30XL) and *Methanosarcina bakeri* AzGVAisoK-RS (Mb_PylRS*, cyan; PDB ID: 30XM), both optimized for AzGVAisoK accommodation. Structures are shown left and right, respectively. Both enzymes are bound to ATP (gold carbons, blue nitrogens, red oxygens), while AzGVAisoK (green carbons) is observed only in Mm_PylRS*. **c:** Sliced surface view of Mm_PylRS* highlighting engineered residues (magenta). **d:** Active site of Mm_PylRS* with the Polder mF_o-DF_c omit map contoured at 3.0σ (grey mesh) for ATP and AzGVAisoK. The black arrow indicates the position of the nucleophilic attack of the ligand's carboxylate on the ATP α-phosphate. Superposition with Mb_PylRS*:ATP (ATP carbons in light gold) and wild-type Mm_PylRS (carbons in grey; PDB ID: 2Q7E) is shown. The C=C bond of AzGVAisoK is highlighted in black and stabilized by Trp384. Ser348 and Gln346 form hydrogen bonds to the ligand backbone, interactions not possible in the wild-type enzyme, which lacks these residues. Ala306 enlarges the pocket to accommodate the N_3 group, whereas Tyr306 in the wild type causes a steric clash. Mb_PylRS* numbering follows that of Mm_PylRS*.

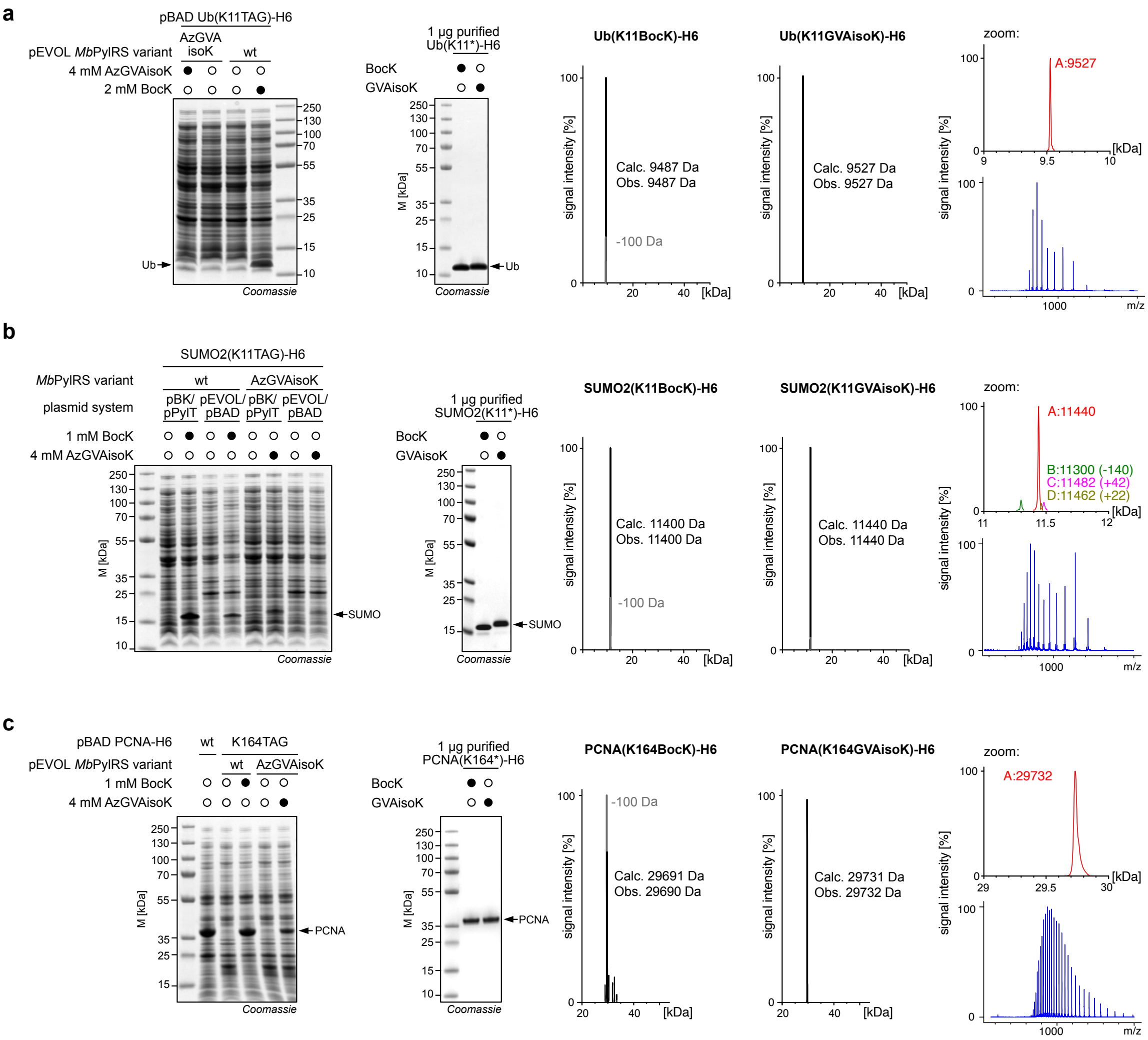

Supplementary Figure S18: Expression and purification of GVAisoK-bearing and BocK-bearing acceptor POIs Ub(K11*)-H6, SUMO2(K11*)-H6 and PCNA(K164*)-H6. a: *E. coli* K12 cells were co-transformed with pBAD Ub(K11TAG)-H6 and pEVOL *Mb*PylRS (for BocK incorporation) or pEVOL *Mb*AzGVAisoK-RS and 2xYT starter cultures were used to inoculate expression cultures in 14 aa AI medium supplemented with either 2 mM BocK or 4 mM AzGVAisoK. Protein expression was conducted at 37 °C, 200 rpm, overnight and amber suppression efficiency judged by SDS-PAGE. Protein purification via Ni-NTA resin followed by Staudinger reduction in presence of 1 mM TCEP and subsequent rebuffering to 20 mM Tris pH 7.5, 100 mM NaCl afforded Ub(K11*)-H6 amber-suppressed with BocK or GVAisoK. SDS-PAGE analysis of 1 µg of the final products confirmed the purity of the obtained proteins and LC-MS verified incorporation of BocK (M calc: 9487 Da, obs: 9487 Da and minor -100 Da species corresponding to loss of Boc during MS) and GVAisoK (M calc: 9527 Da, obs: 9527 Da). **b:** *E. coli* K12 cells were co-transformed with *Mb*PylRS or *Mb*AzGVAisoK-RS and SUMO(K11TAG)-H6 encoded by pBK/pPylT or pEVOL/pBAD plasmid systems. Aber suppression efficiency in 14 aa AI medium supplemented with 1 mM BocK or 4 mM AzGVAisoK at 37°C, 200 rpm, was evaluated by SDS-PAGE. Amber-suppression SUMO(K11*)-H6 was purified by Ni-NTA affinity chromatography and subjected to Staudinger reduction followed by rebuffering to 20 mM Tris pH 7.5, 100 mM NaCl. SDS-PAGE analysis of 1 µg of the final products confirmed the purity of the obtained proteins and LC-MS verified incorporation of BocK (M calc: 11400 Da, obs: 11400 Da and minor -100 Da species corresponding to loss of Boc during MS) and GVAisoK (M calc:11440 Da, obs: 11440 Da). **c:** *E. coli* K12 cells were co-transformed with pBAD encoding either wt PCNA-H6 or PCNA(K164TAG)-H6 and pEVOL *Mb*PylRS (for BocK incorporation) or pEVOL *Mb*AzGVAisoK-RS. Protein expression and amber suppression at 37 °C in 14 aa AI medium -/+ 1 mM BocK or 4 mM AzGVAisoK were efficient and amber-suppressed PCNA(K164*)-H6 afforded by Ni-NTA purification, Staudinger reduction and rebuffering to 20 mM Tris pH 7.5, 100 mM NaCl was obtained in sufficient purity as judged by SDS-PAGE. Finally, LC-MS analysis confirmed incorporation of BocK (M calc: 29691 Da, obs: 29691 Da and -100 Da species corresponding to loss of Boc during MS) and GVAisoK (M calc: 29731 Da, obs: 29732 Da).
Zoom-ins of the deconvoluted mass spectra and m/z are provided for GVAisoK-bearing proteins.

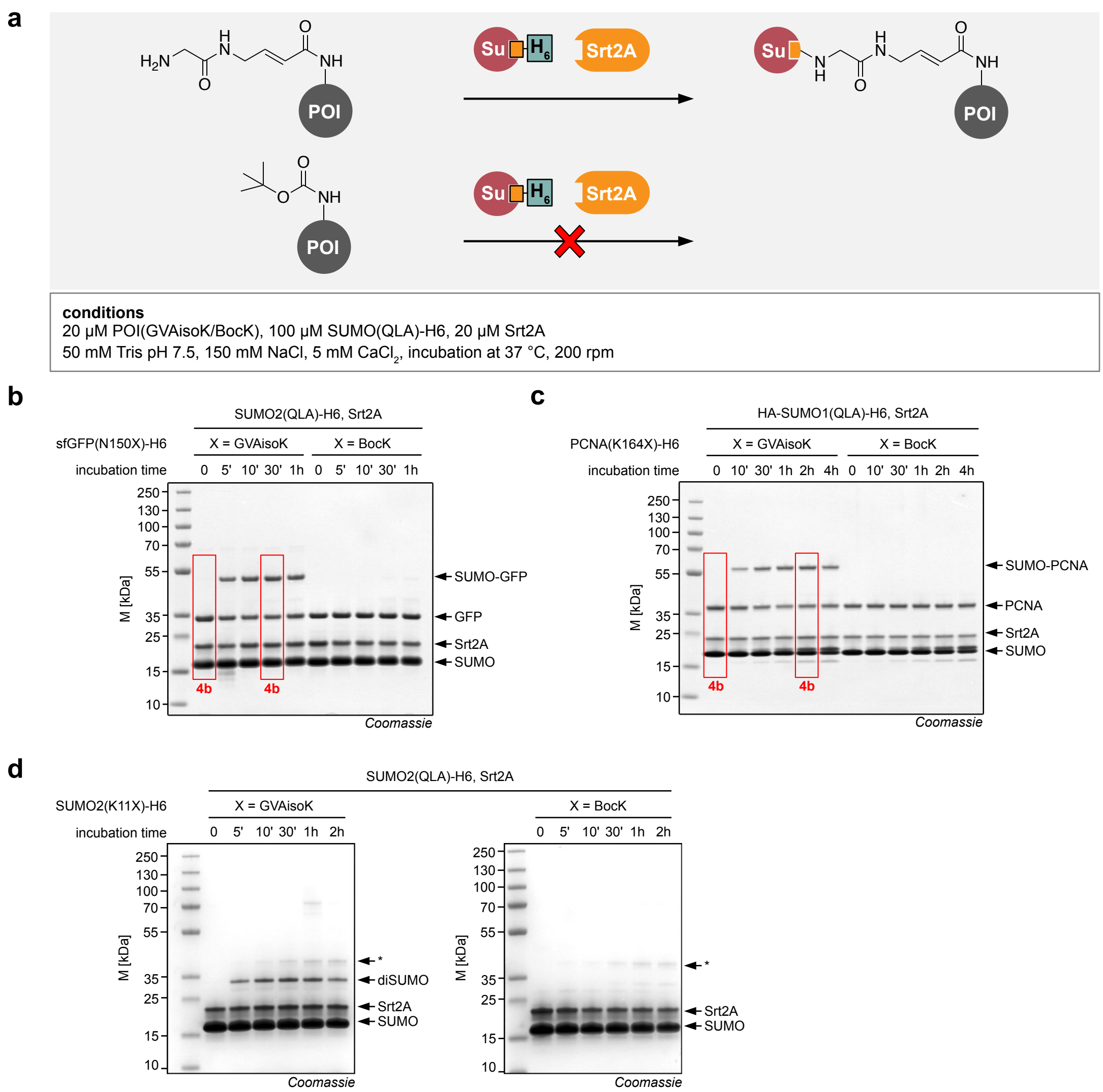

Supplementary Figure S19: Sortylation exclusively SUMOylates the free amine of GVAisoK presented by amber-suppressed POIs. a: Schematic display and conditions of sortylation reactions: Incubation of 100 µM SUMO1/2(QLA)-H6 and 20 µM Srt2A with 20 µM POI(GVAisoK) but not POI(BocK) in 50 mM Tris pH 7.5, 150 mM NaCl, 5 mM $CaCl_2$ at 37 °C, 200 rpm, leads to SUMOylation of POI. **b-d:** SDS-PAGE analysis of time course of sortylation reactions shows fast efficient sortylation of GVAisoK-bearing sfGFP(N150*)-H6 (in **b**), PCNA(K164*)-H6 (in **c**) and SUMO2(K11*)-H6 (in **d**) while the respective BocK controls remain unmodified. In the sortylation reaction with the SUMO2(K11GVAisoK)-H6 acceptor POI, the thioester between Srt2A and the donor SUMO2(QLA) is visible in the SDS-PAGE and marked with an asterisk.
Red boxes indicate sections of the SDS-PAGE gels shown in main figure 4b.

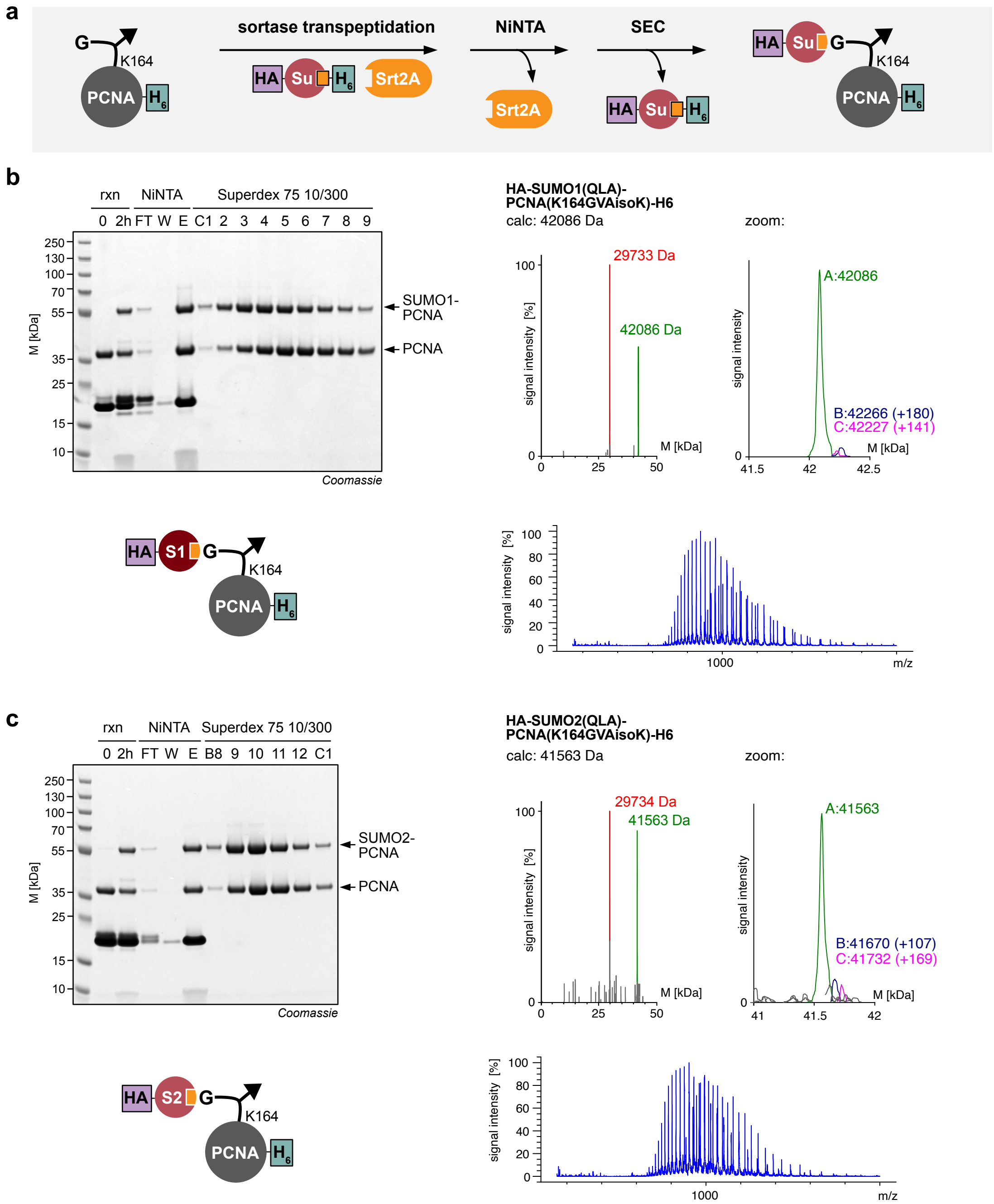

Supplementary Figure S20: Preparative generation of HA-SUMO1/2(QLA)-PCNA(K164GVAisoK)-H6. a: Overview of workflow for generation and isolation of GVAisoK-linked HA-SUMO1/2(QLA)-PCNA(K164GVAisoK)-H6: Sortylation was conducted by incubation of 100 µM SUMO1/2(QLA)-H6 donor, 20 µM PCNA(K164GVAisoK)-H6 and 20 µM Srt2A in 50 mM Tris pH 7.5, 150 mM NaCl, 5 mM $CaCl_2$ at 37 °C, 200 rpm, for 2 h. NiNTA purification was used to remove Srt2A and SEC (20 mM Tris pH 7.5, 100 mM NaCl) was used to remove remaining SUMO1/2(QLA)-H6 donor from the product. PCNA is a trimeric protein, and because the sortylation reaction does not reach full conversion, the resulting product consists of statistically mixed trimers containing both SUMOylated and non-SUMOylated PCNA. **b, c:** SDS-PAGE analysis of samples taken during sortylation reaction (rxn), NiNTA purification (FT: flow-through, W: wash, E: elution) and SEC purification of SUMO-PCNA for HA-SUMO1(QLA) donor (in **b**) or HA-SUMO2(QLA) donor (in **c**). LC-MS analysis of the obtained products shows presence of non-SUMOylated PCNA (red peak, M calc: 29731 Da, obs: 29733 Da and 29734 Da) and HA-SUMO1(QLA)-PCNA(K164GVAisoK)-H6 (green peak in b, M calc: 42086 Da, obs: 42086 Da) or SUMO2(QLA)-PCNA(K164GVAisoK)-H6 (green peak in c, M calc: 41563 Da, obs: 41563 Da).

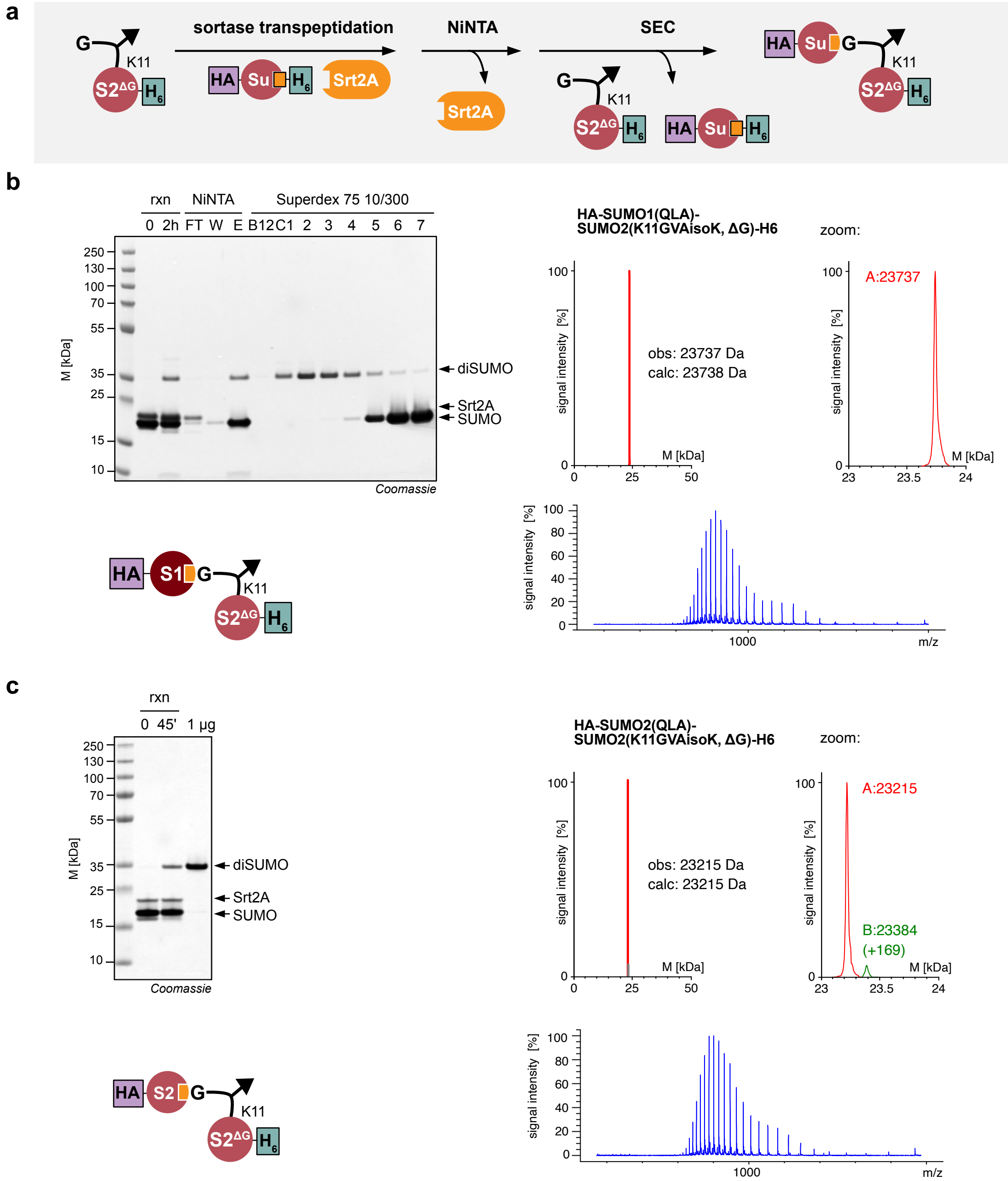

Supplementary Figure S21: Preparative generation of HA-SUMO1/2(QLA)-SUMO2(K11GVAisoK, ΔG)-H6. a: Overview of workflow for generation and isolation of GVAisoK-linked diSUMO2: Sortylation was conducted by incubation of 100 µM SUMO1/2(QLA)-H6 donor, 20 µM SUMO2(K11GVAisoK, ΔG)-H6 and 20 µM Srt2A in 50 mM Tris pH 7.5, 150 mM NaCl, 5 mM $CaCl_2$ at 37 °C, 200 rpm, for 2 h (SUMO1 donor) or 45 min (SUMO2 donor), respectively. NiNTA purification was used to remove Srt2A and SEC in 20 mM Tris pH 7.5, 100 mM NaCl was used to remove remaining SUMO1/2(QLA)-H6 donor from the product. **b:** SDS-PAGE analysis of samples taken during sortylation reaction (rxn), NiNTA purification (FT: flow-through, W: wash, E: elution) and SEC purification; and LC-MS analysis of obtained HA-SUMO1(QLA)-SUMO2(K11GVAisoK, ΔG)-H6 (M calc: 23738 Da, obs: 23737 Da). **c:** SDS-PAGE analysis of samples taken during sortylation reaction (rxn) and of 1 µg of the final isolated product as well as LC-MS analysis of obtained HA-SUMO2(QLA)-SUMO2(K11GVAisoK, ΔG)-H6 (M calc: 23215 Da, obs: 23215 Da).

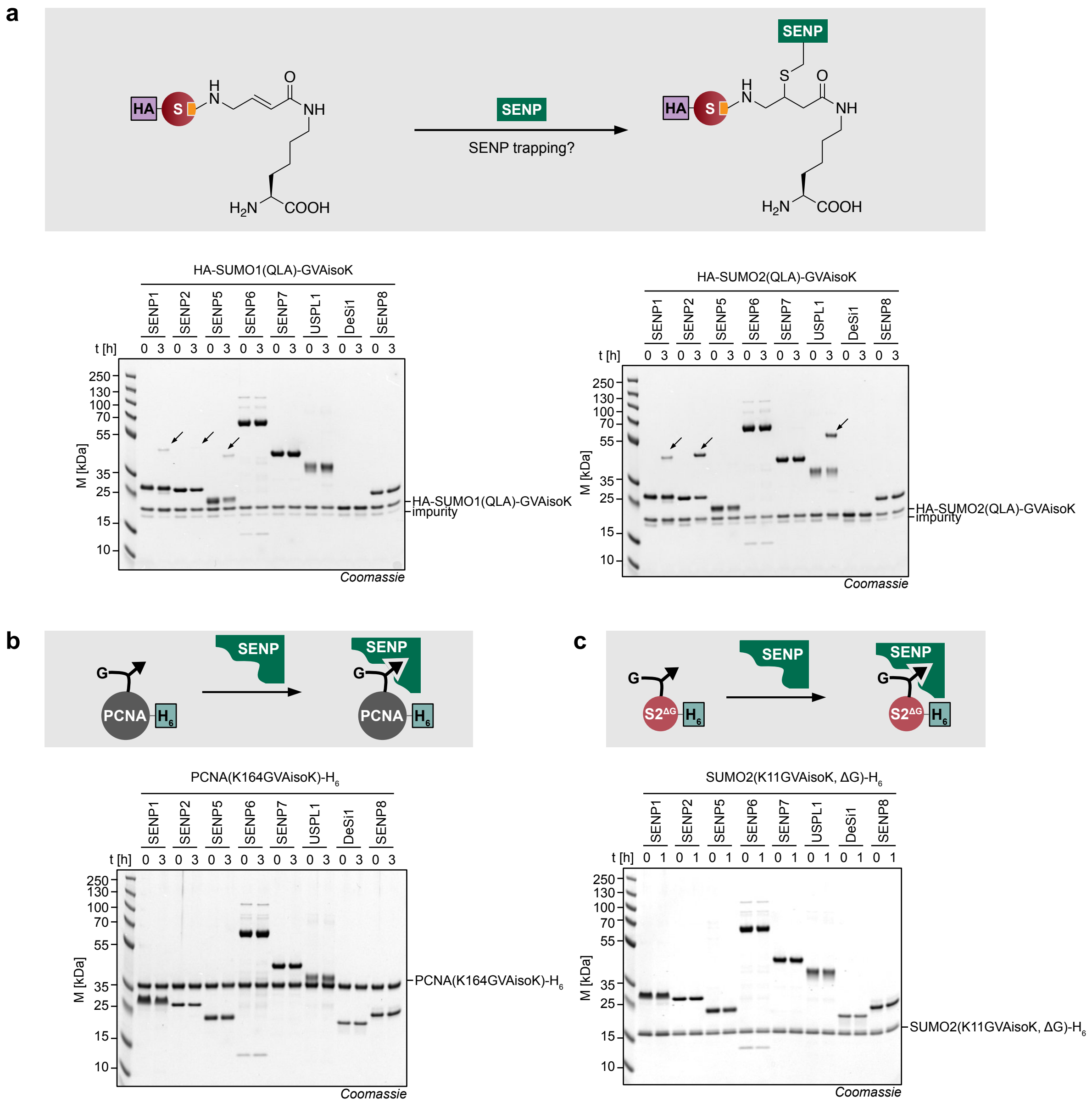

Supplementary Figure S22: "No acceptor control" and "no donor control" SENP trapping assay control for GVAisoK-linked ABPs. a: The "no acceptor control" SENP assays were conducted by incubation of 15 µM HA-SUMO1/2(QLA)-GVAisoK with 15 µM SENP1(CD), SENP2(CD), SENP5(CD), SENP6(CD), SENP7(CD), USPL1, DeSi1, or SENP8 in 20 mM Tris pH 7.5, 100 mM NaCl, 0.5 mM TCEP at 37 °C for 3 h. SDS-PAGE analysis shows covalent complex formation of HA-SUMO1(QLA)-GVAisoK with SENP1, SENP2 and SENP5 as well as HA-SUMO2(QLA)-GVAisoK with SENP1, SENP2, and USPL1. **b,c :** The "no donor control" SENP assays for GVAisoK-bearing acceptor POIs were conducted by incubating 15 µM PCNA(K164GVAisoK)-H6 (in **b**) or 15 µM SUMO2(K11GVAisoK, ΔG)-H6 (in **c**) with 15 µM SENP1(CD), SENP2(CD), SENP5(CD), SENP6(CD), SENP7(CD), USPL1, DeSi1, or SENP8 in 20 mM Tris pH 7.5, 100 mM NaCl, 0.5 mM TCEP at 37 °C for 3 h or 1 h, respectively. SDS-PAGE analysis shows no covalent complex formation for any assay.

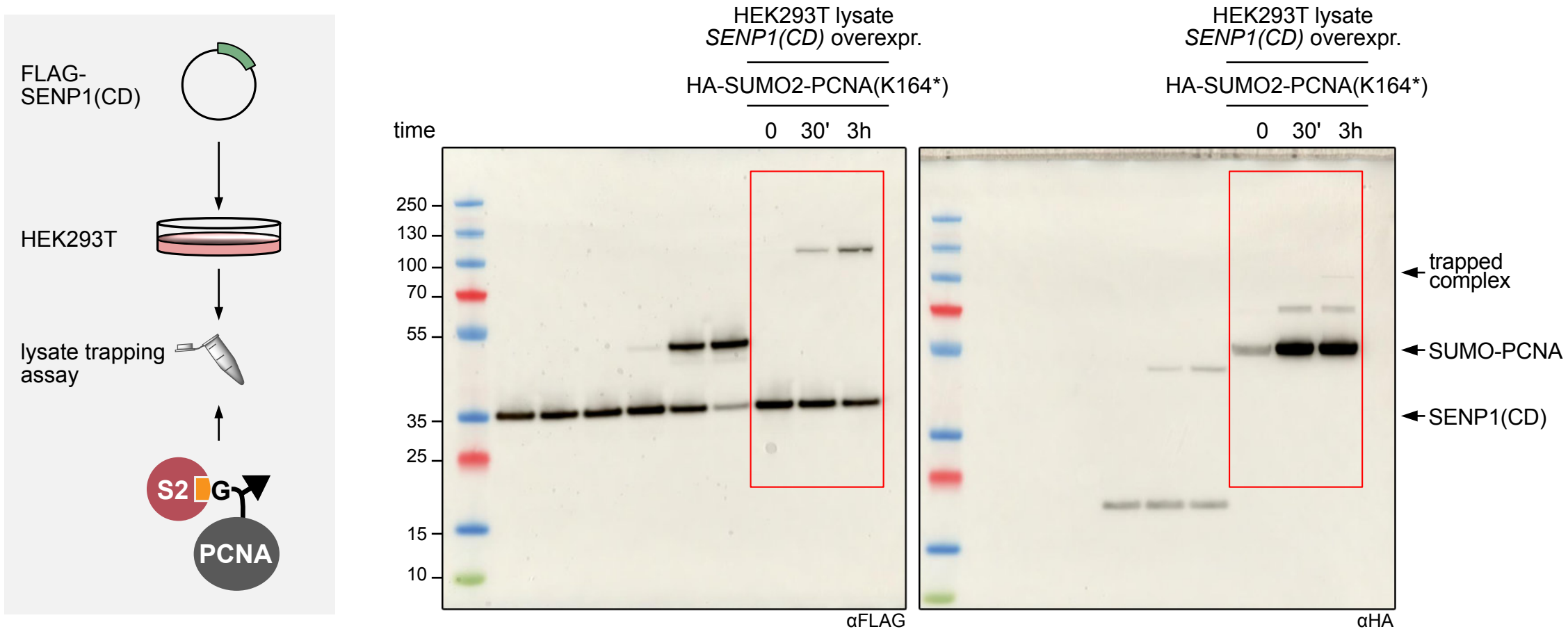

Supplementary Figure S23: Addition of assembled HA-SUMO2(QLA)-PCNA(K164GVAisoK)-H6 to HEK293T lysates leads to trapping of overexpressed FLAG-SENP1. HEK293T lysate (total protein concentration: 4 mg/mL; buffer: 50 mM Tris pH 7.5, 150 mM NaCl, 0.5% Triton-X-100 (w/v), 1x Roche protease inhibitor) prepared from cells overexpressing FLAG-SENP1(CD) were incubated -/+ 5 µM HA-SUMO2(QLA)-PCNA(K164GVAisoK)-H6 at 37 °C and SDS samples were taken at t = 0, 30 min, and 3 h. Samples were applied to SDS-PAGE (Bolt 4-12% Bis-Tris Plus gels and active site cysteine-dependent SENP1 trapping was confirmed by Western blot against FLAG and HA.

Red boxes indicate the relevant sections of the Western blots.

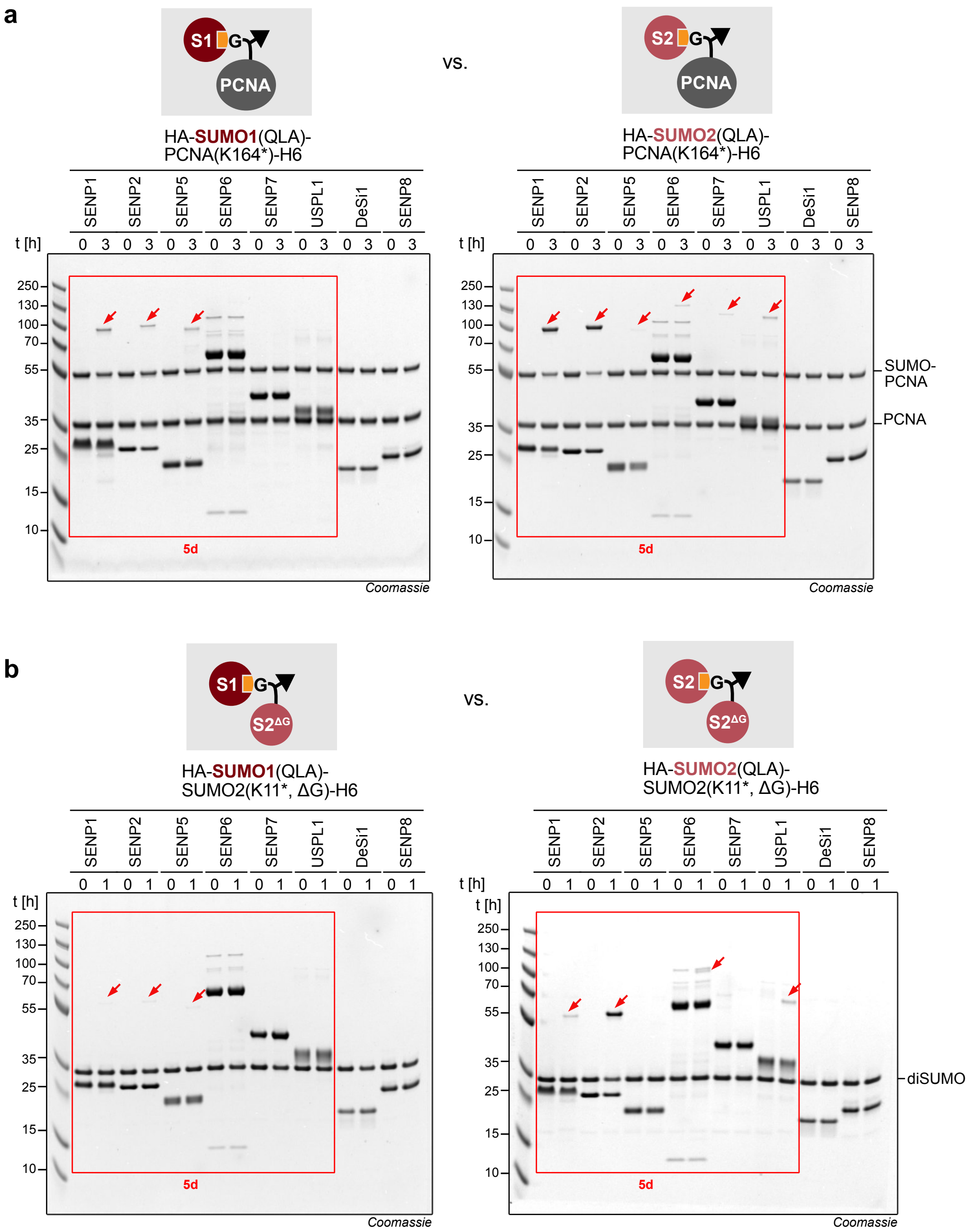

Supplementary Figure S24: Profiling of SENPs with different GVAisoK-linked ABPs. a: Comparative SENP trapping assay with SUMO1- or SUMO2-modified PCNA(K164GVAisoK) were conducted by incubation of 15 µM SUMO1/2(QLA)-PCNA(K164GVAisoK)-H6 with 15 µM SENP1(CD), SENP2(CD), SENP5(CD), SENP6(CD), SENP7(CD), USPL1, DeSi1, or SENP8 in 20 mM Tris pH, 100 mM NaCl, 0.5 mM TCEP at 37 °C for 3 h. SDS-PAGE analysis shows distinct trapping patterns, indicated by red arrows in the figure. **b:** Comparative SENP trapping assay with SUMO1- or SUMO2-modified SUMO2(K11GVAisoK, ΔG)-H6 were conducted by incubation of 15 µM SUMO1/2(QLA)- SUMO2(K11GVAisoK, ΔG)-H6 with 15 µM SENP1(CD), SENP2(CD), SENP5(CD), SENP6(CD), SENP7(CD), USPL1, DeSi1, or SENP8 in 20 mM Tris pH, 100 mM NaCl, 0.5 mM TCEP at 37 °C for 1 h. SDS-PAGE analysis shows distinct trapping patterns, indicated by red arrows in the figure.
Red boxes indicate sections of the SDS-PAGE gels shown in main figure 5d.

2. Experimental Procedures

2.1 General Remarks

E. coli DH10β cells (NEB) were used for applications demanding high transformation efficiencies such as cloning and the PylRS library selection campaign. *E. coli* BL21(De3) cells (NEB) and *E. coli* Rosetta II(De3) cells (Sigma Aldrich/Novagen) were used for recombinant protein expression from pET vectors. *E. coli* K12 (former GE Healthcare) were used for recombinant protein expression under araBAD promoters and production of amber-suppressed proteins.

Rich media 2xYT (2x yeast extract and tryptone), LB (Lysogeny broth, Lennox formulation) and SOB (Super Optimal Broth) (Carl Roth, Sigma Aldrich, or LLG Labware) were prepared as recommended by the distributor, autoclaved and stored at rt. SOC (Super Optimal Broth with catabolite repression) was prepared from SOB medium by addition of glucose to 20 mM. Defined 14 aa AI medium (14 amino acid arabinose auto-inducing medium) was prepared analogous to the previously described 18 aa AI medium[1] with the alteration that it only contained 14 amino acids: Ala, Asp, Gly, His, Ile, Leu, Met, Asn, Pro, Arg, Ser, Thr, Val, Trp.

The standard working concentration for antibiotics were 100 µg/mL ampicillin (Amp), 50 µg/mL kanamycin (Kan), 50 µg/mL chloramphenicol (Cam), and 17 µg/mL tetracycline (Tet). Instances where different antibiotic concentrations were used are specified in the respective procedures.

Codon optimized gene strings (Twist) were cloned into plasmid backbones by digesting with restriction enzymes (NEB) and ligation with T4 DNA ligase (NEB) according to manufacturer's protocol. Plasmids used in this study are listed in Table S4.

SDS-PAGE was performed with self-prepared gels (15% or 17%) or commercially available Bolt™ 4-12% Bis-Tris Plus gels (Invitrogen) in 1xMES Running Buffer (50 mM MES, 50 mM Tris, 1 mM EDTA, 0.1% SDS w/v, pH not adjusted and approx. 7.3), or commercially NuPAGE 3-8% Tris-Acetate gels (Invitrogen) in 1xTricine Running Buffer (50 mM Tricine, 50 mM Tris, 0.1% SDS w/v, pH not adjusted). Samples were prepared by mixing with 4xSDS Laemmli Loading Buffer (50 mM Tris pH 6.8, 40% glycerol (w/v), 8% SDS (w/v), 5% β-mercaptoethanol (v/v), 0.4% Bromophenol blue (w/v)) in a ratio sample:buffer of 3:1 or as stated in the respective procedure. Coomassie staining was performed with QuickBlue Protein Stain (Lubio Biosciences) according to manufacturer's protocol.

HEK293T cells (Horizon Discovery) and MCF-7 cells (ATCC) were cultured in DMEM (Dulbecco's Modified Eagle Medium; high glucose, GlutaMAX™, pyruvate; Thermo Fisher Scientific) supplemented with 10% FBS (fetal bovine serum; FBS Good, Pan Biotech) (w/v) and 1x Penicillin-Streptomycin (100x Penicillin-Streptomycin (10,000 U/mL), Thermo Fisher Scientific) at 37 °C and 5% CO_2.

Transient transfection of mammalian cells was performed with transfection agent PEI (Polyethylenimine, Linear, MW 25000, Transfection Grade, Polysciences) that was dissolved in water to 1 mg/mL as a stock solution. Transfection mixes were prepared with 3 µL PEI stock solution and 100 µL Opti-MEM (Opti-MEM™ I Reduced Serum Medium, Thermo Fisher Scientific) per 1 µg of DNA. All components were mixed by vortexing and transfection mixes were incubated at rt for 20 min before dropwise addition to the culture vessel.

Chemicals and solvents were purchased from commercial distributors (Sigma Aldrich, Carbolution, Fisher Scientific, Tokyo Chemical Industry, Fluorochem, Carl Roth) and used without further purification. Unless stated otherwise, all reactions were set up at rt and atmospheric pressure with stirring.

Flash column chromatography was either performed manually using self-packed silica gel columns (pore size 60 Å, 230-400 mesh, 40-63 µm particle size, Sigma Aldrich) or with an automated Pure C-850 Flash System (Büchi) equipped with commercially available cartridges.

NMR spectra were recorded in deuterated solvents at rt on a Bruker AVHD 300 MHz, a Bruker AVHD 400 MHz and a Bruker AVHD 500 MHz provided by TUM NMR service or on a Bruker Avance III 400 MHz provided by the ETH Zurich NMR facilities. Spectra were analyzed with MestReNova software (Mestrelab Research S.L.). Chemical shifts δ are reported in ppm (parts per million) and were referenced to solvent peaks.

2.2 Protein Expression and Purification

2.2.1 Expression and purification of SUMO donors for sortase-mediated ligation

H6-tagged SUMO donors for sortase-mediated ligation were expressed in *E. coli* Rosetta II(De3) and isolated via NiNTA affinity chromatography followed by SEC.

First, chemically competent *E. coli* Rosetta II(De3) cells were transformed with the respective pET expression vector via heat shock and a starter culture was prepared in 50 mL 2xYT (Amp, Cam). The starter culture was incubated at 37 °C, 200 rpm, until OD600 reached 0.9-1.1, and then used to inoculate the expression culture ($V_{expression}$ = 0.5-3 L; 2xYT, ½ Amp, ½ Cam) to an OD600 of 0.01-0.05. The culture was incubated at 37 °C, 200 rpm, until OD600 reached 0.6-0.8, before protein expression was induced by the addition of IPTG to a final concentration of 0.5 mM. The expression culture was then incubated at 18 °C for 16 h (or at 37 °C for 3 h) and cells harvested (5000 xg, 4 °C, 30 min) with OD600 typically reaching 1.8-4.5.

The obtained pellet was resuspended in NiNTA Lysis Buffer (20 mM Tris pH 8, 300 mM NaCl, 30 mM imidazole, 0.5 mM PMSF, DNaseI, cOmplete™ protease inhibitor (Roche, 1 tablet per 100 mL)), cells were mechanically disrupted using a CF1 Cell Disruptor at 30 kPsi (Constant Systems) and the crude lysate cleared by centrifugation (14500 xg, 4 °C, 25 min). Cleared lysate was incubated with NiNTA

resin (Ni Sepharose 6 Fast Flow, Cytiva; V_{slurry} = 2 CV = 1/500 * $V_{expression}$) at 4 °C on a tube roller for 1 h before the suspension was transferred to a fritted plastic gravity flow column. The resin was washed three times with 15 CV NiNTA Wash Buffer (20 mM Tris pH 8, 300 mM NaCl, 30 mM imidazole) before H6-tagged SUMO was eluted by sequential addition of four times 2 CV NiNTA Elution Buffer (20 mM Tris pH 8, 300 mM NaCl, 300 mM imidazole). Obtained elution fractions were pooled and further purified via SEC on a HiLoad 16/600 Superdex75 prep grade column (Cytiva; SEC buffer: 50 mM Tris pH 7.5, 150 mM NaCl, 5 mM $CaCl_2$). Fractions that contained the target protein were pooled and concentrated to up to 3 mM by spin filtration (3 kDa cut-off Amicon Ultra, Merck Millipore). Aliquots were flash frozen in liquid nitrogen and stored at -80 °C until further use.
Protein yields typically averaged 30 mg of SUMO-H6 per litre of expression culture.

2.2.2 Expression of HA-SUMO(ΔG)-intein constructs and purification of HA-SUMO(ΔG)-MESNa thioesters

Intein (MxeGyrA) and chitin binding domain (CBD) fusions HA-SUMO(ΔG)-MxeGyrA-CBD were expressed in *E. coli* BL21(De3) and isolated via chitin resin followed by on-column thiolysis with MESNa.

Chemically competent *E. coli* BL21(De3) cells were transformed with the respective pTXB1 expression vector via heat shock and a starter culture was prepared in 10 mL 2xYT (Amp). After overnight incubation at 37 °C, 200 rpm, the dense starter culture was used to inoculate 200 mL 2xYT (Amp) to OD600 of 0.02. The expression culture was incubated at 37 °C, 200 rpm, until OD600 reached 0.6, then protein expression was induced by addition of IPTG to 0.5 mM and the culture was further incubated at 30 °C, 200 rpm, for 3 h. Cells were harvested (4000 xg, 4 °C, 10 min) with OD600 typically reaching approx. 1.5.

The cell pellet was resuspended in 35 mL Intein Lysis Buffer (50 mM MOPS pH 6.8, 500 mM NaCl, 0.1 mM EDTA, 0.1% (v/v) Triton-X-100, DNaseI, cOmplete™ protease inhibitor (Roche, 1 tablet per 100 mL)) and subjected to pulsed sonication for cell disruption (Branson SFX 250 Sonifier, 6.5 mm tip, 24% amplitude, 4 min, 1 s on ↔ 2 s off) after which the crude lysate was cleared by centrifugation (14500 xg, 4 °C, 30 min). Cleared lysate was incubated with equilibrated chitin resin (NEB; 8 mL slurry) at 4 °C on a tube roller for 2 h, before the suspension was transferred to a fritted plastic gravity flow column. The resin was washed with 160 mL Intein Wash Buffer (50 mM MOPS pH 6.8, 500 mM NaCl, 0.1 mM EDTA) and 16 mL Intein Cleavage Buffer (50 mM MOPS pH 6.8, 500 mM NaCl, 0.1 mM EDTA, 100 mM MESNa) before the flow was stopped and the column was incubated at 4 °C for 72 h for thiolysis. Afterwards, the column was opened again and the elution collected with an additional 3 CV of Intein Cleavage Buffer. Subsequent SEC via a 10/300 GL Superdex 75 Increase column (Cytiva, buffer: 50 mM MOPS pH 6.8, 150 mM NaCl) afforded HA-SUMO(ΔG)-MESNa thioester that was concentrated to up to 500 µM by spin filtration (3 kDa cut-off Amicon Ultra, Merck Millipore). Aliquots were flash frozen in liquid nitrogen and stored at -80 °C until further use.

Protein yields were mostly limited by the low binding capacity of the chitin resin, but remained in the multi-milligram range per litre of expression culture.

2.2.3 Expression and purification of SENP1(CD), SENP2(CD), SENP5(CD) and SENP2(CD, C548A)

Catalytic domains of SENP1, SENP2, SENP5, and the inactive mutant SENP2(C548A) were expressed in *E. coli* Rosetta II(De3) and isolated via NiNTA affinity chromatography followed by SEC.

Chemically competent *E. coli* Rosetta II(De3) cells were transformed with the respective pET expression vector via heat shock, a starter culture was prepared in 50 mL 2xYT (Kan, Cam) and incubated over night at 37 °C, 200 rpm. The starter culture with OD600 0.8-1.0 was used to inoculate the expression culture ($V_{expression}$ = 0.5 L for SENP1/2 or 3 L for SENP5; 2xYT, ½ Kan, ½ Cam) to an OD600 of 0.01-0.05. The culture was incubated at 37 °C, 200 rpm, until OD600 reached 0.8, before protein expression was induced by the addition of IPTG to a final concentration of 0.5 mM. The expression culture was then incubated at 18 °C for 16 h and cells harvested (5000 xg, 4 °C, 30 min) with OD600 typically reaching approx. 2-4.

The obtained pellet was resuspended in 100 mL SENP Lysis Buffer (20 mM Tris pH 8, 300 mM NaCl, 30 mM imidazole, 2 mM PMSF, DNaseI, 0.5 mM DTT), cells were mechanically disrupted using a CF1 Cell Disruptor at 30 kPsi (Constant Systems) and the crude lysate cleared by centrifugation (14500 xg, 4 °C, 60 min). Cleared lysate was incubated with NiNTA resin (Ni Sepharose 6 Fast Flow, Cytiva; V_{slurry} = 2 CV = 1/1000 * $V_{expression}$ for SENP1/2 or 1/2000 * $V_{expression}$ for SENP5) at 4 °C on a tube roller for 1 h before the suspension was transferred to a fritted plastic gravity flow column. The resin was washed three times with 20 CV SENP NiNTA Wash Buffer (20 mM Tris pH 8, 300 mM NaCl, 30 mM imidazole, 0.5 mM DTT) before H6-tagged SENP was eluted by sequential addition of three times 2 CV SENP NiNTA Elution Buffer (20 mM Tris pH 8, 300 mM NaCl, 300 mM imidazole, 0.5 mM DTT). Subsequent SEC via a 10/300 GL Superdex 75 Increase column (Cytiva, buffer: 20 mM Tris pH 7.5, 100 mM NaCl, 2.5 mM DTT) afforded SENP(CD) that was concentrated to up to 100 µM by spin filtration (10 kDa cut-off Amicon Ultra, Merck Millipore). Aliquots were flash frozen in liquid nitrogen and stored at -80 °C until further use.

Protein yields typically averaged 8-10 mg per litre of expression culture for SENP1/2(CD) and 2 mg per litre of expression culture for SENP5(CD).

2.2.4 Expression and purification of SENP6(CD) and SENP7(CD)

Catalytic domains of SENP6 and SENP7 were expressed in *E. coli* Rosetta II(De3) and isolated via NiNTA affinity chromatography followed by anion exchange chromatography.

Chemically competent *E. coli* Rosetta II(De3) cells were transformed with the respective pET expression vector via heat shock, a starter culture was prepared in 50 mL 2xYT (Kan, Cam) and incubated over night at 37 °C, 200 rpm. The starter culture with OD600 0.8-1.2 was used to inoculate

the expression culture (3 L 2xYT, ½ Kan, ½ Cam) to an OD600 of 0.01. The culture was incubated at 37 °C, 200 rpm, until OD600 reached 0.6, before protein expression was induced by the addition of IPTG to a final concentration of 0.2 mM. The expression culture was then incubated at 16 °C for 18-20 h and cells harvested (5000 xg, 4 °C, 30 min) with OD600 typically reaching 1.6-3.5.

The obtained pellet was resuspended in 100 mL SENP6/7 Lysis Buffer (20 mM Tris pH 8, 350 mM NaCl, 30 mM imidazole, 20% (w/v) sucrose, 0.1% (v/v) NP-40, 1 mM β-mercaptoethanol, 1 mM PMSF, DNaseI), cells were mechanically disrupted using a CF1 Cell Disruptor at 30 kPsi (Constant Systems) and the crude lysate cleared by centrifugation (14500 xg, 4 °C, 45 min). Cleared lysate was incubated with NiNTA resin (Ni Sepharose 6 Fast Flow, Cytiva; V_{slurry} = 1.5 mL = 2 CV) at 4 °C on a tube roller for 1 h before the suspension was transferred to a fritted plastic gravity flow column. The resin was washed three times with 20 CV SENP NiNTA Wash Buffer (20 mM Tris pH 8, 300 mM NaCl, 30 mM imidazole, 0.5 mM DTT) before H6-tagged SENP was eluted by sequential addition of three times 2 CV SENP NiNTA Elution Buffer (20 mM Tris pH 8, 300 mM NaCl, 300 mM imidazole, 0.5 mM DTT). The pooled elution fractions were then diluted 1:9 with SENP AnEx Buffer A (25 mM Tris pH 8, 1 mM β-mercaptoethanol) and subjected to anion exchange chromatography via a HiTrap Capto DEAE 1 mL column (Cytiva). The column was first equilibrated with 10 mL SENP AnEx Buffer A, then sample was applied via the sample pump, the column was washed with 10 mL SENP AnEx Buffer A and proteins were eluted with a 45 mL linear gradient 0→50% SENP AnEx Buffer B (25 mM Tris pH 8, 1 mM β-mercaptoethanol, 1 M NaCl) followed by a 5 mL wash with AnEx Buffer B and subsequent 10 mL wash with AnEx Buffer A. Elution fractions of sufficient purity were pooled and concentrated to approx. 60 µM (SENP6) or 300 µM (SENP7), respectively, by spin filtration (10 kDa cut-off Amicon Ultra, Merck Millipore). The final NaCl concentration in the buffer was estimated to be approx. 200 mM. Aliquots were flash frozen in liquid nitrogen and stored at -80 °C until further use.

Isolated protein yields were 0.2 mg per litre of expression culture for SENP6(CD) and 0.5 mg per litre of expression culture for SENP7(CD).

2.2.5 Expression and purification of USPL1(CD)

The catalytic domain of USPL1 was expressed in *E. coli* Rosetta II(De3) and isolated via NiNTA affinity chromatography followed by anion exchange chromatography and subsequent SEC.

Chemically competent *E. coli* Rosetta II(De3) cells were transformed with the respective pET expression vector via heat shock, a starter culture was prepared in 50 mL 2xYT (Kan, Cam) and incubated over night at 37 °C, 200 rpm. The starter culture with OD600 1.0 was used to inoculate the expression culture (0.5 L 2xYT, Kan, Cam) to an OD600 of 0.03. The culture was incubated at 37 °C, 200 rpm, until OD600 reached 0.7, before protein expression was induced by the addition of IPTG to a final concentration of 0.5 mM. The expression culture was then incubated at 20 °C for 16 h and cells harvested (5000 xg, 4 °C, 30 min) with OD600 reaching 9.

The obtained pellet was resuspended in 30 mL USPL1 Lysis Buffer (20 mM Tris pH 8, 350 mM NaCl, 10 mM imidazole, 1 mM DTT) and subjected to pulsed sonication (Branson SFX 250 Sonifier, 6.5 mm tip, 24% amplitude, 7 min, 1 s on ↔ 2 s off). The crude lysate was cleared by centrifugation (14500 xg, 4 °C, 40 min), filtered through a syringe filter (PES membrane, 0.45 µm pore size) and H6-USPL1 was isolated via NiNTA affinity chromatography using a HisTrap HP 1 mL column (Cytiva). The column was first equilibrated with 10 mL USPL1 NiNTA Wash Buffer (20 mM Tris pH 8, 350 mM NaCl, 10 mM imidazole, 1 mM DTT), the sample was applied via the sample pump, the column was washed with 10 mL USPL1 NiNTA Wash Buffer, elution was performed with a linear gradient of 30 mL 0→100% USPL1 NiNTA Elution Buffer (20 mM Tris pH 8, 350 mM NaCl, 300 mM imidazole, 1 mM DTT) followed by a wash with 5 mL USPL1 NiNTA Elution Buffer and subsequent wash with 10 mL MQ H_2O. Elution fractions containing USPL1 were pooled, diluted 1:14 with USPL1 AnEx Buffer A (20 mM Tris pH 8, 3 mM DTT) and subjected to anion exchange chromatography using a ResourceQ 1 mL column (Cytiva). The column was first equilibrated with 10 mL USPL1 AnEx Buffer A, then the sample was applied via the sample pump. Elution was performed over a 45 mL gradient with 0→70% USPL1 AnEx Buffer B (20 mM Tris pH 8, 3 mM DTT, 1 M NaCl) followed by a wash with 5 mL USPL1 AnEx Buffer B and subsequent wash with 10 mL USPL1 AnEx Buffer A. Elution fractions containing USPL1 were pooled, concentrated by spin filtration (10 kDa cut-off Amicon Ultra, Merck Millipore) and further purified via SEC using a 10/300 Superdex 75 column (Cytiva) with USPL1 SEC Buffer (20 mM Tris pH 7.5, 100 mM NaCl, 2.5 mM DTT). Fractions of sufficient purity were pooled and concentrated to approx. 100 µM by spin filtration (10 kDa cut-off Amicon Ultra, Merck Millipore). Aliquots were flash frozen in liquid nitrogen and stored at -80 °C until further use.
The isolated protein yield was 4 mg per litre of expression culture, although it should be noted that this can most likely be increased by improving the purification strategy.

2.2.6 Expression and purification of Desi1 and SENP8

Full-length DeSi1 and SENP8 were expressed in *E. coli* BL21(De3) and isolated by NiNTA and reverse NiNTA affinity chromatography.
Chemically competent *E. coli* BL21(De3) cells were transformed with the respective pET expression vector via heat shock, a starter culture was prepared in 30 mL 2xYT (Kan) and incubated over night at 37 °C, 200 rpm. The starter culture with OD600 1.5-3 was used to inoculate the expression culture (0.5 L 2xYT, Kan) to an OD600 of 0.05. The expression culture was incubated at 37 °C, 200 rpm, until OD600 reached 0.6, before protein expression was induced by the addition of IPTG to a final concentration of 0.5 mM. The expression culture was then incubated at 30 °C for 3 h and cells were harvested (4000 xg, 4 °C, 20 min) with OD600 reaching 2.5-3.0.
The obtained pellet was resuspended in 35 mL SENP Lysis Buffer (20 mM Tris pH 8, 300 mM NaCl, 30 mM imidazole, 2 mM PMSF, DNaseI, 0.5 mM DTT) and subjected to pulsed sonication (Branson SFX 250 Sonifier, 6.5 mm tip, 24% amplitude, 7 min, 1 s on ↔ 2 s off). The crude lysate was cleared

by centrifugation (14500 xg, 4 °C, 30 min) and the obtained supernatant incubated with NiNTA resin (Ni Sepharose 6 Fast Flow, Cytiva; V_{slurry} = 500 µL = 2 CV) at 4 °C on a tube roller for 1 h. The suspension was transferred to a fritted plastic gravity flow column and the resin was washed three times with 20 CV SENP NiNTA Wash Buffer (20 mM Tris pH 8, 300 mM NaCl, 30 mM imidazole, 0.5 mM DTT) before H6-tagged SENP was eluted by sequential addition of four times 2 CV SENP NiNTA Elution Buffer (20 mM Tris pH 8, 300 mM NaCl, 300 mM imidazole, 0.5 mM DTT). Obtained elution fractions were pooled, diluted 1:19 with Storage Buffer (20 mM Tris pH 7.5, 100 mM NaCl, 2.5 mM DTT) and subjected to TEV cleavage by addition of H_6-tagged Tobacco Etch Virus protease H6-TEV (approx. 0.5 mol%). The TEV digest was incubated at 4 °C overnight. Finally, tag-less DeSi1 or SENP8 was recovered by reverse NiNTA purification by incubating the TEV digest with NiNTA beads at 4 °C on a tube roller for 1 h, transferring of the suspension to a fritted plastic gravity flow column and collecting of the flow-through. DeSi1 and SENP8 were concentrated to 600 µM and 150 µM, respectively, by spin filtration (10 kDa cut-off Amicon Ultra, Merck Millipore). Aliquots were flash frozen in liquid nitrogen and stored at -80 °C until further use.
Protein yields were 19 mg and 6 mg per litre of expression culture for DeSi1 and SENP8, respectively.

2.2.7 Expression and purification of Srt2A

Srt2A was expressed in *E. coli* BL21(De3) and isolated by NiNTA and optional reverse NiNTA affinity chromatography.
Chemically competent *E. coli* BL21(De3) cells were transformed with the respective pET expression vector via heat shock, a starter culture was prepared in 15 mL 2xYT (Kan) and incubated over night at 37 °C, 200 rpm. The dense starter culture was used to inoculate the expression culture (0.5 L 2xYT, Kan) to an OD600 of 0.025. The culture was incubated at 37 °C, 200 rpm, until OD600 reached 0.6-0.8, before protein expression was induced by the addition of IPTG to a final concentration of 0.5 mM. The expression culture was then incubated at 30 °C for 3.5 h and cells were harvested (4000 xg, 4 °C, 10 min) with OD600 typically reaching 3-4.
The cell pellet was resuspended in 30 mL Sortase Lysis Buffer (50 mM Tris pH 8, 300 mM NaCl, 1 mM $MgCl_2$, 0.5 mM DTT, 0.5 mM PMSF, DNaseI, cOmplete™ protease inhibitor (Roche, 1 tablet per 100 mL)) and subjected to pulsed sonication (Branson SFX 250 Sonifier, 6.5 mm tip, 24% amplitude, 5 min, 1 s on ↔ 2 s off). The crude lysate was cleared by centrifugation (14500 xg, 4 °C, 25 min) and the obtained supernatant incubated with NiNTA resin (Ni Sepharose 6 Fast Flow, Cytiva; V_{slurry} = 2 mL = 2 CV) at 4 °C on a tube roller for 1 h. The suspension was transferred to a fritted plastic gravity flow column and the resin was washed three times with 15 CV Sortase NiNTA Wash Buffer (20 mM Tris pH 8, 300 mM NaCl, 30 mM imidazole, 0.5 mM DTT) before H6-tagged Srt2A was eluted by sequential addition of four times 1 CV Sortase NiNTA Elution Buffer (20 mM Tris pH 8, 300 mM NaCl, 300 mM imidazole, 0.5 mM DTT).

For down-stream applications requiring H6-tagged Srt2A, the eluted protein was rebuffered to Sortase Buffer (50 mM Tris pH 7.5 (at 37 °C), 150 mM NaCl, 5 mM $CaCl_2$) and concentrated to 700-900 µM by spin filtration (10 kDa cut-off Amicon Ultra, Merck Millipore).
For optional removal of the H6-tag, the pooled NiNTA elution was dialyzed twice against 1 L of Dialysis Buffer (50 mM Tris pH 7.5, 150 mM NaCl, 5 mM $CaCl_2$, 2 mM DTT) at 4 °C and H6-TEV protease (0.5 mol%) was added into the dialysis tubing. Each dialysis step was performed for at least 2 h or overnight and completion of the TEV digest was confirmed by LC-MS. Tag-less Srt2A was recovered by reverse NiNTA affinity chromatography by incubating the TEV digest with NiNTA beads at 4 °C on a tube roller for 1 h, transferring of the suspension to a fritted plastic gravity flow column and collecting of the flow-through. The protein was rebuffered to Sortase Buffer (50 mM Tris pH 7.5 (at 37 °C), 150 mM NaCl, 5 mM $CaCl_2$) and concentrated to 700-1200 µM by spin filtration (10 kDa cut-off Amicon Ultra, Merck Millipore).
Aliquots were flash frozen in liquid nitrogen and stored at -80 °C until further use.
The yield of Srt2A ranged from 45 mg to 100 mg per litre of expression culture.

2.2.8 Expression and purification of C-terminal domain (CTD) of *Mb* and *Mm*AzGVAisoK-RS for X-ray crystallography

The catalytic domains *Mb*AzGVAisoK-RS and *Mm*AzGVAisoK-RS were expressed in *E. coli* and purified via NiNTA, reverse NiNTA and subsequent size exclusion chromatography.
Chemically competent *E. coli* Rosetta II(De3) cells were transformed with the respective pET expression vector via heat shock. After two hours of SOC rescue outgrowth at 37 °C, a starter culture was prepared in 50 mL 2xYT (Cam, Kan, 0.05% glucose) and incubated over night at 37 °C, 200 rpm. The starter culture was used to inoculate 4.5-6 L 2xYT (½ Cam, ½ Kan) to an OD600 of 0.015-0.05 and the expression culture was incubated at 37 °C, 200 rpm, until OD600 reached 0.6. Subsequently, the flask was incubated at 18 °C, 200 rpm, for 10 min before protein expression was induced by addition of IPTG to a final concentration of 0.75 mM and incubation at 18 °C, 200 rpm, was continued for 16-18 h. Cells were harvested (5000 xg, 4 °C, 35 min) with OD600 typically reaching 4.5-5 and pellets corresponding to 2 L expression cultures stored at -20 °C until further use.

E. coli cells (~10 g) were resuspended in 50 mL Buffer A (100 mM Tris-HCl pH 7.5, 500 mM NaCl, 20 mM imidazole, 10 mM β-mercaptoethanol) and disrupted using a Branson Digital Sonifier 250. The lysate was clarified by centrifugation (40,000 xg, 30 min, 4 °C), and the supernatant was applied to a 5 mL HisTrap HP column (GE Healthcare) pre-equilibrated with Buffer A and run on an ÄKTA Pure system at 5 mL/min. After washing with Buffer A containing 5% Buffer B (Buffer A supplemented with 500 mM imidazole) until a stable baseline at 280 nm was reached, the protein was eluted using a 5→100% linear gradient of Buffer B over 50 mL. Fractions containing *Mb*AzGVAisoK-RS or *Mm*AzGVAisoK-RS were pooled and incubated with 0.5 mg SUMO protease (Ulp1 from *S. cerevisiae*),

followed by overnight dialysis at 4 °C against 2 L Buffer C (20 mM Tris-HCl pH 7.5, 100 mM NaCl, 1 mM β-mercaptoethanol). The sample was reapplied to the HisTrap column, and the flow-through containing the cleaved protein was concentrated to ~2 mL using Amicon Ultra-15 devices. After removal of aggregates by centrifugation (20,000 × g, 10 min, 4 °C), the sample was injected to size-exclusion chromatography on a HiLoad Superdex 200 16/60 column equilibrated with Buffer D (Buffer C supplemented with 2 mM DTT and 5 mM $MgCl_2$) and run at 1.5 mL/min. Fractions containing pure protein were pooled, concentrated to >15 mg/mL, and used directly or flash-frozen for storage at -80 °C.

Crystallization trials for *Mb*AzGVAisoK-RS and *Mm*AzGVAisoK-RS were carried out by sitting-drop vapor diffusion at 4 °C. Protein samples (15-20 mg/mL in Buffer D) were mixed with reservoir solutions using an ARI Crystal Gryphon robot (Art Robbins Instruments). All crystallization setups contained final concentrations of 10 mM $MgCl_2$, 2 mM ATP, and 2 mM AzGVAisoK. *Mm*AzGVAisoK-RS crystallized from 0.1 M MES pH 6.5, supplemented with 30% (v/v) PEG 300, whereas *Mb*AzGVAisoK-RS formed crystals in 0.1 M MES pH 6.0, containing 10% (v/v) isopropanol. Crystals suitable for diffraction were typically obtained within several days. Prior to vitrification in liquid nitrogen, single crystals were transferred into reservoir solution supplemented with 20% (v/v) 2,3-butanediol for cryoprotection.

X-ray diffraction data were recorded at beamline P13 of PETRA III (DESY, Hamburg, Germany; proposal IV-2024-MX-970). Diffraction images were indexed, integrated, and scaled with XDS.[2] A summary of data collection and refinement statistics is provided in Table S1. Subsequent phasing, refinement, and validation steps were performed with programs from the CCP4 suite.[3] Resolution limits were selected based on the criteria $I/\sigma(I) > 2.0$, $R_{merge} < 80\%$ in the highest-resolution shell, and redundancy > 3.4. Molecular replacement was performed using the wild-type *Mm*PylRS structure (PDB ID: 2Q7E)[4] as the search model, which was also used as a template for *Mb*AzGVAisoK-RS. Model building and ligand placement were carried out in COOT,[5] followed by restrained refinement with REFMAC5.[6] Water molecules were added automatically using ARP/wARP.[7] Final cycles included TLS modeling, yielding acceptable R_{work}/R_{free} values and standard geometry (bond lengths and angles). Model quality was assessed with MOLPROBITY,[8] and all refined coordinates have been deposited in the Protein Data Bank (see Table S1).

Table S1: X-ray data collection and refinement statistics

	***Mm*AzGVAisoK-RS:ATP: AzGVAisoK**	***Mb*AzGVAisoK-RS:ATP**
**Crystal parameters**		
Space group	C2	$P2_1$
Cell constants	a=102.1<br>b=43.9 Å<br>c=72.8 Å<br>β=119.3	a=60.7<br>b=63.2 Å<br>c=81.6 Å<br>β=95.1

Subunits / AU[a]	1	2
**Data collection**		
Beam line	P13, DESY	P13, DESY
Wavelength (Å)	0.976	0.976
Resolution range (Å)[b]	30-1.25 (1.35-1.25)	30-1.55 (1.65-1.55)
No. observations	263779	297711
No. unique reflections[c]	76312	85191
Completeness (%)[b]	97.7 / 96.4	95.3 / 96.5
R_{merge} (%)[b, d]	7.9 / 55.1	5.4 / 77.6
I/σ (I)[b]	8.3 / 2.1	11.6 / 2.0
**Refinement (REFMAC5)**		
Resolution range (Å)	30-1.25	30-1.55
No. refl. working set	72492	80923
No. refl. test set	3815	4259
No. non hydrogen	2405	4526
No. of Mg^{2+}	3	6
No. of ligand atoms	53	62
Solvent	225	303
R_{work}/R_{free} (%)[e]	15.9 / 18.2	18.4 / 21.4
r.m.s.d. bond (Å) / (angle)[f]	0.008 / 1.4	0.003 / 1.0
Average B-factor ($Å^2$)	21.6	31.5
Ramachandran Plot (%)[g]	98.4 / 1.6 / 0	97.1 / 2.9 / 0
PDB accession code	**30XL**	**30XM**

[a] Asymmetric unit; dataset has been collected from a single crystal
[b] The values in parentheses for resolution range, completeness, R_{merge} and I/σ (I) correspond to the highest resolution shell
[c] Data reduction was carried out with XDS and from a single crystal. Friedel pairs were treated as identical reflections
[d] $R_{merge}(I) = \Sigma_{hkl}\Sigma_j \mid I(hkl)_j - \langle I(hkl)\rangle \mid / \Sigma_{hkl} \Sigma_j I(hkl)_j$, where $I(hkl)_j$ is the jth measurement of the intensity of reflection hkl and <I(hkl)> is the average intensity
[e] $R = \Sigma_{hkl} \mid |F_{obs}| - |F_{calc}| \mid / \Sigma_{hkl} |Fobs|$, where R_{free} is calculated without a sigma cut off for a randomly chosen 5% of reflections, which were not used for structure refinement, and R_{work} is calculated for the remaining reflections
[f] Deviations from ideal bond lengths/angles
[g] Percentage of residues in favoured region / allowed region / outlier region

2.2.9 Expression and purification of sfGFP(N150*)-H6, Ub(K11*)-H6, SUMO2(K11*)-H6 and PCNA(K164*) amber-suppressed with AzGVAisoK or BocK

For the expression of amber-suppressed proteins, *E. coli* K12 cells were co-transformed with the respective plasmids encoding an orthogonal ncAA-RS/tRNA pair and the POI with an amber stop codon at the desired site. Table S2 lists plasmid combinations and expression conditions for the expression of sfGFP(N150*)-H6, Ub(K11*)-H6, SUMO2(K11*)-H6 and PCNA(K164*) amber-suppressed with AzGVAisoK or BocK.

Table S2: Plasmids, ncAA concentrations and extrapolated yields for purified amber-suppressed proteins

**Protein**	**Plasmids**	**ncAA in expression medium**	**Yield [mg per L expression culture]**
sfGFP(N150BocK)-H6	pPylT sfGFP(N150TAG)-H6, *Mm*PylT (TetR)<br>pBK *Mb*PylRS (KanR)	2 mM BocK	81
sfGFP(N150GVAisoK)-H6	pPylT sfGFP(N150TAG)-H6, *Mm*PylT (TetR)<br>pBK *Mb*AzGVAisoK-RS (KanR)	4 mM AzGVAisoK	28
Ub(K11BocK)-H6	pBAD Ub(K11TAG)-H6 (AmpR)<br>pEVOL *Mb*PylRS, *Mm*PylT (CamR)	2 mM BocK	6.7
Ub(K11GVAisoK)-H6	pBAD Ub(K11TAG)-H6 (AmpR)<br>pEVOL *Mb*AzGVAisoK-RS, *Mm*PylT (CamR)	4 mM AzGVAisoK	1.8-2.6
SUMO2(K11BocK)-H6	pPylT SUMO2(K11TAG)-H6, *Mm*PylT (TetR)<br>pBK *Mb*PylRS (KanR)	2 mM BocK	2.2
SUMO2(K11GVAisoK)-H6	pPylT SUMO2(K11TAG)-H6, *Mm*PylT (TetR)<br>pBK *Mb*AzGVAisoK-RS (KanR)	4 mM AzGVAisoK	2
SUMO2(K11GVAisoK, ΔG)-H6	pBAD SUMO2(K11TAG, ΔG)-H6 (AmpR)<br>pEVOL *Mb*AzGVAisoK-RS, *Mm*PylT (CamR)	4 mM AzGVAisoK	1.1
PCNA(K164BocK)-H6	pBAD PCNA(K164TAG)-H6 (AmpR)<br>pEVOL *Mb*PylRS, *Mm*PylT (CamR)	2 mM BocK	20
PCNA(K164GVAisoK)-H6	pBAD PCNA(K164TAG)-H6 (AmpR)<br>pEVOL *Mb*AzGVAisoK-RS, *Mm*PylT (CamR)	4 mM AzGVAisoK	15

Precultures were prepared in 2xYT with the respective antibiotics and incubated overnight at 37 °C, 200 rpm. Dense starter cultures were diluted 1:500 in 14 aa AI medium (with the respective antibiotics) supplemented with the desired ncAA, and expression cultures were incubated at 37 °C, 200 rpm, for approximately 18-22 h.

For protein purification, cells were harvested (4000 xg, 4°C, 10 min) and the pellet was resuspended in NiNTA Lysis Buffer (20 mM Tris pH 8, 300 mM NaCl, 30 mM imidazole, 0.5 mM PMSF, DNaseI, cOmplete^{TM} protease inhibitor (Roche, 1 tablet per 100 mL)). Lysis was performed by pulsed sonication (Branson SFX 250 Sonifier, 6.5 mm tip, 24% amplitude, 4 min, 1 s on ↔ 2 s off) and the crude lysate cleared by centrifugation (14500 xg, 4 °C, 20 min). The obtained supernatant was incubated with NiNTA resin (Ni Sepharose 6 Fast Flow, Cytiva; $V_{slurry} = 1/1000 * V_{expression} = 2$ CV) at 4 °C on a tube roller for 1 h. Subsequently, the suspension was transferred to a fritted plastic gravity flow column, the resin was washed three times with 20 CV NiNTA Wash Buffer (20 mM Tris pH 8, 300 mM NaCl, 30 mM imidazole) followed by three times sequential elution with 2 CV NiNTA Elution Buffer (20 mM Tris pH 8, 300 mM NaCl, 300 mM imidazole). For AzGVAisoK-bearing proteins, the elution fraction was incubated with 1 mM TCEP at 4 °C for several hours or overnight until complete Staudinger reduction was confirmed by LC-MS. Proteins were rebuffered to Storage Buffer (20 mM Tris pH 7.5, 100 mM NaCl) by spin filtration (3 kDa cut-off or 10 kDa cut-off Amicon Ultra, Merck Millipore).

Aliquots were frozen in liquid nitrogen and stored at -80 °C until further use. Table S2 lists protein yields in mg per litre of expression culture for the purified amber-suppressed proteins.

2.2.10 GFP test expressions for determination of amber suppression efficiency promoted by PylRS variants

For the determination of amber suppression efficiency, *E. coli* K12 cells were co-transformed with the respective plasmids encoding an orthogonal ncAA-RS/tRNA pair and the POI with an amber stop codon at the desired site. Table S3 lists the investigated plasmid combinations and ncAAs for individual sfGFP(N150*)-H6 test expressions.

Table S3: Plasmid combinations and ncAA concentrations used for amber suppression efficiency test expressions

Reporter	Synthetase	ncAA
pPylT sfGFP(N150TAG)-H6, *Mm*PylT	pBK *Mb*AzGGisoK-RS	1, 2, or 4 mM AzGGisoK or AzGVAisoK
pPylT sfGFP(N150TAG)-H6, *Mm*PylT	pBK *Mb*PylRS L274A, N311Q, C313V	2 mM AzGGisoK or AzGVAisoK
pPylT sfGFP(N150TAG)-H6, *Mm*PylT	pBK *Mb*PylRS L274A, N311Q, C313V, M315Y	2 mM AzGGisoK or AzGVAisoK
pPylT sfGFP(N150TAG)-H6, *Mm*PylT	pBK *Mm*AzGGisoK-RS	2 mM AzGGisoK or AzGVAisoK
pPylT sfGFP(N150TAG)-H6, *Mm*PylT	pBK *Mm*AzGGisoK-RS (P188G)	2 mM AzGGisoK or AzGVAisoK
pBAD sfGFP(N150TAG)-H6	pEVOL *Mb*AzGGisoK-RS, *Mm*PylT	4 mM AzGGisoK or AzGVAisoK
pPylT sfGFP(N150TAG)-H6, *Ma*PylT	pBK *Ma*AzGGisoK-RS(IP)	4 mM AzGGisoK or AzGVAisoK
pBAD sfGFP(N150TAG)-H6	pEVOL *Ma*AzGGisoK-RS(IP)**,** *Ma*PylT	4 mM AzGGisoK or AzGVAisoK
pPylT sfGFP(N150TAG)-H6, *Mm*PylT	pBK *Mb*AzGVAisoK-RS	1, 2, or 4 mM AzGGisoK or AzGVAisoK
pPylT sfGFP(N150TAG)-H6, *Ma*PylT	pBK *Ma*AzGVAisoK-RS	2 mM AzGVAisoK
pPylT sfGFP(N150TAG)-H6, *Ma*PylT	pBK *Ma*AzGVAisoK-RS(IP)	2 mM AzGVAisoK

Starter cultures were prepared in 2xYT with the respective antibiotics, incubated overnight at 37 °C, 200 rpm, and then diluted 1:500 to expression media with the respective antibiotics. Expression cultures in 14 aa AI medium were incubated at 37 °C, 200 rpm, for 24 h. Expression cultures in 2xYT were manually induced when OD600 reached 0.6 by addition of arabinose to 0.05% (w/v) and then incubated at 37 °C, 200 rpm, for 16-20 h. Ratio of truncated versus full-length GFP in the expression culture was analysed by SDS-PAGE and in some cases additional Western blotting against H6-tag (αH6 antibody: αH6-HRP from mouse IgG_1, Roche 11965085001 purchased from Merck, working dilution 1:5000 in

1% skim milk in TBS-T) and served as indicator for amber suppression efficiency conferred by the different investigated orthogonal translation systems. Corresponding results are shown in Figure S13 and Figure S16.

2.3 SUMO1/2(XLA)-H6 (X = L or Q) processing assay with SENP1(CD) and SENP2(CD)

To investigate whether SUMO1(LLA)-H6, SUMO1(QLA)-H6, SUMO2(LLA)-H6, and SUMO2(QLA)-H6 are processed by SENP1(CD) and SENP2(CD) despite mutations in the C-terminal region of SUMO, an end-point processing assay was conducted. For this, 15 µM wtSUMO1-H6, SUMO1(LLA)-H6, SUMO1(QLA)-H6, wtSUMO2-H6, SUMO2(LLA)-H6, or SUMO2(QLA)-H6 were incubated -/+ 100 nM or 5 µM µM SENP1(CD) or SENP2(CD) in 20 mM Tris pH 7.5, 100 mM NaCl, 2 mM DTT at 37 °C for 20 h. Cleavage of the C-terminal H6-tag was monitored via SDS-PAGE (Self-prepared 17% SDS gels) with processed SUMO traveling further towards the anode than intact SUMO-H6.

Note that the investigated wt SUMO1/2-H6 and SUMO1/2(XLA)-H6 constructs were fusions of a C-terminal H6-tag to mature SUMO and did not include the native pre-SUMO sequences (SUMO1: HSTV, SUMO2: VY) between SUMO and the H6-tag. Corresponding results are shown in Figure S1.

2.4 In vitro Srt2A-mediated ligation

2.4.1 Test-scale Srt2A-mediated ligation

Srt2A-mediated ligation was performed in small scale to evaluate:

- SrtAssay I: Srt2A-mediated attachment of GPA, GVME, and GVDMA to SUMO2(LLA) (Figure S2)
- SrtAssays II: Comparison of SUMO1/2(LLA) and SUMO1/2(QLA) donors (Figure S5)
- SrtAssay III: SUMOylation of sfGFP(N150*), PCNA(K164*), and SUMO2(K11*) bearing GVAisoK or BocK (Figure S19)

<u>SrtAssay I:</u>

To evaluate GPA, GVME, and GVDMA for Srt2A-mediatd ligation, 100 µM SUMO2(LLA)-H6, 15 µM Srt2A (or 20 µM Srt2A in case of GVME) and 5 mM GPA, GVME, or GVDMA were incubated in 50 mM Tris pH 7.5, 150 mM NaCl, 5 mM $CaCl_2$ at 37 °C. The assays were set up in the following order: Buffer → SUMO2(LLA)-H6 → small molecule warhead → Srt2A. At denoted time points (GPA: 1', 15', 30', 45', 60', 180'; GVME: 1', 20', 30', 40', 60'; GVDMA: 1', 60', 150', 180') samples were prepared by diluting 3 µL of the reaction 1:9 in 0.1% formic acid. LC-MS analysis was conducted to determine the ratio of starting material SUMO2(LLA)-H6 (M calc: 11342 Da) and the obtained ABP SUMO2(LLA)-PA (M calc: 10499 Da), SUMO2(LLA)-VME (M calc: 10559 Da), or SUMO2(LLA)-VDMA (M calc: 10499 Da).

SrtAssay II:

To compare SUMO1 and SUMO2 donors with (LLA) and (QLA) mutations, 100 µM SUMO1(LLA)-H6, 100 µM SUMO1(QLA)-H6, 100 µM SUMO2(LLA)-H6, or 100 µM SUMO2(QLA)-H6 were incubated with 10 µM Srt2A and 1 mM GPA in 50 mM Tris pH 7.5, 150 mM NaCl, 5 mM $CaCl_2$ at 37 °C. The assays were set up in the following order: Buffer → SUMO1/2((Q/L)LA)-H6 → GPA → Srt2A. At denoted time points (1', 5', 10', 30', 90') samples were prepared by diluting 3 µL of the reaction 1:9 in 0.1% formic acid. LC-MS analysis was conducted to determine the ratio of starting materials SUMO1(LLA)-H6 (M calc: 11864 Da), SUMO1(QLA)-H6 (M calc: 11879 Da), SUMO2(LLA)-H6 (M calc: 11342 Da) and SUMO2(QLA)-H6 (M calc: 11357 Da), and the respective obtained ABPs SUMO1(LLA)-PA (M calc: 11021 Da), SUMO1(QLA)-PA (M calc: 11036 Da), SUMO2(LLA)-PA (M calc: 10499 Da) and SUMO2(QLA)-PA (M calc: 10514 Da).

SrtAssay III:

To investigate the sortylation of GVAisoK-bearing acceptor POIs and their respective BocK-bearing negative controls, 100 µM SUMO(QLA)-H6, 20 µM Srt2A and 20 µM POI(GVAisoK/BocK) were incubated in 50 mM Tris pH 7.5, 150 mM NaCl, 5 mM $CaCl_2$ at 37 °C. The assays were set up in the following order: Buffer → donor SUMO(QLA)-H6 → acceptor POI(GVAisoK/BocK) → Srt2A. At denoted time points (0 to up to 4 h), SDS samples were prepared by diluting 3 µL of the reaction with 8 µL of 4xSDS Laemmli sample buffer. SDS samples were boiled for 3 min at 95 °C, spun down (17000 xg, 5 min) and analyzed by subjecting 3.5 µL of sample to SDS-PAGE (Bolt 4-12% Bis-Tris Plus, Invitrogen).

2.4.2 Prep-scale Srt2A-mediated ligation

Preparative sortase-mediated ligation was performed to generate:

- SrtPrep I: SUMO2(LLA)-PA
- SrtPrep II: SUMO2(LLA)-VME
- SrtPrep III: SUMO2(LLA)-VDMA
- SrtPrep IV: HA-SUMO1(QLA)-PA
- SrtPrep V: HA-SUMO2(QLA)-PA
- SrtPrep VI: HA-SUMO1(QLA)-VAisoK
- SrtPrep VII: HA-SUMO2(QLA)-VaisoK
- SrtPrep VIII: HA-SUMO1(QLA)-PCNA(K164GVAisoK)-H6
- SrtPrep IX: HA-SUMO2 (QLA)-PCNA(K164GVAisoK)-H6
- SrtPrep X: HA-SUMO1(QLA)-SUMO2(K11GVAisoK, ΔG)-H6
- SrtPrep XI: HA-SUMO2(QLA)-SUMO2(K11GVAisoK, ΔG)-H6

SrtPrep I: SUMO2(LLA)-PA

To generate SUMO2(LLA)-PA, 100 µM SUMO2(LLA)-H6, 20 µM Srt2A and 5 mM GPA were incubated in Sortase Buffer (50 mM Tris pH 7.5, 150 mM NaCl, 5 mM $CaCl_2$) at 37 °C, 200 rpm, for 1 h. Then, phenyl vinyl sulfone (PVS) was added to 200 µM and the reaction incubated at rt for 10 min to inhibit Srt2A. Subsequently, the reaction was diluted 1:19 with SUMO CatEx Buffer A (50 mM NH_4Ac pH 4.5), filtered over a 0.45 µm PES membrane syringe filter and the product was isolated by cation exchange chromatography using a ResourceS 1 mL column (Cytiva). The column was first equilibrated with 10 CV SUMO CatEx Buffer A, then the sample was applied via the sample pump and the column subsequently washed with 10 CV SUMO CatEx Buffer A. Proteins were eluted over 40 CV with a linear gradient of 0→70% SUMO CatEx Buffer B (50 mM NH_4Ac pH 4.5, 1 M NaCl). The column was subsequently washed with 5 CV SUMO CatEx Buffer B followed by re-equilibration with 10 CV SUMO CatEx Buffer A. Elution fractions containing SUMO2(LLA)-PA were pooled, rebuffered to 25 mM Tris pH 7.8, 150 mM NaCl and concentrated to 210 µM by spin filtration (3 kDa cut-off Amicon Ultra, Merck Millipore). LC-MS analysis confirmed product identity (M calc: 10499 Da, obs: 10498 Da). Aliquots were flash-frozen in liquid nitrogen and stored at -80 °C until further use.

The reaction was typically set up with 2.5 mg SUMO2(LLA)-H6 educt and approx. 1 mg of product SUMO2(LLA)-PA was isolated.

SrtPrep II: SUMO2(LLA)-VME

To generate SUMO2(LLA)-VME, 100 µM SUMO2(LLA)-H6, 20 µM Srt2A and 5 mM GVME were incubated in Sortase Buffer (50 mM Tris pH 7.5, 150 mM NaCl, 5 mM $CaCl_2$) at 37 °C, 200 rpm, for 50 min. Then, PVS was added to 500 µM and the reaction incubated on ice for 15 min to inhibit Srt2A. Subsequently, the reaction was diluted 1:19 with SUMO CatEx Buffer A (50 mM NH_4Ac pH 4.5), filtered over a 0.45 µm PES membrane syringe filter and the product was isolated by cation exchange chromatography using a 5/50 MonoS 1 mL column (Cytiva). The column was first equilibrated with 10 CV SUMO CatEx Buffer A, then the sample was applied via the sample pump and the column subsequently washed with 15 CV SUMO CatEx Buffer A. Proteins were eluted over 50 CV with a linear gradient of 0→100% SUMO CatEx Buffer B (50 mM NH_4Ac pH 4.5, 1 M NaCl). The column was subsequently washed with 5 CV SUMO CatEx Buffer B followed by re-equilibration with 10 CV SUMO CatEx Buffer A. Elution fractions containing SUMO2(LLA)-VME were pooled, rebuffered to 25 mM Tris pH 7.8, 150 mM NaCl and concentrated to 560 µM by spin filtration (3 kDa cut-off Amicon Ultra, Merck Millipore). LC-MS analysis confirmed product identity (M calc: 10559 Da, obs: 10558 Da) with minor side product (M obs: 10594 Da, approx. 10%) in the final product. Aliquots were flash-frozen in liquid nitrogen and stored at -80 °C until further use.

The reaction was typically set up with 2.5 mg SUMO2(LLA)-H6 educt and approx. 1.2 mg of product SUMO2(LLA)-VME were isolated.

SrtPrep III: SUMO2(LLA)-VDMA

To generate SUMO2(LLA)-VDMA, 100 µM SUMO2(LLA)-H6, 15 µM Srt2A and 5 mM GVDMA were incubated in Sortase Buffer (50 mM Tris pH 7.5, 150 mM NaCl, 5 mM $CaCl_2$) at 37 °C, 200 rpm, for 1 h. Then, PVS was added to 500 µM and the reaction incubated at rt for 10 min to inhibit Srt2A. Subsequently, the reaction was diluted 1:24 with SUMO CatEx Buffer A (50 mM NH_4Ac pH 4.5), filtered over a 0.45 µm PES membrane syringe filter and the product was isolated by cation exchange chromatography using a ResourceS 1 mL column (Cytiva). The column was first equilibrated with 10 CV SUMO CatEx Buffer A, then the sample was applied via the sample pump and the column subsequently washed with 10 CV SUMO CatEx Buffer A. Proteins were eluted over 40 CV with a linear gradient of 0→70% SUMO CatEx Buffer B (50 mM NH_4Ac pH 4.5, 1 M NaCl). The column was subsequently washed with 5 CV SUMO CatEx Buffer B followed by re-equilibration with 10 CV SUMO CatEx Buffer A. Elution fractions containing SUMO2(LLA)-VDMA were pooled, rebuffered to 25 mM Tris pH 7.8, 150 mM NaCl and concentrated to 340 µM by spin filtration (3 kDa cut-off Amicon Ultra, Merck Millipore). LC-MS analysis confirmed product identity (M calc: 10572 Da, obs: 10571 Da). Aliquots were flash-frozen in liquid nitrogen and stored at -80 °C until further use.
The reaction was typically set up with 2.5 mg SUMO2(LLA)-H6 educt and approx. 1.3 mg of product SUMO2(LLA)-VDMA were isolated.

SrtPrep IV: HA-SUMO1(QLA)-PA

To generate HA-SUMO1(QLA)-PA, 100 µM HA-SUMO1(QLA)-H6, 10 µM Srt2A-H6 and 1 mM GPA were incubated in Sortase Buffer (50 mM Tris pH 7.5, 150 mM NaCl, 5 mM $CaCl_2$) at 37 °C, 200 rpm, for 45 min. Then, PVS was added to 200 µM and the reaction incubated at rt for 10 min to inhibit Srt2A. Subsequently, the reaction was diluted 1:1 with NiNTA Wash Buffer (20 mM Tris pH 8, 300 mM NaCl, 30 mM imidazole) and subjected to reverse NiNTA purification via spin columns (His SpinTrap, Cytiva). The flow-through was diluted 1:25 with SUMO AnEx Buffer A (20 mM Bis-Tris pH 6.5), filtered over a 0.45 µm PES membrane syringe filter and the product was isolated by anion exchange chromatography using a ResourceQ 1 mL column (Cytiva). The column was first equilibrated with 10 CV SUMO AnEx Buffer A, then the sample was applied via the sample pump and the column subsequently washed with 20 CV SUMO AnEx Buffer A. Proteins were eluted over 35 CV with a linear gradient of 0→80% SUMO AnEx Buffer B (20 mM Bis-Tris pH 6.5, 1 M NaCl). The column was subsequently washed with 5 CV SUMO AnEx Buffer B followed by re-equilibration with 10 CV SUMO AnEx Buffer A. Elution fractions containing HA-SUMO1(QLA)-PA were pooled, rebuffered to 20 mM Tris pH 7.5, 100 mM NaCl and concentrated to 390 µM by spin filtration (3 kDa cut-off Amicon Ultra, Merck Millipore). LC-MS analysis confirmed product identity (M calc: 12467 Da, obs: 12466 Da). Aliquots were flash-frozen in liquid nitrogen and stored at -80 °C until further use.
The reaction was typically set up with 1.2 mg HA-SUMO1(QLA)-H6 educt and approx. 0.8 mg of product HA-SUMO1(QLA)-PA were isolated.

SrtPrep V: HA-SUMO2(QLA)-PA

To generate HA-SUMO2(QLA)-PA, 100 µM HA-SUMO2(QLA)-H6, 10 µM Srt2A-H6 and 1 mM GPA were incubated in Sortase Buffer (50 mM Tris pH 7.5, 150 mM NaCl, 5 mM $CaCl_2$) at 37 °C, 200 rpm, for 45 min. Then, PVS was added to 200 µM and the reaction incubated at rt for 10 min to inhibit Srt2A. Subsequently, the reaction was diluted 1:1 with NiNTA Wash Buffer (20 mM Tris pH 8, 300 mM NaCl, 30 mM imidazole) and subjected to reverse NiNTA purification via spin columns (His SpinTrap, Cytiva). The flow-through was diluted 1:25 with SUMO CatEx Buffer A (50 mM NH_4Ac pH 4.5), filtered over a 0.45 µm PES membrane syringe filter and the product was isolated by cation exchange chromatography using a ResourceS 1 mL column (Cytiva). The column was first equilibrated with 10 CV SUMO CatEx Buffer A, then the sample was applied via the sample pump and the column subsequently washed with 20 CV SUMO CatEx Buffer A. Proteins were eluted over 35 CV with a linear gradient of 0→80% SUMO CatEx Buffer B (50 mM NH_4Ac pH 4.5, 1 M NaCl). The column was subsequently washed with 5 CV SUMO CatEx Buffer B followed by re-equilibration with 10 CV SUMO CatEx Buffer A. Elution fractions containing HA-SUMO2(QLA)-PA were pooled, rebuffered to 20 mM Tris pH 7.5, 100 mM NaCl and concentrated to 420 µM by spin filtration (3 kDa cut-off Amicon Ultra, Merck Millipore). LC-MS analysis confirmed product identity (M calc: 11944 Da, obs: 11944 Da). Aliquots were flash-frozen in liquid nitrogen and stored at -80 °C until further use.

The reaction was typically set up with 1.2 mg HA-SUMO2(QLA)-H6 educt and approx. 0.75 mg of product HA-SUMO2(QLA)-PA were isolated.

SrtPrep VI & VII: HA-SUMO1(QLA)-VAisoK and HA-SUMO2(QLA)-VAisoK

To generate HA-SUMO(QLA)-VAisoK, 100 µM HA-SUMO(QLA)-H6, 10 µM Srt2A-H6 and 1 mM GVAisoK were incubated in Sortase Buffer (50 mM Tris pH 7.5, 150 mM NaCl, 5 mM $CaCl_2$) at 37 °C, 200 rpm, for 45 min. Then, PVS was added to 200 µM and the reaction incubated at rt for 10 min to inhibit Srt2A. The reaction was then diluted 1:19 with SUMO AnEx Buffer A (20 mM Bis-Tris pH 6.5), filtered over a 0.45 µm PES membrane syringe filter and the product was isolated by anion exchange chromatography using a ResourceQ 1 mL column (Cytiva). The column was first equilibrated with 10 CV SUMO AnEx Buffer A, then the sample was applied via the sample pump and the column subsequently washed with 20 CV SUMO AnEx Buffer A. Proteins were eluted over 35 CV with a linear gradient of 0→80% SUMO AnEx Buffer B (20 mM Bis-Tris pH 6.5, 1 M NaCl). The column was subsequently washed with 5 CV SUMO AnEx Buffer B followed by re-equilibration with 10 CV SUMO AnEx Buffer A. Elution fractions containing HA-SUMO(QLA)-VAisoK were pooled, rebuffered to 20 mM Tris pH 7.5, 100 mM NaCl and concentrated to 710 µM for HA-SUMO1(QLA)-VAisoK or 790 µM HA-SUMO2(QLA)-VAisoK, respectively, by spin filtration (3 kDa cut-off Amicon Ultra, Merck Millipore). LC-MS analysis confirmed product identity (HA-SUMO1(QLA)-VAisoK M calc: 12641 Da, obs: 12642 Da; HA-SUMO2(QLA)-VAisoK M calc: 12118 Da, obs: 12119 Da). Aliquots were flash-frozen in liquid nitrogen and stored at -80 °C until further use.

For SUMO1, the reaction was typically set up with 2.5 mg HA-SUMO1(QLA)-H6 educt and approx. 1.2 mg of product HA-SUMO1(QLA)-GVAisoK were isolated. For SUMO2, the reaction was typically set up with 3 mg HA-SUMO2(QLA)-H6 educt and approx. 1.4 mg of product HA-SUMO2(QLA)-GVAisoK were isolated.

<u>SrtPrep VIII & IX: HA-SUMO(QLA)-PCNA(K164GVAisoK)-H6</u>

To generate HA-SUMO(QLA)-PCNA(K164GVAisoK)-H6, 100 µM HA-SUMO(QLA)-H6, 20 µM Srt2A and 20 µM PCNA(K164GVAisoK)-H6 were incubated in Sortase Buffer (50 mM Tris pH 7.5, 150 mM NaCl, 5 mM $CaCl_2$) at 37 °C, 200 rpm, for 2 h. PVS was added to 200 µM, the reaction was incubated for 10 min at rt and then diluted 1:5 with NiNTA Wash Buffer (20 mM Tris pH 8, 300 mM NaCl, 30 mM imidazole). To isolate H6-tagged proteins, equilibrated NiNTA resin was added, the suspension incubated at 4 °C for 1 h and then transferred to a fritted plastic gravity flow column. The resin was washed three times with NiNTA Wash Buffer before proteins were eluted with NiNTA Elution Buffer (20 mM Tris pH 8, 300 mM NaCl, 300 mM imidazole). The elution fraction was subsequently subjected to 10/300 Superdex75 SEC (20 mM Tris pH 7.5, 100 mM NaCl) and mixed trimers of SUMOylated and unmodified PCNA were concentrated by spin filtration (10 kDa cut-off Amicon Ultra, Merck Millipore). Concentrations were calculated with an estimated SUMOylation rate of 50% as judged by SDS-PAGE analysis and were approximated to 360 µM for HA-SUMO1(QLA)-PCNA(K164GVAisoK)-H6 and 170 µM for HA-SUMO2(QLA)-PCNA(K164GVAisoK)-H6, respectively. LC-MS analysis confirmed presence of free PCNA(K164GVAisoK)-H6 in both samples (M calc: 29731 Da, obs: 29733 Da and 29734 Da) in addition to the respective SUMOylated species (SUMO1-PCNA: M calc: 42086 Da, obs: 42086 Da; SUMO2-PCNA: M calc: 41563 Da, obs: 41563 Da). Aliquots were flash-frozen in liquid nitrogen and stored at -80 °C until further use.
For both ABPs, the reaction was set up with 1 mg of PCNA(K164GVAisoK)-H6 and approx. 0.6 mg of product were isolated.

<u>SrtPrep X & XI: HA-SUMO(QLA)-SUMO2(K11GVAisoK, ΔG)-H6</u>

To generate HA-SUMO(QLA)-SUMO2(K11GVAisoK, ΔG)-H6, 100 µM HA-SUMO(QLA)-H6, 20 µM Srt2A and 20 µM SUMO2(K11GVAisoK, ΔG)-H6 were incubated in Sortase Buffer (50 mM Tris pH 7.5, 150 mM NaCl, 5 mM $CaCl_2$) at 37 °C, 200 rpm, for 1 h. PVS was added to 200 µM, the reaction was incubated for 10 min at rt and then diluted 1:5 with NiNTA Wash Buffer (20 mM Tris pH 8, 300 mM NaCl, 30 mM imidazole). To isolate H6-tagged proteins, equilibrated NiNTA resin was added, the suspension incubated at 4 °C for 1 h and then transferred to a fritted plastic gravity flow column. The resin was washed three times with NiNTA Wash Buffer before proteins were eluted with NiNTA Elution Buffer (20 mM Tris pH 8, 300 mM NaCl, 300 mM imidazole). The elution fraction was subsequently subjected to 10/300 or 16/600 Superdex75 SEC (20 mM Tris pH 7.5, 100 mM NaCl) and isolated diSUMO was concentrated to 185 µM (SUMO1-SUMO2) or 290 µM (diSUMO2),

respectively, by spin filtration (10 kDa cut-off Amicon Ultra, Merck Millipore). LC-MS analysis confirmed product identity (HA-SUMO1(QLA)-SUMO2(K11GVAisoK, ΔG)-H6 M calc: 23738 Da, obs: 23737 Da; HA-SUMO2(QLA)-SUMO2(K11GVAisoK, ΔG)-H6 M calc: 23215 Da, obs: 23215 Da). Aliquots were flash-frozen in liquid nitrogen and stored at -80 °C until further use.
The reaction was typically set up with 3.5 mg SUMO2(K11GVAisoK, ΔG)-H6 and approx. 1.5-2 mg of GVAisoK-linked diSUMO were isolated.

2.5 Aminolysis of HA-SUMO(ΔG)-MESNa thioesters with propargylamine

HA-SUMO-PA probes were generated by subjecting HA-SUMO(ΔG)-MESNa thioesters to aminolysis with propargylamine. For this, HA-SUMO(ΔG)-MESNa was diluted to 1.5 mg/mL in 50 mM MOPS pH 6.8, 500 mM NaCl, 0.1 mM EDTA. Propargylamine was added from a 5 M stock solution to a final concentration of 300 mM. The pH of the reaction was assessed with pH paper and if needed adjusted to approximately 8 by addition of NaOH from a 100 mM stock solution. Then, the reaction was incubated at rt for 20 min with vigorous shaking (1400 rpm).
HA-SUMO1-PA was isolated via two subsequent PD-10 desalting columns (GE Healthcare), rebuffered (50 mM MOPS pH 6.8, 150 mM NaCl) and concentrated to 140 µM by spin filtration (3 kDa cut-off Amicon Ultra, Merck Millipore). LC-MS analysis confirmed product identity (M calc: 12412 Da, obs: 12411 Da) but also revealed a minor fraction of HA-SUMO1(ΔG)-MESNa thioester educt in the final sample (M calc: 12498 Da, 12.498 Da, approx. 15%).
HA-SUMO2-PA was isolated via cation exchange chromatography. For this, the reaction was diluted 1:175 with SUMO CatEx Buffer A (50 mM NH_4Ac pH 4.5), filtered and applied to a 5/50 MonoS 1 mL column (Cytiva). The column was equilibrated with 10 CV SUMO CatEx Buffer A, the sample was loaded via the sample pump, the column subsequently washed with 10 CV SUMO CatEx Buffer A. The proteins were eluted over 25 CV with a linear gradient of 0→60% SUMO CatEx Buffer B (50 mM NH_4Ac pH 4.5, 1 M NaCl). The column was subsequently washed with 5 CV SUMO CatEx Buffer B followed by re-equilibration with 5 CV SUMO CatEx Buffer A. Elution fractions containing HA-SUMO2-PA were pooled, rebuffered to 20 mM Tris pH 7.5, 100 mM NaCl and concentrated to 50 µM by spin filtration (3 kDa cut-off Amicon Ultra, Merck Millipore). LC-MS analysis confirmed product identity (M calc: 11888 Da, obs: 11887 Da).

2.6 SENP trapping assays

2.6.1 In vitro SENP trapping with catalytic domains of SENPs

In vitro SENP trapping assays with slightly different conditions were conducted to evaluate:

- ST-1: Comparative trapping of SENP2(CD) by SUMO2(LLA)-PA, -VME, -VDMA (Figure 2, Figure S4)
- ST-2: Trapping of SENP1(CD), SENP2(CD), SENP5(CD), SENP6(CD), SENP7(CD), USPL1(CD), DeSi1, SENP8 by SUMO(QLA)-PA versus SUMO-PA (Figure S7)
- ST-3: Trapping of SENP1(CD), SENP2(CD), SENP5(CD), SENP6(CD), SENP7(CD), USPL1(CD), DeSi1, SENP8 by GVAisoK-linked ABP as well as "no donor" and "no acceptor" controls (Figure 5, Figure S22, Figure S24)

<u>ST-1: Comparative trapping of SENP2(CD) by SUMO2(LLA)-PA, -VME, -VDMA</u>

Recombinantly purified SENP2(CD) or SENP2(CD, C548A) (15 µM) was incubated with SUMO2(LLA)-PA, SUMO2(LLA)-VME, SUMO2(LLA)-VDMA, or SUMO2-H6 (50 µM) in 50 mM Tris pH 7.5, 150 mM NaCl, 0.5 mM TCEP at 37 °C, 200 rpm. Covalent complex formation or processing of the C-terminal H6-tag was monitored via SDS-PAGE analysis. For this, samples (3 µL of assay + 1 µL of 4x SDS Laemmli Buffer) were taken at t = 0, 10 min, 30 min, 1 h, and 3 h, boiled for 5 min at 95 °C and subjected to SDS-PAGE gels (self-prepared gels, 15%).

<u>ST-2: Trapping of SENP1(CD), SENP2(CD), SENP5(CD), SENP6(CD), SENP7(CD), USPL1(CD), DeSi1, SENP8 by SUMO(QLA)-PA versus SUMO-PA</u>

SENPs and SUMO-PA probes were mixed equimolarly (both 15 µM) in 20 mM Tris pH 7.5, 100 mM NaCl, 0.5 mM TCEP and incubated at 37 °C for 1 h. The fraction of formed complex was monitored at t = 0 and 1 h by SDS-PAGE analysis. For this, samples (3 µL of assay + 8 µL of 4x SDS Laemmli Buffer) were taken and boiled at 95 °C for 4 min, before 4 µL were subjected to SDS-PAGE gels (Bolt 4-12% Bis-Tris Plus gels).

<u>ST-3: Trapping of SENP1(CD), SENP2(CD), SENP5(CD), SENP6(CD), SENP7(CD), USPL1(CD), DeSi1, SENP8 with GVAisoK-linked ABP as well as "no donor" and "no acceptor" controls</u>

SENPs and GVAisoK-containing probes were mixed equimolarly (both 15 µM) in 20 mM Tris pH 7.5, 100 mM NaCl, 0.5 mM TCEP and incubated at 37 °C for 1 h (for SUMO ABPs) or 3 h (for PCNA ABPs). Covalent complex formation was monitored by taking samples (3 µL of assay + 8 µL of 4x SDS Laemmli buffer) at t = 0 and 1 h or 3 h, boiling at 95 °C for 4 min and subjecting 4 µL to SDS-PAGE gels (Bolt 4-12% Bis-Tris Plus gels).

2.6.2 SENP trapping in cell lysate with assembled ABPs

Purified assembled ABPs were added to different lysates to show trapping in complex environments. For this we investigated:

- STL-1: HA-SUMO2(QLA)-PA + HEK293T lysate (overexpression FLAG-SENP1) (Figure 2, Figure S9)
- STL-2: HA-SUMO2(QLA)-PA + lysate of untransfected HEK293T or MCF-7 cells (Figure 2, Figure S10)
- STL-3: HA-SUMO2(QLA)-PCNA(K164GVAisoK)-H6 + HEK293T lysate (overexpression FLAG-SENP1) (Figure S23)

STL-1: HA-SUMO2(QLA)-PA + HEK293T lysate (overexpression FLAG-SENP1)

Approximately 80% confluent HEK293T cells were transiently transfected with pIRES FLAG-SENP1(CD), pIRES FLAG-SENP1(CD, C603A), pFLAG-CMV SENP1(FL), or pFLAG-CMV SENP1(FL, C603A) (10 µg per 10 cm dish). Cells were cultured for 40 h and then washed twice with 10 mL PBS. Cells were harvested by gently rinsing them off the plate with cold PBS followed by centrifugation (400 xg, 4 °C, 5 min). The supernatant was discarded and the pellet resuspended in 300 µL Lysis Buffer (50 mM Tris pH 7.5, 150 mM NaCl, 0.5% Triton-X-100 (w/v), 1x cOmplete™ Protease inhibitor (Roche)) followed by pulsed probe sonication (Branson SFX 250 Sonifier, µ-tip, 12% amplitude, 30 s, 1 s on ↔ 2 s off). Lysates were cleared by centrifugation (17000 xg, 4 °C, 20 min) after which the total protein concentration was determined by BCA assay (Pierce Rapid Gold BCA Protein Assay Kit, Thermo Fisher Scientific). Trapping assays were set up with 4 mg/mL cell lysate and 5 µM of HA-SUMO2(QLA)-PA by dilution in 20 mM Tris pH 7.5, 100 mM NaCl, and incubated at 37 °C. Samples (5 µL of assay + 4 µL of 4x SDS Laemmli Buffer) were taken at t = 0, 30 min, 3 h, boiled at 95 °C for 3 min and subjected to SDS-PAGE gels, (Bolt 4-12% Bis-Tris Plus gels were used for SENP1(CD) samples, NuPAGE 3-8% Tris-Acetate gels were used for SENP1(FL) samples). The amount of total protein loaded per lane was 20 µg. After electrophoresis, proteins were transferred onto nitrocellulose membranes using the BioRad TurboBlot system (high MW method) and the membranes subsequently blocked with 5% (w/v) skim milk in TBS-T at rt for 1 h. SENP and ABP were detected by Western blot αFLAG (mouse αFLAG-HRP A8592, Sigma Aldrich, 1:10000) and αHA (rat αHA-HRP clone 3F10, Roche, 1:10000). For this, the membranes were incubated with the HRP-coupled antibodies (1:10000) in 5% (w/v) skim milk in TBS-T at 4 °C overnight, washed three times 10 min with TBS-T, and then treated with Immobilon® Forte Western HRP Substrate, after which chemiluminescence was recorded with an Amersham™ ImageQuant™ 800 Western blot imaging system.

STL-2: HA-SUMO2(QLA)-PA + lysate of untransfected HEK293T or MCF-7 cells

HEK293T and MCF-7 cells were cultured in 10 cm dishes to a confluency of approx. 90%. Cells were then washed twice with 10 mL PBS, harvested in PBS (400 xg, 4 °C, 5 min), and the cell pellet resuspended in 300 µL Lysis Buffer (50 mM Tris pH 7.5, 150 mM NaCl, 0.5% Triton-X-100 (w/v), 1x cOmplete™ Protease inhibitor (Roche)) followed by pulsed probe sonication (Branson SFX 250 Sonifier, µ-tip, 12% amplitude, 30 s, 1 s on ↔ 2 s off). Lysates were cleared by centrifugation

(17000 xg, 4 °C, 20 min) after which the total protein concentration was determined by BCA assay (Pierce Rapid Gold BCA Protein Assay Kit, Thermo Fisher Scientific). Trapping assays were set up with 5 mg/mL cell lysate (HEK293T) or 2 mg/mL (MCF-7) -/+ 5 µM of HA-SUMO2(QLA)-PA by dilution in 20 mM Tris pH 7.5, 100 mM NaCl, and incubated at 37 °C. Samples (6 µL of assay + 5 µL of 4x SDS Laemmli buffer for HEK293T assay; 15 µL of assay + 6 µL 4x SDS Laemmli buffer for MCF-7 assay) were taken at t = 0, 3 h, and 18 h, boiled at 95 °C for 5 min, and subjected to SDS-PAGE gels (NuPAGE 3-8% Tris-Acetate gels). The amount of total protein loaded per lane was 30 µg. After electrophoresis, proteins were transferred onto nitrocellulose membranes using the BioRad TurboBlot system (high MW method) and the membranes subsequently blocked with 5% (w/v) skim milk in TBS-T at rt for 30 min. Endogenous SENP1 was detected by Western blot αSENP1 (rabbit αSENP1 EPR3844, Abcam ab108981, 1:1000). For this the membranes were incubated with primary αSENP1 antibody (1:1000) in 5% (w/v) skim milk in TBS-T at 4 °C overnight and washed three times 10 min with TBS-T prior to incubation with secondary αRabbit-HRP antibody (mouse αRabbit-HRP sc-2357, Santa Cruz Biotechnology) (1:10000) in 1% (w/v) skim milk in TBS-T at rt for 1 h. Subsequently, membranes were washed three times 10 min with TBS-T, treated with Immobilon® Forte Western HRP Substrate and chemiluminescence was recorded with an Amersham™ ImageQuant™ 800 Western blot imaging system.

STL-3: HA-SUMO2(QLA)-PCNA(K164GVAisoK)-H6 + HEK293T lysate (overexpression FLAG-SENP1)

The experiment was conducted following procedure STL-1, with the only exception that instead of the HA-SUMO(QLA)-PA probe, 5 µM HA-SUMO2(QLA)-PCNA(K164GVAisoK)-H6 were used.

2.6.3 Concerted sortase reaction and SENP trapping

Concerted sortase-mediated generation of monoSUMO probes and trapping of SENPs by the in situ generated ABPs were investigated in different environments:

- CSST-1: One-pot generation and application of SUMO2(LLA)-PA for SENP2(CD) trapping in vitro (Figure S11a)
- CSST-2: One-pot generation and application of SUMO2(LLA)-PA, -VME, and -VDMA for SENP2(CD) trapping cell lysates (Figure 2e, Figure S11b)

CSST-1: One-pot generation and application of SUMO2(LLA)-PA for SENP2(CD) trapping in vitro

For the in vitro one-pot assay with purified proteins, 100 µM SUMO2(LLA)-H6, 20 µM Srt2A, 20 µM SENP2(CD) and 5 mM GPA (or 5 mM tri-glycine as a control) were incubated in 50 mM Tris pH 7.5, 150 mM NaCl, 5 mM $CaCl_2$, 1 mM DTT, at 37 °C, 200 rpm. To monitor covalent complex formation, samples (1 µL of assay, 3 µL of 4x SDS Laemmli Buffer) were taken at t = 0, 10 min, 30 min, 1 h, and

4 h, boiled at 95 °C for 4 min and subjected to SDS-PAGE gels (self-prepared gels, 15%). Corresponding data is shown in Figure S11a.

CSST-2: One-pot generation and application of SUMO2(LLA)-PA, -VME, and -VDMA for SENP2(CD) trapping cell lysates

HEK293T cells were cultured in 10 cm dishes to a confluency of approx. 70 % and transiently transfected with 8 µg of pIRES FLAG-SENP2(CD) per dish. Approx. 40 h after transfection, cells were washed twice with PBS and harvested by gently rinsing them off the plate with PBS followed by centrifugation (300 xg, 5 min). The cell pellet was resuspended in 500 µL Lysis Buffer (50 mM Tris pH 7.5, 150 mM NaCl, 5 mM $CaCl_2$, 0.5 mM TCEP) and lysed by five freeze-thaw cycles (rt ↔ liquid nitrogen). The lysate was cleared by centrifugation (17000 xg, 4 °C, 20 min) and assays were set up with 100 µL of lysate supplemented with 100 µM recombinant SUMO2(LLA)-H6, 20 µM Srt2A, -/+ 5 mM GPA, GVME, or GVDMA. The assays were incubated at 37 °C, 200 rpm. Samples (15 µL of assay + 5 µL of 4x SDS Laemmli Buffer) were taken at t = 0 and 3 h, boiled at 95 °C for 8 min and subjected to SDS-PAGE gels (Bolt 4-12% Bis-Tris Plus gels). After electrophoresis, proteins were transferred to an iBlot nitrocellulose membrane using an iBlot2 transfer system (7 min, 20 V; Thermo Fisher Scientific). The membrane was blocked with 5% (w/v) skim milk in TBS-T at rt for 1 h, incubated with primary αFLAG antibody (rabbit αFLAG F7425, Merck Millipore) (1:5000) in 1% (w/v) skim milk in TBS-T at rt for 1 h, washed five times 5 min with TBS-T, incubated with αRabbit-HRP antibody (mouse αRabbit-HRP sc-2357, Santa Cruz Biotechnology) (1:10000) in 1% (w/v) skim milk in TBS-T at rt for 1 h, and washed again five times 5 min with TBS-T. The membrane was then treated with Immobilon® Forte Western HRP Substrate and chemiluminescence was recorded. Corresponding data is shown in Figure 2e and Figure S11b.

2.7 In cellulo SENP trapping

2.7.1 In cellulo SENP trapping assay

HEK293T cells were cultured to approx. 70% confluency in 12-well plates and transiently transfected with 1 µg of pIRES HA-SUMO2(QLA)^x-T2AP2A-Srt2A-Myc and 1 µg of pIRES FLAG-SENP1(CD), pIRES FLAG-SENP1(CD, C603A), pFLAG-CMV SENP1(FL), or pFLAG-CMV SENP1(FL, C603A). Treatment with 0.5 mM GPA was started 40 h post transfection and applied for 2 h, before cells were washed twice with 1 mL PBS and harvested by resuspension in 1 mL PBS followed by centrifugation (300 xg, 4 °C, 5 min). Cell pellets were resuspended in RIPA Lysis Buffer (50 mM Tris pH 7.5, 150 mM NaCl, 1 mM EDTA, 1% (w/v) NP40, 0.25% (w/v) sodium deoxycholate, 1x cOmplete™ Protease inhibitor(Roche); 55 µL buffer per pellet corresponding to one well of the 12-well plate), incubated on ice for 30 min and vortexed every 10 min. Lysates were cleared by centrifugation (17000 xg, 4 °C, 20 min), total protein concentration was determined by BCA assay (Pierce Rapid Gold BCA Protein Assay Kit, Thermo Fisher Scientific) and normalized to 3 mg/mL with RIPA Lysis Buffer. Samples

(50 µL lysate + 20 µL 4x SDS Laemmli buffer) were boiled at 95 °C for 10 min and 10 µL of sample were subjected to SDS-PAGE gels (Bolt 4-12% Bis-Tris Plus gels were used for SENP1(CD) samples, NuPAGE 3-8% Tris-Acetate gels were used for SENP1(FL) samples). Total protein loaded per lane was 21 µg. After electrophoresis, proteins were transferred onto nitrocellulose membranes using the BioRad TurboBlot system (high MW method) and the membranes subsequently blocked with 5% (w/v) skim milk in TBS-T at rt for 1 h. SENP1 was monitored by Western blot αFLAG (mouse αFLAG-HRP A8592, Simga Aldrich, 1:10000). For this, the membranes were incubated with the HRP-coupled antibody (1:10000) in 5% (w/v) skim milk in TBS-T at 4 °C overnight, washed three times 10 min with TBS-T, and then treated with Immobilon® Forte Western HRP Substrate, after which chemiluminescence was recorded with an Amersham™ ImageQuant™ 800 Western blot imaging system. Corresponding data is shown in Figure 2f and Figure S12b-c.

2.7.2 Cell viability assay

HEK293T cells were cultured in 96-well plates (Cell Culture Microplate, PS, F-bottom (chimney well), µCLEAR®, black, CELLSTAR®, Greiner) to approx. 80% confluency. To investigate potential toxicity of GPA treatment, cells were treated with 0.1 mM GPA, 0.5 mM GPA or 1 mM GPA in the culture medium. For controls, cells were either left untreated or treated with 0.1% Triton-X-100 in the culture medium. Furthermore, additional wells without cells containing just the culture medium supplemented with 0.5 mM GPA were included. Cells were cultured for 2 h at 37 °C (5% CO_2), before 20 µL of CellTiter-Blue® Cell Viability Assay (Promega) reagent were added to the 100 µL of culture medium. The plate was then gently tilted a few times to ensure proper distribution and further incubated at 37 °C (5% CO_2) for 3.5 h. Fluorescence (excitation: 560 nm, emission: 590 nm) was measured using a Varioskan LUX platereader (Thermo Fisher Scientific). Three wells were set up for every condition. Corresponding data is shown in Figure S12d.

2.8 Synthetase screening and selection campaign

2.8.1 Synthetase screen for AzGVAisoK incorporation by previously published PylRS variants

E. coli K12 cells harbouring a pBK PylRS (KanR or AmpR) variant plasmid and a pPylT plasmid encoding an sfGFP(N150TAG)-H6 reporter and a suppressor tRNA PylT (TetR) were picked from cryo-stocks to inoculate 1 mL 2xYT (Kan or Amp, Tet) in 96-well deep-well plates. Starter cultures were grown overnight at 37 °C, 200 rpm, and used to inoculate 500 µL 14 aa AI medium (Kan or Amp, Tet) 1:500 in 96-well deep-well plates. Each starter culture was used to inoculate two expression cultures, one of which was supplemented with 2 mM AzGVAisoK. Expression cultures were incubated at 37 °C, 200 rpm, for 48 h. To determine ncAA-dependent GFP fluorescence, 100 µL of culture and 100 µL of PBS were transferred to clear-bottom 96-well plate followed by measuring GFP fluorescence (Excitation: 480 nm, Emission: 510 nm) normalized to absorbance at 600 nm. Amber suppression

efficiency was calculated by dividing normalized GFP fluorescence in presence of AzGVAisoK by normalized GFP fluorescence in absence of AzGVAisoK. Corresponding data is shown in Figure S14.

2.8.2 Selection of *Mb*AzGVAisoK-RS from *Mb*PylRS library

For the selection of a synthetase for AzGVAisoK incorporation from a previously published *Mb*PylRS library,[9] the library was transformed into *E. coli* DH10β cells harbouring suitable selection reporter plasmids and plating them on positive or negative selection plates.

For the first round of positive selection, chemically competent *E. coli* DH10β cells were transformed with the single positive reporter plasmid pPylT CAT(111TAG) via heat shock and after rescue and outgrowth in SOC medium, an overnight culture was prepared in 10 mL 2xYT (Tet). For the preparation of electrocompetent reporter cells, the dense starter culture was used to inoculate 200 mL LB medium (½ Tet) to an OD600 of 0.01. The culture was incubated at 37 °C, 200 rpm, until OD600 reached 0.35 and subsequently cooled in an ice-water bath for 30 min. Cells were harvested (3000 xg, 4 °C, 8 min), the supernatant discarded and the pellets resuspended in 200 mL ice-cold autoclaved MQ water by gentle swirling of the tubes in an ice-water bath. The cells were then harvested again (3000 xg, 4 °C, 8 min), resuspended in 100 mL ice-cold autoclaved MQ water, harvested (3000 xg, 4 °C, 8 min), resuspended in 50 mL ice-cold autoclaved MQ water, harvested (3000 xg, 4 °C, 8 min), and resuspended in a final volume of 400 µL ice-cold autoclaved MQ water. The electrocompetent cells with OD600 of approx. 75-100 were used immediately after preparation.

To transform the *Mb*PylRS library into the electrocompetent reporter cells with a transformation efficiency of over $2*10^9$ cfu/µg, 200 µL of cells and 1 µg of plasmid library were transferred to a pre-cooled electroporation cuvette (0.2 cm gap, EP202 Cell Projects). The cuvette was incubated on ice for 15 min before electroporation was conducted with an ECM 399 Electroporation System (BTX) at 2.5 kV with time constant reaching 5.0-5.2 ms. Immediately after electroporation, 900 µL of pre-warmed SOC medium were added to the cuvette and cells were transferred to an Eppendorf tube for subsequent incubation at 37 °C for 1 h, before the rescued cells were added to 200 mL pre-warmed 2xYT (Kan, Tet). The culture was incubated until OD600 reached 0.6, then 200 µL were plated on 15 cm positive selection plates (14 aa AI agar, 1x Tet, 1x Kan, 1.2x Cam) without ncAA or supplemented with 1 mM AzGVAisoK or 1 mM AzGGisoK that was used as a control.

Positive selection plates were incubated at 37° C for approx. 20 h, then 10 mL 2xYT medium were added and the colonies were gently scraped off the plate using a Drigalski spatula. The suspension was transferred to an Erlenmeyer flask and 10 mL 2xYT as well as Kan and Tet (at regular working concentration) were added. The culture was incubated at 37 °C, 200 rpm, for 3 h before cells were harvested and plasmid DNA was isolated using a MidiPrep kit (Zymo). To separate the selected PylRS sub-library and the reporter plasmid, 50 µg of MidiPrepped DNA were subjected to agarose gel electrophoresis. The desired band was excised and purified with a Gel extraction kit (GeneJET Gel Extraction Kit, Thermo Fisher Scientific). To increase DNA purity, the eluted plasmids were subjected

to an additional round of purification with a PCR purification kit (GeneJET PCR Purification Kit, Thermo Fisher Scientific). Final concentrations for the obtained sub-libraries after positive selection step 1 averaged 200 ng/µL.

For the optional negative selection step, chemically competent *E. coli* DH10β cells were transformed with the negative reporter plasmid pYOBB barnase(3TAG, 45TAG) (CamR) and electrocompetent cells were prepared as described above. Electrocompetent negative reporter cells were transformed with 200 ng of the positive selection 1 sub-library by electroporation as described above. Transformed cells were rescued in 2 mL pre-warmed SOB medium at 37 °C, 200 rpm, for 1 h before they were transferred to 200 mL 2xYT (Kan, Cam) and incubated at 37 °C, 200 rpm. When OD600 reached 0.6, 150 µL of culture were plated on 15 cm negative selection plates (LB agar, 1.5 mM phenylalanine, 0.2% arabinose w/v, 1x Kan, 1x Cam). Negative selection plates were incubated at 37 °C for 48 h before the selected positive-negative sub-library was isolated as described above.

For the second round of positive selection, chemically competent *E. coli* DH10β cells were transformed with the pPylT CAT(111TAG), sfGFP(N150TAG)-H6 dual reporter plasmid and electrocompetent cells were prepared as described above. The second round of positive selection was performed with the sub-libraries obtained from positive selection 1 as well as sub-libraries obtained from positive-negative selection. For this, 250 ng of sub-library were transformed into freshly prepared dual positive reporter cells by electroporation, cells were rescued with 2 mL pre-warmed SOC medium and then transferred to 200 mL pre-warmed 2xYT (Kan, Tet). The culture was incubated at 37 °C, 200 rpm, until OD600 reached 0.6. Then, 150 µL were plated on 15 cm positive selection round 2 plates (14 aa AI agar, 1x Tet, 1x Kan, 1.2x Cam) without ncAA or supplemented with 2 mM AzGVAisoK or 2 mM AzGGisoK.

To evaluate amber suppression efficiency promoted by PylRS variants enriched over the course of the selection campaign, single clones were picked from positive selection round two plates into 750 µL 2xYT (Kan, Tet) in 96-well deep well plates and incubated at 37 °C, 200 rpm, overnight. These starter cultures were used to inoculate 500 µL 14 aa AI medium (Kan, Tet, -/+ 3 mM of AzGVAisoK or AzGGisoK) 1:1000. Expression cultures were incubated at 37 °C, 200 rpm, for 48 h before ncAA-dependent GFP fluorescence was determined as described above. PylRS variants from wells showing the highest ncAA-dependent GFP fluorescence were isolated by MiniPrep (NucleoSpin Plasmid Mini Kit, Machery Nagel) and analyzed by Sanger sequencing. Corresponding results are shown in Figure S15.

2. 9 Chemical synthesis

2.9.1 Synthesis of GPA

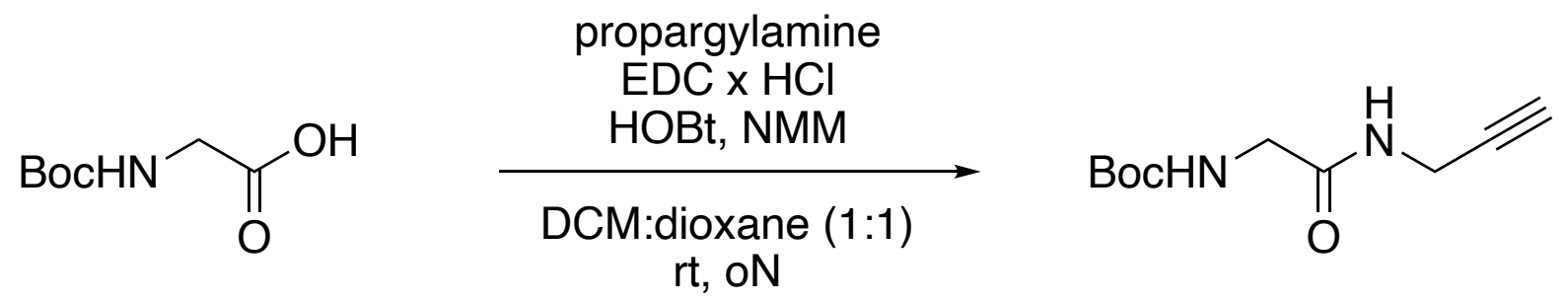

***tert*-butyl (2-oxo-2-(prop-2-yn-1-ylamino)ethyl)carbamate [Boc-GPA].** Propargylamine (1.16 mL, 18.2 mmol, 1 eq) was added to the stirring solution of Boc-Gly-OH (3.50 g, 20.0 mmol, 1.1 eq), EDC × HCl (3.83 g, 20.0 mmol, 1.1 eq), HOBt (2.70 g, 20.0 mmol, 1.1 eq) and *N*-methylmorpholine (2.18 mL, 36.4 mmol, 2 eq) in 20 mL of dry DCM:dioxane (1:1, v/v) and the mixture was stirred overnight at rt. The solvent was removed under reduced pressure, the obtained residue re-dissolved in DCM and sequentially washed with aqueous 10% HCl solution (twice), saturated $NaHCO_3$ solution, and brine. The organic phase was dried over Na_2SO_4, filtered and concentrated in vacuo. Purification by column chromatography over silica (2% MeOH/DCM, v/v) afforded **Boc-GPA** (1.87 g, 48%) as a white crystalline solid.

^{1}H NMR (300 MHz, $CDCl_3$) δ[ppm] = 6.71 (bs, 1H), 5.33 (bs, 1H), 4.05 (dd, *J* = 5.3, 2.5 Hz, 2H), 3.81 (bs, 2H), 2.22 (t, *J* = 2.6 Hz, 1H), 1.44 (s, 9H).
^{13}C NMR (75 MHz, $CDCl_3$) δ[ppm] = 169.4, 156.3, 80.6, 79.4, 71.8, 44.5, 29.2, 28.4.

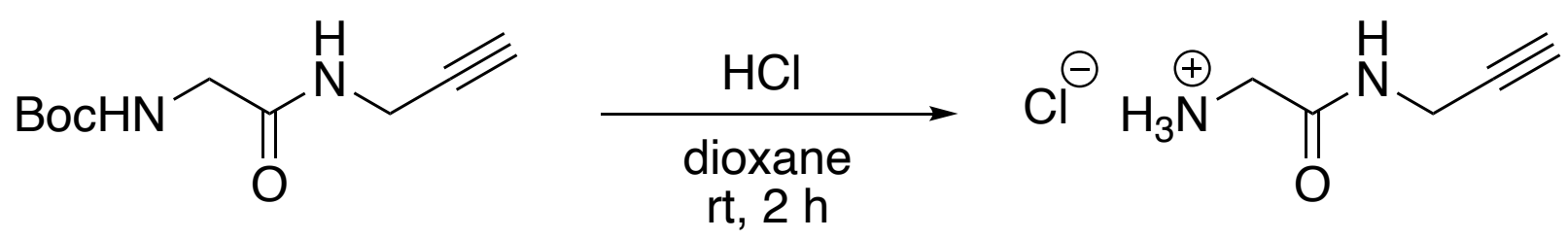

2-amino-*N*-(prop-2-yn-1-yl)acetamide hydrochloride [GPA]. Boc-GPA (1.87 g, 8.82 mmol) was dissolved in 30 mL of 1,4-dioxane and 30 mL of 4 M HCl in 1,4-dioxane were carefully added to the solution. After stirring at rt for 2 hours (the reaction was judged by TLC in 5 % MeOH/DCM as complete), the reaction mixture was concentrated under reduced pressure. The residue was re-dissolved in water, extracted a with Et_2O and the aqueous phase was lyophilised to afford the hydrochloride salt of **GPA** (1.26 g, 96 %) as a white solid.

^{1}H NMR (300 MHz, DMSO-d_6) δ[ppm] = 9.00 (t, *J* = 5.3 Hz, 1H), 8.29 (bs, 3H), 3.92 (dd, *J* = 5.5, 2.5 Hz, 2H), 3.53 (s, 2H), 3.18 (t, *J* = 2.5 Hz, 1H).
^{13}C NMR (75 MHz, DMSO-d_6) δ[ppm] = 165.7, 80.4, 73.6, 40.0, 28.0.

2.9.2 Synthesis of GVME

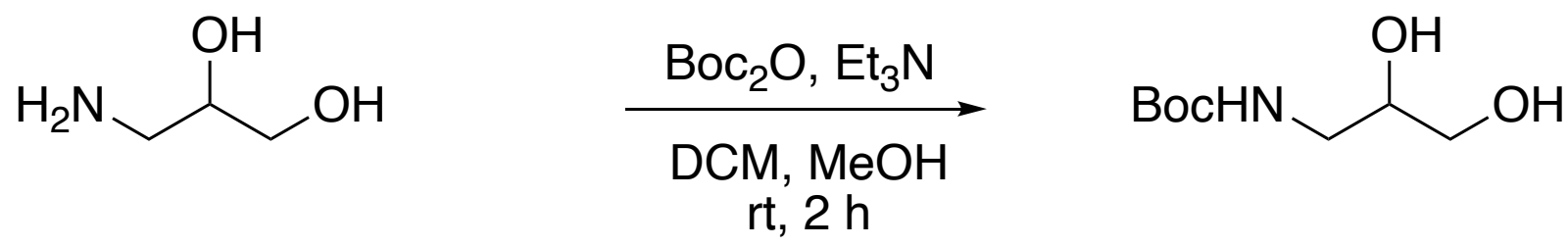

***tert*-butyl (2,3-dihydroxypropyl)carbamate.** (±)-3-amino-1,2-propanediol (11.3 g, 124 mmol) was dissolved in 126 mL of DCM:MeOH (1:5, v/v) mixture. Et_3N (2.0 mL, 14.3 mmol) was added then added followed by addition of Boc_2O (32.5 g, 149 mmol) solution in 186 mL of DCM. After stirring for 2 hours at rt, the solvents were removed under reduced pressure and the obtained residue purified via flash column chromatography over silica (packed in pentane:EtOAc, 4:1, v/v and washed with increasing gradient of EtOAc from 4:1 → 1:1 → 1:4 (v/v) and, finally, 100% EtOAc) in order to afford the Boc-protected diol (18.0 g, 76%) as a white solid.

^{1}H NMR (500 MHz, DMSO-d$_6$) δ[ppm] 6.57 (t, J = 5.8 Hz, 1H), 4.60 (d, J = 5.0 Hz, 1H), 4.45 (t, J = 5.8 Hz, 1H), 3.49-3.39 (m, 1H), 3.31-3.22 (m, 2H), 3.03 (dt, J = 13.4, 5.8 Hz, 1H), 2.84 (dt, J = 13.1, 6.1 Hz, 1H), 1.37 (s, 9H).
^{13}C NMR (101 MHz, DMSO-d$_6$) δ[ppm] 155.8, 77.6, 70.7, 63.8, 43.5, 28.2.

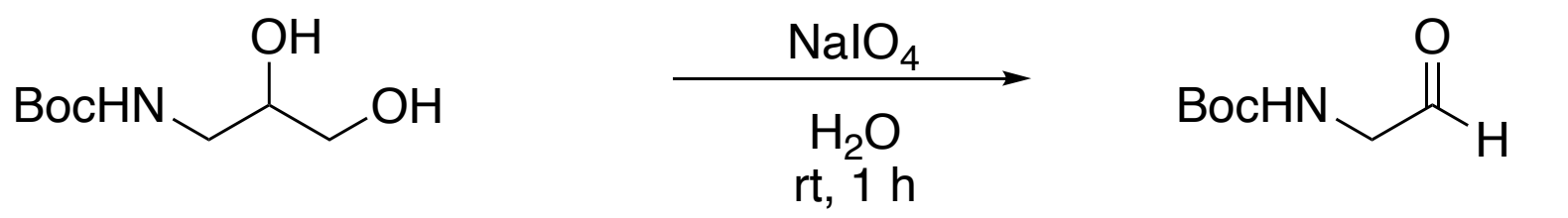

***tert*-butyl (2-oxoethyl)carbamate [Boc-glycinal].** *tert*-butyl (2,3-dihydroxypropyl)carbamate (1.36 g, 7.11 mmol, 1 eq) was dissolved in 12 mL of distilled water, the flask was covered with Al-foil and sodium periodate (1.84 g, 8.61 mmol, 1.2 eq) was added. The reaction mixture was stirred in the dark and at rt for 1 hour after which the reaction showed full conversion, as judged by TLC (pentane:EtOAc, 1:1, v/v). The mixture was then extracted four times with chloroform, the organic phase was dried over Na_2SO_4, filtered and evaporated to afford Boc-glycinal (0.88 g, 5.53 mmol, 78%) as a colourless oil which was used without further purification.

^{1}H NMR (500 MHz, DMSO-d$_6$) δ[ppm] 9.45 (s, 1H), 7.23-7.13 (m, 1H), 3.73 (d, J = 5.6 Hz, 2H), 1.39 (s, 9H).

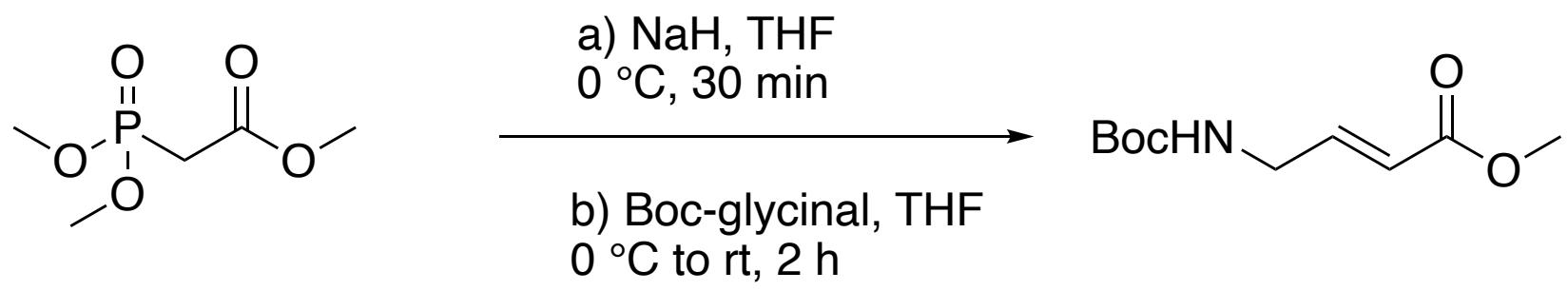

methyl (*E*)-4-((*tert*-butoxycarbonyl)amino)but-2-enoate. Commercially available olefination reagent methyl 2-(dimethoxyphosphoryl)acetate (2.4 mL, 14.7 mmol, 1.15 eq) and NaH (0.59 g, 14.7 mmol, 1.15 eq) were stirred in dry THF (50 mL) at 0 °C for 30 min. Subsequently, a solution of Boc-glycinal (2 g, 12.8 mmol, 1 eq) in dry THF (50 mL) was added and the reaction stirred at 0 °C for 30 min, warmed to rt, and stirred at rt for 1.5 h. The crude product was purified via flash column chromatography over silica (packed in DCM:EtOAc 49:1 v/v and washed with increasing gradient of EtOAc from DCM:EtOAc 49:1→ 23:2 v/v) to exclusively yield the E-isomer product as a colorless oil (1.9 g, 8.84 mmol, 69%).

^{1}H NMR (500 MHz, DMSO-d$_6$): δ[ppm] 7.17 (t, J = 5.8 Hz, 1H), 6.82 (dt, J = 15.7, 4.7 Hz, 1H), 5.85 (dt, J = 15.7, 2.0 Hz, 1H), 3.75 – 3.70 (m, 2H), 3.65 (s, 3H), 1.38 (s, 9H).

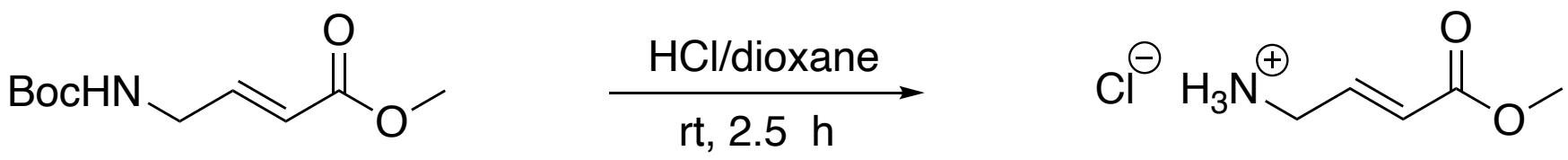

methyl (*E*)-4-aminobut-2-enoate. Boc-deprotection of methyl (*E*)-4-((*tert*-butoxycarbonyl)amino)but-2-enoate (0.48 g, 2.22 mmol, 1 eq) was performed with 4 M HCl/dioxane (3.3 mL, 13.3 mmol, 6 eq) diluted with dry dioxane (2.2 mL). The reaction was stirred at rt for 2.5 h and removal of solvent under reduced pressure afforded the product as an orange solid (0.27 g, 1.79 mmol, 80%) that was used without further purification.

^{1}H NMR (400 MHz, DMSO-d$_6$): δ[ppm] 8.49 (s, br, 3H), 6.87 (dt, J = 15.9, 5.7 Hz, 1H), 6.18 (dt, J = 15.9, 1.8 Hz, 1H), 3.69 (s, 3H), 3.66 (dd, J = 5.7, 1.8 Hz, 2H).
^{13}C NMR (101 MHz, DMSO-d$_6$): δ[ppm] 165.4, 140.7, 123.4, 51.7, 39.1.

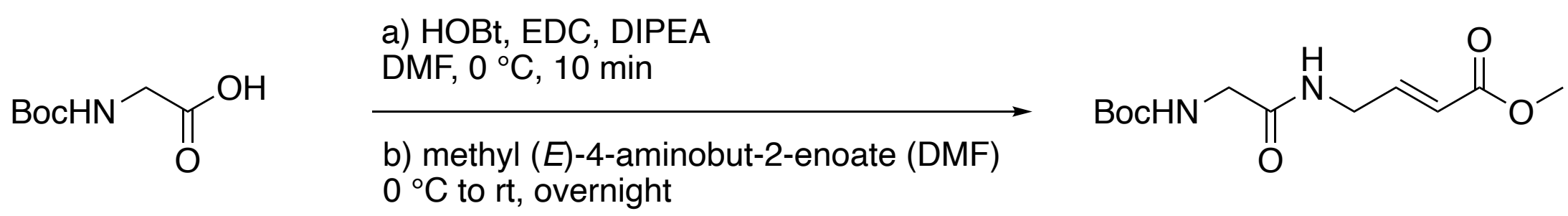

methyl (*E*)-4-(2-((*tert*-butoxycarbonyl)amino)acetamido)but-2-enoate [Boc-GVME]. Commercially available Boc-Gly-OH (1.29 g, 7.38 mmol, 1 eq), EDC x HCl (1.56 g, 8.11 mmol, 1.1 eq), HOBt (1.24 g, 8.11 mmol, 1.1 eq) and DIPEA (2.63 mL, 15.5 mmol, 2.1 eq) were dissolved in dry DMF (17 mL) and stirred at 0 °C for 10 min. Next, a solution of methyl (*E*)-4-aminobut-2-enoate (1.12 g, 7.38 mmol, 1 eq) in dry DMF (10 mL) was added and the reaction stirred at rt overnight. The

crude product was purified via flash column chromatography over silica (DCM:EtOAc 13:7 → 2:3) to furnish the product as a colorless oil (931 mg, 3.42 mmol, 46%).

^{1}H NMR (300 MHz, DMSO-d$_6$): δ[ppm] 8.08 (t, J = 5.9 Hz, 1H), 6.99 (t, J = 6.1 Hz, 1H), 6.83 (dt, J = 15.8, 4.6 Hz, 1H), 5.90 (dt, J = 15.8, 2.0 Hz, 1H), 3.87 (ddd, J = 5.9, 4.6, 2.0 Hz, 2H), 3.65 (s, 3H), 3.55 (d, J = 6.1 Hz, 2H), 1.39 (s, 9H).
^{13}C NMR (75 MHz, DMSO-d$_6$): δ[ppm] 169.5, 166.0, 155.8, 146.2, 119.8, 78.1, 51.3, 43.3, 39.2, 28.2.

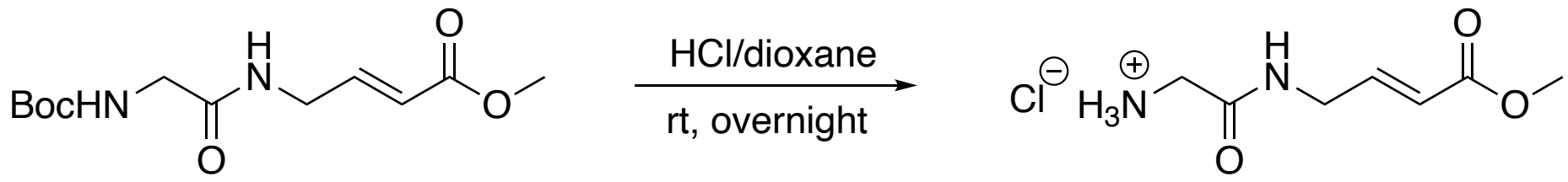

Methyl (*E*)-4-(2-aminoacetamido)but-2-enoate hydrochloride [GVME]. Boc-GVME, methyl (*E*)-4-(2-((*tert*-butoxycarbonyl)amino)acetamido)but-2-enoate, (0.78 g, 2.86 mmol, 1 eq) was dissolved in 10 mL of dry 1,4-dioxane and 10 mL of 4 M HCl in 1,4-dioxane were carefully added to the solution. The reaction mixture was stirred at room temperature overnight (Note: The deprotection reaction should be monitored carefully, since a +36 adduct was observed in LC-MS during prolonged reaction times (above 12 hours)) after which the solvent was removed under reduced pressure. The residue was re-dissolved in water, extracted with Et_2O and the aqueous phase was lyophilised to afford the hydrochloride salt of methyl 4-aminobut-2-enoate (0.59 g, 2.84 mmol, 98 %) as a brown foam.

^{1}H NMR (500 MHz, DMSO-d$_6$) δ[ppm] 8.96 (t, J = 5.7 Hz, 1H), 8.28 (s, 3H), 6.84 (dt, J = 15.8, 4.6 Hz, 1H), 5.98 (dt, J = 15.7, 2.0 Hz, 1H), 3.94 (ddd, J = 6.3, 4.7, 2.0 Hz, 2H), 3.65 (s, 3H), 3.62 (bs, 2H).
^{13}C NMR (75 MHz, DMSO-d$_6$) δ[ppm] 166.1, 165.9, 145.4, 120.2, 51.4, 40.1, 39.4.

2.9.3 Synthesis of GVDMA

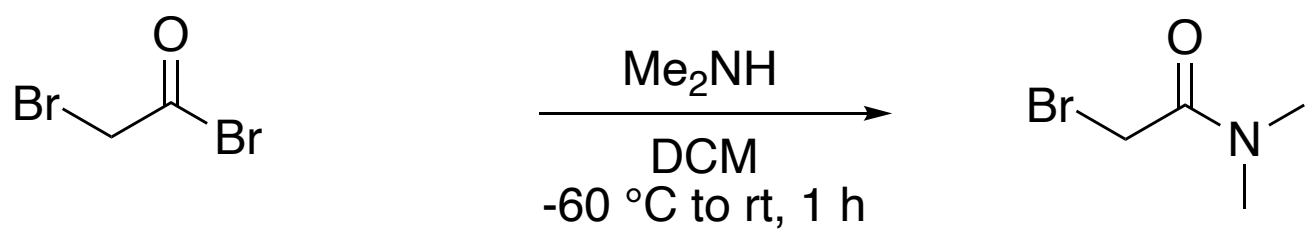

2-bromo-*N,N*-dimethylacetamide. Commercially available bromoacetyl bromide (1 mL, 13.5 mmol, 1 eq) was dissolved in dry DCM (15 mL) and cooled to -60 °C in a cooling bath prepared from EtOAc and liquid nitrogen. To the stirring mixture, a solution of 2 M Me_2NH in dry THF (13.5 mL, 27 mmol, 2 eq) was added dropwise, after which the reaction was allowed to warm to ambient temperature and stirred for 1 h. The reaction was then quenched with water (10 mL) and the organic solvent removed under reduced pressure. The crude aqueous phase was extracted with DCM (4x 30 mL) and the combined organic phases washed with water (50 mL), a saturated aqueous solution of $NaHCO_3$ (50 mL), and brine (50 mL). Subsequently, the organic phase was dried over Na_2SO_4, filtered and concentrated in vacuo. Flash column chromatography over silica (pentane:EtOAc gradient 1:1 → 1:2.5

v/v) and removal of solvent under reduced pressure afforded the product as a solid (1.14 g, 6.86 mmol, 51%).

^{1}H NMR (500 MHz, DMSO-d$_6$,): *δ*[ppm] 4.10 (s, 2H), 3.02 (s, 3H), 2.84 (s, 3H).

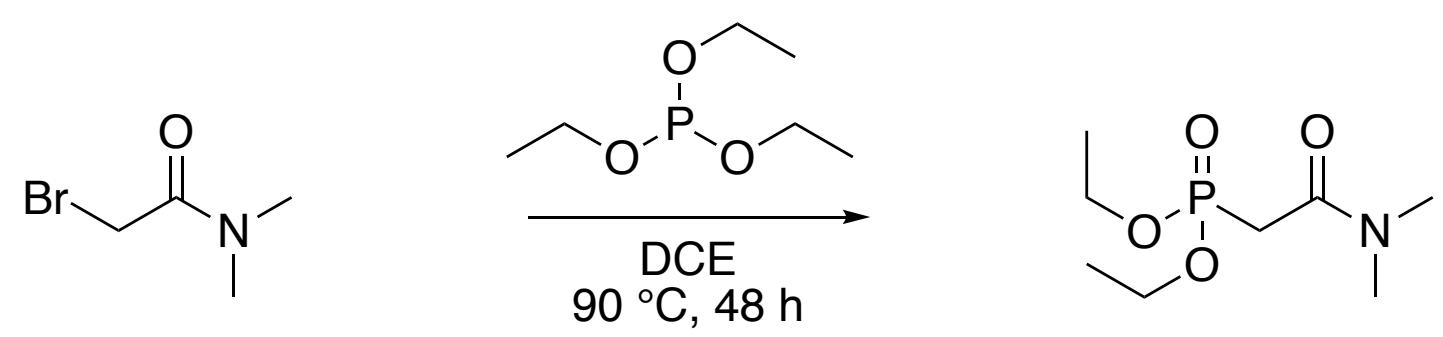

diethyl (2-(dimethylamino)-2-oxoethyl)phosphonate. 2-bromo-*N,N*-dimethylacetamide (1.14 g, 6.86 mmol, 1 eq) was dissolved in dry DCE (3 mL), triethyl phosphite (1.76 mL, 10.3 mmol, 1.5 eq) was added and the mixture heated to reflux. The reaction was stirred at 90 °C for 48 h until full conversion of starting material was observed via TLC. Then the reaction was concentrated under reduced pressure and the crude product purified via flash column chromatography over silica (DCM:MeOH gradient 97:3 → 9:1 v/v). The desired olefination reagent was obtained as a white solid (1.23 g, 5.51 mmol, 80%).

^{1}H NMR (300 MHz, DMSO-d$_6$): *δ*[ppm] 4.02 (dq, $^2J_{H\text{-}P}$ = 8.1, 7.1 Hz, 4H), 3.10 (d, $^1J_{H\text{-}P}$ = 21.6 Hz, 2H), 3.03 (d, $^4J_{H\text{-}P}$ = 0.5 Hz, 3H), 2.82 (d, $^4J_{H\text{-}P}$ = 1.4 Hz, 3H), 1.22 (td, J = 7.1, $^3J_{H\text{-}P}$ = 0.5 Hz, 6H).

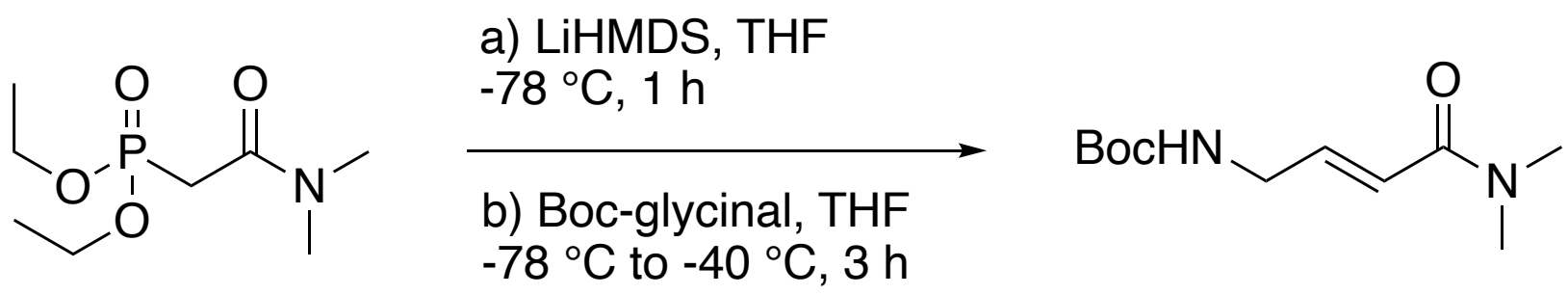

***tert*-butyl (*E*)-(4-(dimethylamino)-4-oxobut-2-en-1-yl)carbamate [Boc-VDMA].** Olefination reagent diethyl (2-(dimethylamino)-2-oxoethyl)phosphonate (410 mg, 1.8 mmol, 1.2 eq) was dissolved in dry THF (7.5 mL) in a heat-dried Schlenk-flask under argon atmosphere. The solution was cooled to -78 °C in a cooling bath prepared from EtOAc and liquid nitrogen. Then a 1 M solution of LiHMDS in THF (1.84 mL, 1.8 mmol, 1.2 eq) was added dropwise. Boc-glycinal (244 mg, 1.5 mmol, 1 eq) was dissolved in dry THF (3 mL) and added dropwise to the reaction at -78 °C. The mixture was stirred at -78 °C for 1 h, allowed to warm to -40 °C and stirred at -40 °C for 2 h. Then, the reaction was quenched with a saturated aqueous solution of $NaHCO_3$ (4 mL) and THF was evaporated under reduced pressure. The crude aqueous phase was extracted with Et_2O (3x 30 mL) and the combined organic phases washed with a saturated aqueous solution of $NaHCO_3$ (30 mL), aqueous 10% $KHSO_4$ (w/v) (30 mL), and brine (30 mL). Next, the organic phase was dried over Na_2SO_4, filtered and concentrated under reduced pressure. Flash column chromatography over silica (DCM:MeOH, gradient 49:1 → 24:1 v/v) afforded a 1:1.5 mixture (*E:Z*) of Boc-VDMA as colorless oil (200 mg, 0.88 mmol, 57%).

^{1}H NMR (500 MHz, DMSO-d$_6$): δ[ppm] 7.13 (s, 1H), 6.52 (dt, J = 15.2, 5.0 Hz, 1H), 6.42 (dt, J = 15.2, 1.7 Hz, 1H), 3.73 – 3.66 (m, 2H), 3.01 (s, 3H), 2.86 (s, 3H), 1.39 (s, 9H).

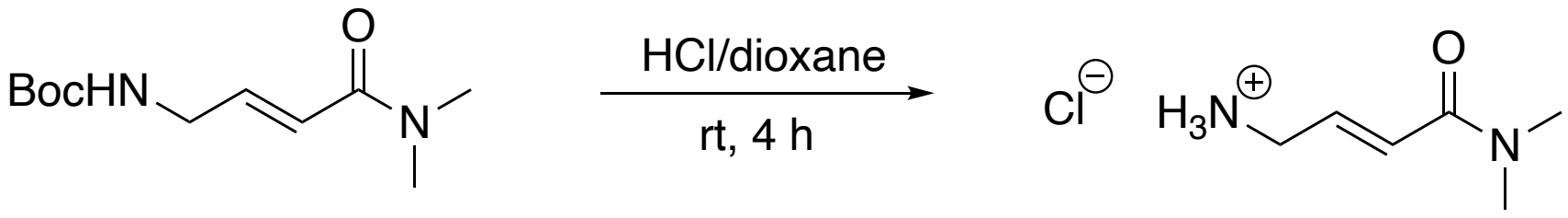

(*E*)-4-amino-*N*,*N*-dimethylbut-2-enamide [VDMA]. Deprotection of *tert*-butyl (*E*)-(4-(dimethylamino)-4-oxobut-2-en-1-yl)carbamate, Boc-VDMA, (200 mg, 0.88 mmol, 1 eq) was performed with 4 M HCl/dioxane (0.8 mL, 3.2 mmol, 3.6 eq) diluted with dry dioxane (0.8 mL). The reaction was stirred at rt for 4 h and removal of solvent under reduced pressure afforded the product (1:1.5-mixture *E:Z*) as a beige powder (0.14 g, 0.82 mmol, 94%) that was used without further purification.

^{1}H NMR (500 MHz, DMSO-d$_6$): δ[ppm] 8.10 (s, br, 3H), 6.76 (dt, J = 15.3, 1.6 Hz, 1H), 6.59 (dt, J = 15.3, 5.8 Hz, 1H), 3.64 (d, J = 5.8 Hz, 2H), 3.06 (s, 3H), 2.89 (s, 3H).

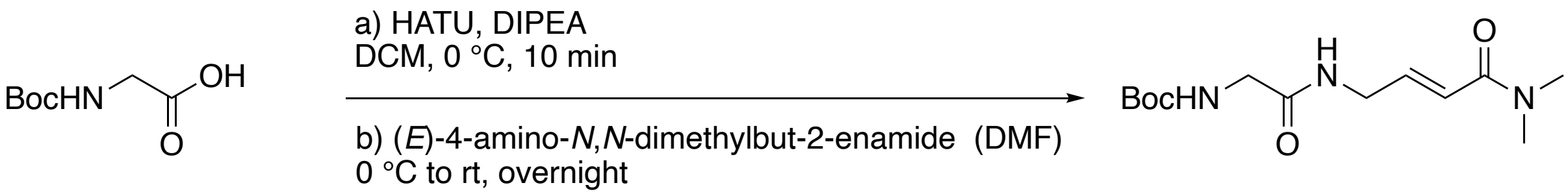

***tert*-butyl (*E*)-(2-((4-(dimethylamino)-4-oxobut-2-en-1-yl)amino)-2-oxoethyl)carbamate [Boc-GVDMA].** Commercially available Boc-Gly-OH (0.13 g, 0.75 mmol, 1 eq) and DIPEA were dissolved in dry DCM (10 mL) and the mixture cooled to 0 °C. To the stirring solution HATU (0.34 g, 0.9 mmol, 1.2 eq) was added and the mixture stirred at 0 °C for 10 min. (*E*)-4-amino-*N*,*N*-dimethylbut-2-enamide, VDMA, was dissolved in dry DMF (3 mL) and added to the reaction that was subsequently allowed to warm to ambient temperature and stirred overnight. The mixture was diluted with an aqueous solution of 10% LiCl (w/v) (40 mL) and the organic phase separated. The aqueous phase was extracted with DCM (3x 15 mL) and the combined organic phases washed with an aqueous solution of 10% LiCl (w/v) (40 mL), water (40 mL), and brine (40 mL). The organic phase was dried over Na_2SO_4, filtered and concentrated under reduced pressure. Flash column chromatography over silica (DCM:MeOH, gradient 47:3 → 23:2 v/v) furnished the product Boc-GVDMA as a solid (41 mg, 0.14 mmol, 19%).

^{1}H NMR (500 MHz, DMSO-d$_6$): δ[ppm] 8.05 (t, J = 5.8 Hz, 1H), 7.01 (t, J = 6.0 Hz, 1H), 6.55 (dt, J = 15.1, 4.6 Hz, 1H), 6.46 (dt, J = 15.1, 1.7 Hz, 1H), 3.88 – 3.82 (m, 2H), 3.53 (d, J = 6.0 Hz, 2H), 3.03 (s, 3H), 2.86 (s, 3H), 1.38 (s, 9H).

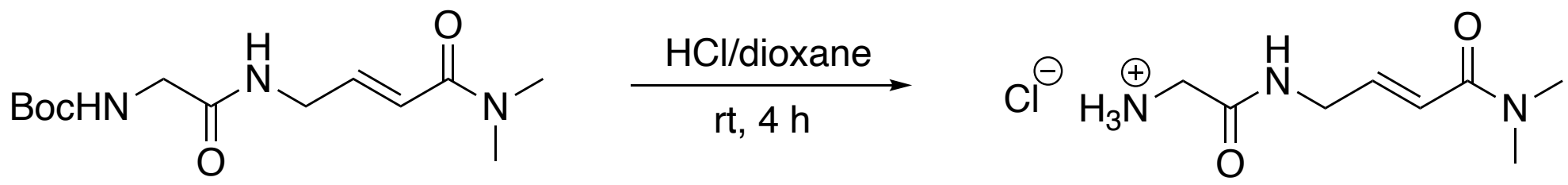

(*E*)-4-(2-aminoacetamido)-*N*,*N*-dimethylbut-2-enamide [GVDMA]. Deprotection of *tert*-butyl (*E*)-(2-((4-(dimethylamino)-4-oxobut-2-en-1-yl)amino)-2-oxoethyl)carbamate, Boc-GVDMA, (36 mg, 0.13 mmol, 1 eq) was performed with 4 M HCl/dioxane (0.1 mL, 0.4 mmol, 3.2 eq) diluted with dry dioxane (0.3 mL). The reaction was stirred at rt for 4 h and removal of solvent under reduced pressure afforded the product GVDMA as a white solid (13 mg, 0.06 mmol, 48%).

^{1}**H NMR** (500 MHz, CD_3OD): δ[ppm] 5.86 (dt, J = 15.3, 5.4 Hz, 1H), 5.74 (dt, J = 15.3, 1.7 Hz, 1H), 3.24 (dd, J = 5.4, 1.7 Hz, 2H), 2.91 (s, 2H), 2.32 (s, 3H), 2.18 (s, 3H).

2.9.4 Synthesis of ncAAs

AzGGisoK was synthesized as previously described.[9]

AzGVAisoK was synthesized as described in the following.

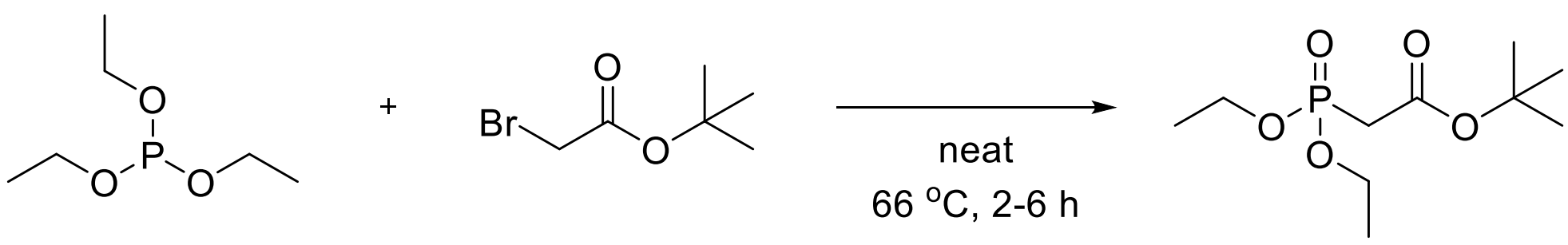

tert-butyl 2-(diethoxyphosphoryl)acetate. Triethyl phosphite (10 mL, 58 mmol, 1 eq) was mixed with *tert*-butyl bromoacetate (8.4 mL, 57 mmol, 0.98 eq) in a flame-dried 100 mL flask, neat. The resulting mixture was allowed to rotate on rotavap at 400 mbar, 66 ˚C for 2-6 h (control with HNMR). The product was obtained as a colorless liquid and was used in the next step without further purification (14.38 g, 57 mmol, 98%).

^{1}**H NMR** (400 MHz, $CDCl_3$) δ[ppm] 4.1 (dq, J = 8.1, 7.0 Hz, 4H), 2.9 (d, J = 21.5 Hz, 2H), 1.4 (s, 9H), 1.3 (td, J = 7.0, 0.6 Hz, 6H).

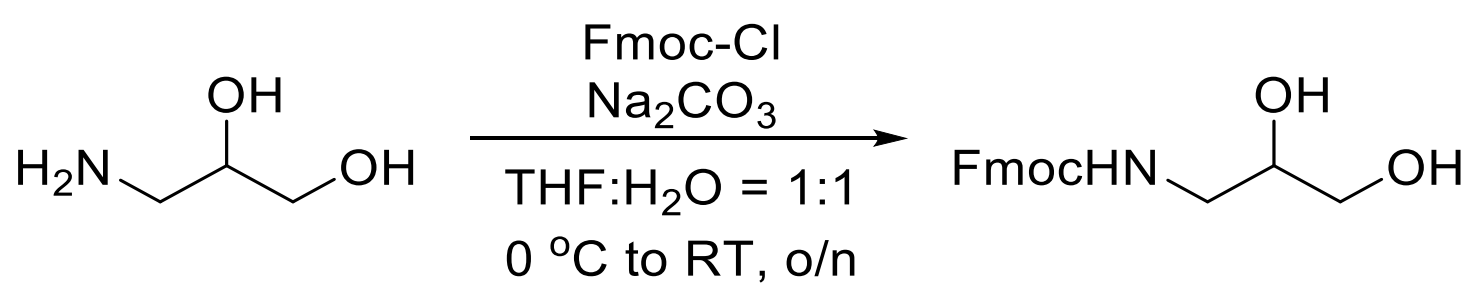

(9H-fluoren-9-yl)methyl (2,3-dihydroxypropyl)carbamate. 3-aminopropane-1,2-diol (4.8 g, 53 mmol, 1.0 eq) was dissolved in $THF:H_2O$ mixture 1:1 (v/v, 300 mL) followed by addition of Na_2CO_3 (5.6 g, 53 mmol, 1.0 eq) and the mixture was cooled to 0 ˚C. Fmoc-Cl (12.9 g, 50 mmol, 0.95 eq) was added portion-wise over 10 min and the mixture was allowed to stir at rt overnight. THF was removed *in vacuo* while the product precipitated. The product was filtered, washed with 5% aq. Na_2CO_3, 10%

citric acid and water. The product was dried on the glass filter resulting in white solid (16.5 g, 52.7 mmol, 99%) and was used in the next step without further purification.

^{1}H NMR (500 MHz, DMSO-d$_6$) δ[ppm] 7.89 (dt, *J* = 7.5, 1.0 Hz, 2H), 7.71 (d, *J* = 7.5 Hz, 2H), 7.42 (td, *J* = 7.5, 1.0 Hz, 2H), 7.33 (td, *J* = 7.5, 1.2 Hz, 2H), 7.17 (t, *J* = 5.7 Hz, 1H), 4.66 (d, *J* = 5.0 Hz, 1H), 4.47 (t, *J* = 5.7 Hz, 1H), 4.27 (d, *J* = 7.7 Hz, 2H), 4.20 (t, *J* = 7.0 Hz, 1H), 3.52 – 3.45 (m, 1H), 3.29 (td, *J* = 5.7, 2.7 Hz, 2H), 3.11 (dt, *J* = 13.3, 5.7 Hz, 1H), 2.92 (ddd, *J* = 13.3, 6.8, 5.7 Hz, 1H).

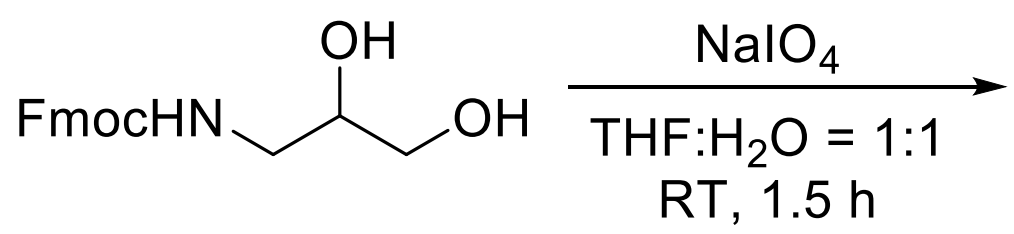

(9H-fluoren-9-yl)methyl (2-oxoethyl)carbamate. (9H-fluoren-9-yl)methyl (2,3-dihydroxypropyl)carbamate (16.5 g, 53 mmol, 1 eq) was dissolved in THF (400 mL) and $NaIO_4$ (14.25 g, 67.5 mmol, 1.3 eq) in water (400 mL) was slowly added (over 15-20 min). The reaction mixture was allowed to stir at rt for 1.5 h. THF was removed *in vacuo*. The aqueous residue was extracted with EtOAc (3x300 mL). Combined organics were dried over Na_2SO_4, filtered and concentrated *in vacuo* to obtain product as a white solid (14.5 g, 52 mmol, 98%) that was dried on a high vacuum overnight and was used in the next step without further purification.

^{1}H NMR (500 MHz, DMSO): *δ*[ppm] 9.46 (s, 1H), 7.90 (d, *J* = 7.5 Hz, 2H), 7.70 (m, 3H), 7.42 (t, *J* = 7.5 Hz, 2H), 7.34 (td, *J* = 7.4, 1.2 Hz, 2H), 4.34 (d, *J* = 6.9 Hz, 2H), 4.24 (t, *J* = 6.9 Hz, 1H), 3.83 (d, *J* = 5.7 Hz, 2H).

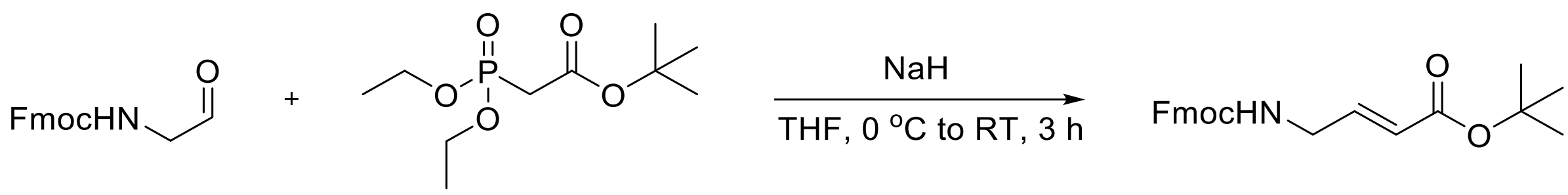

tert-butyl (E)-4-((((9H-fluoren-9-yl)methoxy)carbonyl)amino)but-2-enoate. Under Ar atmosphere in a flame-dried flask, NaH (60%, 0.16 g, 3.9 mmol, 1.15 eq) was suspended in THF (21 mL) and the suspension was cooled down to 0 ˚C. *Tert*-butyl 2-(diethoxyphosphoryl)acetate (0.95 mL, 4.04 mmol, 1.2 eq) was added dropwise and the mixture was allowed to stir at 0 ˚C for 30 min. (9H-fluoren-9-yl)methyl (2-oxoethyl)carbamate (0.95 g, 3.4 mmol, 1.0 eq) was added at 0 ˚C and the mixture was allowed to stir at rt for 2.5 h. The reaction was stopped by addition of carbonate buffer (100 mL, 0.5 M $NaHCO_3$, 0.5 M Na_2CO_3, pH 10-11). The mixture was extracted with EtOAc (3x50 mL) and the combined organics were washed with 100 mL $NaHSO_4$ (10%), dried over Na_2SO_4, filtered and concentrated *in vacuo*. The mixture was purified by flash column chromatography (0-50% EtOAc in Hexane) yielding product as a colorless to brownish solid (0.78 g, 2.1 mmol, 61%).

^{1}H NMR (500 MHz, DMSO-d$_6$): δ[ppm] 7.89 (d, J = 7.5 Hz, 2H), 7.70 (d, J = 7.4 Hz, 2H), 7.62 (t, J = 5.9 Hz, 1H), 7.42 (t, J = 7.4 Hz, 2H), 7.33 (td, J = 7.4, 1.2 Hz, 2H), 6.72 (dt, J = 15.7, 4.6 Hz, 1H), 5.75 (dt, J = 15.7, 1.9 Hz, 1H), 4.33 (d, J = 7.0 Hz, 2H), 4.23 (t, J = 7.0 Hz, 1H), 3.80 – 3.67 (m, 2H), 1.44 (s, 9H).

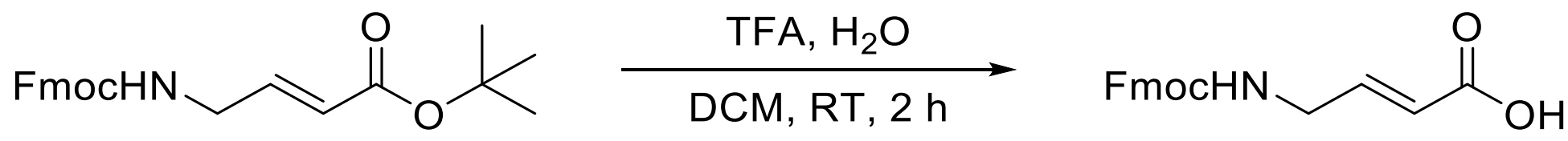

(E)-4-((((9H-fluoren-9-yl)methoxy)carbonyl)amino)but-2-enoic acid. The compound (1.6 g, 4.2 mmol) was dissolved in DCM and several drops of water were added. TFA (40% of the resulting volume) was slowly added to the stirring mixture and the resulting mixture was allowed to stir at rt for 2 h. All solvents were removed *in vacuo* and the resulting oil was co-evaporated with toluene twice to obtain product as a brown solid (1.36 g, 4.2 mmol, quant.).

^{1}H NMR (400 MHz, DMSO-d$_6$) δ[ppm] 12.29 (s, 1H), 7.89 (d, J = 7.5 Hz, 2H), 7.70 (d, J = 7.4 Hz, 2H), 7.65 (t, J = 5.9 Hz, 1H), 7.46 – 7.38 (m, 2H), 7.37-7.29 (m, 2H), 6.75 (dt, J = 15.7, 4.7 Hz, 1H), 5.79 (dt, J = 15.7, 2.0 Hz, 1H), 4.33 (d, J = 6.8 Hz, 2H), 4.23 (t, J = 6.9 Hz, 1H), 3.83 – 3.75 (m, 2H).

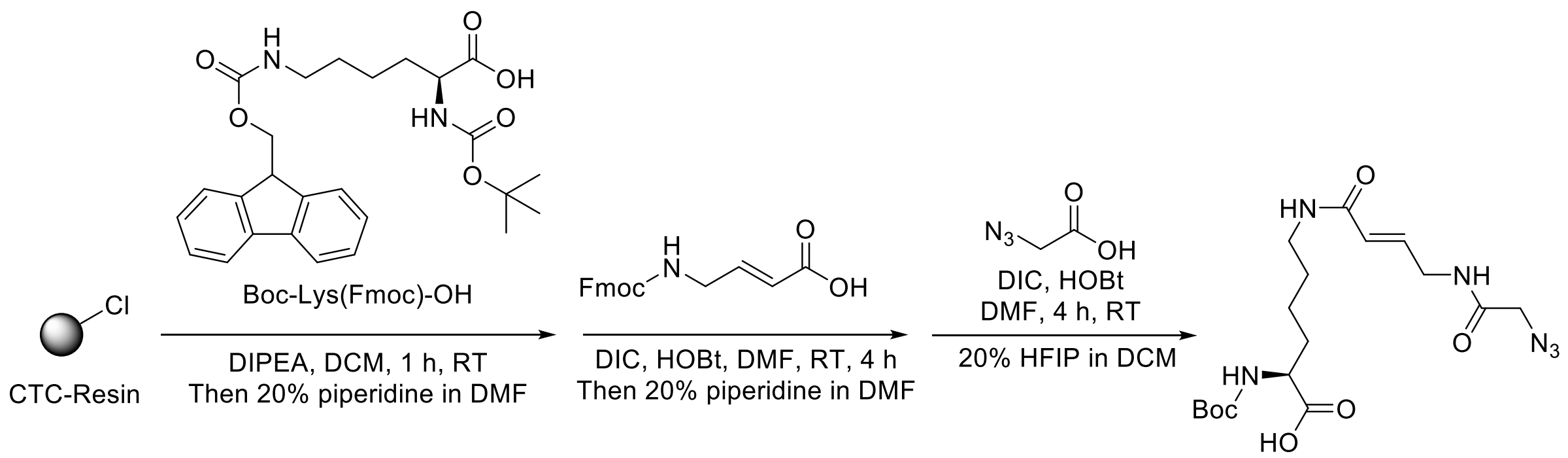

Boc-AzGVAK. CTC resin (2.8 mmol) was loaded into a column with a frit under Ar. DCM (40 mL) was added. Boc-Lys(Fmoc)-OH (3g, 6.4 mmol, 2.25 eq) was mixed with DIPEA (1.95 mL, 11.2 mmol, 4 eq) in 40 mL of DCM and added to the resin. The resulting mixture was agitated for 1 h. The resin was washed with DCM five times and with DMF twice. Fmoc deprotection was performed in 20% piperidine in DMF, two times 15 min each. Between these runs, the resin was washed twice with DMF. After deprotection, the resin was washed 5 times with DMF.

Prior to the coupling reaction, the resin was washed twice with dry DMF under Ar and was swollen in dry DMF. (E)-4-((((9H-fluoren-9-yl)methoxy)carbonyl)amino)but-2-enoic acid (1.36 g, 4.2 mmol, 1.5 eq) and HOBt (0.814 g, 6 mmol, 2.15 eq) were dissolved in dry DMF (40 mL) and added to the resin. DIC (1.32 mL, 8.4 mmol, 3 eq) was added and the reaction mixture was agitated for 5 h. The resin was

washed 5 times with DMF. Fmoc deprotection was performed in 20% piperidine in DMF, two times 15 min each. Between these runs, the resin was washed twice with DMF. After deprotection, the resin was washed 5 times with DMF.

Prior to the coupling reaction, the resin was washed twice with dry DMF under Ar and was swollen in dry DMF. Azidoacetic acid (0.85 g, 8.4 mmol, 3 eq) and HOBt (0.814 g, 6 mmol, 2.15 eq) were mixed in DMF (40 mL) and added to the resin. DIC (1.55 mL, 10 mmol, 3.5 eq) was added and the reaction mixture was agitated for 5 h. The resin was washed 5 times with DMF and 5 times with DCM.

The cleavage of the amino acid was performed by washing the resin 3 times 15 min each with 20% HFIP in DCM. Between these runs, the resin was washed with DCM 3 times. All filtrates were combined and concentrated *in vacuo*. The mixture was purified by flash column chromatography (0-10% MeOH in DCM, 0.5% AcOH). The product was lyophilized. Yields over SPPS were within the range of 65-75% (white solid).

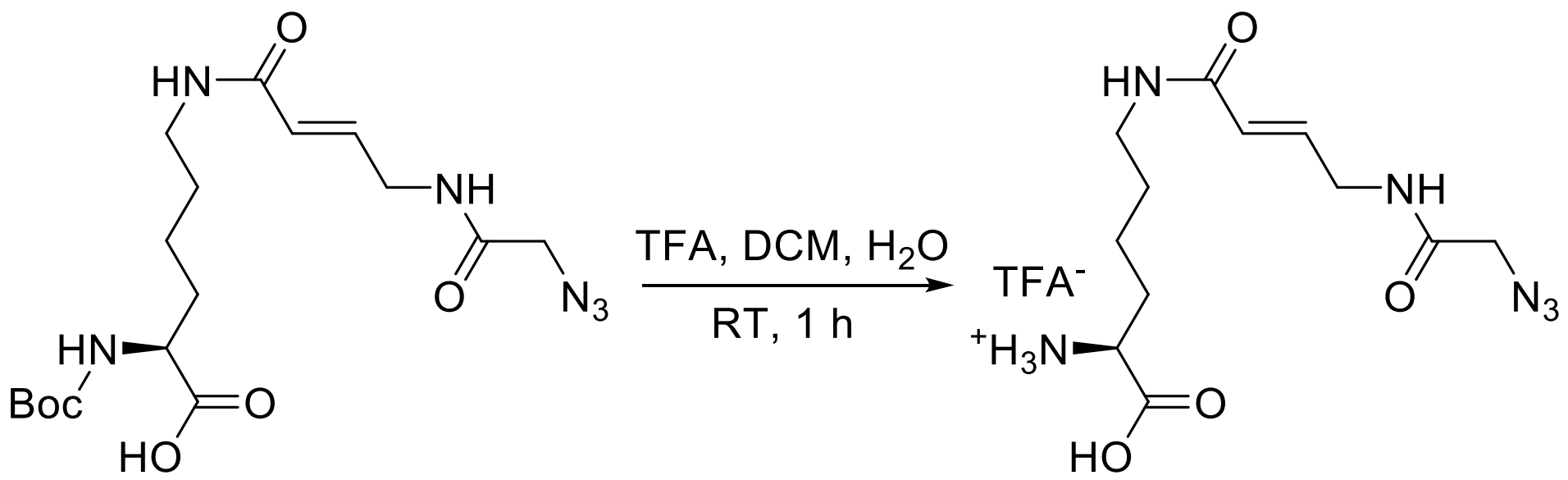

AzGVAK. Boc-AzGVAK (3.1 g, 7.5 mmol) was dissolved in DCM (60 mL) and 1 mL of water was added. TFA (40 mL) was slowly added and the mixture was allowed to stir at rt for 1 h. All solvents were removed *in vacuo* and the residue was dissolved in water and lyophilized. Yields were quantitative (3.1 g, 7.3 mmol) and the product was obtained as a white to yellowish solid.

^{1}H NMR (500 MHz, D_2O) δ[ppm] 6.68 (dt, J = 15.6, 4.9 Hz, 1H), 6.02 (dt, J = 15.6, 1.9 Hz, 1H), 4.09 (s, 2H), 4.07-3.98 (m, 2H), 3.96-3.83 (m, 1H), 3.27 (t, J = 6.9 Hz, 2H), 2.08 – 1.83 (m, 2H), 1.64-1.54 (m, 2H), 1.55 – 1.35 (m, 2H).

^{13}C NMR (125 MHz, D_2O) δ[ppm] 174.0, 171.8, 169.2, 140.6, 124.3, 54.4, 53.1, 41.2, 40.1, 30.7, 29.1, 22.8.

HRMS (ESI-QTOF) $[M+H]^+$: calc. 313.1619, obs. 313.1617.

3. Plasmids and Proteins

3.1 Plasmids

Table S4: List of plasmids used in this study

Plasmid	Description
pET17b SUMO1(LLA)-H6	SUMO1(1-91)-LLAQTGG-HHHHHH (flanked by NdeI/EcoRI restriction sites) under inducible T7 promoter; ampicillin resistance
pET17b SUMO2(LLA)-H6	SUMO2(1-87)-LLAQTGG-HHHHHH (flanked by NdeI/EcoRI restriction sites) under inducible T7 promoter, ampicillin resistance
pET17b SUMO1(QLA)-H6	SUMO1(1-91)-QLAQTGG-HHHHHH (flanked by NdeI/EcoRI restriction sites) under inducible T7 promoter; ampicillin resistance
pET17b SUMO2(QLA)-H6	SUMO2(1-87)-QLAQTGG-HHHHHH (flanked by NdeI/EcoRI restriction sites) under inducible T7 promoter, ampicillin resistance
pET17b HA-SUMO1(QLA)-H6	N-terminally HA-tagged SUMO1(1-91)-QLAQTGG-HHHHHH (flanked by NdeI/EcoRI restriction sites) under inducible T7 promoter; ampicillin resistance
pET17b HA-SUMO2(QLA)-H6	N-terminally HA-tagged SUMO2(1-87)-QLAQTGG-HHHHHH (flanked by NdeI/EcoRI restriction sites) under inducible T7 promoter, ampicillin resistance
pET29b Srt2A-TEV-H6	*Staphylococcus aureus* sortase A mutant Srt2A(60-206)[10] with C-terminal TEV cleavage site and subsequent H6-tag (flanked by NdeI/StyI restriction sites) under inducible T7 promoter, kanamycin resistance
pET28a H6-Throm-SENP1(CD)	Catalytic domain of SENP1(419-644) with N-terminal thrombin-cleavable H6-tag, under inducible T7 promoter, kanamycin resistance;<br>pET28a-SENP1 (catalytic domain) was a gift from Guy Salvesen[11] (Addgene plasmid # 16356; http://n2t.net/addgene:16356 ; RRID:Addgene_16356)
pET28a H6-Throm-SENP2(CD)	Catalytic domain of SENP2(364-589) with N-terminal thrombin-cleavable H6-tag (SENP fraction flanked by NdeI/NotI restriction sites) under inducible T7 promoter, kanamycin resistance
pET28a H6-Throm-SENP5(CD)	Catalytic domain of SENP5(567-755) with N-terminal thrombin-cleavable H6-tag, under inducible T7 promoter, kanamycin resistance;<br>pET28a-SENP5 (catalytic domain) was a gift from Guy Salvesen[11] (Addgene plasmid # 16358; http://n2t.net/addgene:16358 ; RRID:Addgene_16358)
pET28a H6-TEV-SENP6(CD)	Catalytic domain of SENP6(637-1112) with N-terminal TEV-cleavable H6-tag (flanked by NcoI/XhoI restriction sites) under inducible T7 promoter, kanamycin resistance
pET28a H6-TEV-SENP7(CD)	Catalytic domain of SENP7(733-1050) with N-terminal TEV-cleavable H6-tag (flanked by NcoI/XhoI restriction sites) under inducible T7 promoter, kanamycin resistance
pET28a H6-TEV-SENP8	Full-length SENP8 with N-terminal TEV-cleavable H6-tag (flanked by NcoI/XhoI restriction sites) under inducible T7 promoter, kanamycin resistance
pET28a H6-Throm-USPL1(CD)	Catalytic domain of USPL1(212-514) with N-terminal thrombin-cleavable H6-tag (flanked by NcoI/HindIII restriction sites) under inducible T7 promoter, kanamycin resistance

pET28a H6-TEV-DeSi1	Full-length DeSi1 with N-terminal TEV-cleavable H6-tag (flanked by NcoI/XhoI restriction sites) under inducible T7 promoter, kanamycin resistance
pET28a H6-Throm-SENP2(CD, C548A)	Catalytically inactive mutant C548A of catalytic domain of SENP2(364-589) with N-terminal thrombin-cleavable H6-tag (SENP fraction flanked by NdeI/NotI restriction sites) under inducible T7 promoter, kanamycin resistance
pET28a H6-Throm-SUMO-*Mm*AzGVAisoK-RS	C-terminal domain of *Methanosarcina mazei* AzGVAisoK-RS(189-454) with N-terminal H6-Throm-SUMO-tag (flanked by NcoI/NotI restriction sites) under inducible T7 promoter, kanamycin resistance
pET28a H6-Throm-SUMO-*Mb*AzGVAisoK-RS	C-terminal domain of *Methanosarcina barkeri* AzGVAisoK-RS(154-419) with N-terminal H6-Throm-SUMO-tag (flanked by NcoI/NotI restriction sites) under inducible T7 promoter, kanamycin resistance
pTXB1 HA-SUMO1(ΔG)-MxeGyrA-CBD	N-terminally HA-tagged SUMO1(1-96) with C-terminal fusion of MxeGyrA(N198A) intein and chitin binding domain (CBD) (flanked by NdeI/PstI restriction sites) under inducible T7 promoter, ampicillin resistance
pTXB1 HA-SUMO1(ΔG)-MxeGyrA-CBD	N-terminally HA-tagged SUMO1(1-92) with C-terminal fusion of MxeGyrA(N198A) intein and chitin binding domain (CBD) (flanked by NdeI/PstI restriction sites) under inducible T7 promoter, ampicillin resistance
pPylT sfGFP(N150TAG)-H6, *Mm*PylT	Superfolder GFP(V2P, N150TAG) with C-terminal H6-tag under arabinose inducible promoter, suppressor tRNA *Mathanosarcina mazei* PylT under lpp promoter, tetracycline resistance
pBAD sfGFP(N150TAG)-H6	Superfolder GFP(V2P, N150TAG) with C-terminal H6-tag under arabinose inducible promoter, ampicillin resistance
pBAD Ub(K11TAG)-H6	Ubiquitin(K11TAG) with C-terminal H6-tag (flanked by NdeI/XhoI restriction sites) under arabinose inducible promoter, ampicillin resistance
pPylT SUMO2(K11TAG)-H6, *Mm*PylT	SUMO2(K11TAG) with C-terminal H6-tag (flanked by NcoI/XhoI restriction sites) under arabinose inducible promoter, suppressor tRNA *Mathanosarcina mazei* PylT under lpp promoter, tetracycline resistance
pBAD SUMO2(K11TAG)-H6	SUMO2(K11TAG) with C-terminal H6-tag (flanked by NdeI/XhoI restriction sites) under arabinose inducible promoter, ampicillin resistance
pBAD SUMO2(K11TAG, ΔG)-H6	SUMO2(1-K11TAG) with C-terminal H6-tag (flanked by NdeI/XhoI restriction sites) under arabinose inducible promoter, ampicillin resistance
pBAD PCNA-H6	PCNA with C-terminal H6-tag under arabinose inducible promoter, ampicillin resistance
pBAD PCNA(K164TAG)-H6	PCNA(K164TAG) with C-terminal H6-tag under arabinose inducible promoter, ampicillin resistance
pBK *Mb*PylRS	*Methanosarcina barkeri* PylRS (flanked by NdeI/PstI restriction sites) under constitutive glnS promoter, kanamycin resistance
pEVOL *Mb*PylRS, *Mm*PylT	Two copies of *Methanosarcina barkeri* PylRS under a constitutive glnS promoter and under an arabinose inducible promoter (flanked by NdeI/PstI and BglII/SalI restriction sites, respectively), suppressor tRNA *Mathanosarcina mazei* PylT under proK promoter, chloramphenicol resistance
pBK *Mb*AzGGisoK-RS	*Methanosarcina barkeri* AzGGisoK-RS (mutations from wt PylRS: L274A, N311Q, C313S; flanked by NdeI/PstI restriction sites) under constitutive glnS promoter, kanamycin resistance

pEVOL *Mb*AzGGisoK-RS, *Mm*PylT	Two copies of *Methanosarcina barkeri* AzGGisoK-RS (mutations from wt PylRS: L274A, N311Q, C313S) under a constitutive glnS promoter and under an arabinose inducible promoter (flanked by NdeI/PstI and BglII/SalI restriction sites, respectively), suppressor tRNA *Mathanosarcina mazei* PylT under proK promoter, chloramphenicol resistance
pBK *Mb*PylRS (L274A, N311Q, C313V)	*Methanosarcina barkeri* PylRS (L274A, N311Q, C313V, flanked by NdeI/PstI restriction sites) under constitutive glnS promoter, kanamycin resistance
pBK *Mb*PylRS (L274A, N311Q, C313V, M315Y)	*Methanosarcina barkeri* PylRS (L274A, N311Q, C313V, M315Y, flanked by NdeI/PstI restriction sites) under constitutive glnS promoter, kanamycin resistance
pBK *Mm*AzGGisoK-RS	*Methanosarcina mazei* AzGGisoK-RS (mutations from wt PylRS: L309A, N346Q, C348S) under constitutive glnS promoter, kanamycin resistance
pBK *Mm*AzGGisoK-RS (P188G)	*Methanosarcina mazei* AzGGisoK-RS(P188G) (mutations from wt PylRS: P188G, L309A, N346Q, C348S) under constitutive glnS promoter, kanamycin resistance
pBK *Ma*AzGGisoK-RS(IP)	*Methanomethylophilus alvus* AzGGisoK-RS with second layer IP mutations (mutations from wt PylRS: M129A, N166Q, V168S, H227I, Y228P) under constitutive glnS promoter, kanamycin resistance
pPylT sfGFP(N150TAG)-H6, *Ma*PylT	Superfolder GFP(V2P, N150TAG) with C-terminal H6-tag under arabinose inducible promoter, suppressor tRNA *Methanomethylophilus alvus* PylT under lpp promoter, tetracycline resistance
pEVOL *Ma*AzGGisoK-RS, *Ma*PylT	Two copies of *Methanomethylophilus alvus* AzGGisoK-RS with second layer IP mutations (mutations from wt PylRS: M129A, N166Q, V168S, H227I, Y228P) under a constitutive glnS promoter and under an arabinose inducible promoter, suppressor tRNA *Methanomethylophilus alvus* PylT under proK promoter, chloramphenicol resistance
pBK *Mb*AzGVAisoK-RS	*Methanosarcina barkeri* AzGVAisoK-RS (mutations from wt PylRS: Y271A, N311Q, C313S, Y349W; flanked by NdeI/PstI restriction sites) under constitutive glnS promoter, kanamycin resistance
pEVOL *Mb*AzGVAisoK-RS, *Mm*PylT	Two copies of *Methanosarcina barkeri* AzGGisoK-RS (mutations from wt PylRS: Y271A, N311Q, C313S, Y349W) under a constitutive glnS promoter and under an arabinose inducible promoter (flanked by NdeI/PstI and BglII/SalI restriction sites, respectively), suppressor tRNA *Mathanosarcina mazei* PylT under proK promoter, chloramphenicol resistance
pBK *Ma*AzGVAisoK-RS	*Methanomethylophilus alvus* AzGVAisoK-RS (mutations from wt PylRS: Y126A, N166Q, V168S, Y206W; flanked by NdeI/PstI restriction sites) under constitutive glnS promoter, kanamycin resistance
pBK *Ma*AzGVAisoK-RS(IP)	*Methanomethylophilus alvus* AzGVAisoK-RS(IP) (mutations from wt PylRS: Y126A, N166Q, V168S, Y206W, H227I, Y228P; flanked by NdeI/PstI restriction sites) under constitutive glnS promoter, kanamycin resistance
pBK *Mb*PylRS library (ABShuffle)	*Methanosarcina barkeri* PylRS library (randomized by DNA shuffling and error prone PCR, previously published) under constitutive glnS promoter, kanamycin resistance

pPylT CAT(111TAG)	Single reporter positive selection plasmid: Chloramphenicol-acetyltransferase with amber codon CAT(111TAG) under constitutive promoter, suppressor tRNA *Mathanosarcina mazei* PylT under lpp promoter, tetracycline resistance
pYOBB barnase(3TAG, 45TAG)	Negative selection plasmid: barnase(3TAG, 45TAG) under arabinose inducible promoter, suppressor tRNA *Mathanosarcina mazei* PylT under lpp promoter, chloramphenicol resistance
pPylT CAT(111TAG), sfGFP(N150TAG)-H6	Dual reporter positive selection plasmid: Chloramphenicol-acetyltransferase with amber codon CAT(111TAG) under constitutive promoter, superfolder GFP(150TAG)-H6 under arabinose inducible promoter, suppressor tRNA *Mathanosarcina mazei* PylT under lpp promoter, tetracycline resistance
pIRES FLAG-SENP1(CD)	Catalytic domain of SENP1(419-644) with N-terminal FLAG-tag (flanked by BamHI/NotI restriction sites) under CMV promoter, ampicillin resistance
pIRES FLAG-SENP1(CD, C603A)	Catalytically inactive mutant C603A of catalytic domain of SENP1(419-644) with N-terminal FLAG-tag (flanked by BamHI/NotI restriction sites) under CMV promoter, ampicillin resistance
pFLAG-CMV SENP1(FL)	Full-length SENP1 with N-terminal FLAG-tag under CMV promoter, ampicillin resistance<br>Flag-SENP1 was a gift from Edward Yeh[12] (Addgene plasmid # 17357; http://n2t.net/addgene:17357 ; RRID:Addgene_17357),<br>Comment: The Addgene plasmid contains 3 mutations: I193F, T207A, T623A
pFLAG-CMV SENP1(FL, C603A)	Catalytically inactive mutant C603A of full-length SENP1 with N-terminal FLAG-tag, ampicillin resistance<br>Contains additional mutations: I193A, T207A, T623A
pIRES FLAG-SENP2(CD)	Catalytic domain of SENP2(364-589) with N-terminal FLAG-tag (flanked by BamHI/NotI restriction sites) under CMV promoter, ampicillin resistance
pIRES HA-SUMO2(QLA)x-T2AP2A-Srt2A-Myc	N-terminally HA-tagged SUMO2(QLA)x (with x denoting a short helical tag that replaces G94 to prevent) and C-terminally Myc-tagged Srt2A separated by self-cleaving peptide sequence T2AP2A under CMV promoter, ampicillin resistance

3.2 Amino acid sequences

SUMO1(LLA)-H6

MSDQEAKPSTEDLGDKKEGEYIKLKVIGQDSSEIHFKVKMTTHLKKLKESYCQRQGVPMNSLRFLFEG
QRIADNHTPKELGMEEEDVIEVYLLAQTGGHHHHHH

SUMO2(LLA)-H6

MADEKPKEGVKTENNDHINLKVAGQDGSVVQFKIKRHTPLSKLMKAYCERQGLSMRQIRFRFDGQPIN
ETDTPAQLEMEDEDTIDVFLLAQTGGHHHHHH

SUMO1(QLA)-H6

MSDQEAKPSTEDLGDKKEGEYIKLKVIGQDSSEIHFKVKMTTHLKKLKESYCQRQGVPMNSLRFLFEG
QRIADNHTPKELGMEEEDVIEVYQLAQTGGHHHHHH

SUMO2(QLA)-H6

MADEKPKEGVKTENNDHINLKVAGQDGSVVQFKIKRHTPLSKLMKAYCERQGLSMRQIRFRFDGQPIN
ETDTPAQLEMEDEDTIDVFQLAQTGGHHHHHH

HA-SUMO1(QLA)-H6

MAYPYDVPDYAGSMSDQEAKPSTEDLGDKKEGEYIKLKVIGQDSSEIHFKVKMTTHLKKLKESYCQRQ
GVPMNSLRFLFEGQRIADNHTPKELGMEEEDVIEVYQLAQTGGHHHHHH

HA-SUMO2(QLA)-H6

MAYPYDVPDYAGSMADEKPKEGVKTENNDHINLKVAGQDGSVVQFKIKRHTPLSKLMKAYCERQGLS
MRQIRFRFDGQPINETDTPAQLEMEDEDTIDVFQLAQTGGHHHHHH

Srt2A-TEV-H6

MQAKPQIPKDKSKVAGYIEIPDADIKEPVYPGPATREQLNRGVCFHDENESLDDQNISIAGHTFIDRPNY
QFTNLKAAKPGSMVYFKVGNETRIYKMTSIRKVHPNAVEVLDEQEGKDKQLTLVTCDDYNEETGVWE
SRKIFVATEVKGSENLYFQGHHHHHH

H6-Throm-SENP1(CD:419-644), Addgene #16356

MGSSHHHHHHSSGLVPRGSHMEFPEITEEMEKEIKNVFRNGNQDEVLSEAFRLTITRKDIQTLNHLNWL
NDEIINFYMNMLMERSKEKGLPSVHAFNTFFFTKLKTAGYQAVKRWTKKVDVFSVDILLVPIHLGVHW
CLAVVDFRKKNITYYDSMGGINNEACRILLQYLKQESIDKKRKEFDTNGWQLFSKKSQEIPQQMNGSD
CGMFACKYADCITKDRPINFTQQHMPYFRKRMVWEILHRKLL

H6-Throm-SENP2(CD: 364-589)

MPGSSHHHHHHSSGLVPRGSHMDLLELTEDMEKEISNALGHGPQDEILSSAFKLRITRGDIQTLKNYHW
LNDEVINFYMNLLVERNKKQGYPALHVFSTFFYPKLKSGGYQAVKRWTKGVNLFEQEIILVPIHRKVH
WSLVVIDLRKKCLKYLDSMGQKGHRICEILLQYLQDESKTKRNSDLNLLEWTHHSMKPHEIPQQLNGS
DCGMFTCKYADYISRDKPITFTQHQMPLFRKKMVWEILHQQLL

H6-Throm-SENP5(CD: 567-755), Addgene #16358

MGSSHHHHHHSSGLVPRGSHMLDMDDLATLDGQNWLNDQVINMYGELIMDAVPDKVHFFNSFFHRQ
LVTKGYNGVKRWTKKVDLFKKSLLLIPIHLEVHWSLITVTLSNRIISFYDSQGIHFKFCVENIRKYLLTEA
REKNRPEFLQGWQTAVTKCIPQQKNDSDCGVFVLQYCKCLALEQPFQFSQEDMPRVRKRIYKELCECR
LMD

H6-TEV-SENP6(CD: 637-1112)

MGSSHHHHHHSSGENLYFQGEEETGENHTIFIGPVEKLIVYPPPPAKGGISVTNEDLHCLNEGEFLNDVII
DFYLKYLVLEKLKKEDADRIHIFSSFFYKRLNQRERRNHETTNLSIQQKRHGRVKTWTRHVDIFEKDFIF
VPLNEAAHWFLAVVCFPGLEKPKYEPNPHYHENAVIQKCSTVEDSCISSSASEMESCSQNSSAKPVIKK
MLNKKHCIAVIDSNPGQEESDPRYKRNICSVKYSVKKINHTASENEEFNKGESTSQKVADRTKSENGLQ

NESLSSTHHTDGLSKIRLNYSDESPEAGKMLEDELVDFSEDQDNQDDSSDDGFLADDNCSSEIGQWHL
KPTICKQPCILLMDSLRGPSRSNVVKILREYLEVEWEVKKGSKRSFSKDVMKGSNPKVPQQNNFSDCG
VYVLQYVESFFENPILSFELPMNLANWFPPPRMRTKREEIRNIILKLQEDQSKEKRKHKDTYSTEAPLGE
GTEQYVNSISD

H6-TEV-SENP7(CD: 733-1050)

MGSSHHHHHHSSGENLYFQGDEEWREVRHTGLVQKLIVYPPPPTKGGLGVTNEDLECLEEGEFLNDVII
DFYLKYLILEKASDELVERSHIFSSFFYKCLTRKENNLTEDNPNLSMAQRRHKRVRTWTRHINIFNKDYIF
VPVNESSHWYLAVICFPWLEEAVYEDFPQTVSQQSQAQQSQNDNKTIDNDLRTTSTLSLSAEDSQSTES
NMSVPKKMCKRPCILILDSLKAASVQNTVQNLREYLEVEWEVKLKTHRQFSKTNMVDLCPKVPKQDN
SSDCGVYLLQYVESFFKDPIVNFELPIHLEKWFPRHVIKTKREDIRELILKLHLQQQKGSSS

H6-TEV-SENP8

MGSSHHHHHHSSGENLYFQGMDPVVLSYMDSLLRQSDVSLLDPPSWLNDHIIGFAFEYFANSQFHDCS
DHVSFISPEVTQFIKCTSNPAEIAMFLEPLDLPNKRVVFLAINDNSNQAAGGTHWSLLVYLQDKNSFFHY
DSHSRSNSVHAKQVAEKLEAFLGRKGDKLAFVEEKAPAQQNSYDCGMYVICNTEALCQNFFRQQTESL
LQLLTPAYITKKRGEWKDLITTLAKK

H6-Throm-USPL1(CD: 212-514)

MGSSHHHHHHSSGLVPRGSSSPMPLESKCTSFPQALCVQWKNAYALCWLDCILSALVHSEELKNTVTG
LCSKEESIFWRLLTKYNQANTLLYTSQLSGVKDGDCKKLTSEIFAEIETCLNEVRDEIFISLQPQLRCTLG
DMESPVFAFPLLLKLETHIEKLFLYSFSWDFECSQCGHQYQNRHMKSLVTFTNVIPEWHPLNAAHFGPC
NNCNSKSQIRKMVLEKVSPIFMLHFVEGLPQNDLQHYAFHFEGCLYQITSVIQYRANNHFITWILDADGS
WLECDDLKGPCSERHKKFEVPASEIHIVIWERKISQVTDKEAACLPL

H6-TEV-DeSi1

MGSSHHHHHHSSGENLYFQGMEPPNLYPVKLYVYDLSKGLARRLSPIMLGKQLEGIWHTSIVVHKDEF
FFGSGGISSCPPGGTLLGPPDSVVDVGSTEVTEEIFLEYLSSLGESLFRGEAYNLFEHNCNTFSNEVAQFLT
GRKIPSYITDLPSEVLSTPFGQALRPLLDSIQIQPPGGSSVGRPNGQS

H6-Throm-SENP2(CD: 364-589, C548A)

MGSSHHHHHHSSGLVPRGSHMDLLELTEDMEKEISNALGHGPQDEILSSAFKLRITRGDIQTLKNYHWL
NDEVINFYMNLLVERNKKQGYPALHVFSTFFYPKLKSGGYQAVKRWTKGVNLFEQEIILVPIHRKVHWS
LVVIDLRKKCLKYLDSMGQKGHRICEILLQYLQDESKTKRNSDLNLLEWTHHSMKPHEIPQQLNGSDA
GMFTCKYADYISRDKPITFTQHQMPLFRKKMVWEILHQQLL

H6-Throm-SUMO-*Mm*AzGVAisoK-RS(CTD: 189-454) (mutations from wt PylRS: Y306A, N346Q, C348S, Y384W)

MGSSHHHHHHSSGLVPRGSHMSDSEVNQEAKPEVKPEVKPETHINLKVSDGSSEIFFKIKKTTPLRRLM
EAFAKRQGKEMDSLRFLYDGIRIQADQTPEDLDMEDNDIIEAHREQIGGSALTKSQTDRLEVLLNPKDEI

SLNSGKPFRELESELLSRRKKDLQQIYAEERENYLGKLEREITRFFVDRGFLEIKSPILIPLEYIERMGIDN
DTELSKQIFRVDKNFCLRPMLAPNLANYLRKLDRALPDPIKIFEIGPCYRKESDGKEHLEEFTMLQFSQM
GSGCTRENLESIITDFLNHLGIDFKIVGDSCMVWGDTLDVMHGDLELSSAVVGPIPLDREWGIDKPWIG
AGFGLERLLKVKHDFKNIKRAARSESYYNGISTNL

H6-Throm-SUMO-*Mb*AzGVAisoK-RS(CTD: 154-419) (mutations from wt PylRS: Y271A, N311Q, C313S, Y349W)

MGSSHHHHHHSSGLVPRGSHMSDSEVNQEAKPEVKPEVKPETHINLKVSDGSSEIFFKIKKTTPLRRLM
EAFAKRQGKEMDSLRFLYDGIRIQADQTPEDLDMEDNDIIEAHREQIGGSSLTRSQLDRVEALLSPEDKIS
LNMAKPFRELEPELVTRRKNDFQRLYTNDREDYLGKLERDITKFFVDRGFLEIKSPILIPAEYVERMGIN
NDTELSKQIFRVDKNLCLRPMLAPTLANYLRKLDRILPGPIKIFEVGPCYRKESDGKEHLEEFTMVQFSQ
MGSGCTRENLEALIKEFLDYLEIDFEIVGDSCMVWGDTLDIMHGDLELSSAVVGPVSLDREWGIDKPWI
GAGFGLERLLKVMHGFKNIKRASRSESYYNGISTNL

HA-SUMO1(ΔG)-MxeGyrA(N198A)-CBD

MAYPYDVPDYAGSMSDQEAKPSTEDLGDKKEGEYIKLKVIGQDSSEIHFKVKMTTHLKKLKESYCQRQ
GVPMNSLRFLFEGQRIADNHTPKELGMEEEDVIEVYQEQTGCITGDALVALPEGESVRIADIVPGARPNS
DNAIDLKVLDRHGNPVLADRLFHSGEHPVYTVRTVEGLRVTGTANHPLLCLVDVAGVPTLLWKLIDEIK
PGDYAVIQRSAFSVDCAGFARGKPEFAPTTYTVGVPGLVRFLEAHHRDPDAQAIADELTDGRFYYAKVA
SVTDAGVQPVYSLRVDTADHAFITNGFVSHATGLTGLNSGLTTNPGVSAWQVNTAYTAGQLVTYNGKT
YKCLQPHTSLAGWEPSNVPALWQLQ

HA-SUMO2(ΔG)-MxeGyrA(N198A)-CBD

MAYPYDVPDYAGSMADEKPKEGVKTENNDHINLKVAGQDGSVVQFKIKRHTPLSKLMKAYCERQGLS
MRQIRFRFDGQPINETDTPAQLEMEDEDTIDVFQQQTGCITGDALVALPEGESVRIADIVPGARPNSDNAI
DLKVLDRHGNPVLADRLFHSGEHPVYTVRTVEGLRVTGTANHPLLCLVDVAGVPTLLWKLIDEIKPGD
YAVIQRSAFSVDCAGFARGKPEFAPTTYTVGVPGLVRFLEAHHRDPDAQAIADELTDGRFYYAKVASVT
DAGVQPVYSLRVDTADHAFITNGFVSHATGLTGLNSGLTTNPGVSAWQVNTAYTAGQLVTYNGKTYKC
LQPHTSLAGWEPSNVPALWQLQ

sfGFP(V2P, N150TAG)-H6

MPSKGEELFTGVVPILVELDGDVNGHKFSVRGEGEGDATNGKLTLKFICTTGKLPVPWPTLVTTLTYGV
QCFSRYPDHMKRHDFFKSAMPEGYVQERTISFKDDGTYKTRAEVKFEGDTLVNRIELKGIDFKEDGNIL
GHKLEYNFNSH*VYITADKQKNGIKANFKIRHNVEDGSVQLADHYQQNTPIGDGPVLLPDNHYLSTQS
VLSKDPNEKRDHMVLLEFVTAAGITHGMDELYKGSHHHHHH

Ub(K11TAG)-H6

MQIFVKTLTG*TITLEVEPSDTIENVKAKIQDKEGIPPDQQRLIFAGKQLEDGRTLSDYNIQKESTLHLVLR
LRGGHHHHHH

SUMO2(K11TAG)-H6

MADEKPKEGV*TENNDHINLKVAGQDGSVVQFKIKRHTPLSKLMKAYCERQGLSMRQIRFRFDGQPIN
ETDTPAQLEMEDEDTIDVFQQQTGGHHHHHH

SUMO2(K11TAG, ΔG)-H6

MADEKPKEGV*TENNDHINLKVAGQDGSVVQFKIKRHTPLSKLMKAYCERQGLSMRQIRFRFDGQPIN
ETDTPAQLEMEDEDTIDVFQQQTGHHHHHH

PCNA-H6

MFEARLVQGSILKKVLEALKDLINEACWDISSSGVNLQSMDSSHVSLVQLTLRSEGFDTYRCDRNLAM
GVNLTSMSKILKCAGNEDIITLRAEDNADTLALVFEAPNQEKVSDYEMKLMDLDVEQLGIPEQEYSCV
VKMPSGEFARICRDLSHIGDAVVISCAKDGVKFSASGELGNGNIKLSQTSNVDKEEEAVTIEMNEPVQLT
FALRYLNFFTKATPLSSTVTLSMSADVPLVVEYKIADMGHLKYYLAPKIEDEEGSHHHHHH

PCNA(K164TAG)-H6

MFEARLVQGSILKKVLEALKDLINEACWDISSSGVNLQSMDSSHVSLVQLTLRSEGFDTYRCDRNLAM
GVNLTSMSKILKCAGNEDIITLRAEDNADTLALVFEAPNQEKVSDYEMKLMDLDVEQLGIPEQEYSCV
VKMPSGEFARICRDLSHIGDAVVISCA*DGVKFSASGELGNGNIKLSQTSNVDKEEEAVTIEMNEPVQLT
FALRYLNFFTKATPLSSTVTLSMSADVPLVVEYKIADMGHLKYYLAPKIEDEEGSHHHHHH

***Mb*PylRS:**

MDKKPLDVLISATGLWMSRTGTLHKIKHHEVSRSKIYIEMACGDHLVVNNSRSCRTARAFRHHKYRKT
CKRCRVSDEDINNFLTRSTESKNSVKVRVVSAPKVKKAMPKSVSRAPKPLENSVSAKASTNTSRSVPSP
AKSTPNSSVPASAPAPSLTRSQLDRVEALLSPEDKISLNMAKPFRELEPELVTRRKNDFQRLYTNDREDY
LGKLERDITKFFVDRGFLEIKSPILIPAEYVERMGINNDTELSKQIFRVDKNLCLRPMLAPTLYNYLRKLD
RILPGPIKIFEVGPCYRKESDGKEHLEEFTMVNFCQMGSGCTRENLEALIKEFLDYLEIDFEIVGDSCMV
YGDTLDIMHGDLELSSAVVGPVSLDREWGIDKPWIGAGFGLERLLKVMHGFKNIKRASRSESYYNGIST
NL

***Mb*AzGGisoK-RS (mutations from wt PylRS: L274A, N311Q, C313S):**

MDKKPLDVLISATGLWMSRTGTLHKIKHHEVSRSKIYIEMACGDHLVVNNSRSCRTARAFRHHKYRKT
CKRCRVSDEDINNFLTRSTESKNSVKVRVVSAPKVKKAMPKSVSRAPKPLENSVSAKASTNTSRSVPSP
AKSTPNSSVPASAPAPSLTRSQLDRVEALLSPEDKISLNMAKPFRELEPELVTRRKNDFQRLYTNDREDY
LGKLERDITKFFVDRGFLEIKSPILIPAEYVERMGINNDTELSKQIFRVDKNLCLRPMLAPTLYNYARKLD
RILPGPIKIFEVGPCYRKESDGKEHLEEFTMVQFSQMGSGCTRENLEALIKEFLDYLEIDFEIVGDSCMVY
GDTLDIMHGDLELSSAVVGPVSLDREWGIDKPWIGAGFGLERLLKVMHGFKNIKRASRSESYYNGISTN
L

***Mb*PylRS variant L274A, N311Q, C313V:**

MDKKPLDVLISATGLWMSRTGTLHKIKHHEVSRSKIYIEMACGDHLVVNNSRSCRTARAFRHHKYRKT
CKRCRVSDEDINNFLTRSTESKNSVKVRVVSAPKVKKAMPKSVSRAPKPLENSVSAKASTNTSRSVPSP
AKSTPNSSVPASAPAPSLTRSQLDRVEALLSPEDKISLNMAKPFRELEPELVTRRKNDFQRLYTNDREDY
LGKLERDITKFFVDRGFLEIKSPILIPAEYVERMGINNDTELSKQIFRVDKNLCLRPMLAPTLYNYARKLD
RILPGPIKIFEVGPCYRKESDGKEHLEEFTMVQFVQMGSGCTRENLEALIKEFLDYLEIDFEIVGDSCMV
YGDTLDIMHGDLELSSAVVGPVSLDREWGIDKPWIGAGFGLERLLKVMHGFKNIKRASRSESYYNGIST
NL

***Mb*PylRS variant L274A, N311Q, C313V, M315Y:**

MDKKPLDVLISATGLWMSRTGTLHKIKHHEVSRSKIYIEMACGDHLVVNNSRSCRTARAFRHHKYRKT
CKRCRVSDEDINNFLTRSTESKNSVKVRVVSAPKVKKAMPKSVSRAPKPLENSVSAKASTNTSRSVPSP
AKSTPNSSVPASAPAPSLTRSQLDRVEALLSPEDKISLNMAKPFRELEPELVTRRKNDFQRLYTNDREDY
LGKLERDITKFFVDRGFLEIKSPILIPAEYVERMGINNDTELSKQIFRVDKNLCLRPMLAPTLYNYARKLD
RILPGPIKIFEVGPCYRKESDGKEHLEEFTMVQFVQYGSGCTRENLEALIKEFLDYLEIDFEIVGDSCMVY
GDTLDIMHGDLELSSAVVGPVSLDREWGIDKPWIGAGFGLERLLKVMHGFKNIKRASRSESYYNGISTN
L

***Mm*AzGGisoK-RS (mutations from wt PylRS: L309A, N346Q, C348S):**

MDKKPLNTLISATGLWMSRTGTIHKIKHHEVSRSKIYIEMACGDHLVVNNSRSSRTARALRHHKYRKTC
KRCRVSDEDLNKFLTKANEDQTSVKVKVVSAPTRTKKAMPKSVARAPKPLENTEAAQAQPSGSKFSPAI
PVSTQESVSVPASVSTSISSISTGATASALVKGNTNPITSMSAPVQASAPALTKSQTDRLEVLLNPKDEISL
NSGKPFRELESELLSRRKKDLQQIYAEERENYLGKLEREITRFFVDRGFLEIKSPILIPLEYIERMGIDNDT
ELSKQIFRVDKNFCLRPMLAPNLYNYARKLDRALPDPIKIFEIGPCYRKESDGKEHLEEFTMLQFSQMGS
GCTRENLESIITDFLNHLGIDFKIVGDSCMVYGDTLDVMHGDLELSSAVVGPIPLDREWGIDKPWIGAGF
GLERLLKVKHDFKNIKRAARSESYYNGISTNL

***Mm*AzGGisoK-RS (P188G) (mutations from wt PylRS: P188G, L309A, N346Q, C348S):**

MDKKPLNTLISATGLWMSRTGTIHKIKHHEVSRSKIYIEMACGDHLVVNNSRSSRTARALRHHKYRKTC
KRCRVSDEDLNKFLTKANEDQTSVKVKVVSAPTRTKKAMPKSVARAPKPLENTEAAQAQPSGSKFSPAI
PVSTQESVSVPASVSTSISSISTGATASALVKGNTNPITSMSAPVQASAGALTKSQTDRLEVLLNPKDEISL
NSGKPFRELESELLSRRKKDLQQIYAEERENYLGKLEREITRFFVDRGFLEIKSPILIPLEYIERMGIDNDT
ELSKQIFRVDKNFCLRPMLAPNLYNYARKLDRALPDPIKIFEIGPCYRKESDGKEHLEEFTMLQFSQMGS
GCTRENLESIITDFLNHLGIDFKIVGDSCMVYGDTLDVMHGDLELSSAVVGPIPLDREWGIDKPWIGAGF
GLERLLKVKHDFKNIKRAARSESYYNGISTNL

***Ma*AzGGisoK-RS(IP) (mutations from wt PylRS: M129A, N166Q, V168S, H227I, Y228P):**

MMTVKYTDAQIQRLREYGNGTYEQKVFEDLASRDAAFSKEMSVASTDNEKKIKGMIANPSRHGLTQL
MNDIADALVAEGFIEVRTPIFISKDALARMTITEDKPLFKQVFWIDEKRALRPMLAPNLYSVARDLRDHT
DGPVKIFEMGSCFRKESHSGMHLEEFTMLQLSDMGPRGDATEVLKNYISVVMKAAGLPDYDLVQEES

DVYKETIDVEINGQEVCSAAVGPIPLDAAHDVHEPWSGAGFGLERLLTIREKYSTVKKGGASISYLNGA
KIN

***Mb*AzGVAisoK-RS (mutations from wt PylRS: Y271A, N311Q, C313S, Y349W):**
MMDKKPLDVLISATGLWMSRTGTLHKIKHHEVSRSKIYIEMACGDHLVVNNSRSCRTARAFRHHKYRK
TCKRCRVSDEDINNFLTRSTESKNSVKVRVVSAPKVKKAMPKSVSRAPKPLENSVSAKASTNTSRSVPS
PAKSTPNSSVPASAPAPSLTRSQLDRVEALLSPEDKISLNMAKPFRELEPELVTRRKNDFQRLYTNDREDY
LGKLERDITKFFVDRGFLEIKSPILIPAEYVERMGINNDTELSKQIFRVDKNLCLRPMLAPTLANYLRKLD
RILPGPIKIFEVGPCYRKESDGKEHLEEFTMVQFSQMGSGCTRENLEALIKEFLDYLEIDFEIVGDSCMV
WGDTLDIMHGDLELSSAVVGPVSLDREWGIDKPWIGAGFGLERLLKVMHGFKNIKRASRSESYYNGIS
TNL

***Ma*AzGVAisoK-RS (mutations from wt PylRS: Y126A, N166Q, V168S, Y206W):**
MMTVKYTDAQIQRLREYGNGTYEQKVFEDLASRDAAFSKEMSVASTDNEKKIKGMIANPSRHGLTQL
MNDIADALVAEGFIEVRTPIFISKDALARMTITEDKPLFKQVFWIDEKRALRPMLAPNLASVMRDLRDH
TDGPVKIFEMGSCFRKESHSGMHLEEFTMLQLSDMGPRGDATEVLKNYISVVMKAAGLPDYDLVQEES
DVWKETIDVEINGQEVCSAAVGPHYLDAAHDVHEPWSGAGFGLERLLTIREKYSTVKKGGASISYLNG
AKIN

***Ma*AzGGisoK-RS(IP) (mutations from wt PylRS: Y126A, N166Q, V168S, Y206W, H227I, Y228P):**
MMTVKYTDAQIQRLREYGNGTYEQKVFEDLASRDAAFSKEMSVASTDNEKKIKGMIANPSRHGLTQL
MNDIADALVAEGFIEVRTPIFISKDALARMTITEDKPLFKQVFWIDEKRALRPMLAPNLASVMRDLRDH
TDGPVKIFEMGSCFRKESHSGMHLEEFTMLQLSDMGPRGDATEVLKNYISVVMKAAGLPDYDLVQEES
DVWKETIDVEINGQEVCSAAVGPIPLDAAHDVHEPWSGAGFGLERLLTIREKYSTVKKGGASISYLNGA
KIN

CAT(111TAG):
MEKKITGYTTVDISQWHRKEHFEAFQSVAQCTYNQTVQLDITAFLKTVKKNKHKFYPAFIHILARLMNA
HPEFRMAMKDGELVIWDSVHPCYTVFHEQTETFSSLWSEYH*DFRQFLHIYSQDVACYGENLAYFPKGF
IENMFFVSANPWVSFTSFDLNVANMDNFFAPVFTMGKYYTQGDKVLMPLAIQVHHAVCDGFHVGRML
NELQQYCDEWQGGA

barnase(3TAG, 45TAG):
MA*VINTFDGVADYLQTYHKLPDNYITKSEAQALGWVASKGNLA*VAPGKSIGGDIFSNREGKLPGKSG
RTWREADINYTSGFRNSDRILYSSDWLIYKTTDHYQTFTKIR

FLAG-SENP1(CD: 419-644):
MDYKDHDGDYKDHDIDYKDDDDKSGGEFPEITEEMEKEIKNVFRNGNQDEVLSEAFRLTITRKDIQTL
NHLNWLNDEIINFYMNMLMERSKEKGLPSVHAFNTFFFTKLKTAGYQAVKRWTKKVDVFSVDILLVPI

HLGVHWCLAVVDFRKKNITYYDSMGGINNEACRILLQYLKQESIDKKRKEFDTNGWQLFSKKSQEIPQ
QMNGSDCGMFACKYADCITKDRPINFTQQHMPYFRKRMVWEILHRKLL

FLAG-SENP1(CD: 419-644, C603A):
MDYKDHDGDYKDHDIDYKDDDDKSGGEFPEITEEMEKEIKNVFRNGNQDEVLSEAFRLTITRKDIQTL
NHLNWLNDEIINFYMNMLMERSKEKGLPSVHAFNTFFFTKLKTAGYQAVKRWTKKVDVFSVDILLVPI
HLGVHWCLAVVDFRKKNITYYDSMGGINNEACRILLQYLKQESIDKKRKEFDTNGWQLFSKKSQEIPQ
QMNGSDAGMFACKYADCITKDRPINFTQQHMPYFRKRMVWEILHRKLL

FLAG-SENP1(FL) (additional mutations I194F, T206A, T623A):
MDYKDDDDKLAAANSSIDLMDDIADRMRMDAGEVTLVNHNSVFKTHLLPQTGFPEDQLSLSDQQILS
SRQGHLDRSFTCSTRSAAYNPSYYSDNPSSDSFLGSGDLRTFGQSANGQWRNSTPSSSSSLQKSRNSRSL
YLETRKTSSGLSNSFAGKSNHHCHVSAYEKSFPIKPVPSPSWSGSCRRSLLSPKKTQRRHVSTAEETVQE
EEREFYRQLLQMVTGKQFAIAKPTTHFPLHLSRCLSSSKNTLKDSLFKNGSSCASQIIGSDTSSSGSASILT
NQEQLSHSVYSLSSYTPDVAFGSKDSGTLHHPHHHHSVPHQPDNLAASNTQSEGSDSVILLKVKDSQTP
TPSSTFFQAELWIKELTSVYDSRARERLRQIEEQKALALQLQNQRLQEREHSVHDSVELHLRVPLEKEIP
VTVVQETQKKGHKLTDSEDEFPEITEEMEKEIKNVFRNGNQDEVLSEAFRLTITRKDIQTLNHLNWLND
EIINFYMNMLMERSKEKGLPSVHAFNTFFFTKLKTAGYQAVKRWTKKVDVFSVDILLVPIHLGVHWCL
AVVDFRKKNITYYDSMGGINNEACRILLQYLKQESIDKKRKEFDTNGWQLFSKKSQEIPQQMNGSDCG
MFACKYADCITKDRPINFAQQHMPYFRKRMVWEILHRKLL

FLAG-SENP1(FL, C603A) (additional mutations I194F, T206A, T623A):
MDYKDDDDKLAAANSSIDLMDDIADRMRMDAGEVTLVNHNSVFKTHLLPQTGFPEDQLSLSDQQILS
SRQGHLDRSFTCSTRSAAYNPSYYSDNPSSDSFLGSGDLRTFGQSANGQWRNSTPSSSSSLQKSRNSRSL
YLETRKTSSGLSNSFAGKSNHHCHVSAYEKSFPIKPVPSPSWSGSCRRSLLSPKKTQRRHVSTAEETVQE
EEREFYRQLLQMVTGKQFAIAKPTTHFPLHLSRCLSSSKNTLKDSLFKNGSSCASQIIGSDTSSSGSASILT
NQEQLSHSVYSLSSYTPDVAFGSKDSGTLHHPHHHHSVPHQPDNLAASNTQSEGSDSVILLKVKDSQTP
TPSSTFFQAELWIKELTSVYDSRARERLRQIEEQKALALQLQNQRLQEREHSVHDSVELHLRVPLEKEIP
VTVVQETQKKGHKLTDSEDEFPEITEEMEKEIKNVFRNGNQDEVLSEAFRLTITRKDIQTLNHLNWLND
EIINFYMNMLMERSKEKGLPSVHAFNTFFFTKLKTAGYQAVKRWTKKVDVFSVDILLVPIHLGVHWCL
AVVDFRKKNITYYDSMGGINNEACRILLQYLKQESIDKKRKEFDTNGWQLFSKKSQEIPQQMNGSDAG
MFACKYADCITKDRPINFAQQHMPYFRKRMVWEILHRKLL

FLAG-SENP2(CD: 364-589):
MDYKDHDGDYKDHDIDYKDDDDKSGGDLLELTEDMEKEISNALGHGPQDEILSSAFKLRITRGDIQTL
KNYHWLNDEVINFYMNLLVERNKKQGYPALHVFSTFFYPKLKSGGYQAVKRWTKGVNLFEQEIILVPI
HRKVHWSLVVIDLRKKCLKYLDSMGQKGHRICEILLQYLQDESKTKRNSDLNLLEWTHHSMKPHEIPQ
QLNGSDCGMFTCKYADYISRDKPITFTQHQMPLFRKKMVWEILHQQLL

HA-SUMO2(QLA)^{x}-T2AP2A-Srt2A-Myc

MAYPYDVPDYAGSMADEKPKEGVKTENNDHINLKVAGQDGSVVQFKIKRHTPLSKLMKAYCERQGLS
MRQIRFRFDGQPINETDTPAQLEMEDEDTIDVFQLAQTGYEAAAKGSGEGRGSLLTCGDVEENPGPGSG
ATNFSLLKQAGDVEENPGPMAQAKPQIPKDKSKVAGYIEIPDADIKEPVYPGPATREQLNRGVCFHDEN
ESLDDQNISIAGHTFIDRPNYQFTNLKAAKPGSMVYFKVGNETRIYKMTSIRKVHPNAVEVLDEQEGKD
KQLTLVTCDDYNEETGVWESRKIFVATEVKGSAGSGEQKLISEEDL

4. NMR Spectra and HRMS

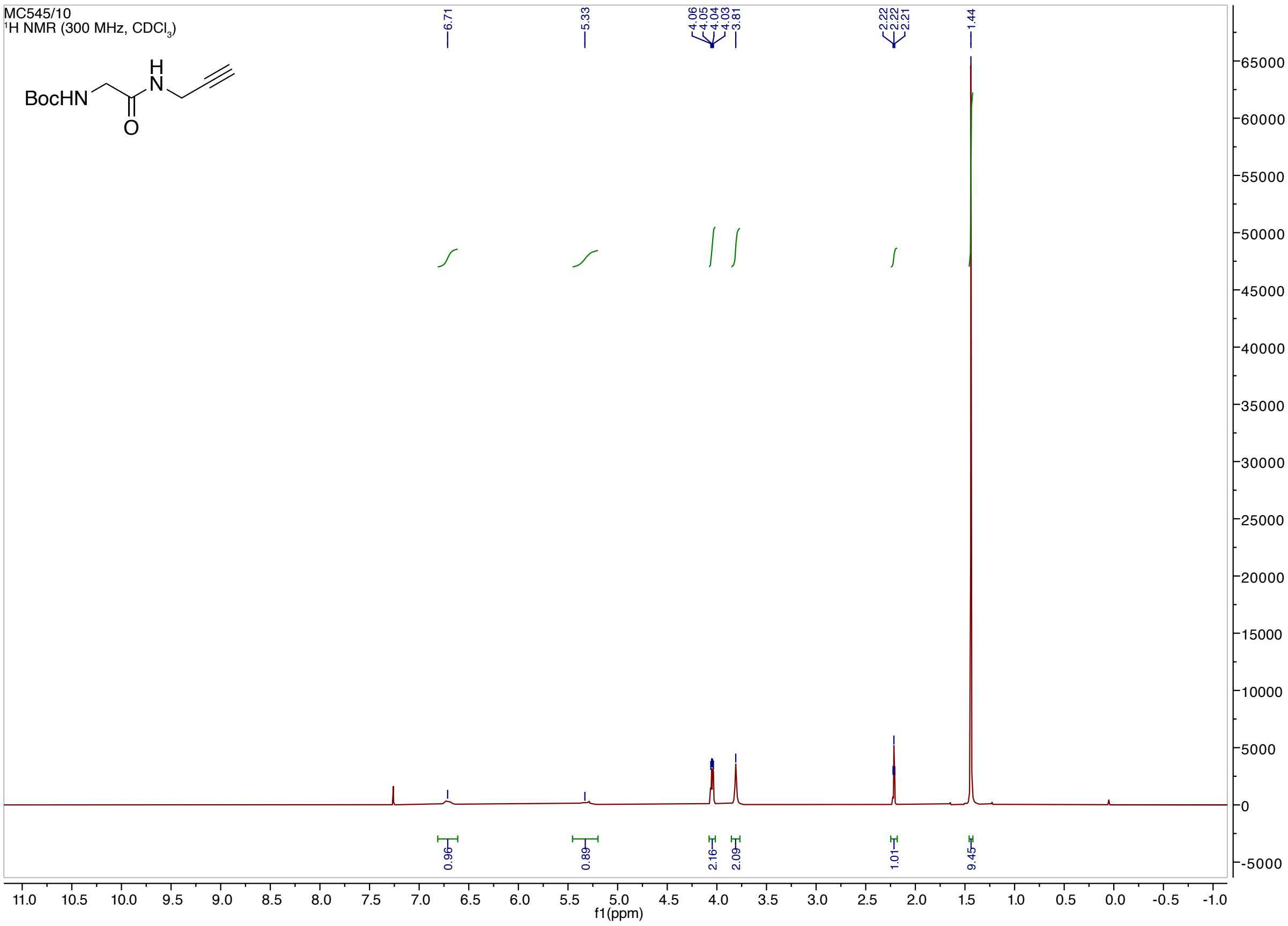

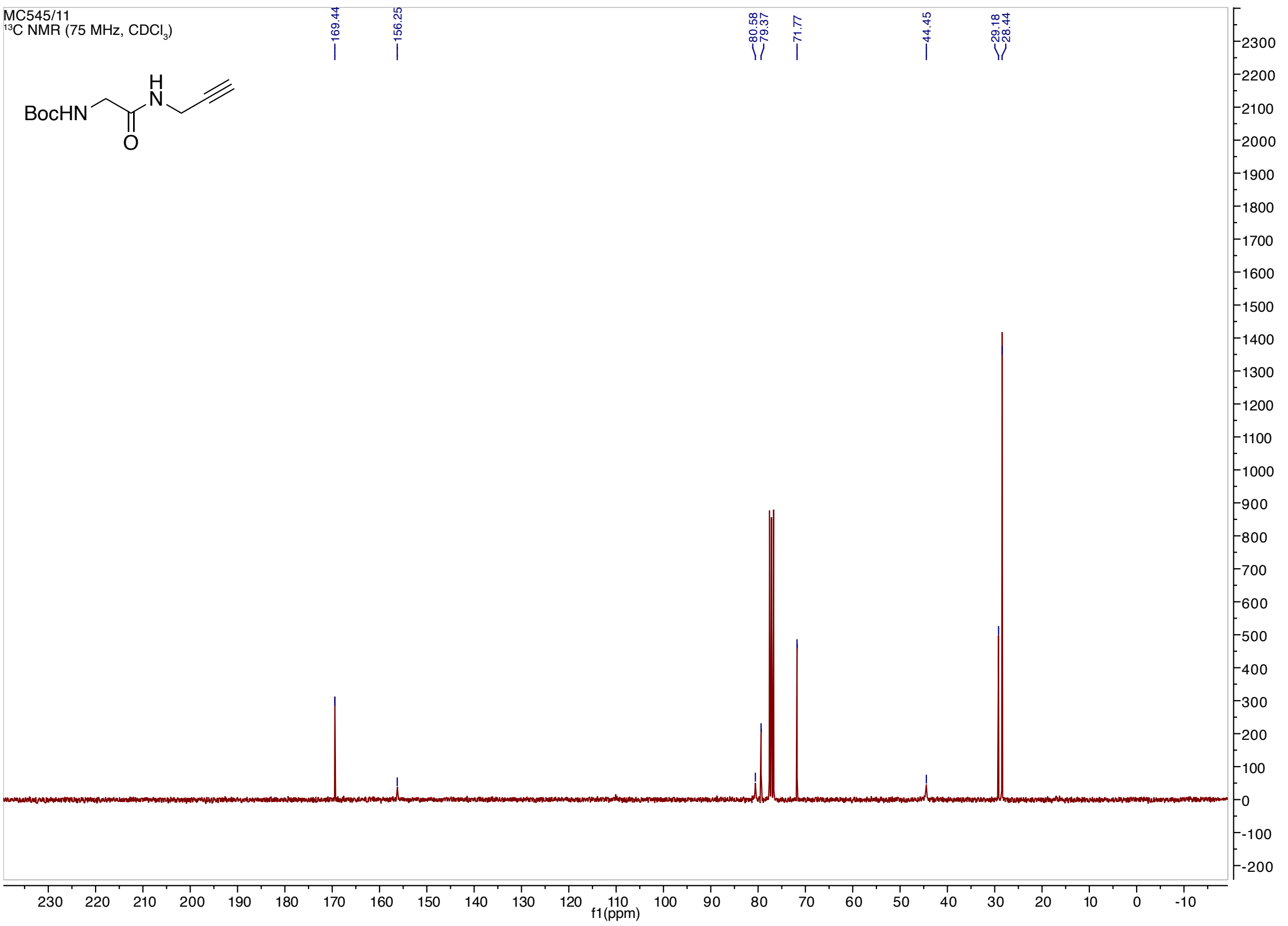

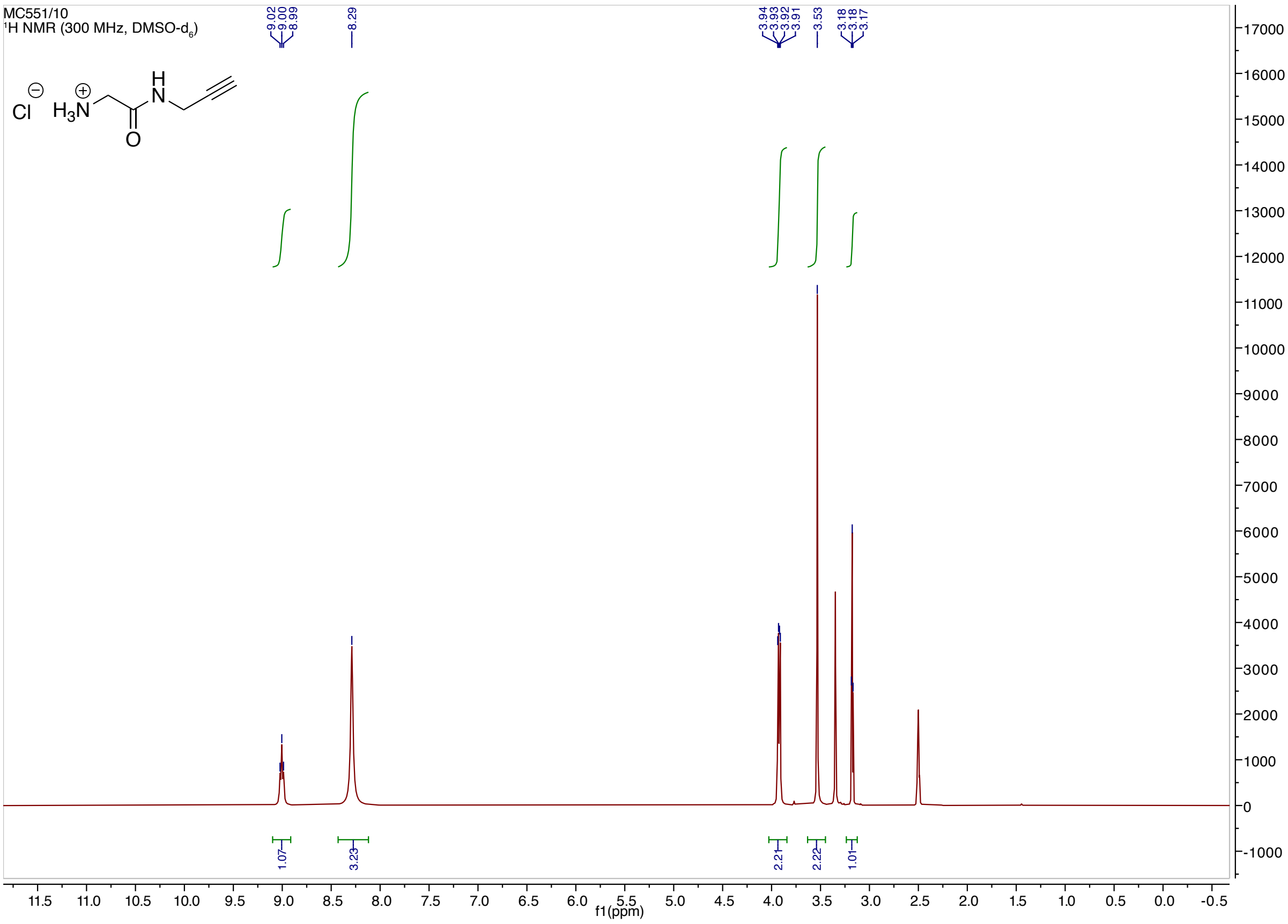
MC551/10
1H NMR (300 MHz, DMSO-d6)
9.02
9.00
8.99
8.29
3.94
3.93
3.92
3.91
3.53
3.18
3.18
3.17
1.07
3.23
2.21
2.22
1.01
f1(ppm)

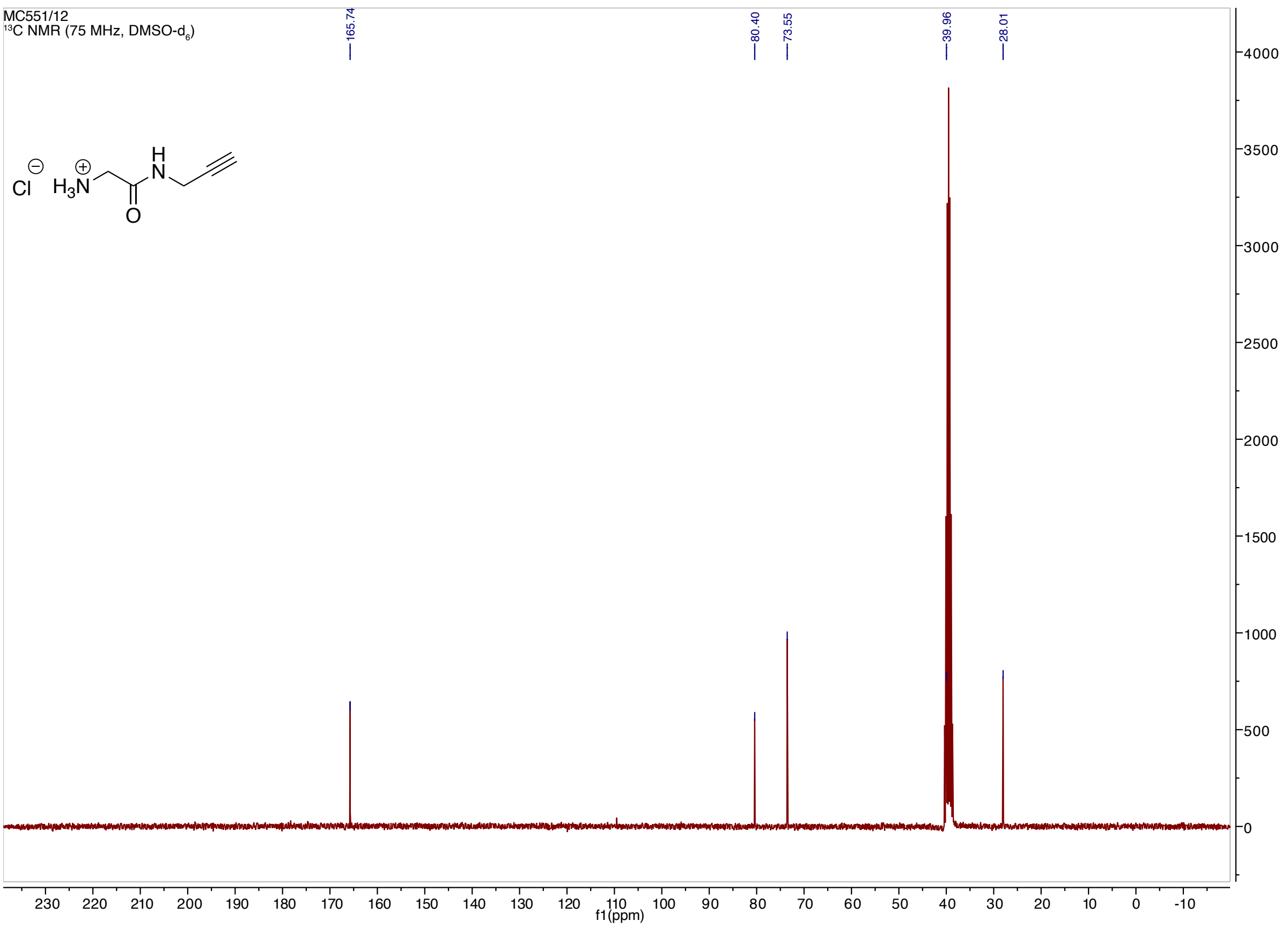
MC551/12
13C NMR (75 MHz, DMSO-d6)
165.74
80.40
73.55
39.96
28.01
f1(ppm)

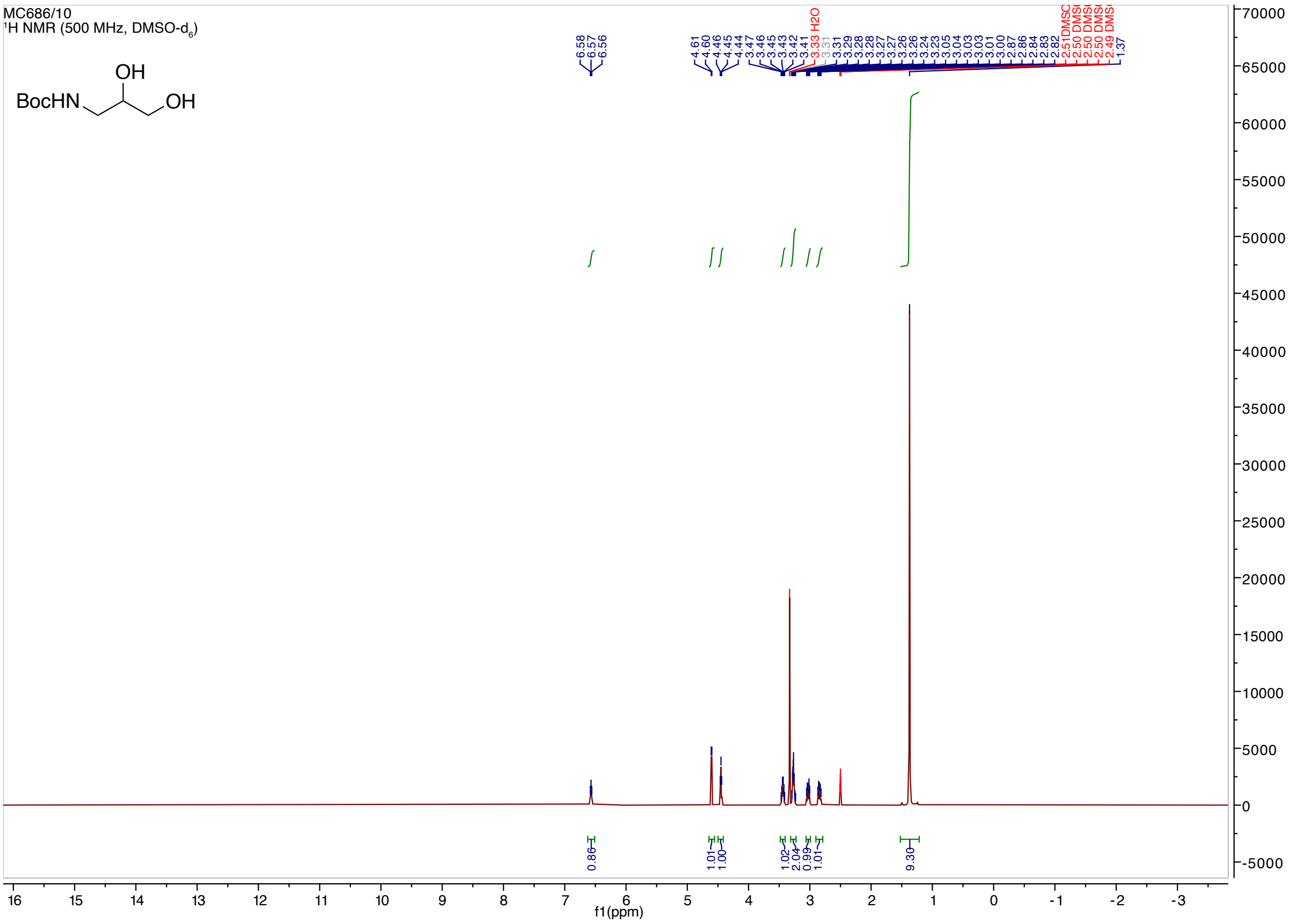
MC686/10
¹H NMR (500 MHz, DMSO-d6)
BocHN OH OH
f1 (ppm)

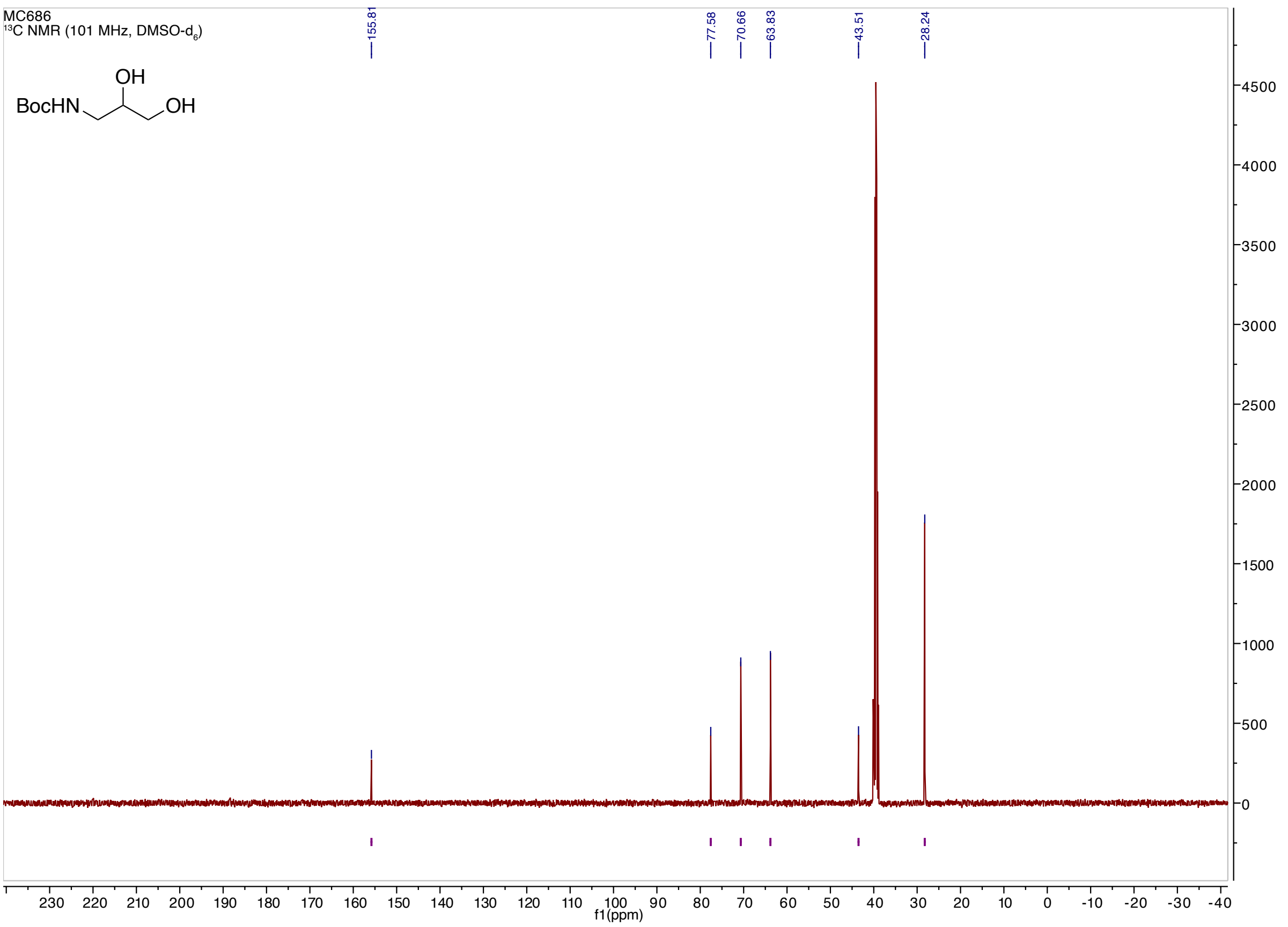
MC686
¹³C NMR (101 MHz, DMSO-d6)
BocHN OH OH
155.81
77.58
70.66
63.83
43.51
28.24
f1 (ppm)

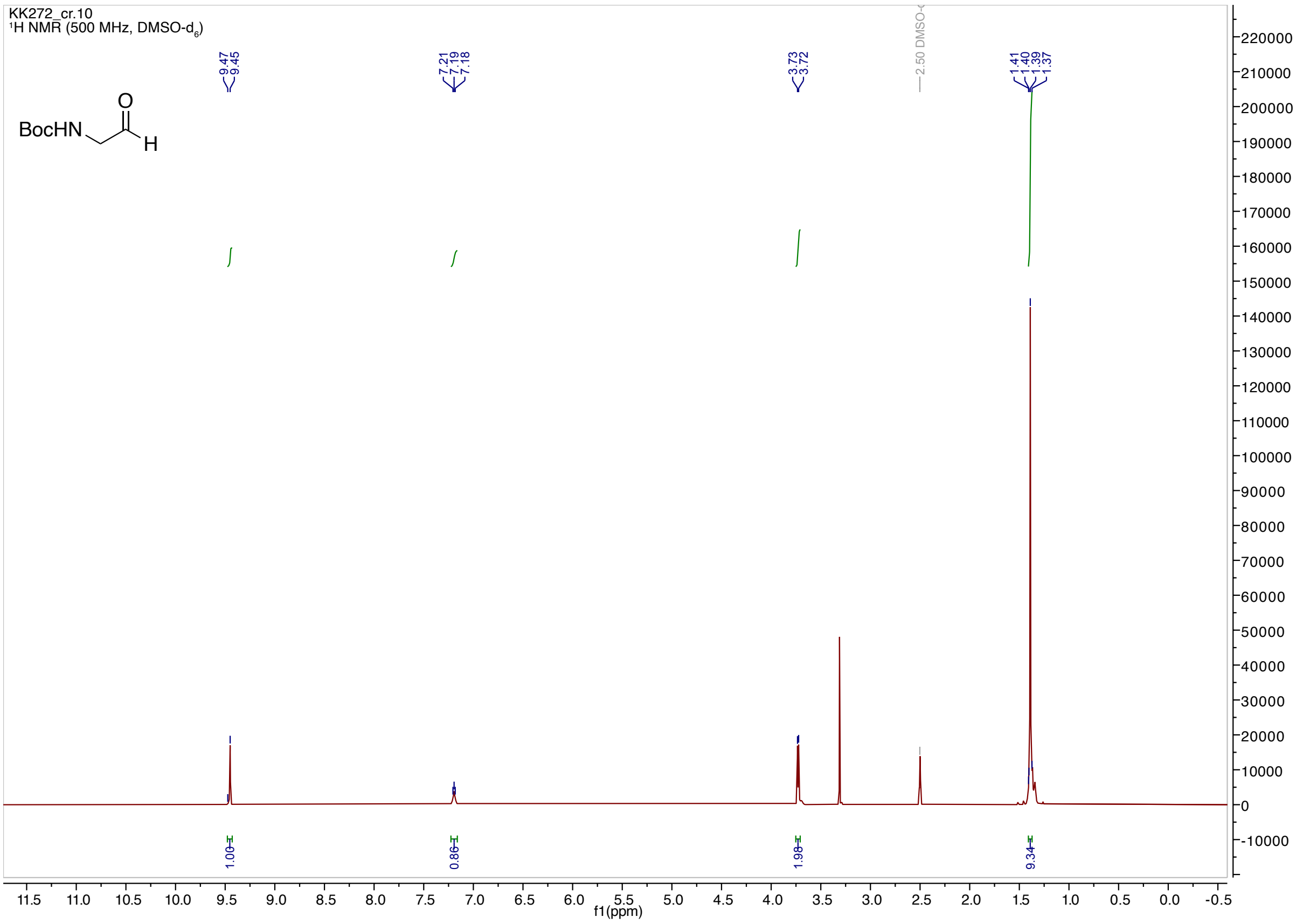
KK272_cr.10
¹H NMR (500 MHz, DMSO-d6)
BocHN
O
H
f1(ppm)

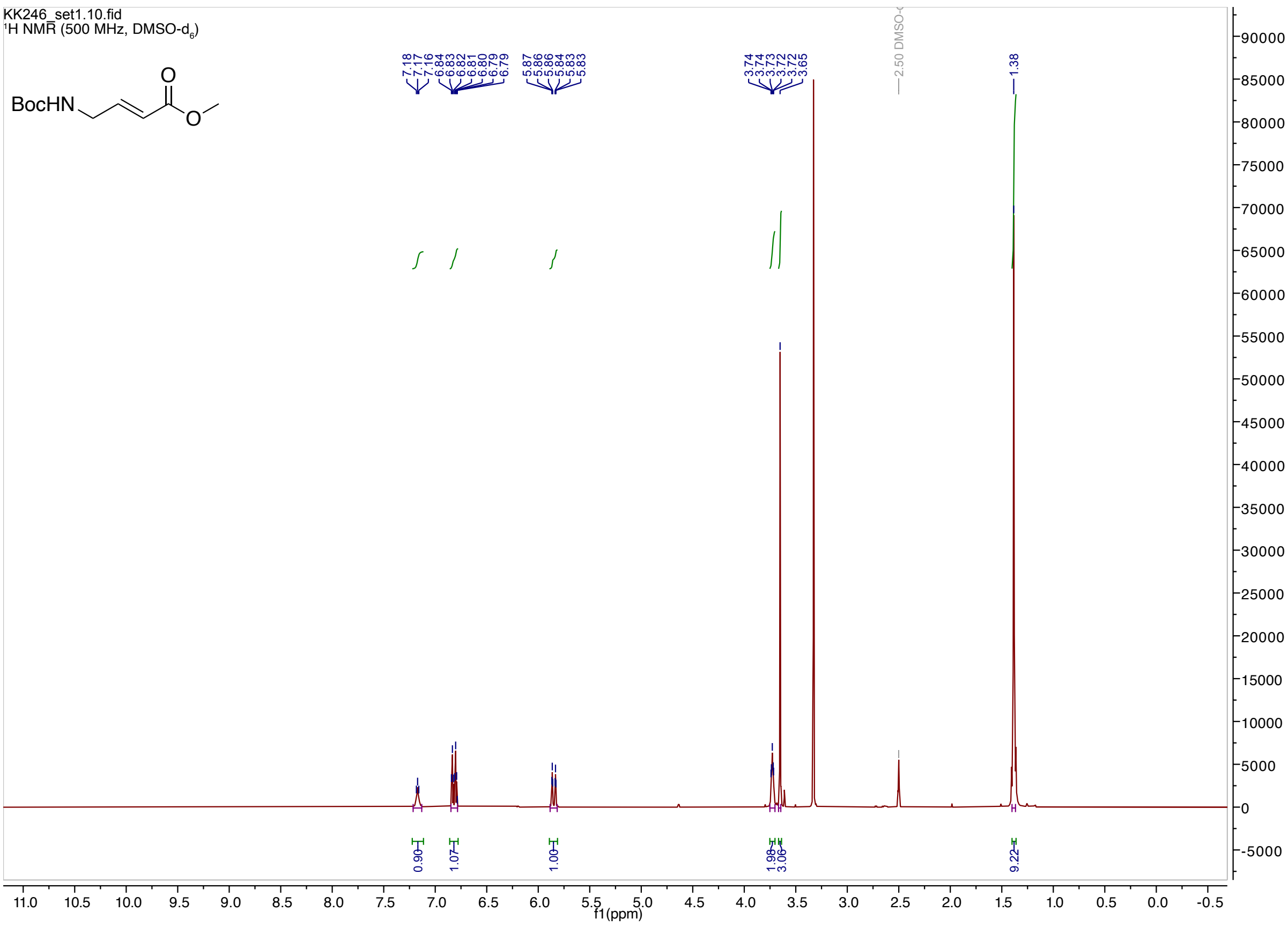
KK246_set1.10.fid
¹H NMR (500 MHz, DMSO-d6)
BocHN
O
O
f1(ppm)

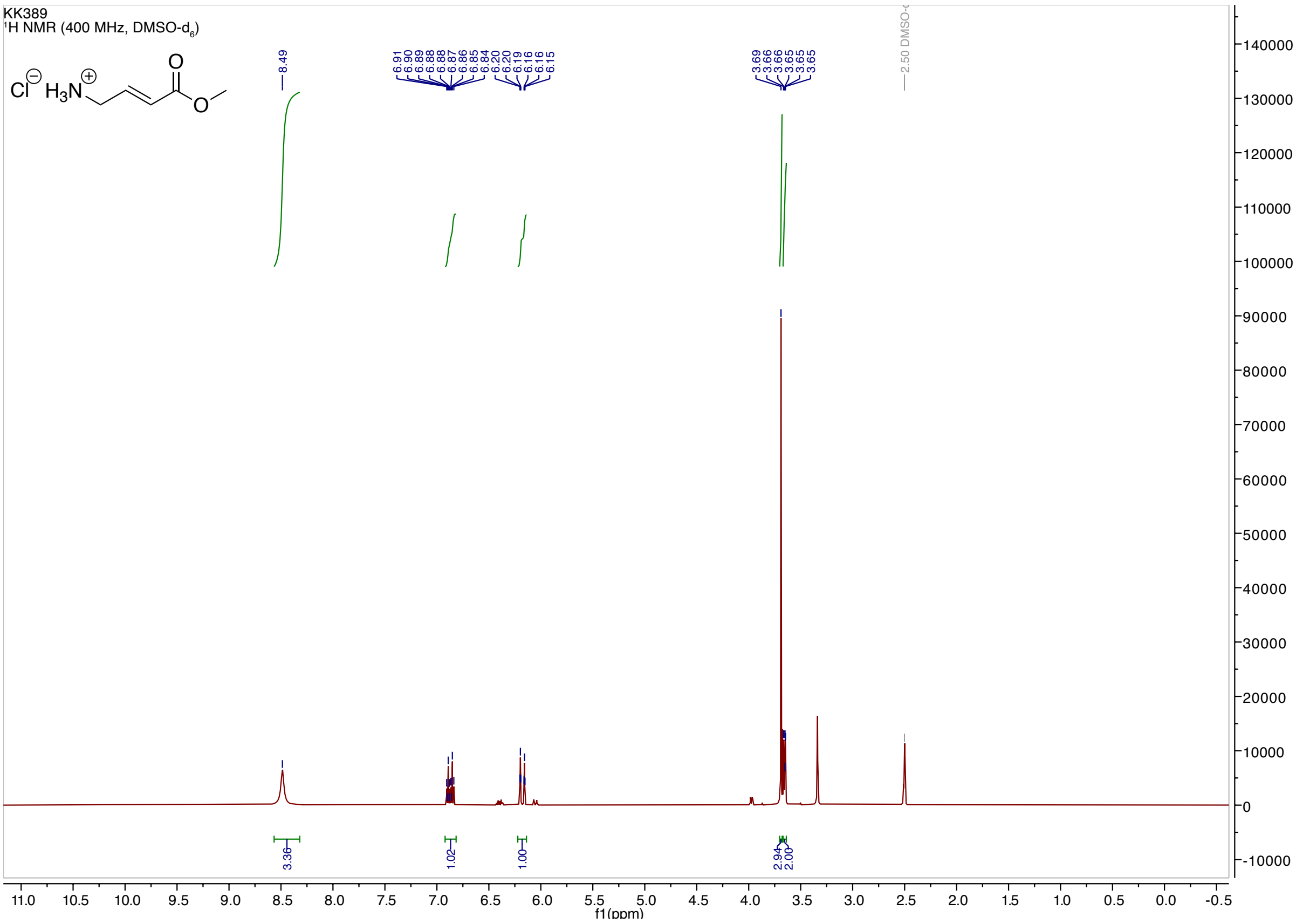
KK389
¹H NMR (400 MHz, DMSO-d6)
f1 (ppm)

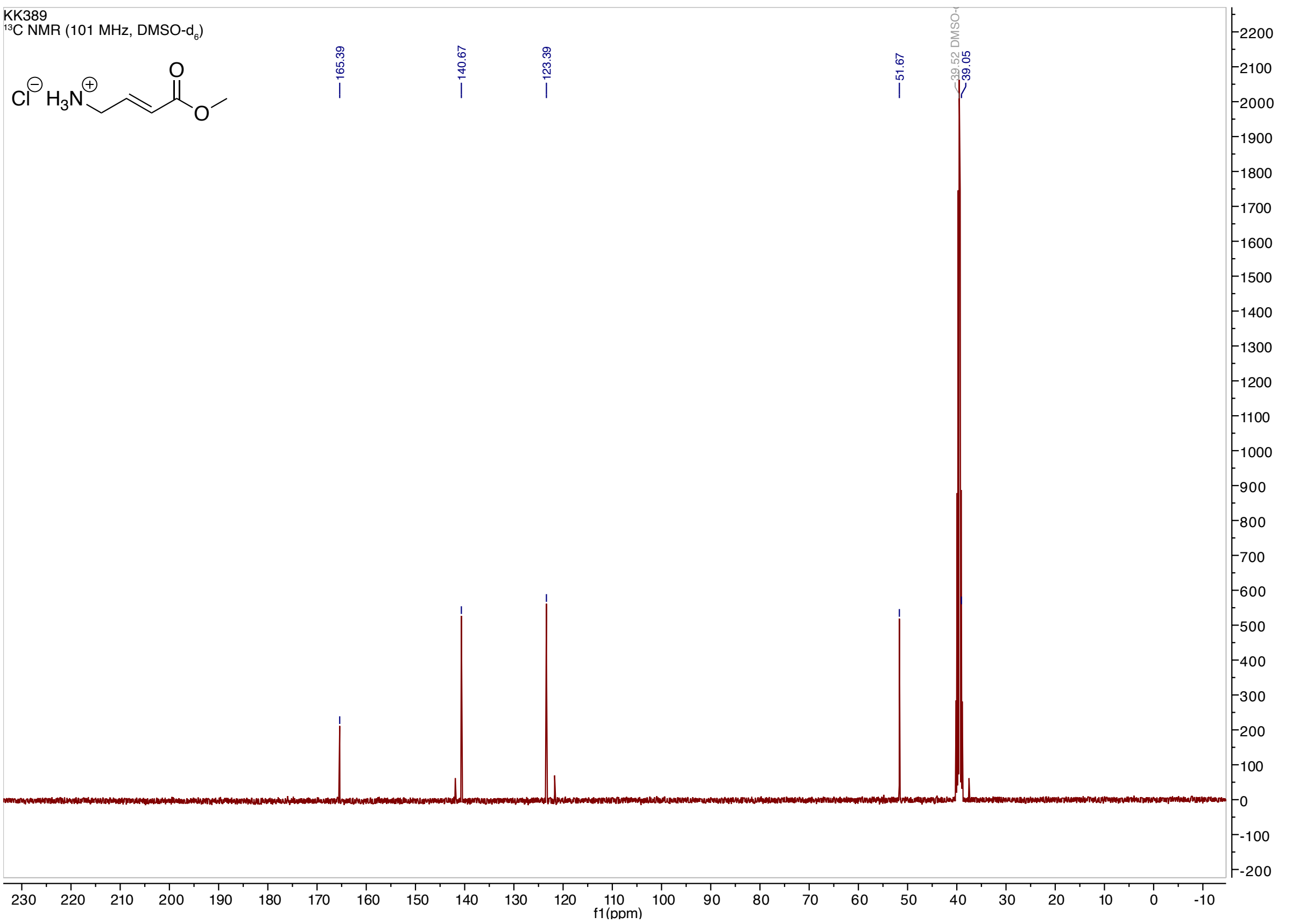
KK389
¹³C NMR (101 MHz, DMSO-d6)
f1 (ppm)

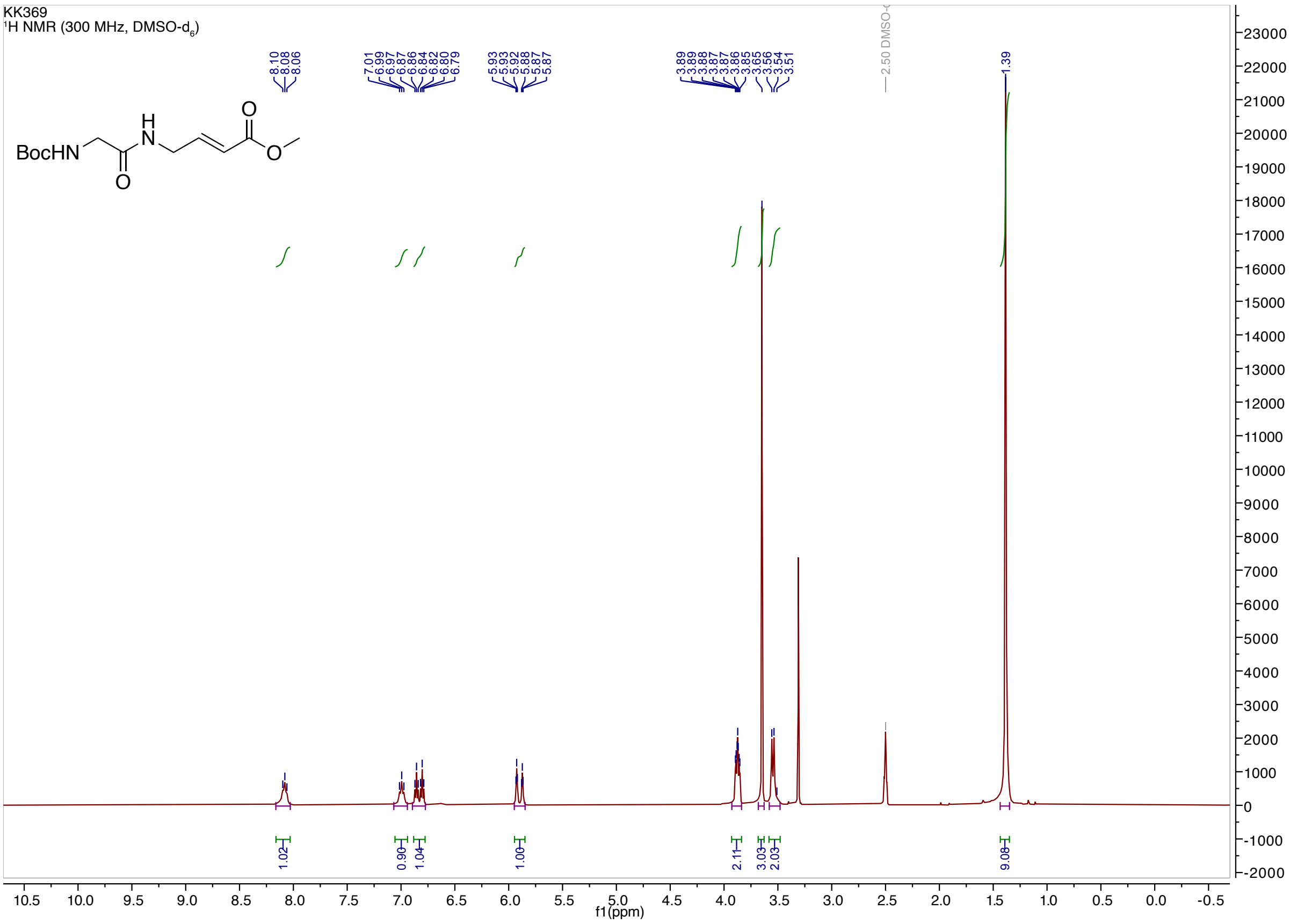
KK369
1H NMR (300 MHz, DMSO-d6)
BocHN
H
N
O
O
O
8.10
8.08
8.06
7.01
6.99
6.97
6.87
6.86
6.84
6.82
6.80
6.79
5.93
5.93
5.92
5.88
5.87
5.87
3.89
3.89
3.88
3.87
3.87
3.86
3.85
3.65
3.56
3.54
3.51
2.50 DMSO-d
1.39
1.02
0.90
1.04
1.00
2.11
3.03
2.03
9.08
f1 (ppm)

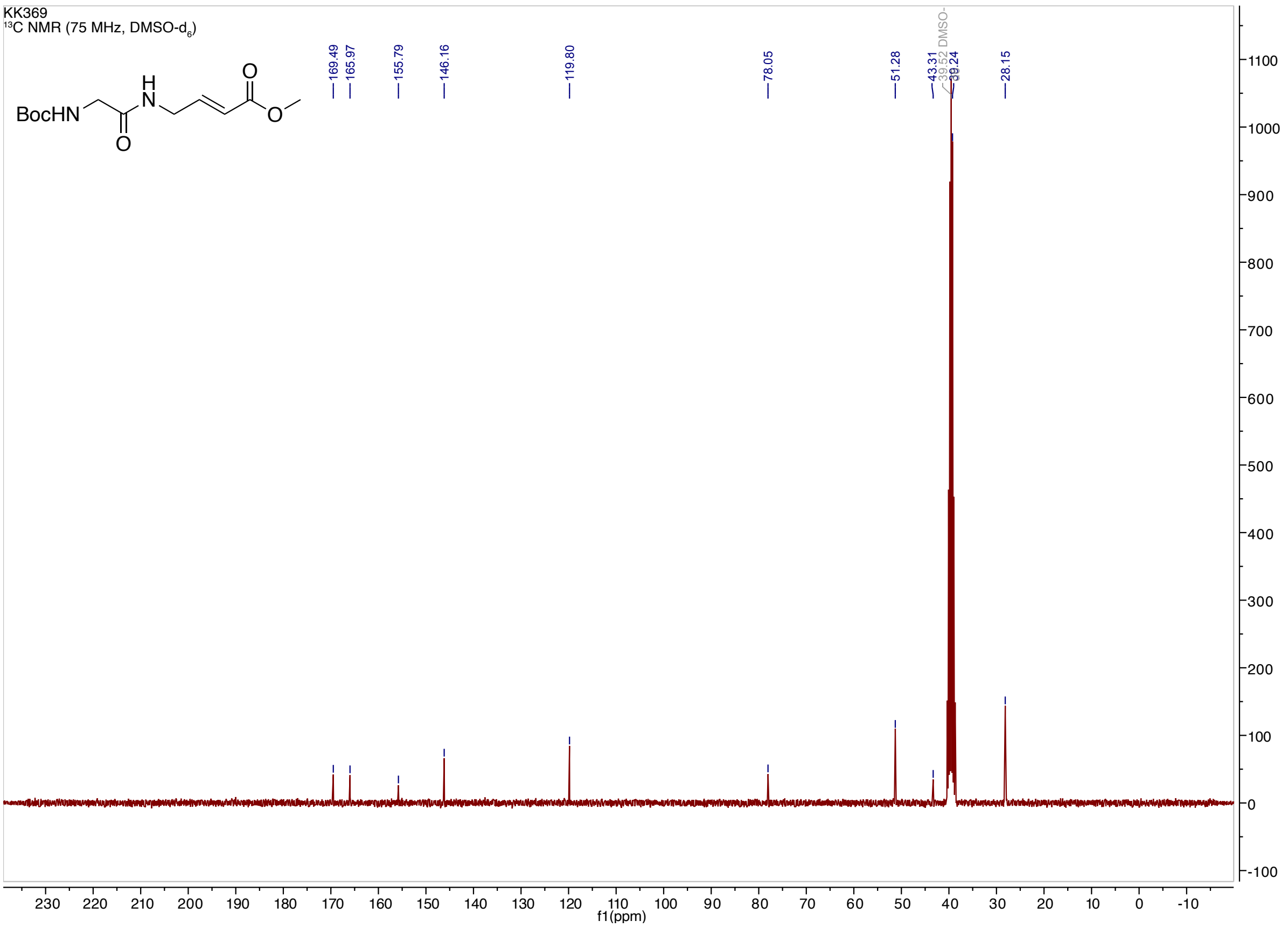
KK369
13C NMR (75 MHz, DMSO-d6)
BocHN
H
N
O
O
O
169.49
165.97
155.79
146.16
119.80
78.05
51.28
43.31
39.52 DMSO-
39.24
28.15
f1 (ppm)

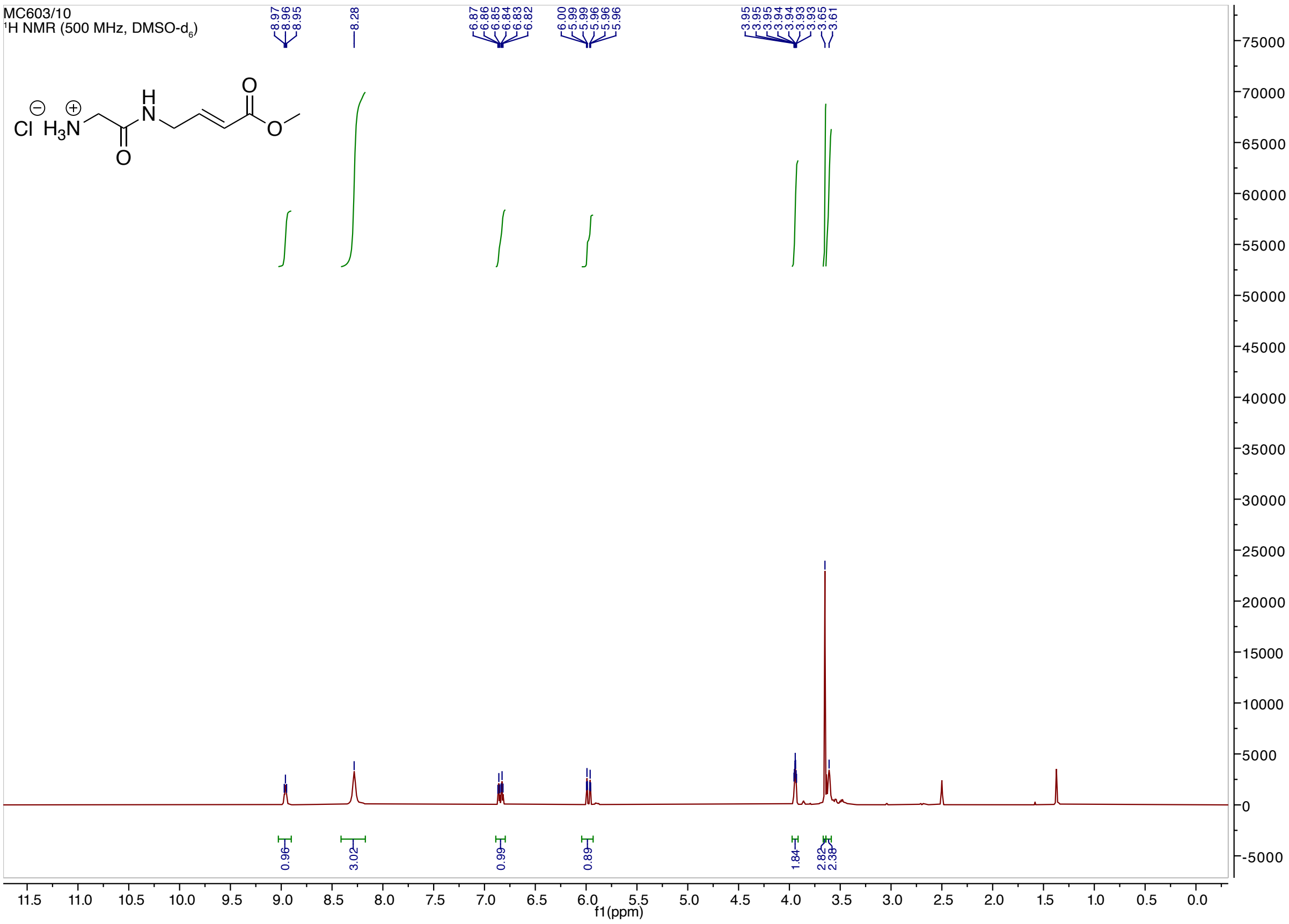

MC603/10
¹H NMR (500 MHz, DMSO-d6)

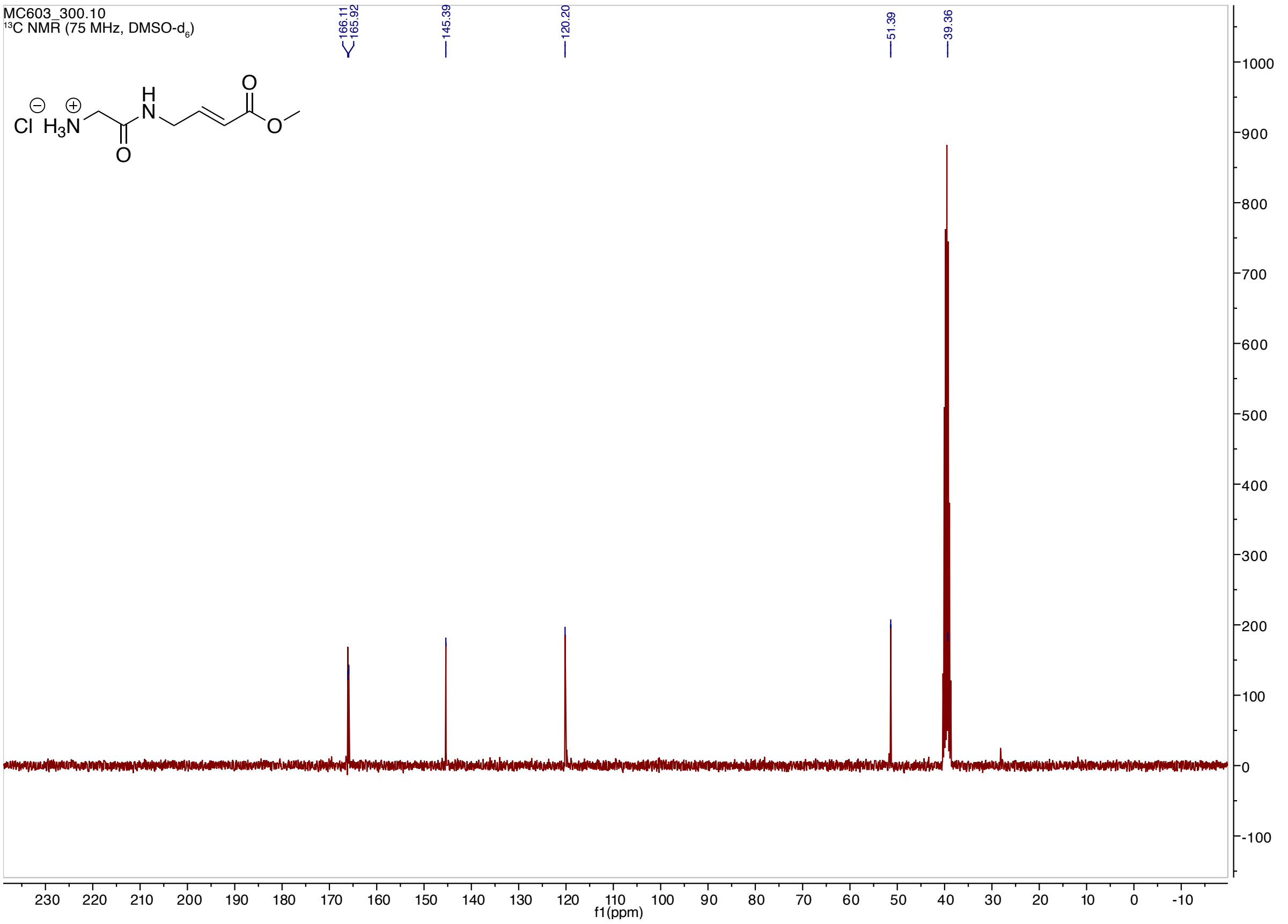

MC603_300.10
¹³C NMR (75 MHz, DMSO-d6)

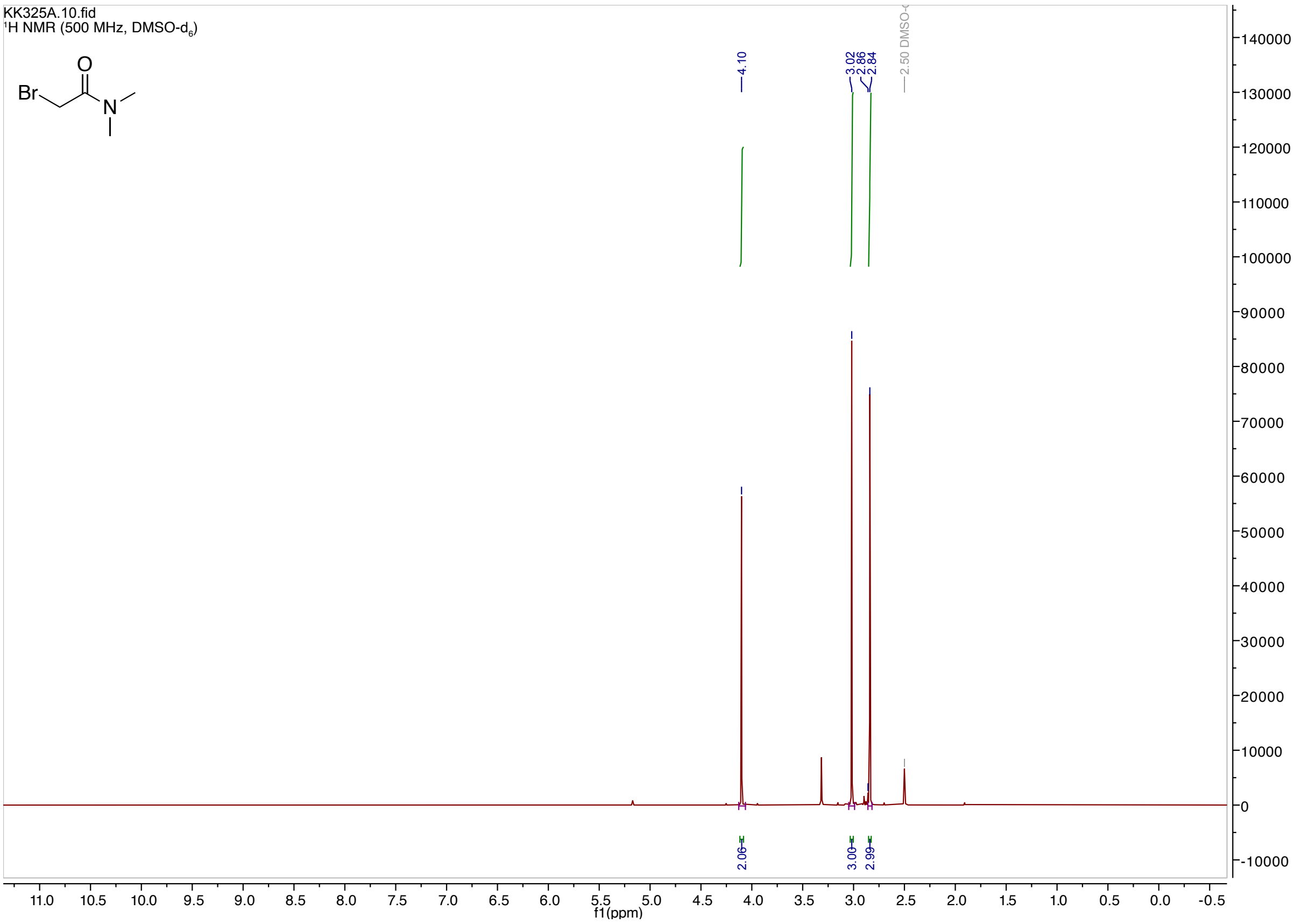
KK325A.10.fid
¹H NMR (500 MHz, DMSO-d6)
4.10
3.02
2.86
2.84
2.50 DMSO-d
2.06
3.00
2.99
f1 (ppm)

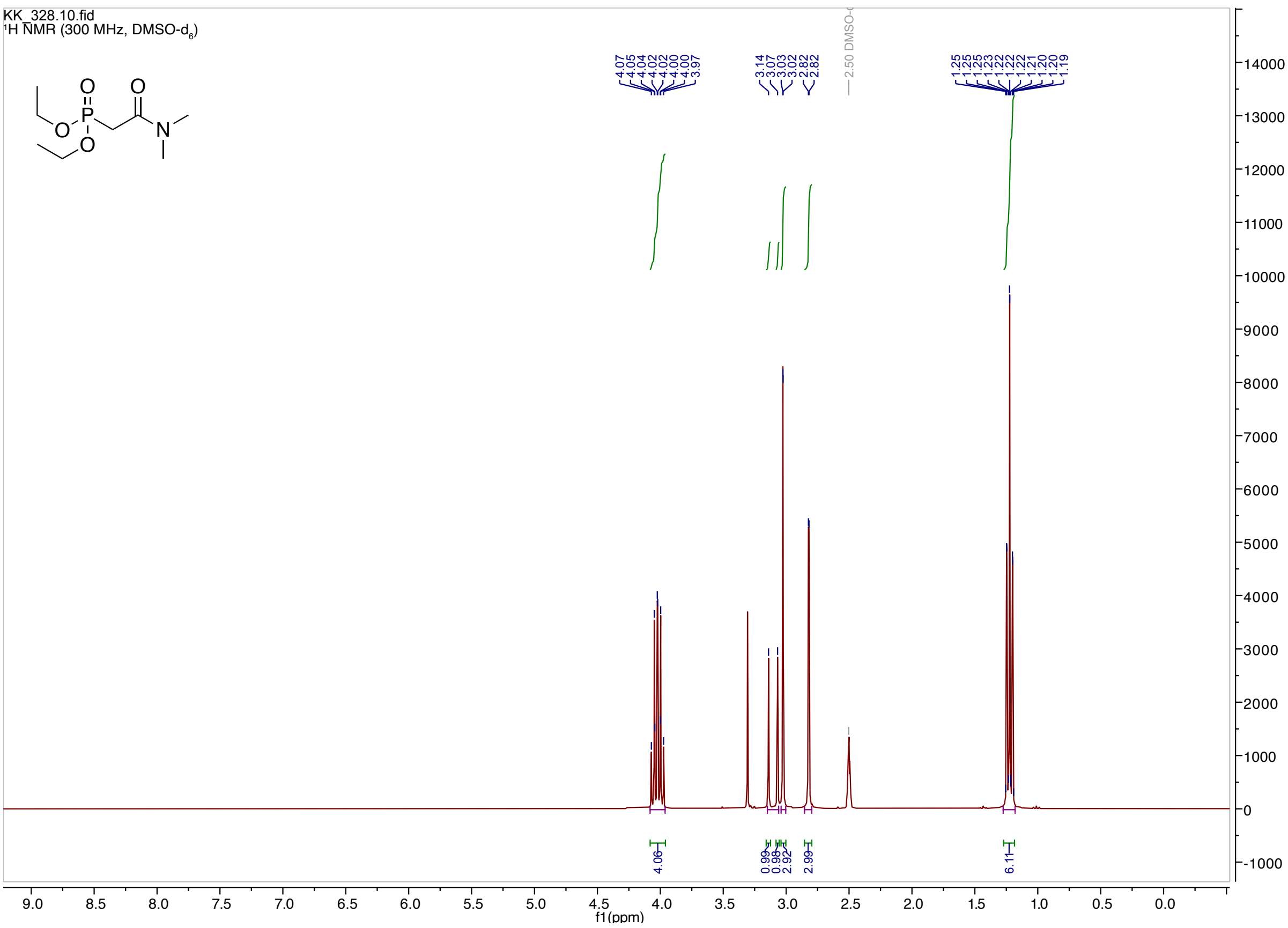
KK_328.10.fid
¹H NMR (300 MHz, DMSO-d6)
4.07
4.05
4.04
4.02
4.02
4.00
4.00
3.97
3.14
3.07
3.03
3.02
2.82
2.82
2.50 DMSO-d
1.25
1.25
1.23
1.23
1.22
1.22
1.22
1.21
1.20
1.20
1.19
4.06
0.99
0.98
2.92
2.99
6.11
f1 (ppm)

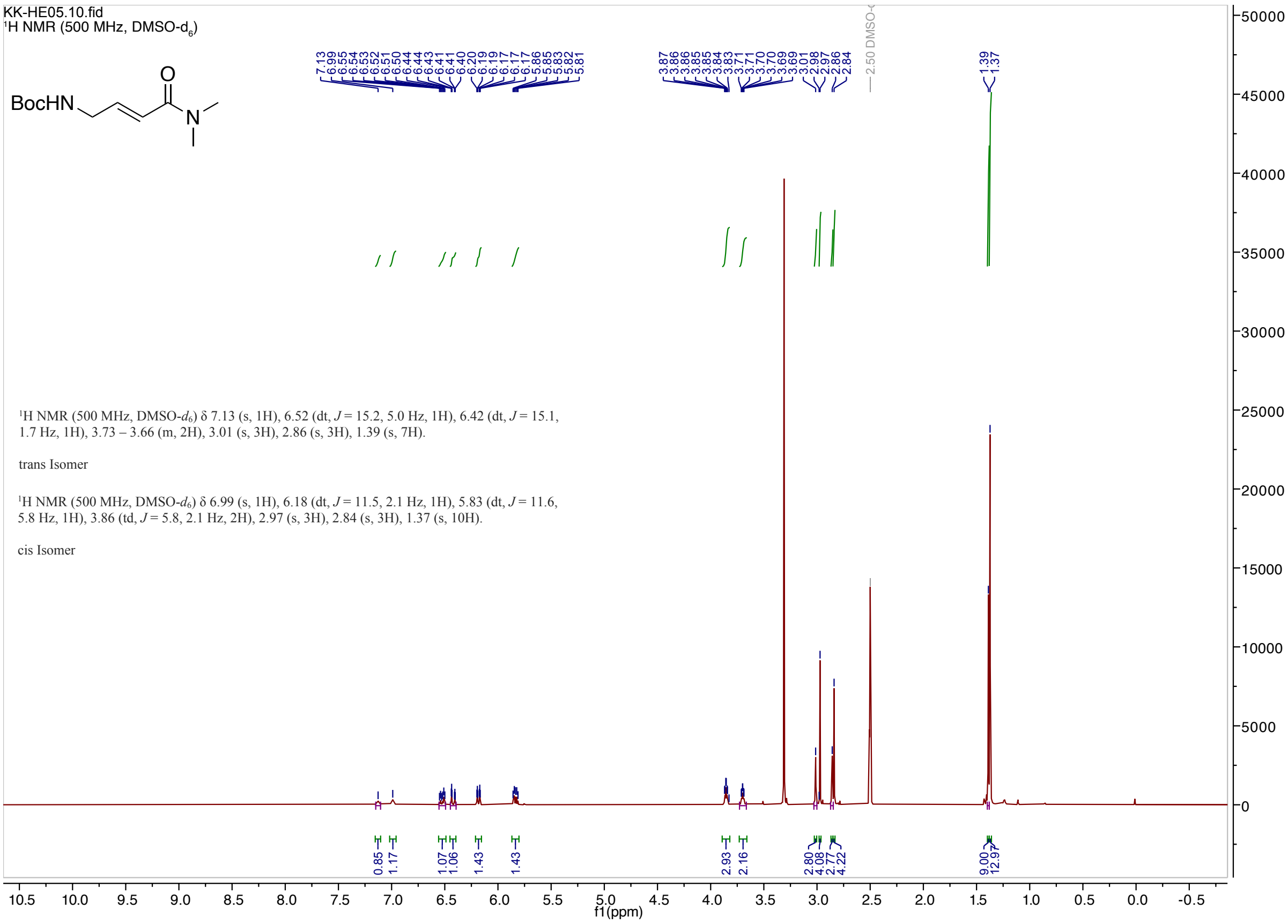
KK-HE05.10.fid
¹H NMR (500 MHz, DMSO-d6)
BocHN
O
N
¹H NMR (500 MHz, DMSO-d_6) δ 7.13 (s, 1H), 6.52 (dt, J = 15.2, 5.0 Hz, 1H), 6.42 (dt, J = 15.1, 1.7 Hz, 1H), 3.73 – 3.66 (m, 2H), 3.01 (s, 3H), 2.86 (s, 3H), 1.39 (s, 7H).
trans Isomer
¹H NMR (500 MHz, DMSO-d_6) δ 6.99 (s, 1H), 6.18 (dt, J = 11.5, 2.1 Hz, 1H), 5.83 (dt, J = 11.6, 5.8 Hz, 1H), 3.86 (td, J = 5.8, 2.1 Hz, 2H), 2.97 (s, 3H), 2.84 (s, 3H), 1.37 (s, 10H).
cis Isomer
f1(ppm)

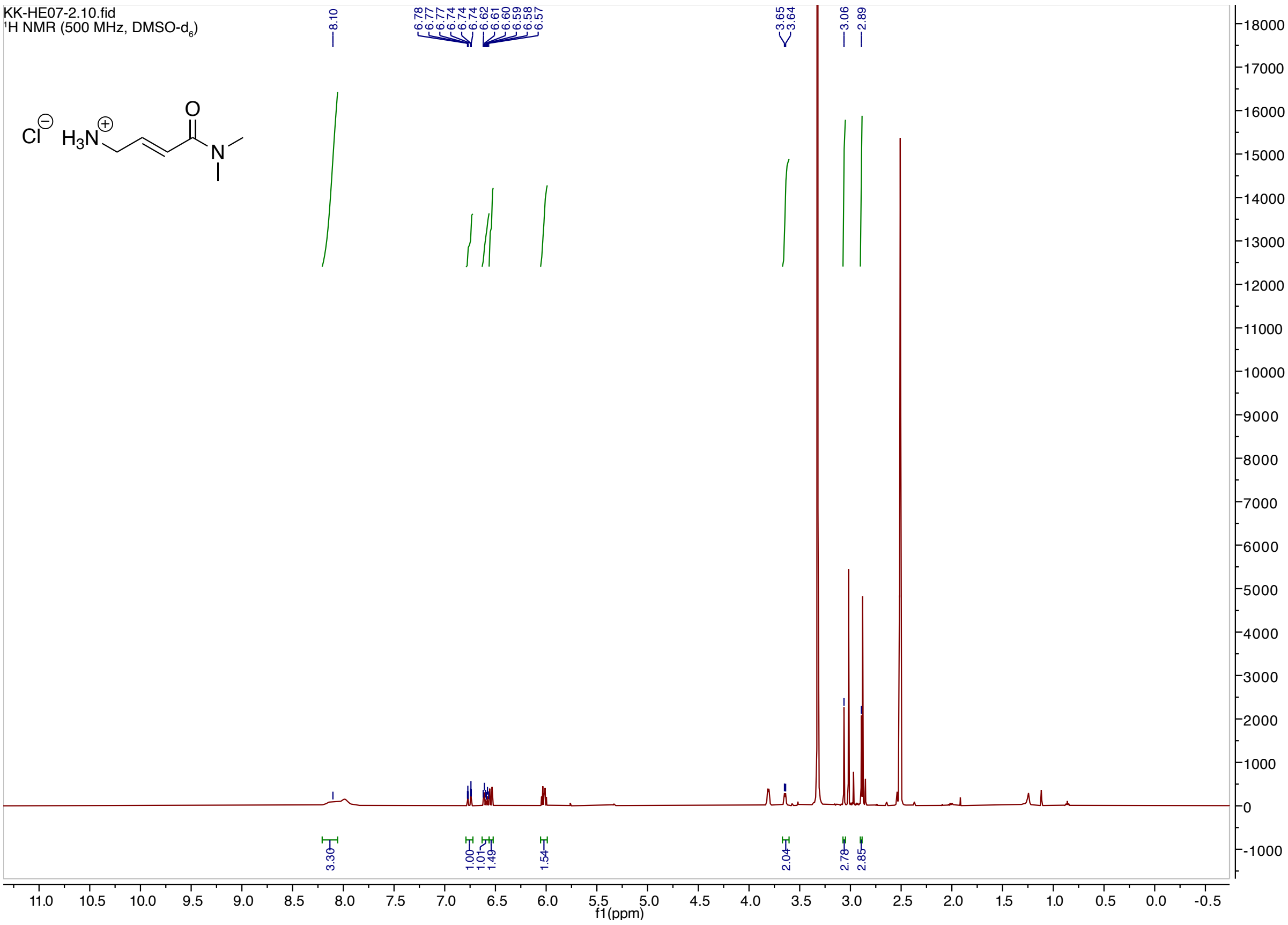
KK-HE07-2.10.fid
¹H NMR (500 MHz, DMSO-d6)
Cl⊖ H3N⊕
O
N
f1(ppm)

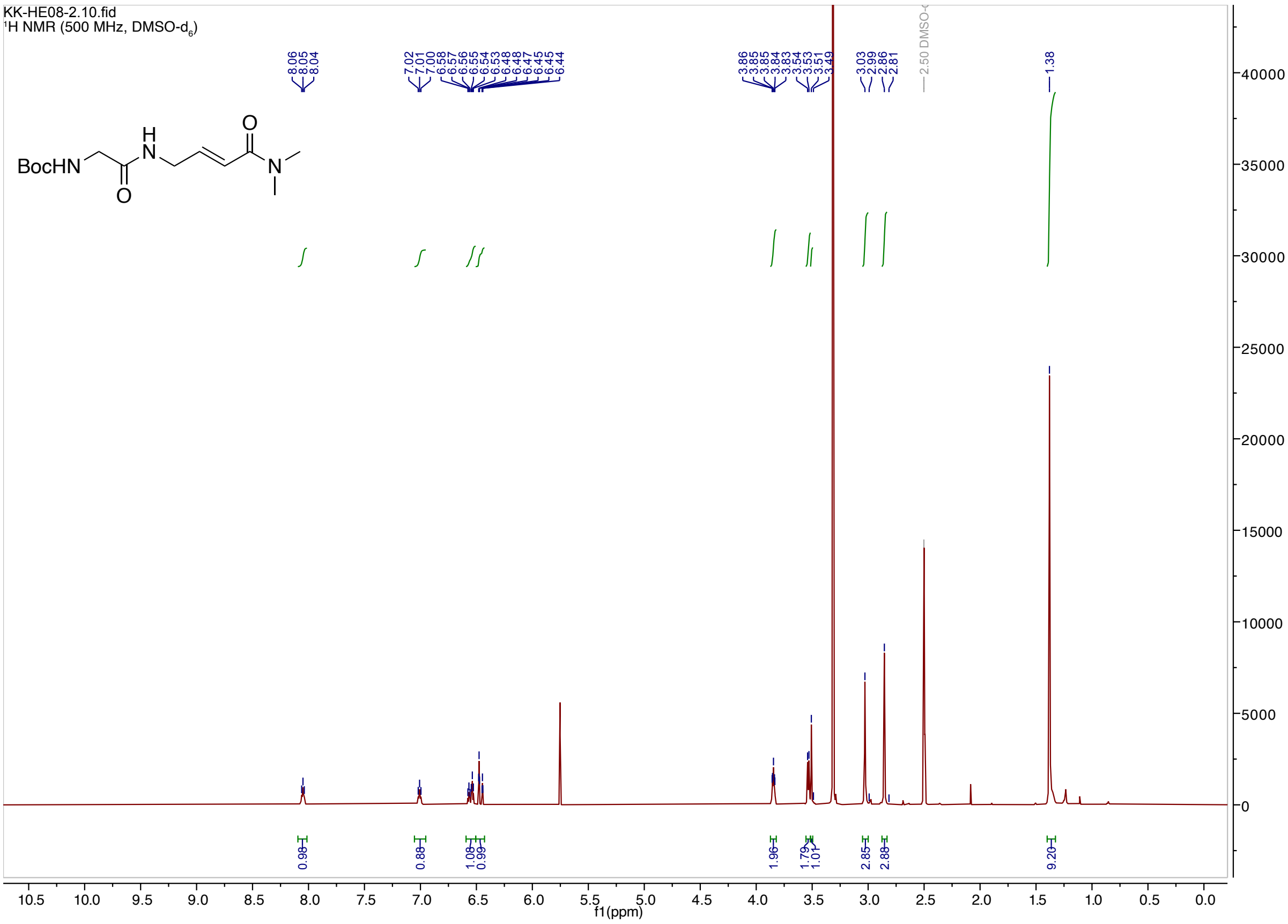
KK-HE08-2.10.fid
1H NMR (500 MHz, DMSO-d6)
BocHN
f1(ppm)

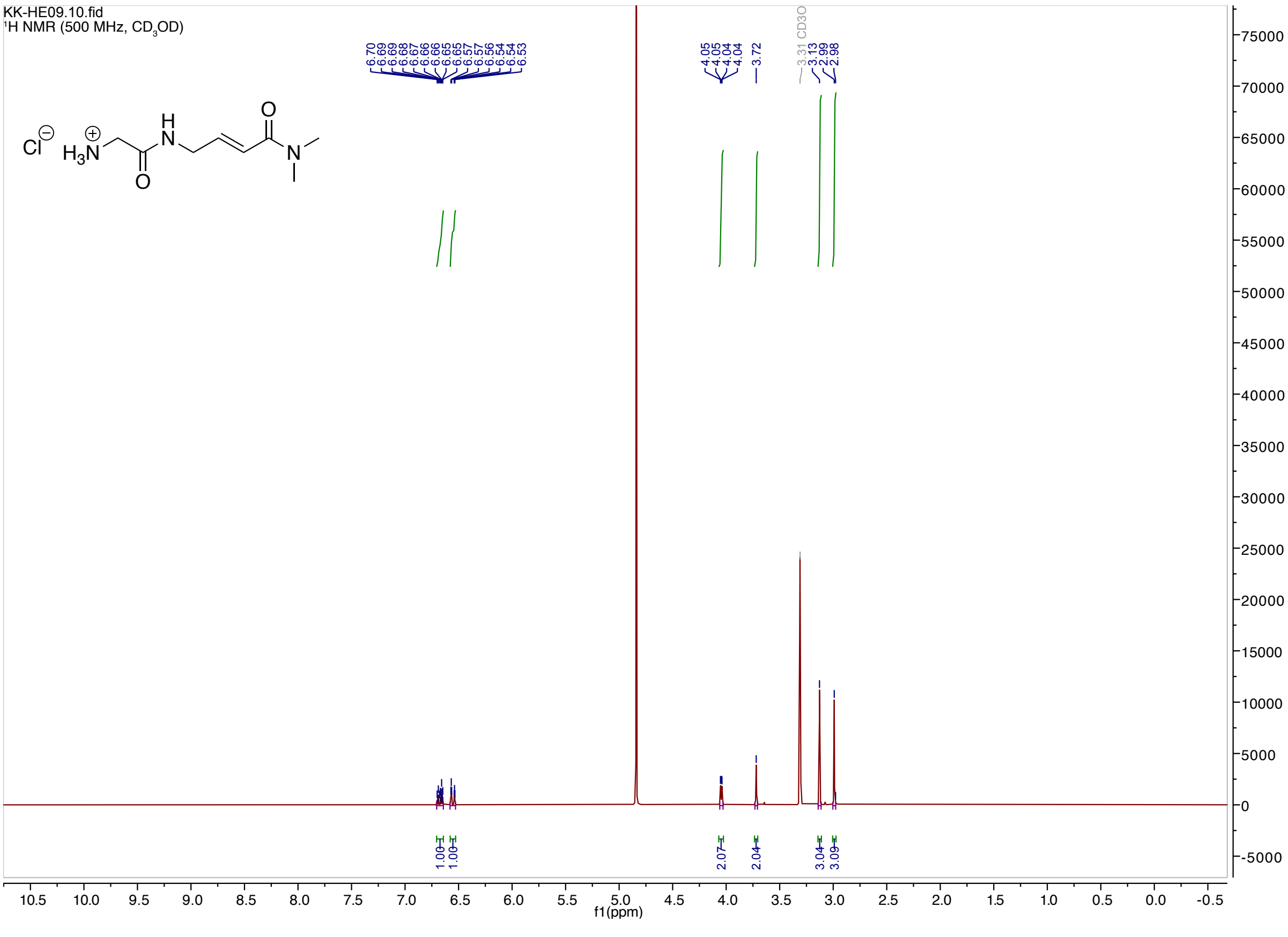
KK-HE09.10.fid
1H NMR (500 MHz, CD3OD)
f1(ppm)

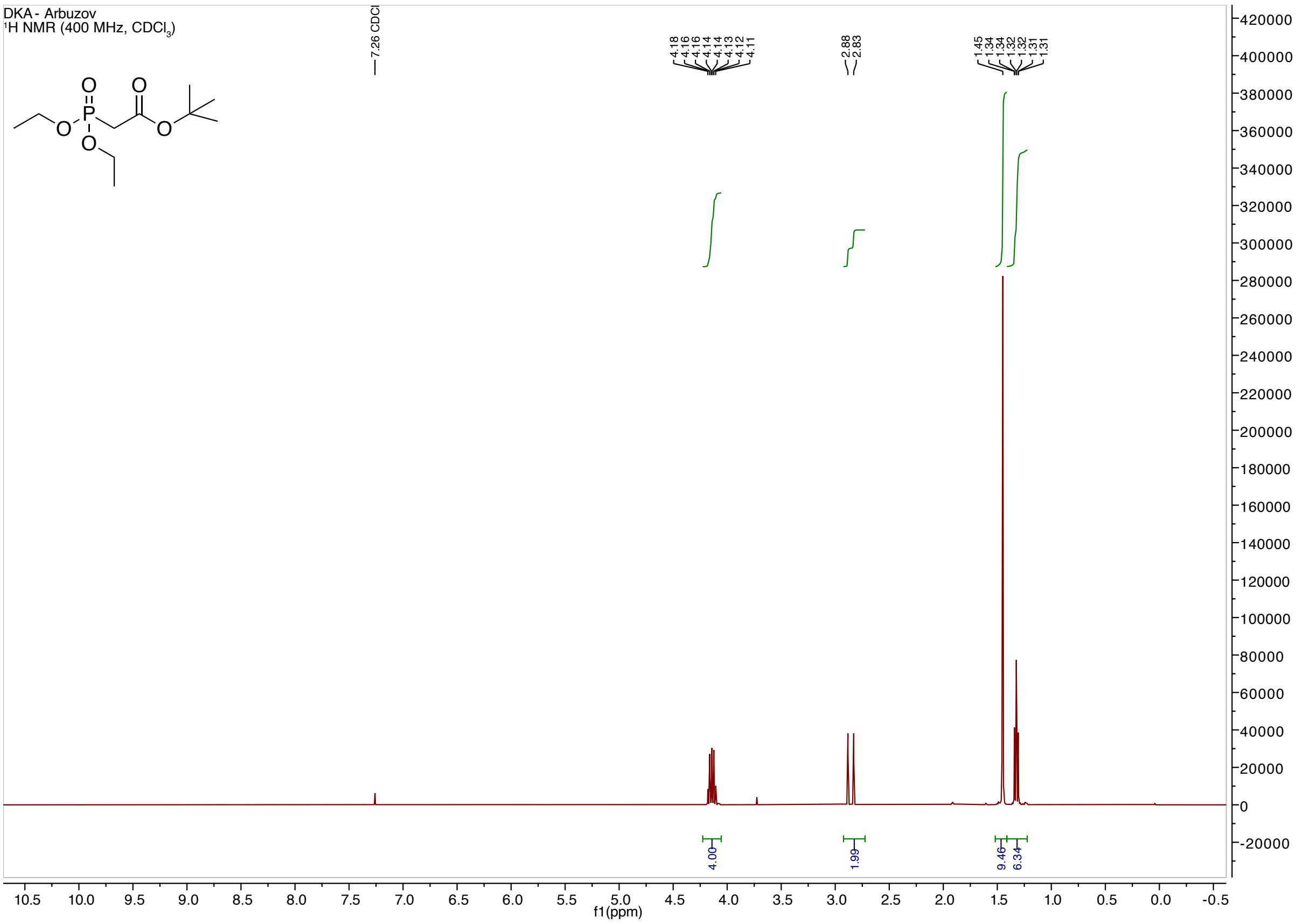
DKA - Arbuzov
1H NMR (400 MHz, CDCl3)

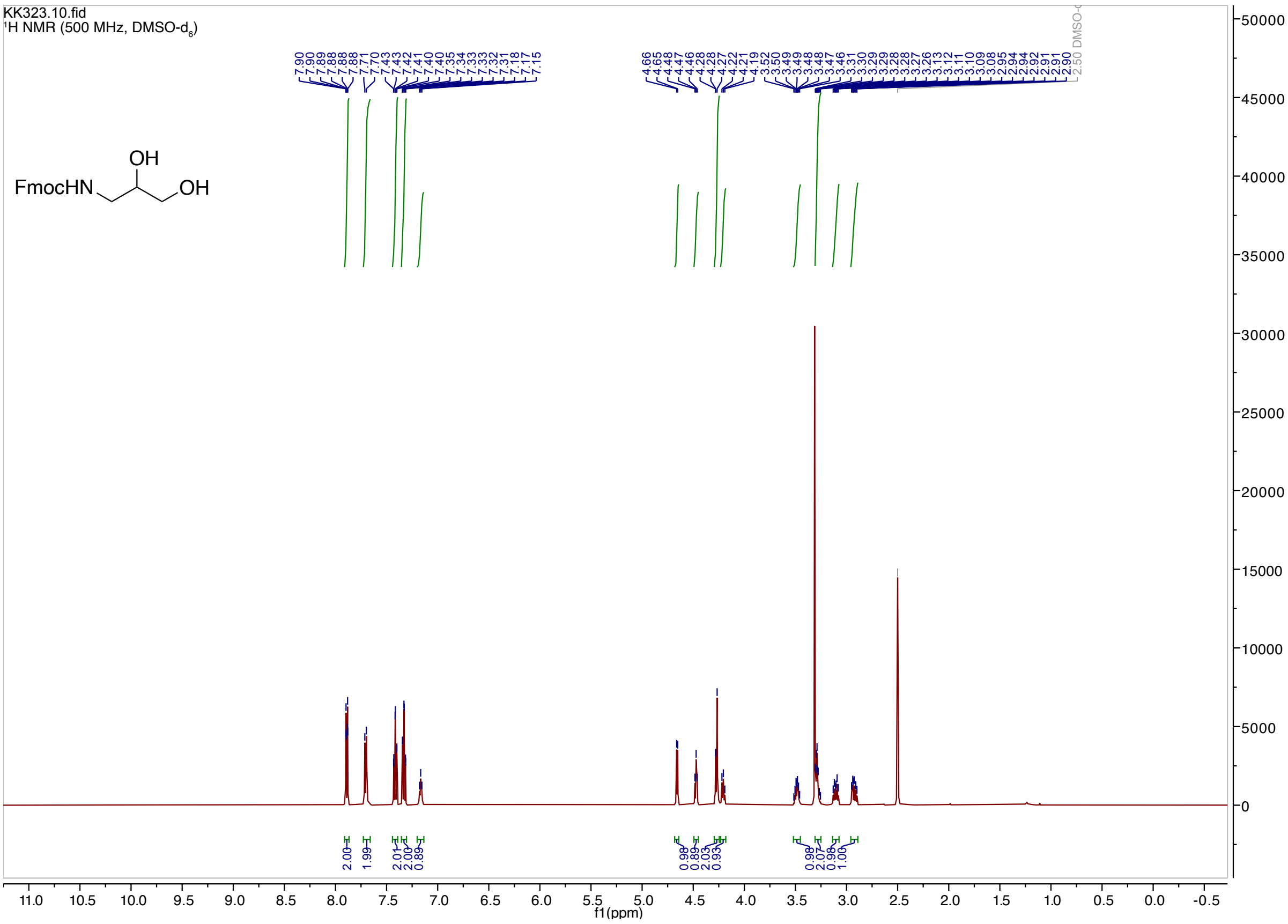
KK323.10.fid
1H NMR (500 MHz, DMSO-d6)
FmocHN
OH
OH

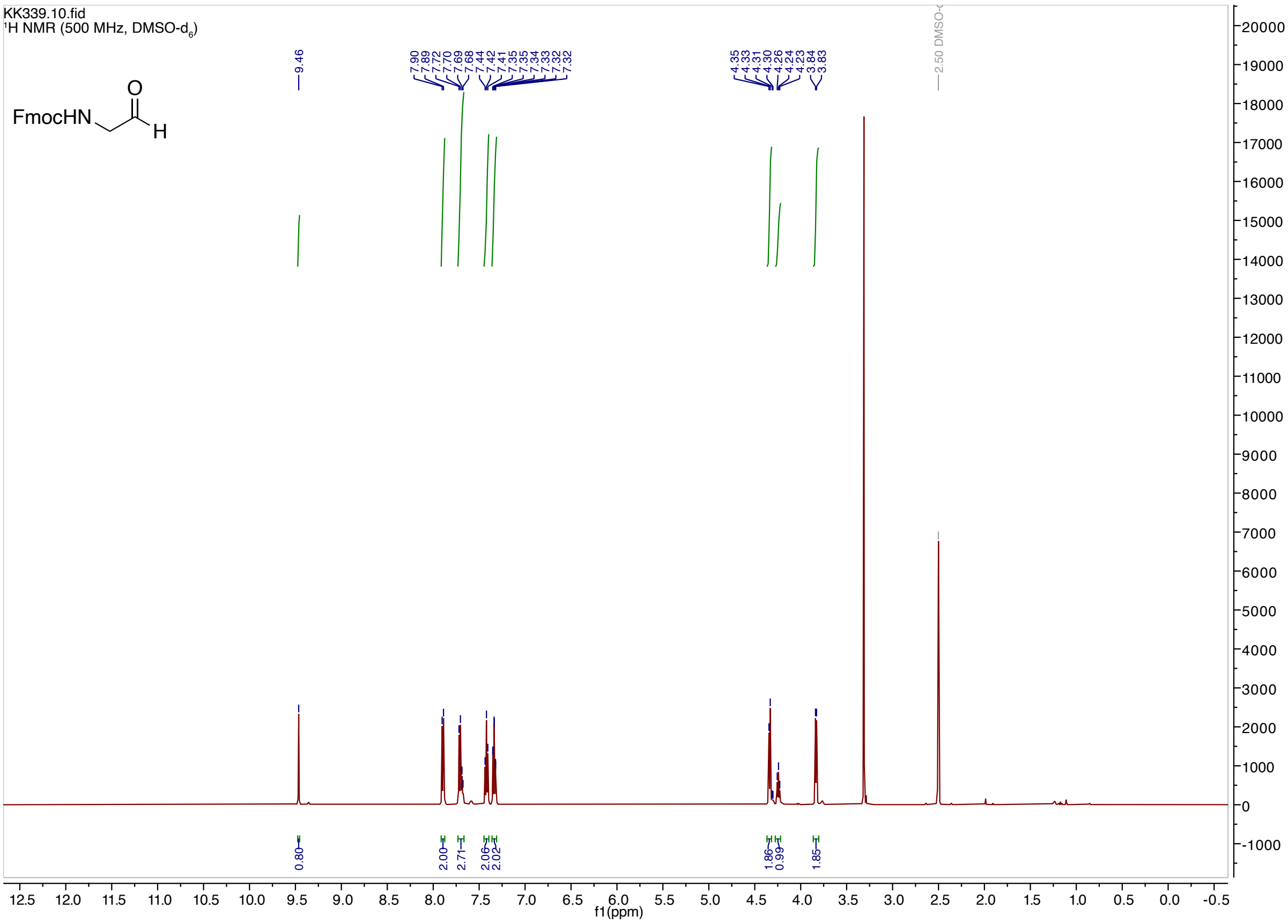
KK339.10.fid
¹H NMR (500 MHz, DMSO-d6)
FmocHN
O
H
f1 (ppm)

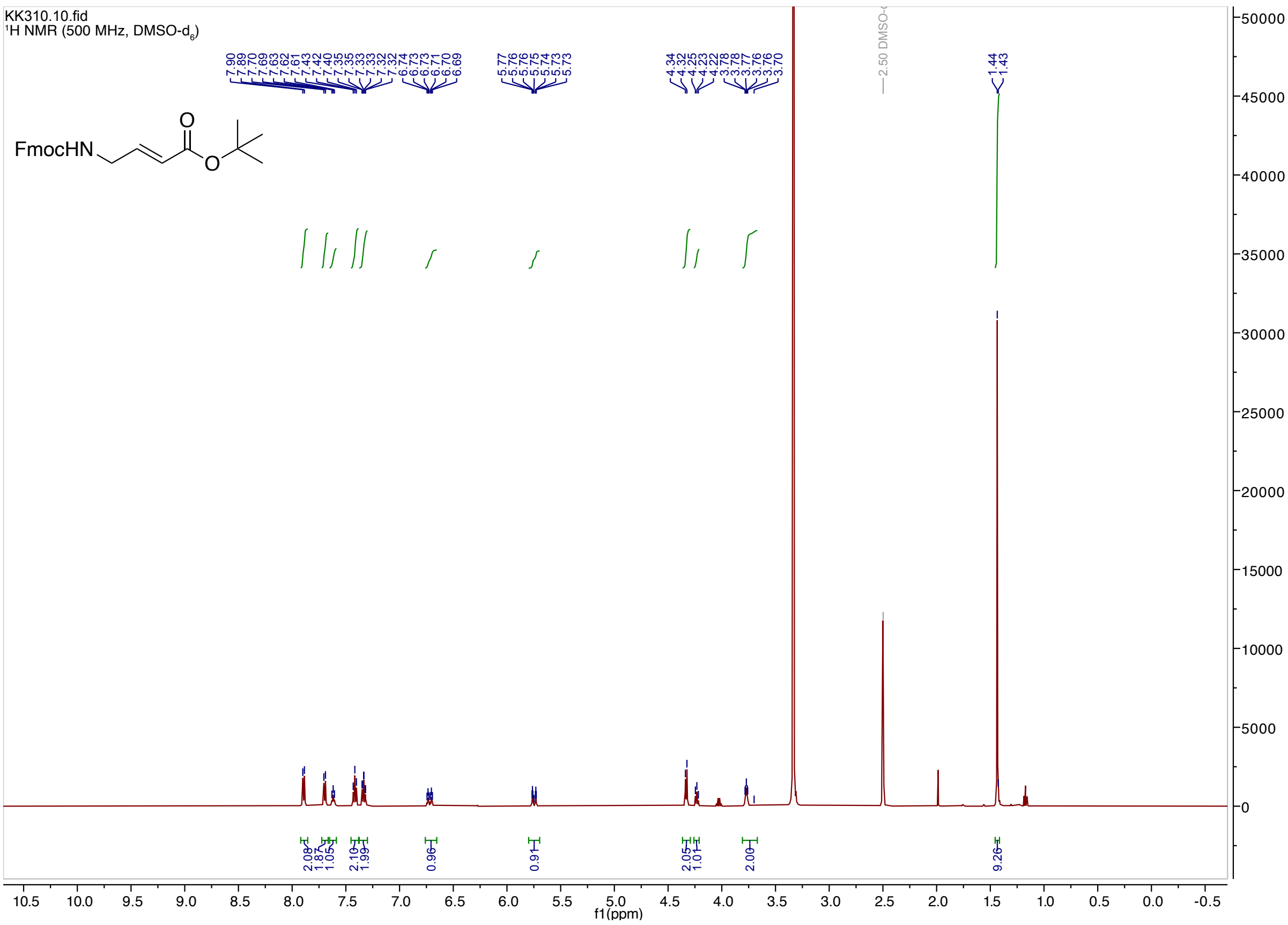
KK310.10.fid
¹H NMR (500 MHz, DMSO-d6)
FmocHN
O
O
f1 (ppm)

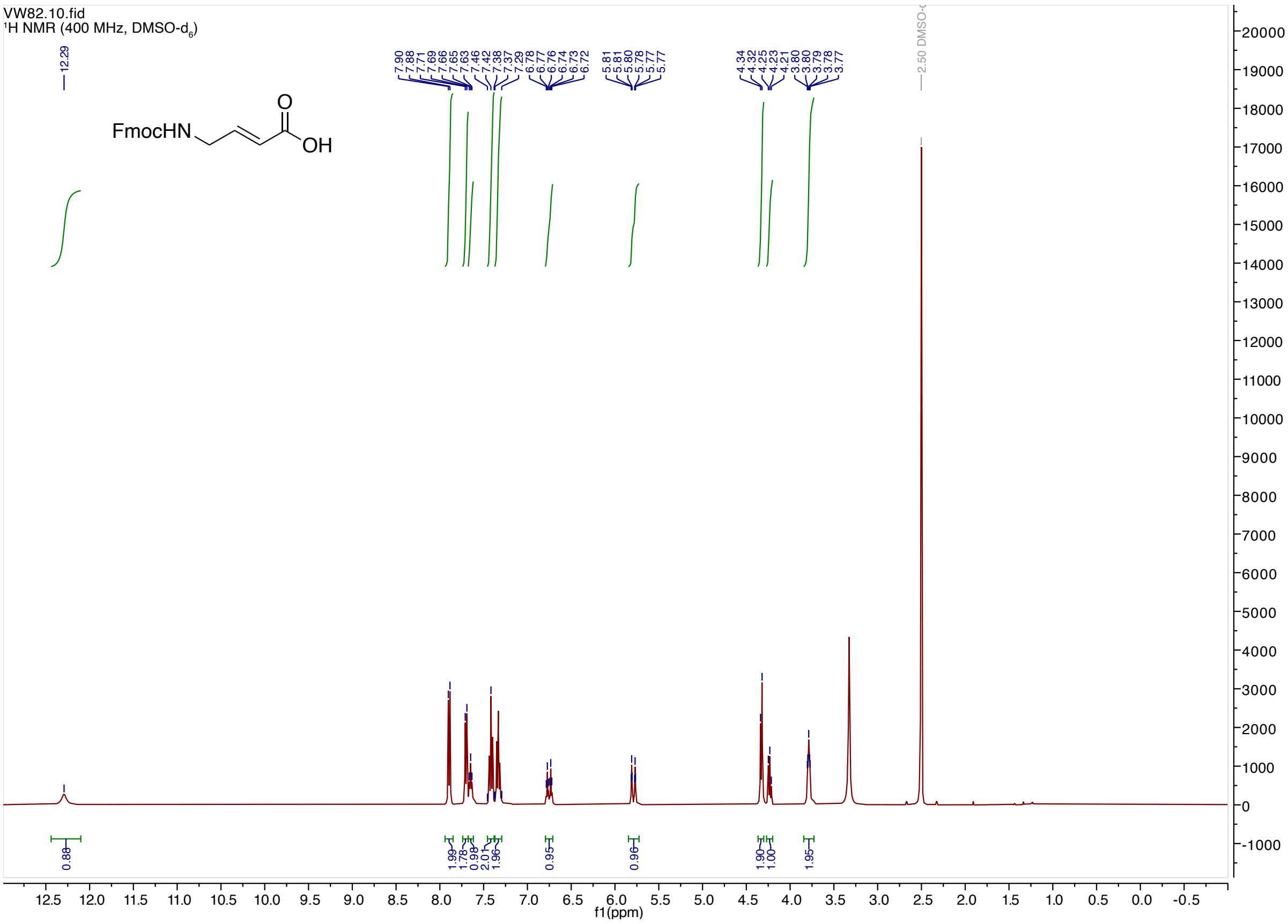
VW82.10.fid
¹H NMR (400 MHz, DMSO-d6)
FmocHN
O
OH
f1(ppm)

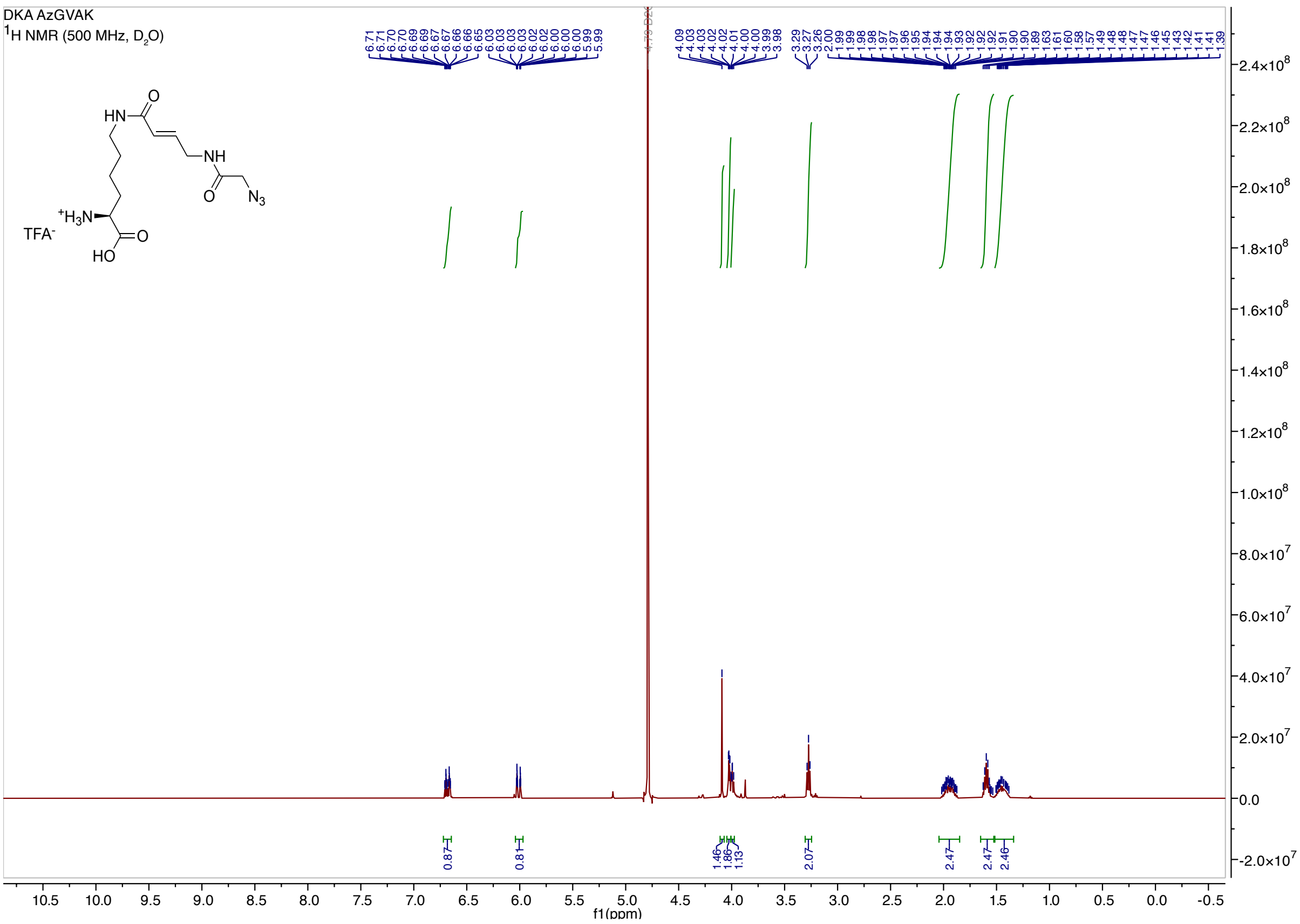
DKA AzGVAK
¹H NMR (500 MHz, D2O)
HN
O
NH
O
N3
+H3N
TFA-
O
HO
f1(ppm)

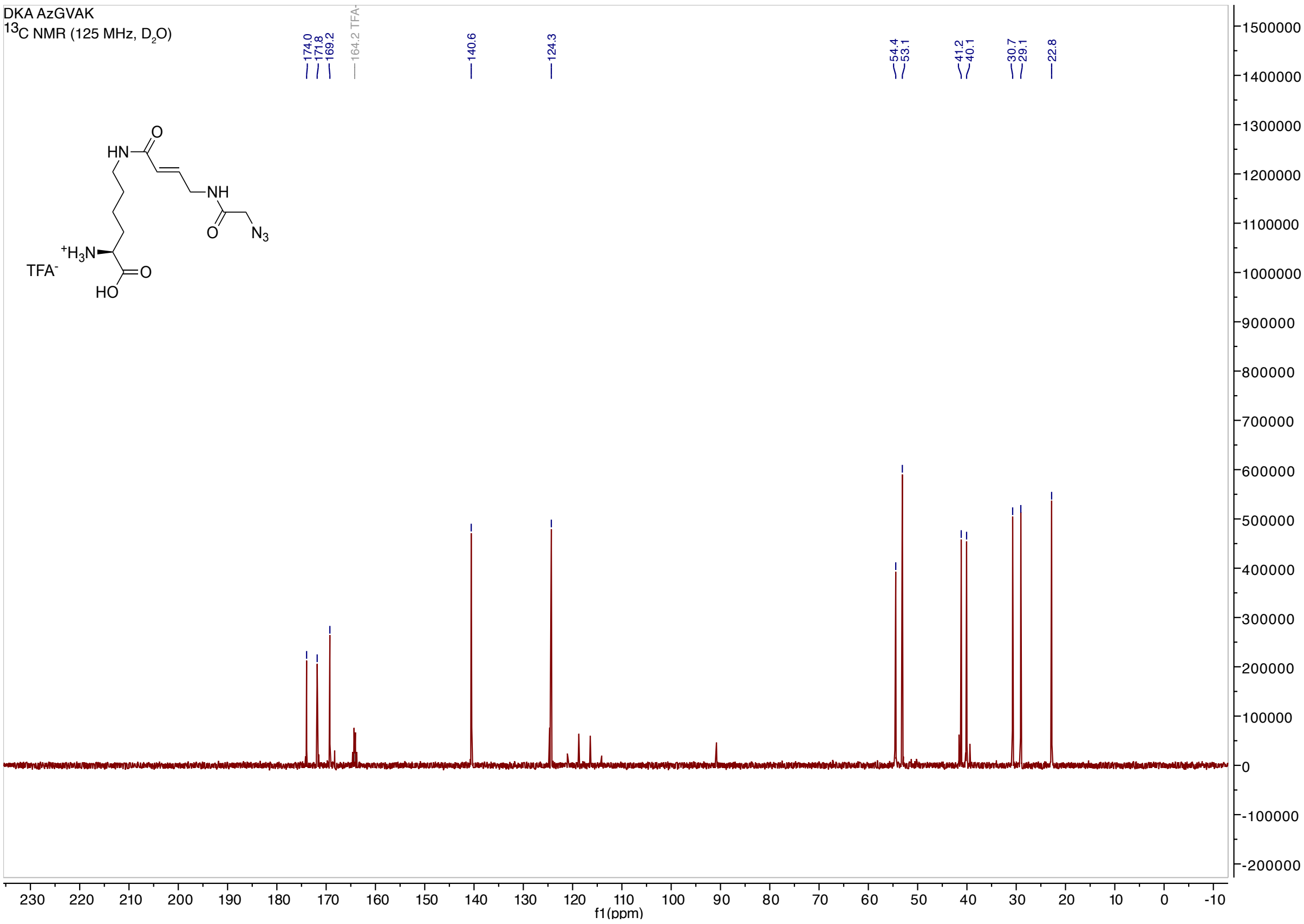
DKA AzGVAK
13C NMR (125 MHz, D2O)
174.0
171.8
169.2
164.2 TFA
140.6
124.3
54.4
53.1
41.2
40.1
30.7
29.1
22.8
HN
O
NH
O
N3
+H3N
TFA-
HO
O
f1(ppm)

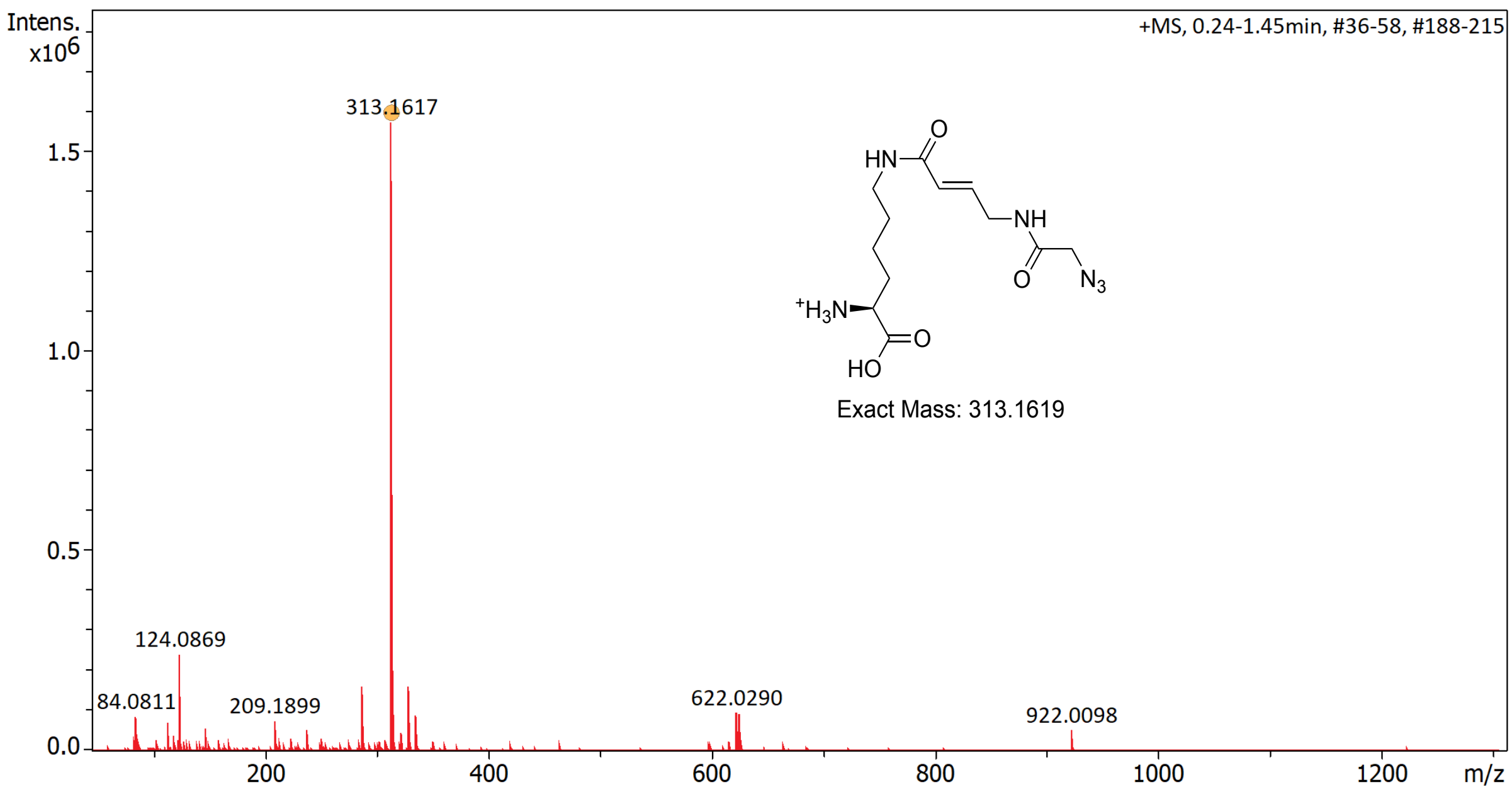

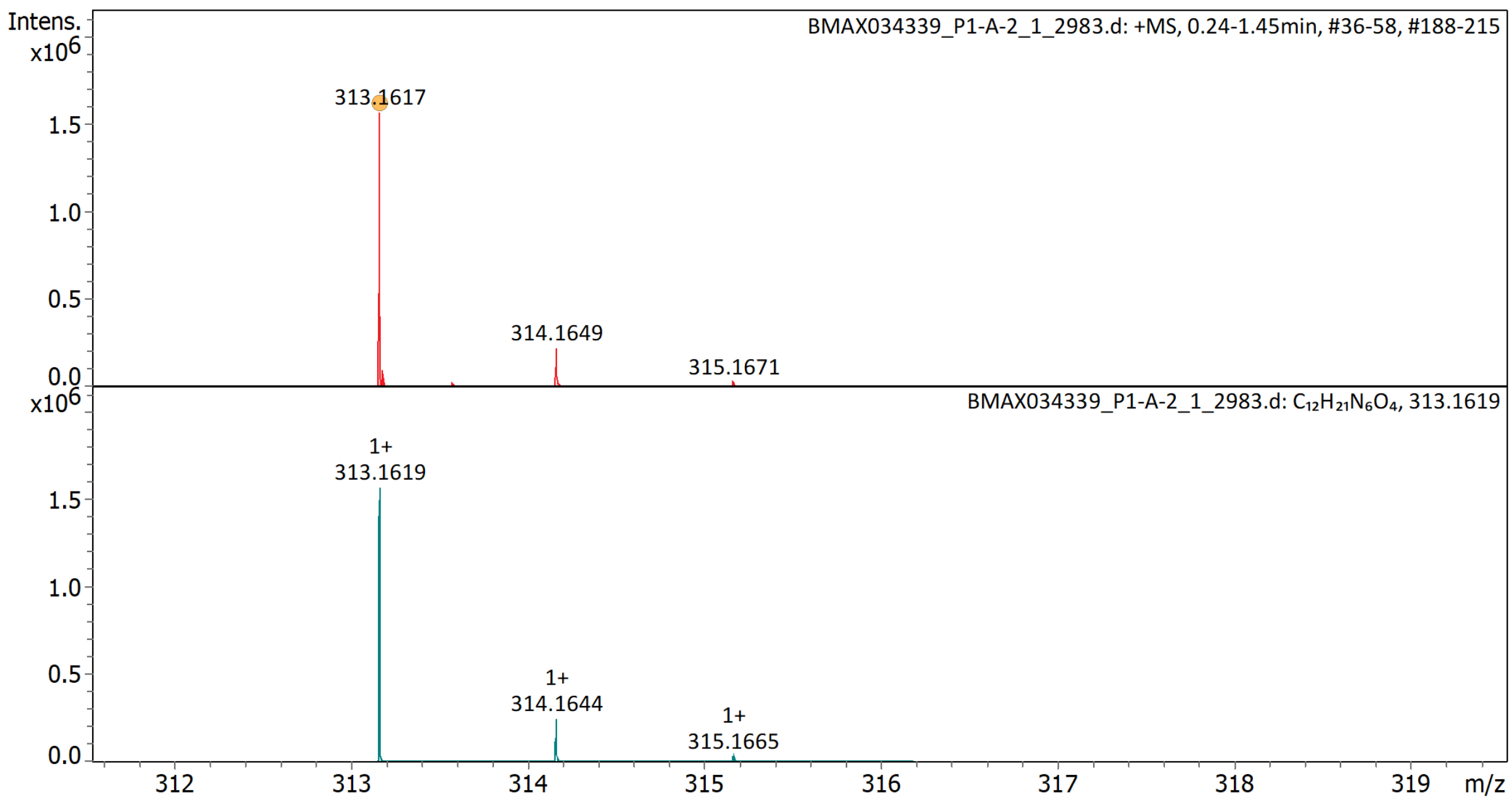

Supplementary Information References

(1) Hammill, J. T.; Miyake-Stoner, S.; Hazen, J. L.; Jackson, J. C.; Mehl, R. A. Preparation of Site-Specifically Labeled Fluorinated Proteins for 19f-Nmr Structural Characterization. *Nat Protoc* **2007**, *2*, 2601-2607. DOI: 10.1038/nprot.2007.379

(2) Kabsch, W. Xds. *Acta Crystallogr D Biol Crystallogr* **2010**, *66*, 125-132. DOI: 10.1107/S0907444909047337

(3) Agirre, J.; Atanasova, M.; Bagdonas, H.; Ballard, C. B.; Basle, A.; Beilsten-Edmands, J.; Borges, R. J.; Brown, D. G.; Burgos-Marmol, J. J.; Berrisford, J. M.; et al. The Ccp4 Suite: Integrative Software for Macromolecular Crystallography. *Acta Crystallogr D Struct Biol* **2023**, *79*, 449-461. DOI: 10.1107/S2059798323003595

(4) Kavran, J. M.; Gundllapalli, S.; O'Donoghue, P.; Englert, M.; Soll, D.; Steitz, T. A. Structure of Pyrrolysyl-Trna Synthetase, an Archaeal Enzyme for Genetic Code Innovation. *Proc Natl Acad Sci U S A* **2007**, *104*, 11268-11273. DOI: 10.1073/pnas.0704769104
(5) Emsley, P.; Lohkamp, B.; Scott, W. G.; Cowtan, K. Features and Development of Coot. *Acta Crystallogr D Biol Crystallogr* **2010**, *66*, 486-501. DOI: 10.1107/S0907444910007493
(6) Murshudov, G. N.; Skubak, P.; Lebedev, A. A.; Pannu, N. S.; Steiner, R. A.; Nicholls, R. A.; Winn, M. D.; Long, F.; Vagin, A. A. Refmac5 for the Refinement of Macromolecular Crystal Structures. *Acta Crystallogr D Biol Crystallogr* **2011**, *67*, 355-367. DOI: 10.1107/S0907444911001314
(7) Morris, R. J.; Perrakis, A.; Lamzin, V. S. Arp/Warp and Automatic Interpretation of Protein Electron Density Maps. *Methods Enzymol* **2003**, *374*, 229-244. DOI: 10.1016/S0076-6879(03)74011-7
(8) Williams, C. J.; Headd, J. J.; Moriarty, N. W.; Prisant, M. G.; Videau, L. L.; Deis, L. N.; Verma, V.; Keedy, D. A.; Hintze, B. J.; Chen, V. B.; et al. Molprobity: More and Better Reference Data for Improved All-Atom Structure Validation. *Protein Sci* **2018**, *27*, 293-315. DOI: 10.1002/pro.3330
(9) Fottner, M.; Brunner, A. D.; Bittl, V.; Horn-Ghetko, D.; Jussupow, A.; Kaila, V. R. I.; Bremm, A.; Lang, K. Site-Specific Ubiquitylation and Sumoylation Using Genetic-Code Expansion and Sortase. *Nat Chem Biol* **2019**, *15*, 276-284. DOI: 10.1038/s41589-019-0227-4
(10) Dorr, B. M.; Ham, H. O.; An, C.; Chaikof, E. L.; Liu, D. R. Reprogramming the Specificity of Sortase Enzymes. *Proc Natl Acad Sci U S A* **2014**, *111*, 13343-13348. DOI: 10.1073/pnas.1411179111
(11) Mikolajczyk, J.; Drag, M.; Bekes, M.; Cao, J. T.; Ronai, Z.; Salvesen, G. S. Small Ubiquitin-Related Modifier (Sumo)-Specific Proteases: Profiling the Specificities and Activities of Human Senps. *J Biol Chem* **2007**, *282*, 26217-26224. DOI: 10.1074/jbc.M702444200
(12) Cheng, J.; Kang, X.; Zhang, S.; Yeh, E. T. Sumo-Specific Protease 1 Is Essential for Stabilization of Hif1alpha During Hypoxia. *Cell* **2007**, *131*, 584-595. DOI: 10.1016/j.cell.2007.08.045
